# Mapping access to domestic water supplies from incomplete data in developing countries: An illustrative assessment for Kenya

**DOI:** 10.1371/journal.pone.0216923

**Published:** 2019-05-17

**Authors:** Weiyu Yu, Nicola A. Wardrop, Robert E. S. Bain, Victor Alegana, Laura J. Graham, Jim A. Wright

**Affiliations:** 1 University of Southampton, Southampton, Hampshire, United Kingdom; 2 Division of Data, Research and Policy, United Nations Children's Fund (UNICEF), New York, New York, United States of America; 3 Population Health Unit, Kenya Medical Research Institute—Wellcome Trust Research Programme, Nairobi, Kenya; Leiden University, NETHERLANDS

## Abstract

Water point mapping databases, generated through surveys of water sources such as wells and boreholes, are now available in many low and middle income countries, but often suffer from incomplete coverage. To address the partial coverage in such databases and gain insights into spatial patterns of water resource use, this study investigated the use of a maximum entropy (MaxEnt) approach to predict the geospatial distribution of drinking-water sources, using two types of unimproved sources in Kenya as illustration. Geographic locations of unprotected dug wells and surface water sources derived from the Water Point Data Exchange (WPDx) database were used as inputs to the MaxEnt model alongside geological/hydrogeological and socio-economic covariates. Predictive performance of the MaxEnt models was high (all > 0.9) based on Area Under the Receiver Operator Curve (AUC), and the predicted spatial distribution of water point was broadly consistent with household use of these unimproved drinking-water sources reported in household survey and census data. In developing countries where geospatial datasets concerning drinking-water sources often have necessarily limited resolution or incomplete spatial coverage, the modelled surface can provide an initial indication of the geography of unimproved drinking-water sources to target unserved populations and assess water source vulnerability to contamination and hazards.

## 1. Introduction

Access to water is a basic human necessity and plays a key role in well-being and sustainable development [1]. Following the recognition of the human right to drinking-water [2], the United Nations’ Sustainable Development Goals (SDGs) included a target that aims to achieve universal and equitable access to safe and affordable drinking-water by 2030 (Goal 6 Target 6.1) [3]. Realisation of the ambitious SDG target requires robust progress monitoring to underpin development planning for service improvements and to identify disadvantaged groups, so that those with the greatest needs can be prioritised accordingly. Geographic location is often an important factor that influences access to basic housing infrastructures including water supply [4]. International guidance on monitoring of inequalities in drinking-water services therefore recommends data are disaggregated by geographic location and rurality to reveal and address sub-national disparities [5]. As such, this expanded need for detailed locational information where water is accessed for household use is likely to place greater demands on existing data sources.

Currently, there are few data sets describing the spatial distribution of access to drinking-water sources. Conventional monitoring of drinking-water services at international and national levels relies heavily on household surveys and censuses [6]. Even though several previous studies have created spatially disaggregated map layers using such data [7,8], these data sets necessarily have limited spatial resolution because of data protection needs (i.e. scrambling of household survey GPS cluster coordinates, aggregation of census data, etc.) and often focus on water sources for drinking only. Other relevant geospatial data sources include water point mapping databases, which contain geo-referenced water source locations alongside associated attributes. Water point data are not affected by data protection concerns, so have greater spatial precision than household surveys and censuses. Often, such databases capture water points used for many purposes, not solely for drinking by households. However, very often they are project-specific, focus on particular geographic areas and water source types, and are collected and stored by multiple agencies. Because of geographic gaps in project coverage and agency responsibilities, there thus remain few nationwide, complete and consistent water point datasets [9]. To gain a more complete picture of domestic water use from water point mapping, one therefore has to ‘gap-fill’ the data first.

One potential way to address concerning water points is to estimate their overall geographical distribution by predicting the probability of water point presence or relative site suitability across the landscape of interest. This problem is analogous to mapping habitat suitability from incomplete species occurrences in ecological studies. Various spatial predictive modelling techniques have been developed to address this issue, including a variety of algorithms based on bioclimatic envelopes [10,11], Gower distance [12], Mahalanobis distance [13,14], statistical regression [15–17], and machine learning [18–22]. These methods have been used to examine impacts of climate changes [23–26], invasive species [27–30], conservation assessment [31–33], and species richness [34]. In addition, they have also been used to track disease vectors [35–40], assess landslide susceptibility [41–43], map soil phosphorus [44], and wildfire risk [45–47]. Furthermore, spatial predictive modelling techniques have been combined with water point data for groundwater potential delineation for groundwater resource assessment [48–55] in recent years. However, to our knowledge, spatial predictive modelling has not yet been used for predicting the spatial distribution of infrastructure or services such as domestic water supplies by incorporating socio-economic as well as biophysical covariates.

Based on the type of observational data used, spatial predictive models can be classified as presence-only, presence-absence, and presence-background (or presence-pseudo-absence) methods. Since water point data only record observed locations of water access points, whilst the absence of a water source at a given location cannot be inferred from such records, they may be considered as presence data. In this study, we therefore employ a machine learning approach developed for spatial ecology [22], namely maximum entropy (MaxEnt) modelling, since it only requires ‘presence’ data from water point mapping enables use of categorical variables as predictors, has good predictive performance [56], user-friendly software [57,58], and is suitable for integration into a reproducible workflow [59,60]. As a novel spatial predictive modelling method, MaxEnt is more realistic than simple measures such as distance models [12,14], but more straightforward to implement than more complex methods, such as likelihood analysis [61] and hierarchical species distribution models [62]. However, despite its advantages, because MaxEnt relies on presence-only data, it lacks information on the proportion of occupied sites (i.e. prevalence) and the logistic transformation of raw outputs used by MaxEnt only represents a relative ranking rather than a true probability [63]. In this study, we adopted a MaxEnt model merely as an illustrative example of how a wider suite of techniques can make predictions of the potential geographical distribution of unimproved domestic water sources from physical and socio-economic characteristics. We used unimproved water point data to examine the feasibility of introducing this method into the water sector, assuming that the locations of observed water points reflect suitable conditions for siting such water sources. Kenya was selected as a case study, given household use of unimproved domestic water sources there [64] and availability of suitable data. The main objectives of this study are to (1) examine the potential applicability of MaxEnt modelling for predicting the geographical occupancy (or relative site suitability) of access to domestic water supplies using water point data; and (2) analyse the importance of predictive covariates that potentially explain the spatial distribution of drinking-water sources.

## 2. Methods

### 2.1 Study area

Kenya is a lower-middle income country [65] in Sub-Saharan Africa (SSA), which did not achieve the MDG drinking-water target by 2015. However, substantial progress in access to improved drinking-water sources was demonstrated during the MDG period [66]. According to the most recent Demographic and Health Survey (DHS) [67], among Kenya’s estimated population of 43 million in 2014, 31.6% were using unimproved drinking-water sources, including 7.3% using unprotected dug wells, 4.4% unprotected springs, 1.5% tanker trucks or carts with drum, and 18.4% surface water. The majority (85.7%) of the urban population had access to improved drinking-water sources, whilst nearly half (41.5%) of the rural population were using unimproved drinking-water sources.

### 2.2 Target water sources and locations

**Fig 1:**
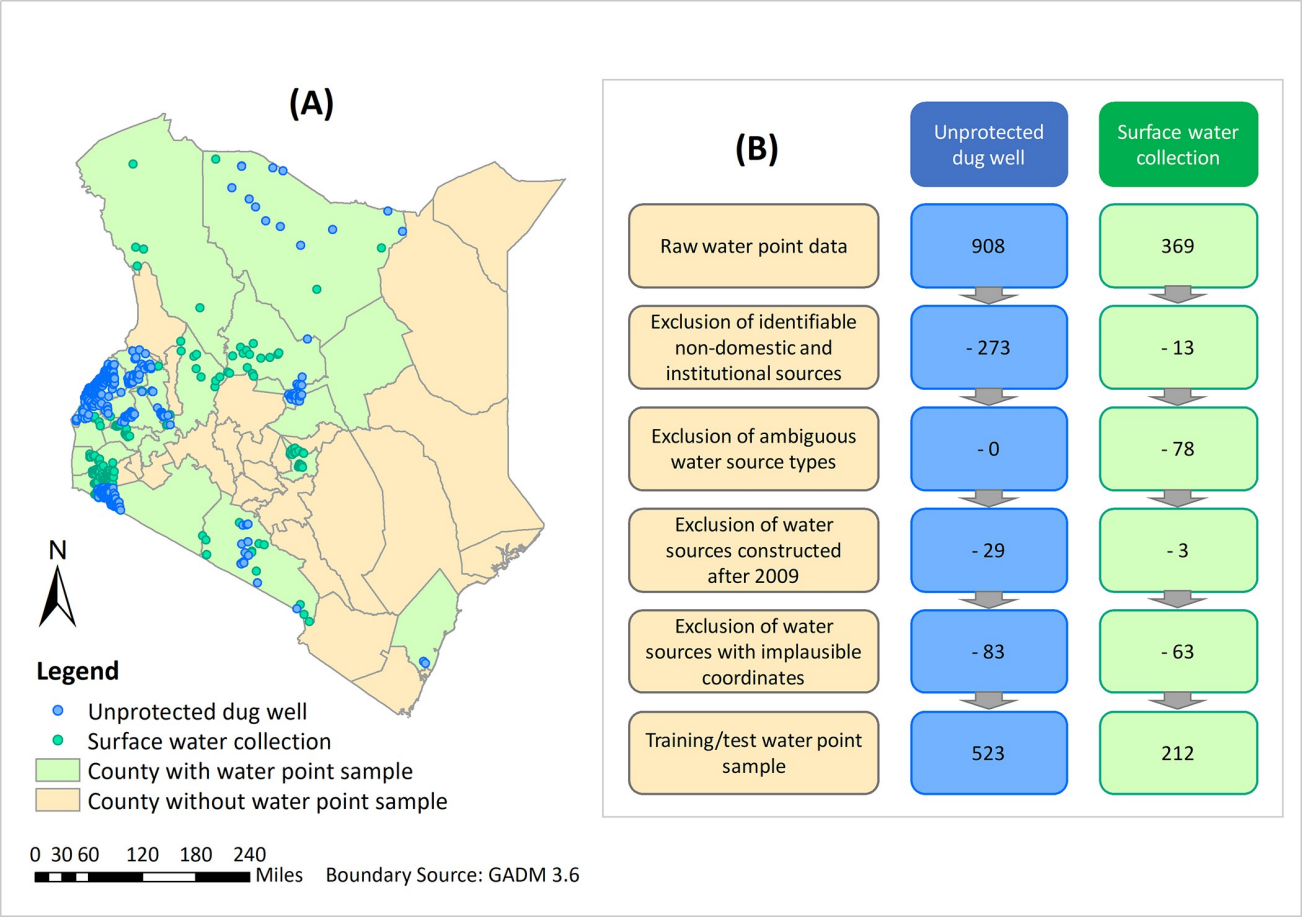
Unprotected dug wells and surface water points included for the MaxEnt modelling in this study. (A) Map showing the distribution of included unprotected dug wells and surface water points; (B) numbers of water points excluded during data pre-processing based on the exclusion criteria.

For illustration purposes, we focused specifically on two types of unimproved water sources, namely points representing unprotected dug wells (n_u_ = 523) and surface water (i.e. where households directly draw untreated water from rivers, streams, ponds, and lakes, n_s_ = 212) at a spatial resolution of 1 km (see Fig 1). This was selected considering both the availability and quality of relevant predictive covariates as well as water point data. We acquired water point data from the WPDx (http://www.waterpointdata.org/) database on 10^th^ April 2018. This included user-uploaded water point inventories from diverse data sources, and therefore increases the variety of sample data. We restricted our analysis to household domestic sources, excluding institutional sources and water points potentially used for non-domestic purposes such as irrigation and watering livestock. For surface water, we further excluded water points where an ambiguous water source type (e.g. spring, dam, pan etc.) or a water lifting or extraction mechanism was recorded, as this may indicate a higher service level than direct consumption of surface water, as defined in the water ladder used in international monitoring [68]. To increase temporal consistency with covariate map layers, water points reportedly installed after 2009 were excluded in our analysis, assuming that sources lacking installation dates were installed before 2009. There was insufficient information recorded to exclude non-functional sources, so water sources with reported functionality and service continuity issues were retained for analysis. Water points located outside the study area or within large inland water bodies were also excluded.

### 2.3 Predictive covariates

We identified predictive covariates that may affect the distribution of unprotected dug wells and surface water based upon our conceptual framework (**Table 1**): surface water or groundwater availability, feasibility of shallow well construction, and compatibility of water source type with local socio-economic conditions. For all predictive covariates, we selected data sources from as close to the year 2009 as possible. When a covariate layer lacked temporal meta-data, we assumed that the state of that covariate did not change significantly over time.

**Table 1:** Conceptual framework of potential factors influencing the distribution of unprotected dug wells and surface water.

**Type of factor**	**Perspective**	**Unprotected dug well**	Surface water
Environmental factors	Original source of water	Potential presence of available groundwater according to hydrogeological and geological conditions (e.g. groundwater productivity; depth to groundwater; etc.)	Presence of available surface water (e.g. rivers, streams, lakes, ponds, etc.)
Technological factors	Technological preferences and avoidance	Feasibility of manual digging; ease of access for construction; avoidance of contamination hazards	N/A
Socio-economic factors	Demand for water	Presence of people without alternative water sources, or with alternative water sources that are not reliable (e.g. of poor quality, poor accessibility, with supply interruptions, etc.)	Presence of people without alternative water sources, or with alternative water sources that are not reliable (e.g. of poor quality, poor accessibility, with supply interruptions, etc.)
Socio-economic factors	Consumer accessibility	Ease of access for water collection (e.g. shorter walking-distance or water fetching times; no restriction due to land ownership; etc.)	Ease of access for water collection (e.g. less walking-distance; less time to spend; no restriction due to land ownership; etc.)
Socio-economic factors	Local preferences	Affordable water source; good perceived water quality; avoidance of places of perceived local importance	Affordable water source; good perceived water quality; potential avoidance of interference with places of high local importance

For environmental and technological factors, we selected covariates characterising the hydrogeological and geological environment which relate to groundwater and surface water availability and in turn affect feasibility of shallow well construction [48–52,54,55,69–72], including depth to groundwater, groundwater productivity, groundwater storage, drainage density, elevation, slope, topographic wetness index (TWI), proximity to inland water, land use, lithology, and soil texture. For unprotected dug wells, we created groundwater productivity and storage covariate layers using the Surficial Geology of Africa data developed by U.S. Geological Survey (USGS), Central Energy Resources Team as geological base map (nominally at 1:5,000,000), subsequently rasterising this layer at 1km spatial resolution. Detailed aquifer types, groundwater productivity and storage maps were defined for this layer with reference to 5 km resolution quantitative digital groundwater maps of Africa [73]. Polygons smaller than the 5 km grid resolution were characterised by visual comparison with the hydrogeological information published on the British Geological Survey (BGS) channel (http://earthwise.bgs.ac.uk).

For socio-economic factors, the main covariates employed in this study include Euclidean distance to 1km grid cells containing buildings, town centres, villages and roads in a consideration of accessibility and proximity to human settlements. For surface water, we also created a gridded layer of cost distance (i.e. walking time) to inland water based on slope and land use to reflect ease of access for water collection, as this is an important criteria for households when selecting water sources [74,75]. In addition, since healthcare facilities are often considered a strong predictor of population presence [76], we assumed that they may in turn correlate with constructed water sources and therefore included Euclidean distance to healthcare facilities in modelling unprotected dug wells. Furthermore, we searched for map layers reflecting planning restrictions on well development [77], using Euclidean distance to areas protected for conservation as one such covariate. Furthermore, given the many accounts of disparities in unimproved source use between urban and rural areas and between rich and poor [67,74,75], we also included poverty defined by a Multidimensional Poverty Index (MPI) [78] and urban/rural settlement areas in both models.

All covariate layers were prepared at a spatial resolution of 30 arc-seconds (approximately 1km at the equator) showing terrestrial areas only, excluding large water bodies and all covariate map layers used the same extent and resolution as this layer. In addition, we calculated the Pearson’s correlation [79] between continuous covariates; Polychoric correlation [80] between ordinal categorical covariates; and Polyserial correlation [80] between continuous and ordinal categorical covariates for each model. This analysis was to identify and remove strongly correlated covariate pairs (correlation coefficient > 0.7 or < -0.7) to reduce collinearity, as recommended in a previous study [57], retaining the variable in each pair most obviously related to water point distribution. All data pre-processing was carried out using ArcGIS 10; whilst all correlation analyses were carried out using R 3.4.0, where Polychoric and Polyserial correlations were computed with the polycor package [81]. Details of the predictive covariates used and corresponding data sources can be found in S1 File.

### 2.4 Model implementation

**Fig 2:**
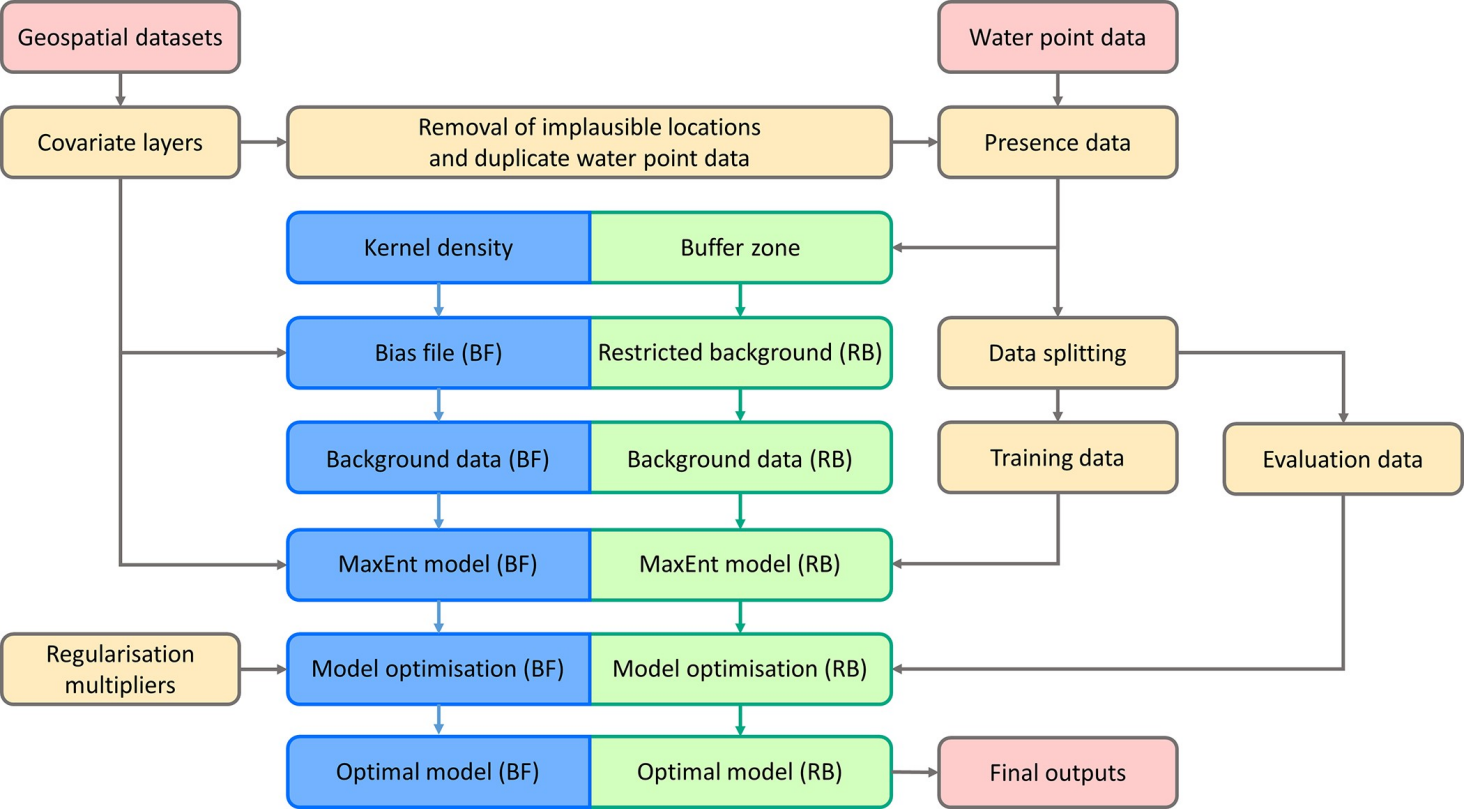
Flow chart of the MaxEnt modelling methodology.

Fig 2 depicts the processing flow for the MaxEnt modelling adopted in this study. Each model was built using a random sample of 70% of the included water points. The remaining 30% of water point presences were used to test model performance based on Monte Carlo cross-validation [82]. We applied two different strategies to control for spatial variation in water point mapping effort and resultant sampling bias, namely use of a bias file and a restricted background correction [83]. For the first method, we generated 10,000 background points by randomly selecting points within the entire study area, weighted by a bias file. A kernel density surface derived from locations of all obtained water points was used as the bias file, assuming that survey effect was concentrated around these known water points. For the second method, we restricted the selection of 10,000 background points to 100 km buffer areas around water point locations. For both unprotected dug wells and surface water, we repeated the sampling of training and test points, model fitting, model evaluation and prediction 50 times for each bias correction strategy, and then computed aggregated predictions and model performance metrics from all 50 model runs.

Model fitting and analysis were carried out using Maxent v3.3.3k [22]. We kept all non-correlated covariates identified by our framework in this illustrative study, since one of our aims is to examine the potential environmental and socio-economic drivers of domestic water access distribution. In addition, we selected all available functional transformations of the predictive covariates (i.e. ‘linear’, ‘quadratic’, ‘product’, ‘threshold’, ‘hinge’, and ‘discrete’) [57] to capture the potential non-linear relationships between the covariates and target water points, to allow for complex relationships between socio-ecological factors and locations of water points. To optimise MaxEnt model complexity, we selected the best model corresponding to the regularisation multiplier with the largest AUC instead of using information criteria (AIC, AICc, BIC), due to the known issues with threshold and hinge features [84]. For the purposes of simple illustration and reduction of computational cost, we restricted our test of regularisation multipliers to integers from 1 to 10 based on the results of 50 replicated runs of each model, and selected the best model accordingly. We kept all other settings at their default value, and used logistic output for easier interpretation. However, as this logistic output arbitrarily assumes an occurrence prevalence of 0.5 [57], we interpreted this as the relative ranking of each cell across the landscape rather than true occurrence probabilities. The changes in regularised gains (percent contribution), changes in training AUC based on permuted data (permutation importance), and Jackknife test were used to evaluate the contribution of the covariates to the MaxEnt predictions.

### 2.5 Performance evaluation

Evaluation of model performance was carried out using Area Under the Receiver Operator Curve (AUC) [85] from the 30% testing sample for the 50 replicated runs. The AUC value is in the range of 0.5 to 1.0, where 1.0 reflects perfect discriminatory power, whilst 0.5 indicates that the prediction failed to capture any patterns and was no better than a random distribution. As the AUC value approaches 1.0, it indicates potentially useful discrimination by the model [58,86].

As an additional means of model evaluation, we calculated the density (persons per hectare) of population using unprotected dug wells or surface water as their main drinking-water source at the most disaggregated levels available via open data sources. For surface water, county level (post-2013 administrative level 1) data from the 2009 Kenya Population and Housing Census was acquired from the Kenya Open Data portal (http://opendata.go.ke/). For unprotected dug wells, publicly available census data did not distinguish water wells from boreholes and springs, so we acquired 2008–09 DHS data at regional level (pre-2013 administrative level 1) [87]. We used Spearman’s Rho to examine the correlation across sub-national areas between water consumer density and (a) the density of ‘raw’ input water points; (b) the model output, namely the average predicted relative occupancy/suitability in populated areas. The populated areas were defined based on the Global Human Settlement (GHS) grid [88].

### 2.6 Ethics statement

This study involved use of aggregated nationally representative household survey data and population and housing census data from openly accessible data sources; it did not entail any work with records concerning individual human subjects nor collect any data. The research proposal (ID 18551) was submitted to the University of Southampton’s Ethics and Research Governance Online (ERGO) system on 10^th^ December 2015 and was reviewed and approved by the ethics committee on 15^th^ January 2016.

## 3. Results

### 3.1 Model output and performance evaluation

**Fig 3:**
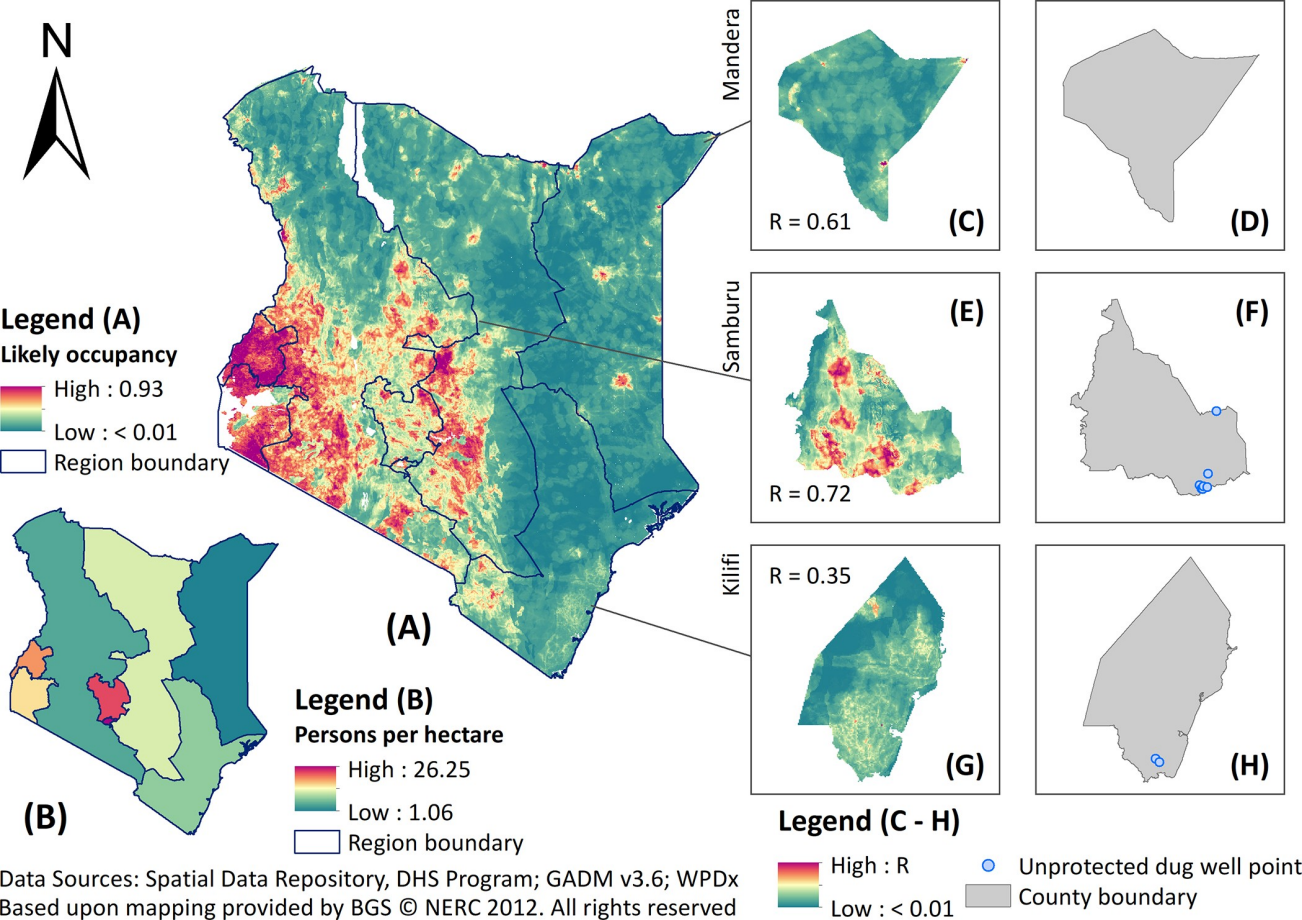
Predicted unprotected dug well occupancy across Kenya with inset maps highlighting Mandera, Samburu, and Kilifi counties. (A) Unprotected well occupancy predicted by MaxEnt at 1 km resolution; (B) DHS-based consumer density map of unprotected dug wells at the regional level; inset maps show MaxEnt prediction versus the initial water point coverage in Mandera (C, D), Samburu (E, F), and Kilifi (G, H) counties.

**Fig 4:**
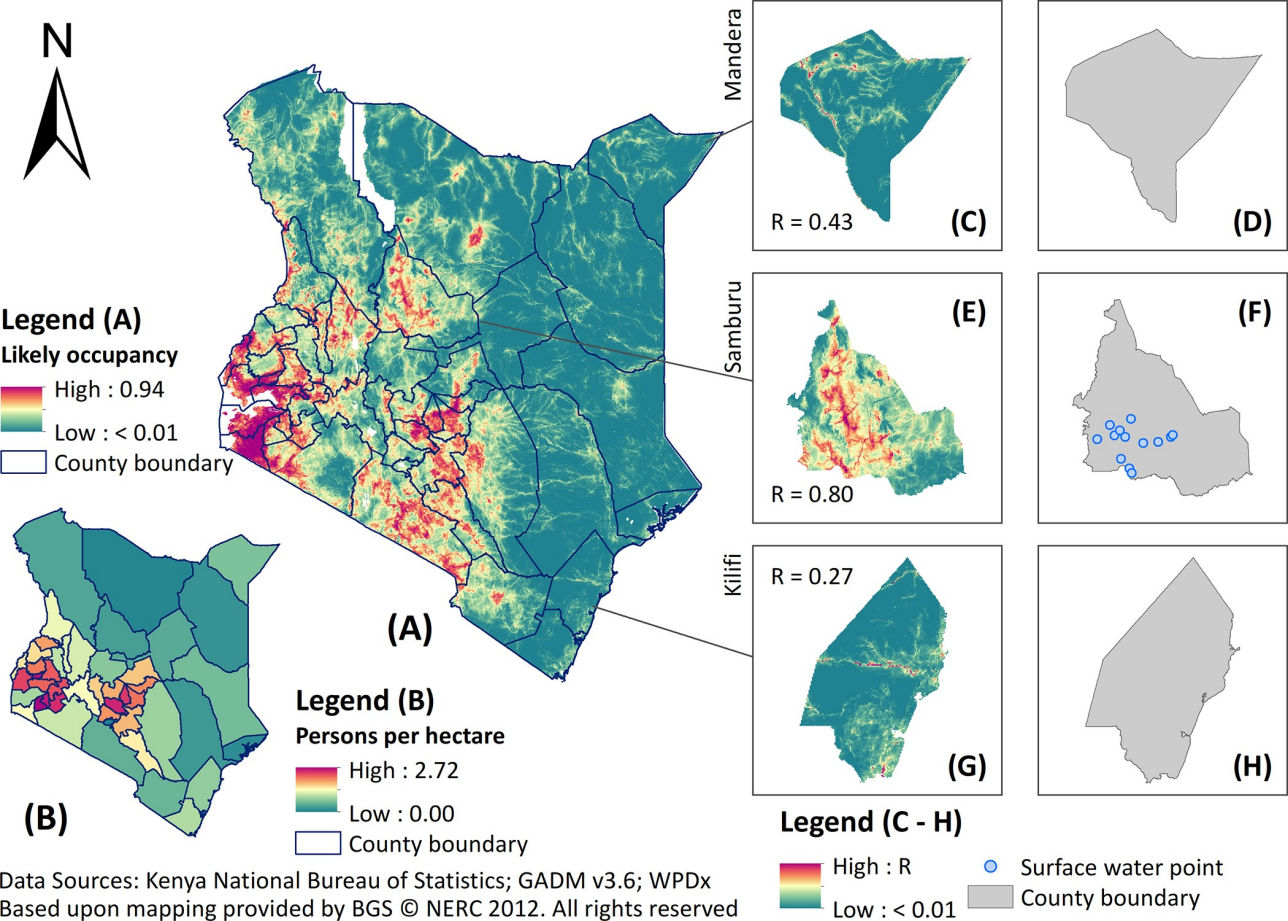
Predicted surface water occupancy across Kenya with inset maps highlighting Mandera, Samburu, and Kilifi counties. (A) Surface water occupancy predicted by MaxEnt at 1 km resolution; (B) census-based consumer density map of surface water at the county level; inset maps show MaxEnt prediction versus the initial water point coverage in Mandera (C, D), Samburu (E, F), and Kilifi (G, H) counties.

In this illustrative study, 1 is found to be the optimal regularisation multiplier among the tested integers for both types of water point and bias correction methods. Our models display high predictive power according to the AUC values (all above 0.9), which suggests that the predictions successfully captured relationships between water points and relevant covariates. For unprotected dug wells (Fig 3A), a substantial area of western and central Kenya was predicted to have higher unprotected dug well occupancy, with isolated spots of relatively high occupancy in towns and urban centres in the north and northeast parts of the country, as well as the coastal regions in the south. For surface water (Fig 4A), the areas predicted to have high likely occupancy were also concentrated in western and central Kenya, but sparsely distributed in eastern Kenya. In arid regions lacking mapped water points, such as the insecurity-affected counties bordering Somalia, both models showed limited variation in predicted relative occupancy values relative to western and central parts of Kenya, which had more input water points. The final predictions for unprotected dug wells show patterns consistent with water consumer density (persons per hectare) derived from 2008–09 DHS (Fig 3B; r_s_ = 0.714), whilst those for surface water were consistent with 2009 census data (Fig 4B; r_s_ = 0.722). These correlations with water consumers reported in demographic data were greater than those calculated using the ‘raw’ input water points (r_s_ = -0.073 for unprotected dug wells and r_s_ = 0.188 for surface water). This suggests some success in gap-filling the initial, incomplete spatial coverage of water point mapping, as detailed in the inset maps in Figs 3 and 4 for example. S1 File provides further details about each prediction and associated uncertainty maps by source type and bias correction method.

### 3.2 Covariate contribution analysis

**Fig 5:**
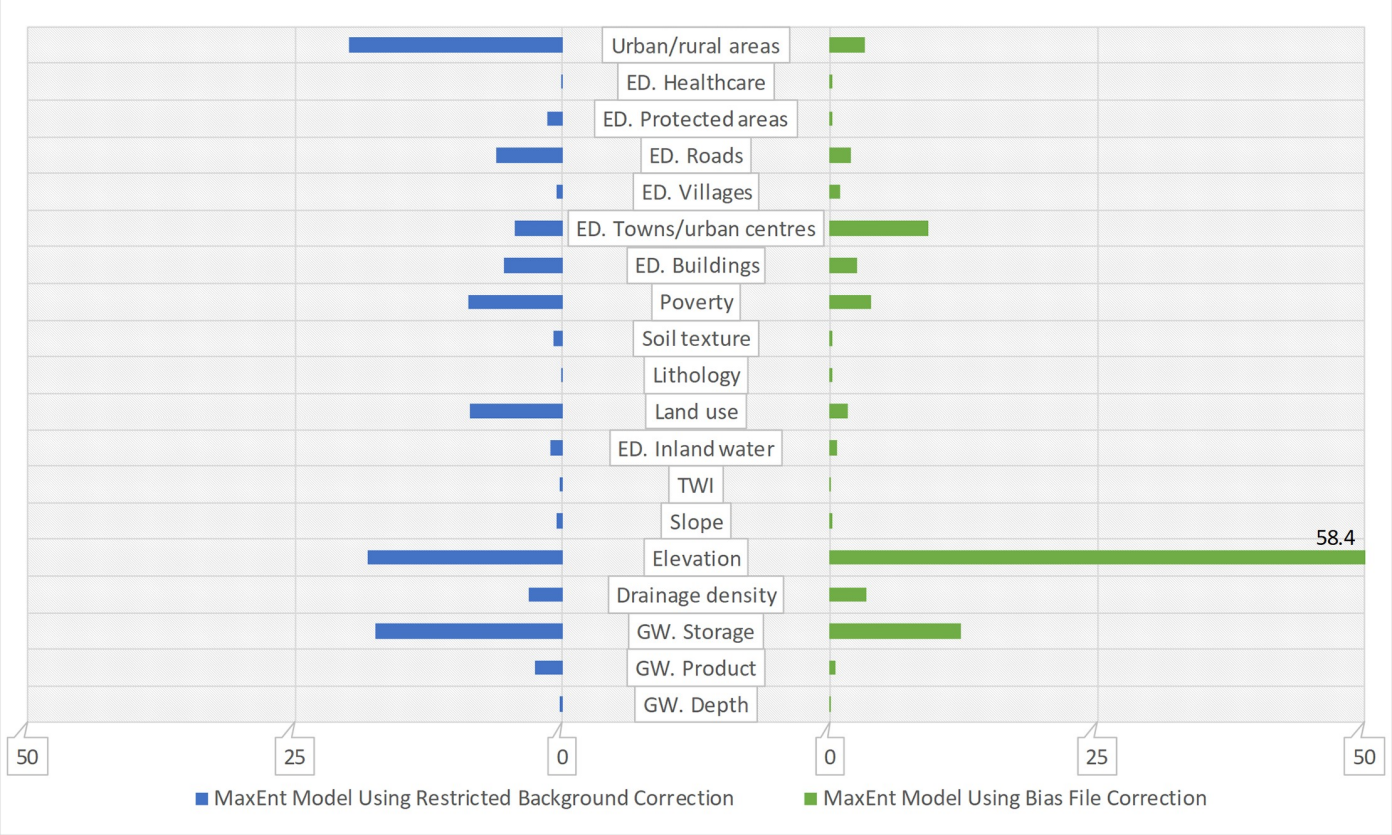
Covariate contribution to the MaxEnt model of unprotected dug wells for two bias correction methods. ED. denotes Euclidean distance; GW. denotes groundwater; the x-axis is the percent contribution of the predictive covariate based on changes in regularised gain.

**Fig 6:**
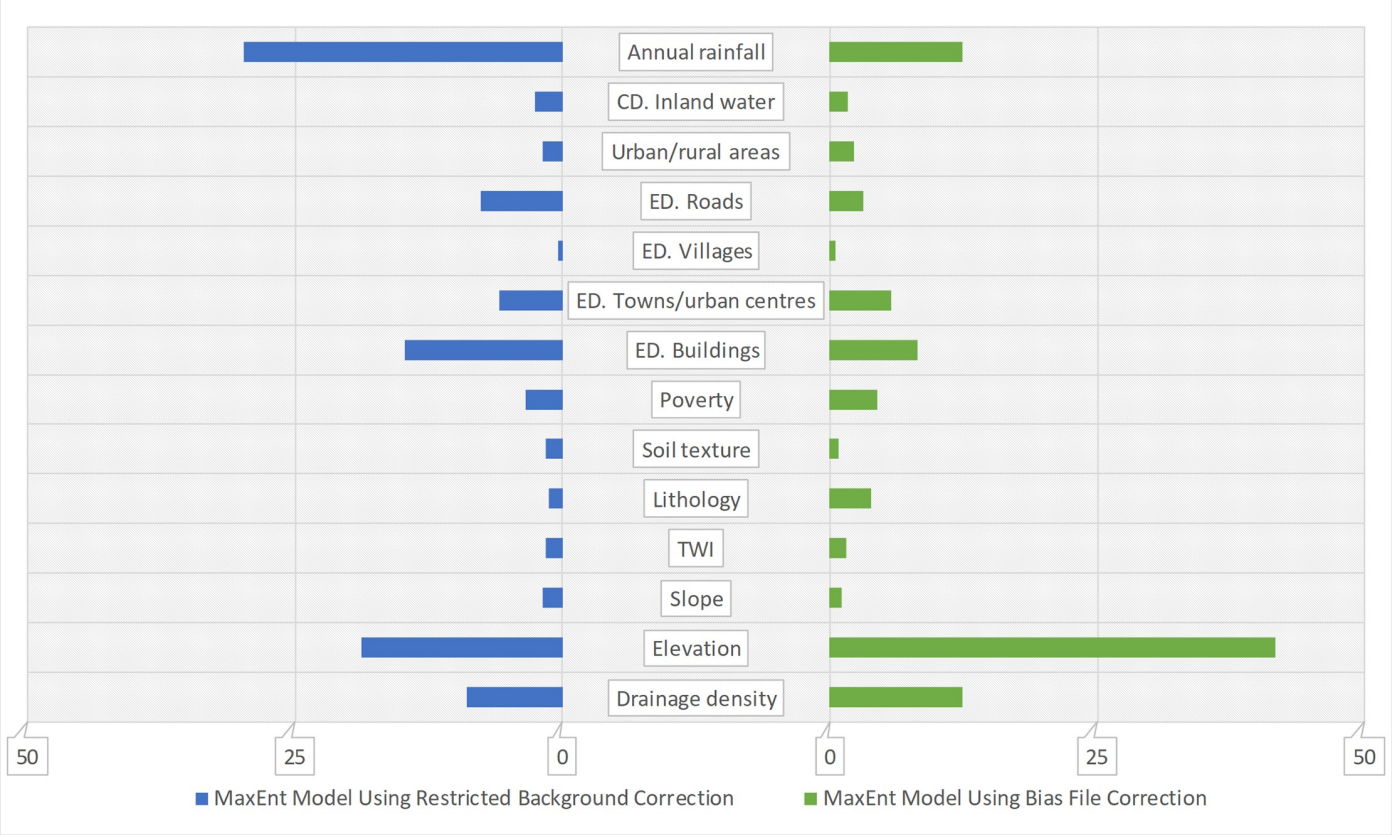
Covariate contribution to the MaxEnt model of surface water for two bias correction methods. CD. denotes cost distance. ED. denotes Euclidean distance; the x-axis is the percent contribution of the predictive covariate based on changes in regularised gain.

The relative contribution of the covariates varied, depending on bias correction method. For the unprotected dug wells model based on the restricted background correction, percent contribution analysis indicated that urban-rural divide and groundwater storage provided the most useful information, with elevation closely following (Fig 5, data in blue bars). However, elevation had the greatest contribution to the unprotected dug wells model when the bias file correction was applied, whilst urban-rural divide only had a moderate contribution. Groundwater storage and Euclidean distance to towns and urban centres were respectively the second and third most important covariates in the model (Fig 5, data in green bars). For surface water (Fig 6), percent contribution analysis indicated annual rainfall was the most influential covariate in the model based on restricted background correction, whilst elevation had the greatest contribution to the model with bias file correction.

Different covariate contribution analysis methods also yielded different results (see S1 Table). In general, however, for unprotected dug wells, elevation, poverty, and urban-rural divide were found to have the most useful information, whilst other covariates such as groundwater storage, drainage density, Euclidean distance to buildings and Euclidean distance to towns and urban centres were also very important to the models. For surface water, elevation, annual precipitation and drainage density were found to be the most important covariates, whilst Euclidean distance to buildings and cost distance to inland water also appeared relatively important. A full list of the response curves of predictive covariates can be found in S1 Fig.

## 4. Discussion

For the first time, our study has applied a spatial ecology technique, namely maximum entropy modelling, to map point locations of access to domestic water supplies. The output gridded map layer of relative likely occupancy adjusts the raw input water point mapping data to account for incomplete coverage, which forms the principle advantage of the technique. Incompleteness is a known issue with some water point mapping data sets: for example, Yu et al. [9] found that Tanzanian and Cambodian databases contained insufficient water points to account for the number of households reporting use of such water sources in census data, which therefore undermined their utility in national level monitoring of water access. The frequency of missing values in water point attribute fields (including water source type) is often high [89], further exacerbating incompleteness when enumerating water points of a particular type. By examining the relationship between covariates and the presence of water points, this spatial predictive modelling technique adjusts for such coverage gaps.

Spatial predictive modelling techniques have been widely employed in different fields for depicting spatial distributions with incomplete locational observations. Although previous studies suggest algorithms based on bioclimatic envelopes [10,11], Gower distance [12] and Mahalanobis distance [13,14] may be unsuitable for categorical covariates, have poor predictive performance, and thus be unsuitable for this application, this illustrative assessment with a MaxEnt model indicates the potential for using other novel spatial predictive models in isolation or in ensemble to map access to domestic water supplies. Some novel methods such as random forests require presence-absence data, but these can still be used with water point data by treating cells unoccupied by water points as pseudo-absence data. Regardless of which method is used, however, the outputs need to be interpreted with caution because no absence data are available.

In this study, MaxEnt’s logistic output was used instead of the raw output for easier interpretation, since the logistic output values typically correspond better with predicted relative site occupancy/suitability in comparison with the raw output. However, due to the arbitrarily assumed occurrence prevalence (0.5) in the calculation of the logistic output [57,90], the predicted likely occupancy values only indicate ranked relative site suitability, as they are theoretically the order-preserved transformations of the probability of water point occurrence. In addition, due to the use of background data rather than true absence data, a predicted likely occupancy value should not be considered as the true probability of occurrence.

The gridded output surfaces could be used in several ways. In the same way that ecologists have successfully used such model outputs to target follow-up surveys to find unmapped species occurrences [91], so the output map layers could potentially be used to target follow-up surveys of areas likely to contain unmapped water points. The output layer could also be compared to similar, gridded layers depicting household use of different water source types [8], which have been generated in several countries by applying geostatistical methods to household survey data. The WHO/UNICEF core question on drinking-water used in household surveys asks ‘what is the main source of drinking-water for members of your household?’ [92] and therefore captures only the main water source for drinking purpose. In contrast, water point mapping captures secondary, seasonal, and domestic water sources for purposes other than drinking. Gridded outputs derived from water points versus household surveys would thus potential capture different dimensions of domestic water resource use. Furthermore, because of the need to protect data concerning human subject, household survey GPS locations are randomly displaced [93], which restricts the resolution of derived gridded representations of household source use to 5km by 5km grid cells. In contrast, this data protection issue does not affect the spatial precision of water points, since they do not relate to human subjects. The MaxEnt output could also be combined with map layers depicting aquifer vulnerability [94] to contamination or geogenic contaminants [95], so as to identify areas where water users are exposed to such hazards, since relevant map layers often come in the gridded format.

Our case study predicted high relative occupancy of unprotected wells in Kenya’s densely populated urban areas. This is consistent both with the high population densities and high unprotected well use reported in the Langas slum in Eldoret [96] and in Kisumu’s slums [97]. Since many rivers and streams in semi-arid and arid Kenyan counties are seasonal rather than perennial, predicted direct domestic consumption of surface waters in these counties may also be seasonal.

A secondary potential benefit of the technique is the understanding gained of the association between covariate map layers and the spatial distribution of water points. Given ever-increasing demand on water resources [98], such insights into landscape-level characteristics associated with water resource exploitation could prove valuable. In this Kenyan case study, a high percentage contribution to surface water and unprotected well occurrence was attributed to elevation. A similarly strong correlation with elevation has been found for population density [99,100], so this apparent association between elevation and water points may reflect human settlement patterns. Globally, according to census data, unimproved source use, particularly direct consumption of surface waters, is known to be higher among rural populations and the poor [64]. This is reflected in the high covariate contributions for map layers depicting poverty in both sets of models and a high covariate contribution for rurality in the models based on restricted background correction. Unsurprisingly, rainfall and surface drainage density also had great covariate contributions to the surface water model.

In general, the response curves are consistent with our understanding of the hydrogeological and socio-economic determinants of unimproved water access distribution. However, some model covariates exhibit complex, random or somewhat implausible response curves. Some covariates included in the model for unprotected dug well and surface water collection (see S1 Fig) exhibit such response curves, likely due to potential sampling biases. However, such covariates had only small contributions to the final model, reducing concern over their impact on predictions. Given that we wished to illustrate application of a simplified model to water point mapping here, we did not attempt to reduce model complexity to resolve such issues, though further optimisation would be possible. The spatial ecology literature identifies several known limitations of MaxEnt modelling. The output is highly dependent on the spatial distribution of the input occurrences and choice of covariates. In our case study, the human settlements layer that we used as a covariate does not differentiate slums from other urban areas, which affects predicted relative occurrence of unprotected wells in such areas. Our Kenyan case study used very coarse spatial resolution hydrogeological data, which may have reduced the apparent association between unprotected well occurrences and hydrogeology. In addition, some covariate layers (e.g. annual rainfall, inland water, etc.) dated from different periods to the water point survey dates. Such differences in temporal coverage could have reduced the strength of association between these layers and water point occurrences. Similarly, in our case study implementation, the input water points are heavily concentrated in only a few Kenyan counties. A particular concern where the input occurrence data are concentrated in a small area is out-of-sample prediction whereby the modelled output makes predictions for areas that experience combinations of environmental and socio-economic conditions not found in the training data [63]. In our case study implementation, for example, the training data include very few water points with elevations below 500 metres. More generally, the quality of the input water point data will affect the output gridded probability layer. An example of one such issue would be the misclassification of water source types arising from difficulty in differentiating protected versus unprotected wells or boreholes versus protected wells.

Aside from these issues affecting model calibration, there are further drawbacks of this approach to analysing water points. One difficulty is in evaluating the output relative occurrence grids, which we attempted via census and survey data in the Kenyan case study implementation. However, the coarse spatial resolution of household survey data in particular somewhat limits the usefulness of this evaluation. Apart from the WPDx there are other water point databases for Kenya [101,102], but differences between databases in the water source typologies used inhibit their usefulness for model validation. Thus, unprotected wells can be unambiguously identified in WPDx records, but not in the other data set. Points where surface water is used for domestic purposes could be corroborated via remotely sensed images of such surface water bodies, though not all surface water bodies will be used by households. In future, evaluation of model outputs by an expert panel may therefore be more effective than data-driven model validation. As with all data products generated through complex analysis protocols, a further difficulty is in communicating the nature of the product and the underlying data processing to a wider audience.

Although our case study implementation of the methodology in Kenya may be subject to these limitations, there are freely available, easy-to-use interfaces to both MaxEnt and other related environmental niche modelling techniques [103]. It would therefore be possible for other research groups with access to more appropriate covariate map layers or more representative, higher quality input water point data sets to refine the methodology and address any limitations in input data or covariate choices in our case study implementation. However, appropriate model development and parameterisation require a strong understanding of the technique’s underlying principles [57]. Subject to sufficient expertise in the software’s use, the approach could thus be customised to local conditions and data availability and, given the existence of a global water point data exchange platform [104], is potentially also scaleable. In our case study, we used a single ‘presence-background’ technique (MaxEnt) for illustration, but many ecologists use an ensemble approach, whereby multiple environmental niche modelling techniques are used in combination [105,106]. An ensemble approach to water point analysis would thus be a logical future methodological refinement. Although we have applied the method to just two types of water point here, in principle it could be applied to other types such as boreholes, kiosks, or rainwater harvesting systems. Mobile forms of water provision, such as water sold from tanker truckers or vendors using carts, are however inherently difficult to capture via water point mapping. In ecology, environmental niche modelling has proved more successful for endemic or specialist species occupying narrow environmental niches, rather than more generalist species found in many environments [106,107]. This implies that the technique may perform better for water source types found only in a narrow range of socio-economic and environmental conditions, as opposed to source types that are installed under wide-ranging conditions.

## 5. Conclusion

This illustrative study takes a technique widely used in spatial ecology to analyse incomplete occurrence data and applies it to two types of water point in Kenya. The technique has potential to correct water point databases for incomplete survey coverage and provide insights into environmental and socio-economic characteristics associated with water points as landscape features. However, the spatial ecology literature also highlights some important limitations of the approach. These include the potential pitfalls of making predictions for environmental conditions not represented in training data and poorer model performance when predicting occurrences of generalist species (or here water points) found in a wide range of environments. Although we only applied the maximum entropy model in Kenya, the methodology could potentially be adapted to other predictive modelling algorithms, settings, types of water points and local data availability. It is also potentially scaleable given the existence of a global water point mapping data exchange, but its further uptake requires expert knowledge and strong understanding of the underlying principles of ecological niche understanding.

## Supporting information

S1 File A document containing additional information on covariate layers, data sources, and output prediction surfaces by water source type and bias correction method. (PDF)

S1 Table A Microsoft Excel file containing tables that describe covariate contributions by water source type and bias correction method. (XLSX)

S1 Fig A document containing graphs of response curves by predictive covariate, by water source type and bias correction method. (PDF)
