# Supplementary material for: Mapping access to domestic water supplies from incomplete data in developing countries: An illustrative assessment for Kenya
Source: PLoS One. 2019 May 17;14(5):e0216923. doi: 10.1371/journal.pone.0216923 (PMC6524943; doi:10.1371/journal.pone.0216923)
Supplement: S1 File — (PDF) [file pone.0216923.s001.pdf]

S1 File. Additional information

1. Additional details on covariate layers and data sources

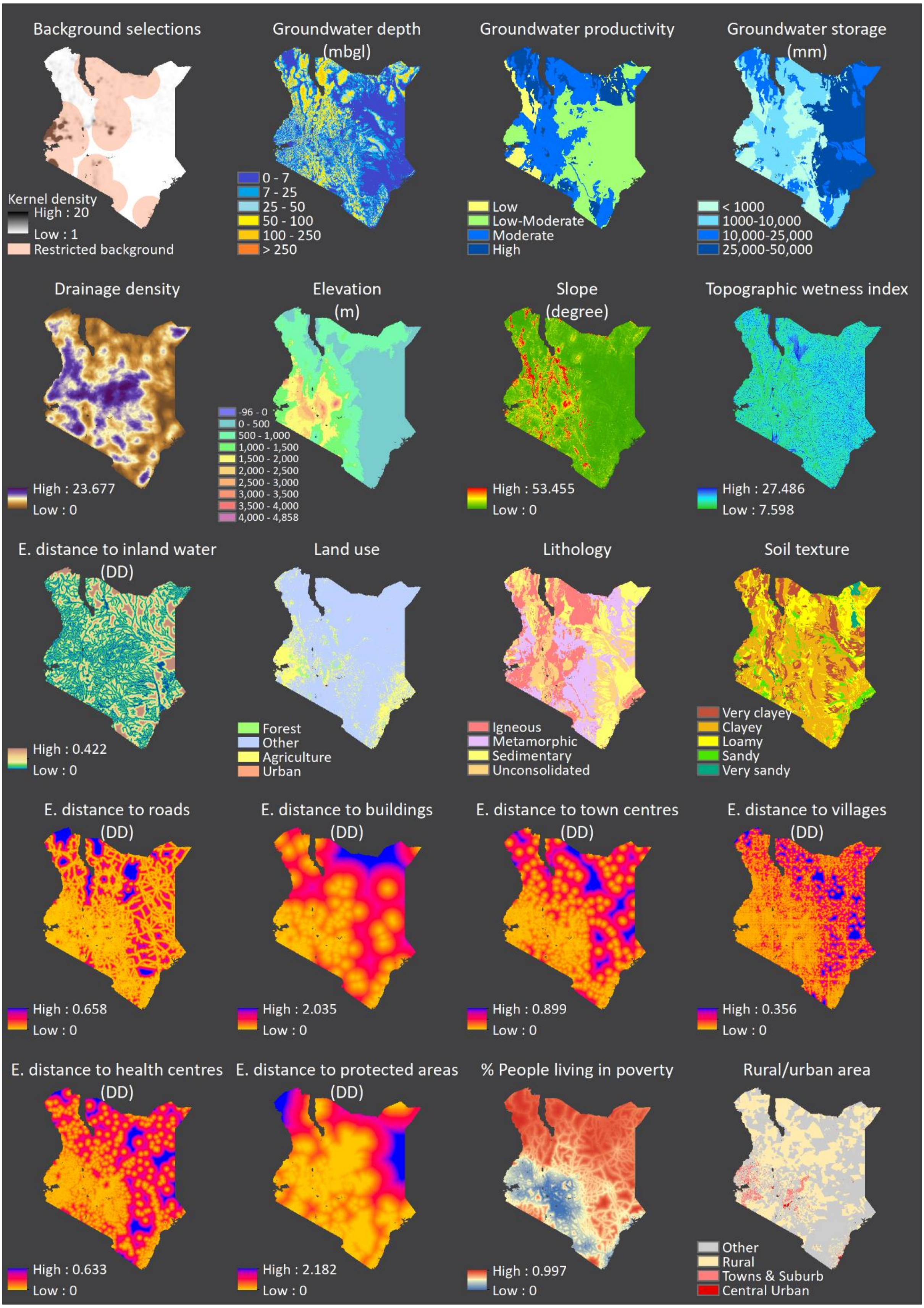

Figure S1_File.1. Selected predictive covariates for the MaxEnt model of unprotected dug wells

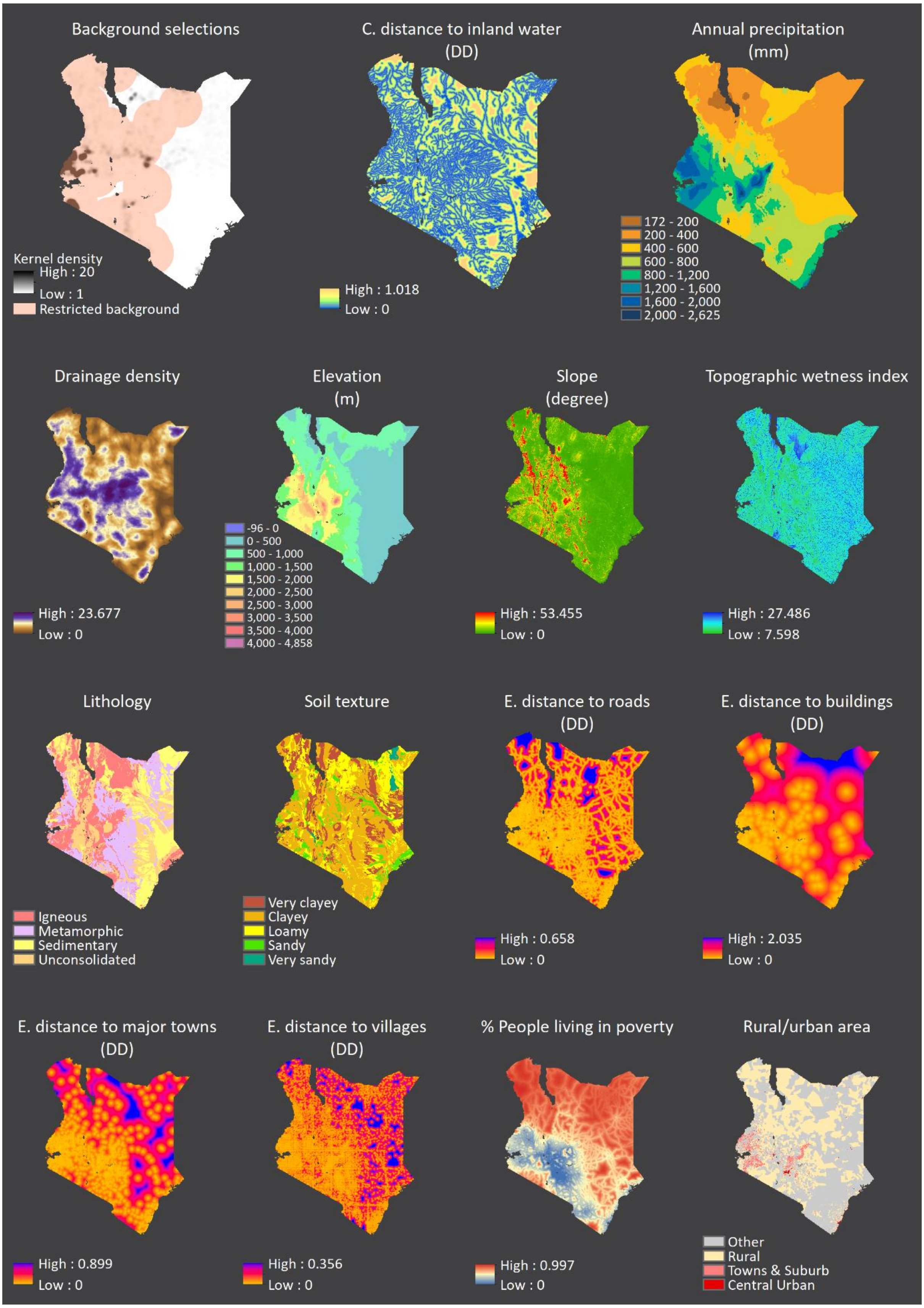

Figure S1_File.2. Selected predictive covariates for the MaxEnt model of surface water sources

Table S1_File.1. Information about the predictive covariates used for estimating the potential spatial distribution of unprotected dug wells and surface water

Covariate name	Type	Model	Data type	Data source
Depth to groundwater table	Continuous	U	Depth to groundwater table, 1km NetCDF raster	Equilibrium Water Table Africa Model version 2 [1]
Groundwater productivity	Ordinal	U	Groundwater productivity map, 5km ASCII XYZ raster	Digital groundwater maps of Africa [2]
			Geology, 1:5,000,000 scale polygon vector	Surficial Geology of Africa version 2.0 [3]
Groundwater storage	Ordinal	U	Groundwater storage map, 5km ASCII XYZ raster	Digital groundwater maps of Africa [2]
			Geology, 1:5,000,000 scale polygon vector	Surficial Geology of Africa version 2.0 [3]
Drainage density	Continuous	U, S	Inland waterways, 1:1,000,000 scale polyline vector	Digital Chart of the World (DCW)
Elevation	Continuous	U, S	Digital elevation model, 30m Geotiff raster	ASTER GDEM Version 2 [4]
Slope	Continuous	U, S	Digital elevation model, 30m Geotiff raster	ASTER GDEM Version 2 [4]
Topographic wetness index (TWI)	Continuous	U, S	Digital elevation model, 30m Geotiff raster	ASTER GDEM Version 2 [4]
Euclidean distance to inland water	Continuous	U	Inland water, 1:1,000,000 scale polyline/polygon vector	Digital Chart of the World (DCW)
Land cover/land use	Categorical	U	Land cover, 500m Geotiff raster	MODIS Land Cover Type (MCD12Q1) Version 5.1 [5]
Lithology	Categorical	U, S	Lithology, 1:1,000,000 scale polygon vector	Kenya Soil Survey (KSS)
Soil texture	Categorical	U, S	Properties of Kenya soils, 1:1,000,000 scale polygon vector	Kenya Soil Survey (KSS)
% resident living in MPI-defined [6] poverty	Continuous	U, S	% residents living in MPI-defined* poverty, 1km Geotiff raster	Worldpop [7]
Euclidean distance to residential buildings	Continuous	U, S	Point locations of residential buildings, point vector	OpenStreetMap [8]
Euclidean distance to towns and urban centres	Continuous	U, S	Point locations of towns and urban centres, 1:250,000 (Northern Kenya) and 1:50,000 (rest areas) scale point vector	International Livestock Research Institute (ILRI)
Euclidean distance to villages	Continuous	U, S	Point locations of villages, point vector	Almanac Characterisation Tool (ACT) database
Euclidean distance to roads	Continuous	U, S	Roads, polyline vector	OpenStreetMap [8]
Euclidean distance to protected areas	Continuous	U	Protected areas, polygon vector	The World Database on Protected Areas (WDPA)
Euclidean distance to healthcare facilities	Continuous	U	Point locations of healthcare facilities, point vector	Kenya Medical Research Institute (KEMRI); TALA Research Group; Department of Zoology, University of Oxford; Ministry of Health and Center for Tropical Medicine
Rural-urban areas	Categorical	U, S	Classified human settlements model, 1km Geotiff raster	Global Human Settlement (GHS) Settlement Grid [9]

Cost distance to inland water	Continuous	S	Inland water, 1:1,000,000 scale polyline/polygon vector	Digital Chart of the World (DCW)
			Digital elevation model, 30m Geotiff raster	ASTER GDEM Version 2 [4]
			Land cover, 500m Geotiff raster	MODIS Land Cover Type (MCD12Q1) Version 5.1 [5]
Annual precipitation	Continuous	S	Annual precipitation, 1km Geotiff raster	WorldClim Global Climate Data version 1.4 [10]

Abbreviations in the table: 'U' means the covariate is used for creating the MaxEnt model of unprotected dug wells, and 'S' means it is used for surface water model.

Table S1_File.2. Download links of the raw data sets used in this study

Data set	Download link
Water Point Data Exchange (WPDx) water point data	WPDx https://www.waterpointdata.org/ (Accessed: 2018-04-10)
Equilibrium Water Table Africa Model v2 [1] depth to groundwater table NetCDF raster	Global Water Scarcity Information Service (GLOWASIS): https://glowasis.deltares.nl/thredds/catalog/opendap/opendap/Equilibrium_Water_Table/catalog.html (Accessed: 2017-03-29)
Digital groundwater maps of Africa [2] groundwater maps	British Geological Survey (BGS): https://www.bgs.ac.uk/research/groundwater/international/africanGroundwater/mapsDownload.html (Accessed: 2017-05-08)
Surficial Geology of Africa version 2.0 [3] geology map	USGS Energy Data Finder: https://certmapper.cr.usgs.gov/geoportal/catalog/search/resource/details.page?uuid=%7BC7E34C48-9BD9-45C6-AD08-E549CFB3715A%7D (Accessed: 2016-06-08)
Digital Chart of the World (DCW) ESRI Shapefiles	DIVA-GIS: https://www.diva-gis.org/gdata (Accessed: 2017-04-02)
ASTER GDEM Version 2 [4] digital elevation model surface	USGS Earth Explorer: https://earthexplorer.usgs.gov/ (Accessed: 2017-04-26)
MODIS Land Cover Type (MCD12Q1) Version 5.1 [5]	Global Land Cover Facility (GLCF): http://glcf.umd.edu/data/lc/ (Accessed: 2017-05-11)
KSS lithology map of Kenya ESRI Shapefile	International Livestock Research Institute (ILRI) GIS services: http://192.156.137.110/gis/search.asp?id=424 (Accessed: 2017-07-25)
KSS properties of Kenya soils ESRI Shapefile	International Livestock Research Institute (ILRI) GIS services: http://192.156.137.110/gis/search.asp?id=419 (Accessed: 2018-05-27)
Worldpop [7] poverty map	Worldpop: https://www.worldpop.org/geodata/summary?id=1262 (Accessed: 2018-04-10)
OpenStreetMap [8] ESRI Shapefiles	Geofabrik http://download.geofabrik.de/africa/kenya.html (Accessed: 2017-04-19)
Towns and urban centres in Kenya ESRI Shapefile	International Livestock Research Institute (ILRI) GIS services: http://192.156.137.110/gis/search.asp?id=280 (Accessed: 2018-05-22)
ACT villages in Kenya ESRI Shapefile	International Livestock Research Institute (ILRI) GIS services: http://192.156.137.110/gis/search.asp?id=372 (Accessed: 2018-05-22)
WDPA protected areas in Kenya ESRI Shapefile	Protected Planet: https://www.protectedplanet.net/country/KE (Accessed: 2018-05-17)
Healthcare facilities in Kenya ESRI Shapefile	International Livestock Research Institute (ILRI) GIS services: http://192.156.137.110/gis/ (Accessed: 2018-05-17)
GHS settlement grid [9]	European Commission Global Human Settlement: https://ghsl.jrc.ec.europa.eu/ghs_smod.php (Accessed: 2018-05-28)
WorldClim 1.4 [10] annual precipitation ESRI grids	WorldClim https://www.worldclim.org/current (Accessed: 2017-04-28)

References

(1) Fan, Y.; Li, H.; Miguez-Macho, G. Global Patterns of Groundwater Table Depth. *Science (80-.).* **2013**, *339* (6122), 940–943.

(2) MacDonald, A. M.; Bonsor, H. C.; Dochartaigh, B. É. Ó.; Taylor, R. G. Quantitative Maps of Groundwater Resources in Africa. *Environ. Res. Lett.* **2012**, *7* (2), 1–7.

(3) Persits, F.; Ahlbrandt, T.; Tuttle, M.; Charpentier, R.; Brownfield, M.; Takahashi, K. *Map Showing Geology, Oil and Gas Fields, and Geologic Provinces of Africa, U.S. Geological Survey Open File Report 97-470A, Version 2.0*; 2002.

(4) NASA/METI/AIST/Japan Spacesystems; U.S./Japan ASTER Science Team. ASTER Global Digital Elevation Model [Dataset]. NASA EOSDIS Land Processes DAAC 2009.

(5) Friedl, M. A.; Sulla-Menashe, D.; Tan, B.; Schneider, A.; Ramankutty, N.; Sibley, A.; Huang, X. MODIS Collection 5 Global Land Cover: Algorithm Refinements and Characterization of New Datasets. *Remote Sens. Environ.* **2010**, *114* (1), 168–182.

(6) Alkire, S.; Foster, J.; Seth, S.; Santos, M. E.; Roche, J. M.; Ballon, P. *Multidimensional Poverty Measurement and Analysis*; Oxford University Press, 2015.

(7) Tatem, A. J.; Gething, P. W.; Bhatt, S.; Weiss, D.; Pezzulo, C. Pilot High Resolution Poverty Maps. University of Southampton/Oxford 2013.

(8) OpenStreetMap contributors. OpenStreetMap http://www.openstreetmap.org (accessed Jan 1, 2017).

(9) Pesaresi, M.; Freire, S. GHS Settlement Grid Following the REGIO Model 2014 in Application to GHSL Landsat and CIESIN GPW v4 -Multitemporal (1975-1990-2000-2015) [Dataset]. European Commission, Joint Research Centre (JRC) 2016.

(10) Hijmans, R. J.; Cameron, S. E.; Parra, J. L.; Jones, P. G.; Jarvis, A. Very High Resolution Interpolated Climate Surfaces for Global Land Areas. *Int. J. Climatol.* **2005**, *25* (15), 1965–1978.

2. Additional details on MaxEnt predictions

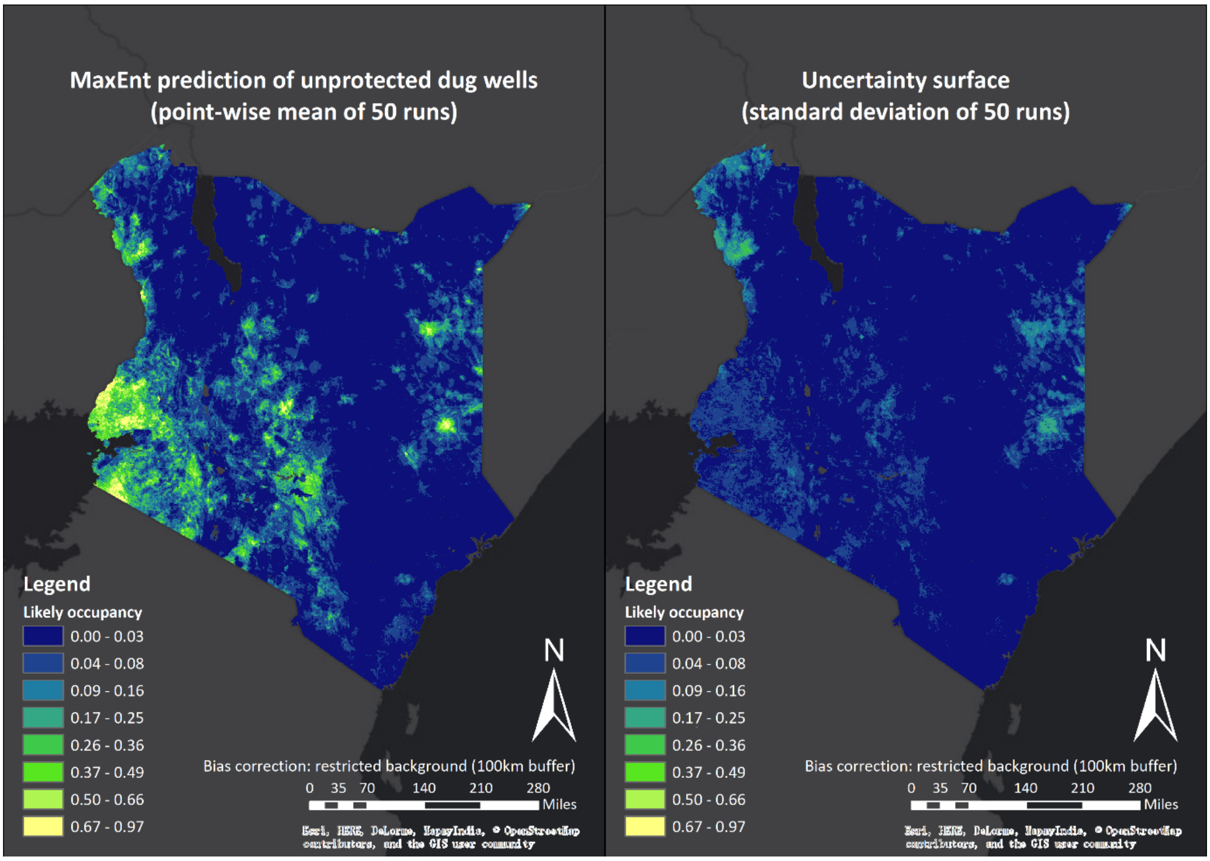

Figure S1_File.3. Distribution of unprotected dug wells predicted by MaxEnt (Left) and associated uncertainty (Right) based on restricted background method of bias correction

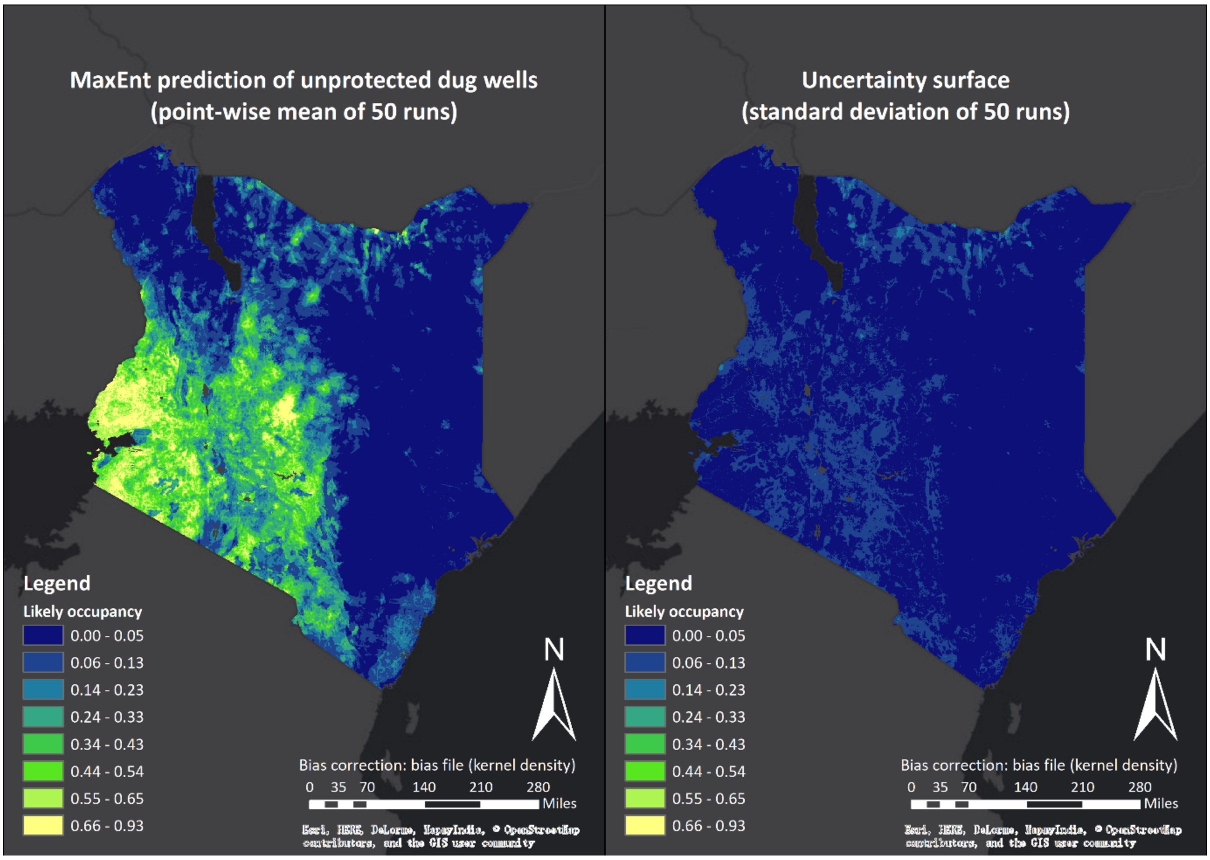

Figure S1_File.4. Distribution of unprotected dug wells predicted by MaxEnt (Left) and associated uncertainty (Right) based on bias file method of bias correction

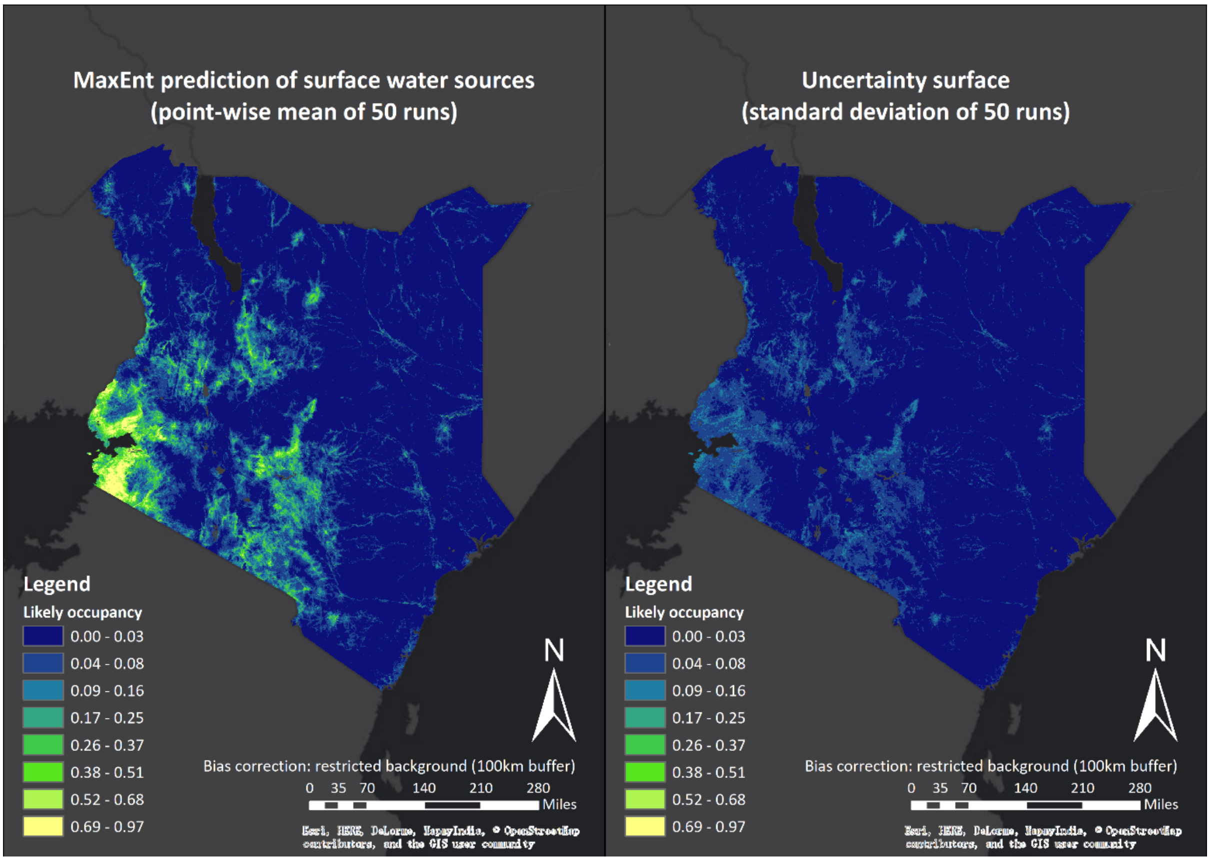

Figure S1_File.5. Distribution of surface water sources predicted by MaxEnt (Left) and associated uncertainty (Right) based on restricted background method of bias correction

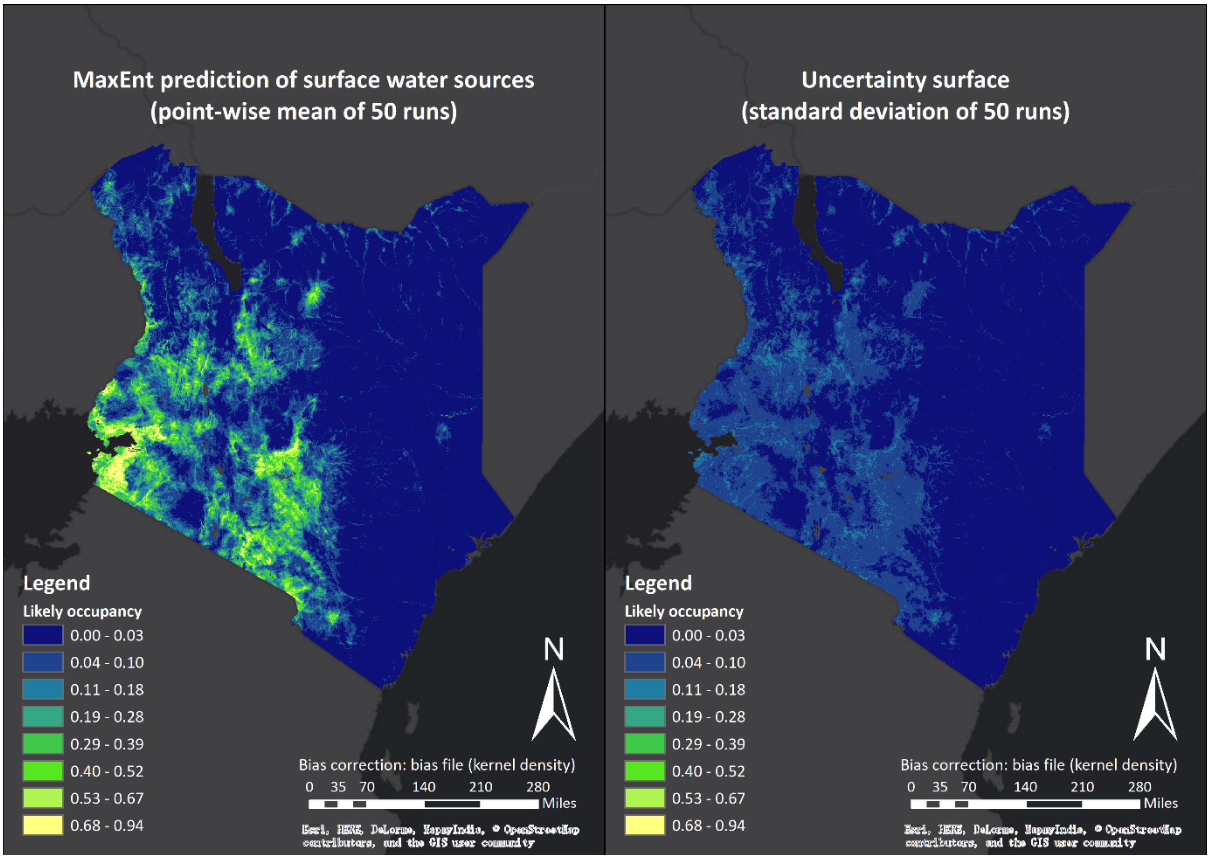

Figure S1_File.6. Distribution of surface water sources predicted by MaxEnt (Left) and associated uncertainty (Right) based on bias file method of bias correction
