# Supplementary material for: Mapping access to domestic water supplies from incomplete data in developing countries: An illustrative assessment for Kenya
Source: PLoS One. 2019 May 17;14(5):e0216923. doi: 10.1371/journal.pone.0216923 (PMC6524943; doi:10.1371/journal.pone.0216923)
Supplement: S1 Fig — (PDF) [file pone.0216923.s003.pdf]

S1 Fig. Graphs of response curves

Unprotected dug wells (restricted background)

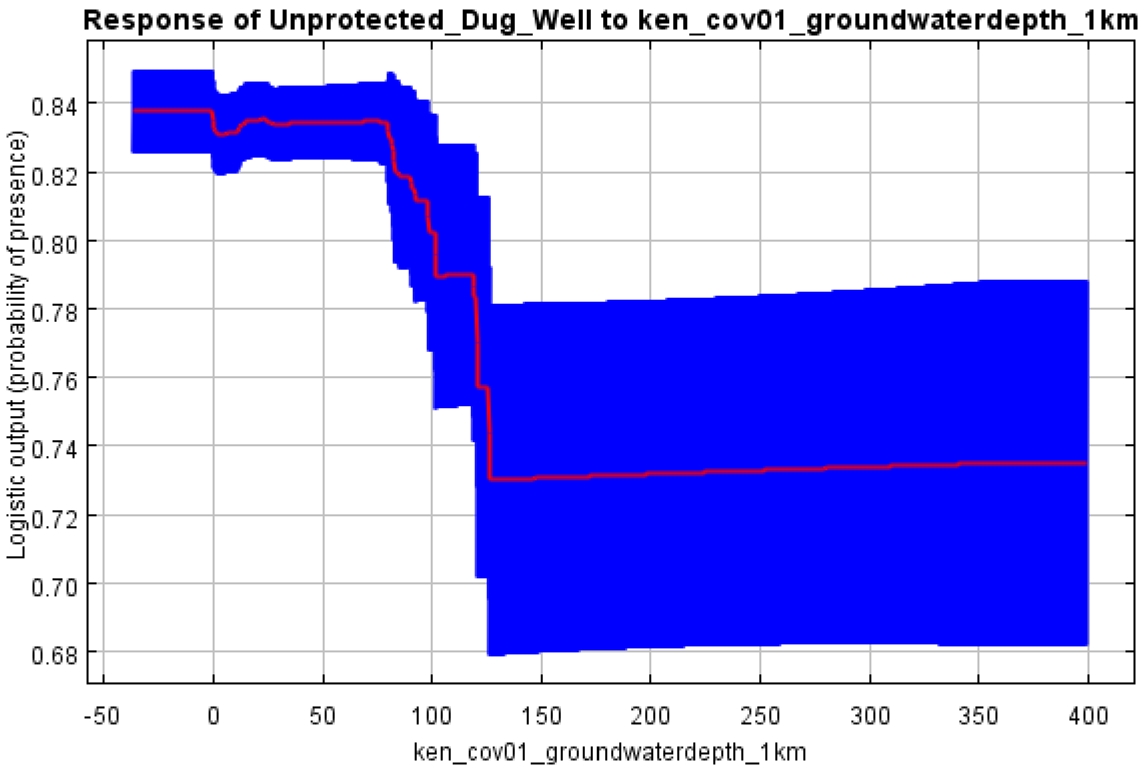

S1_Fig.1. Response curve of groundwater depth presented as means (red) of 50 replicate runs with standard deviation in blue; model built with other predictive covariates being kept at their average sample values. X-axis: depth to groundwater table (m).

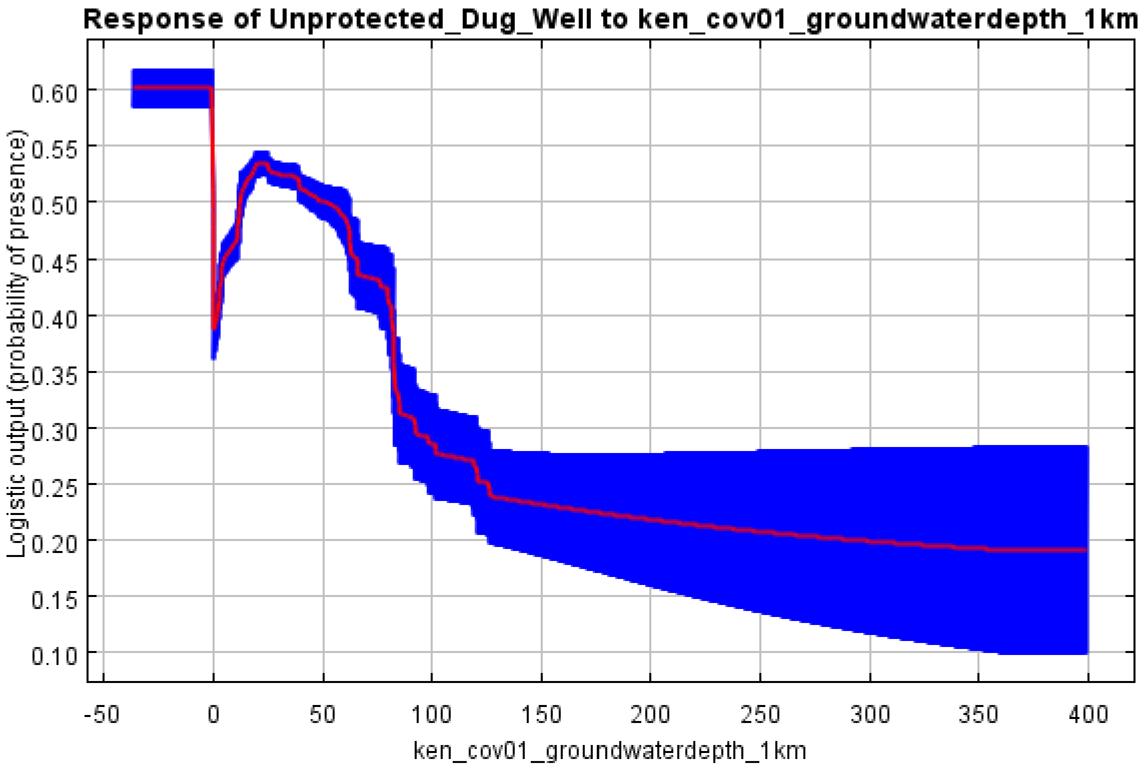

S1_Fig.2. Response curve of groundwater depth presented as means (red) of 50 replicate runs with standard deviation in blue; model built without other predictive covariates. X-axis: depth to groundwater table (m).

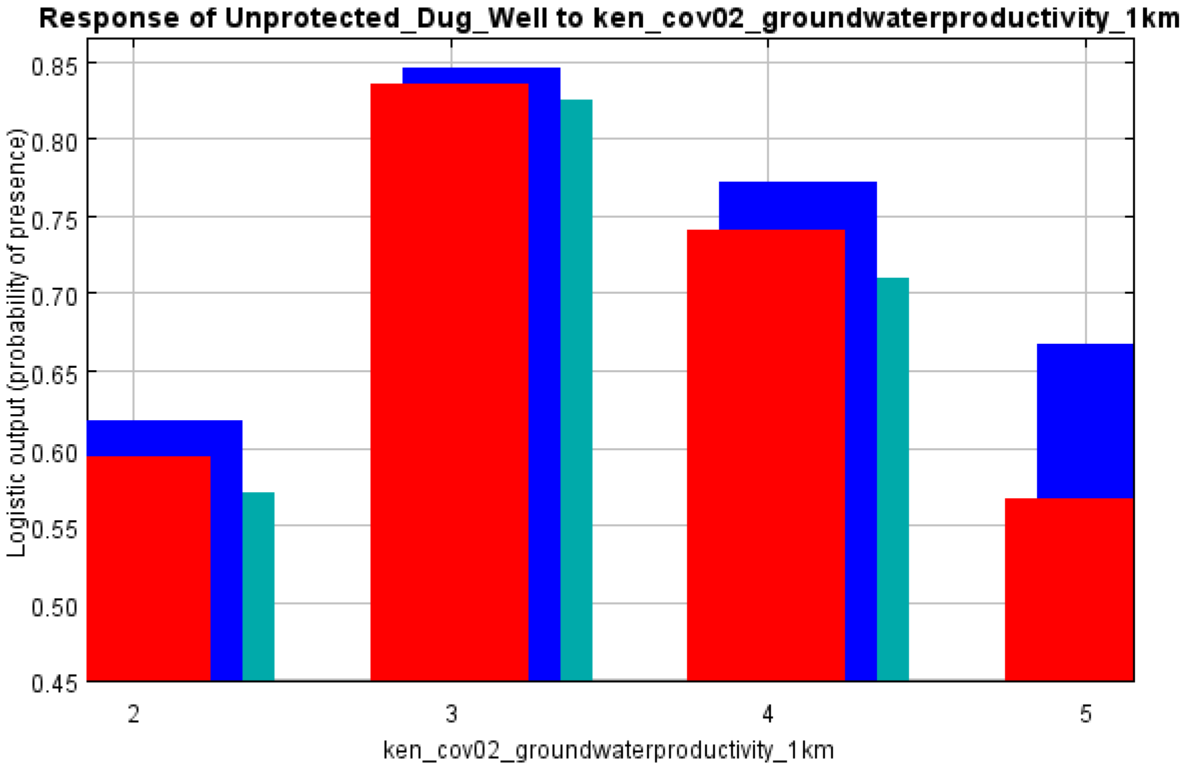

S1_Fig.3. Response curve of groundwater productivity presented as means (red) of 50 replicate runs with standard deviation in blue; model built with other predictive covariates being kept at their average sample values. X-axis: productivity: 2 – Low (0.1-0.5 l/s); 3 – Low-Moderate (0.5-1 l/s); 4 – Moderate (1-5 l/s); 5 – High (5-20 l/s).

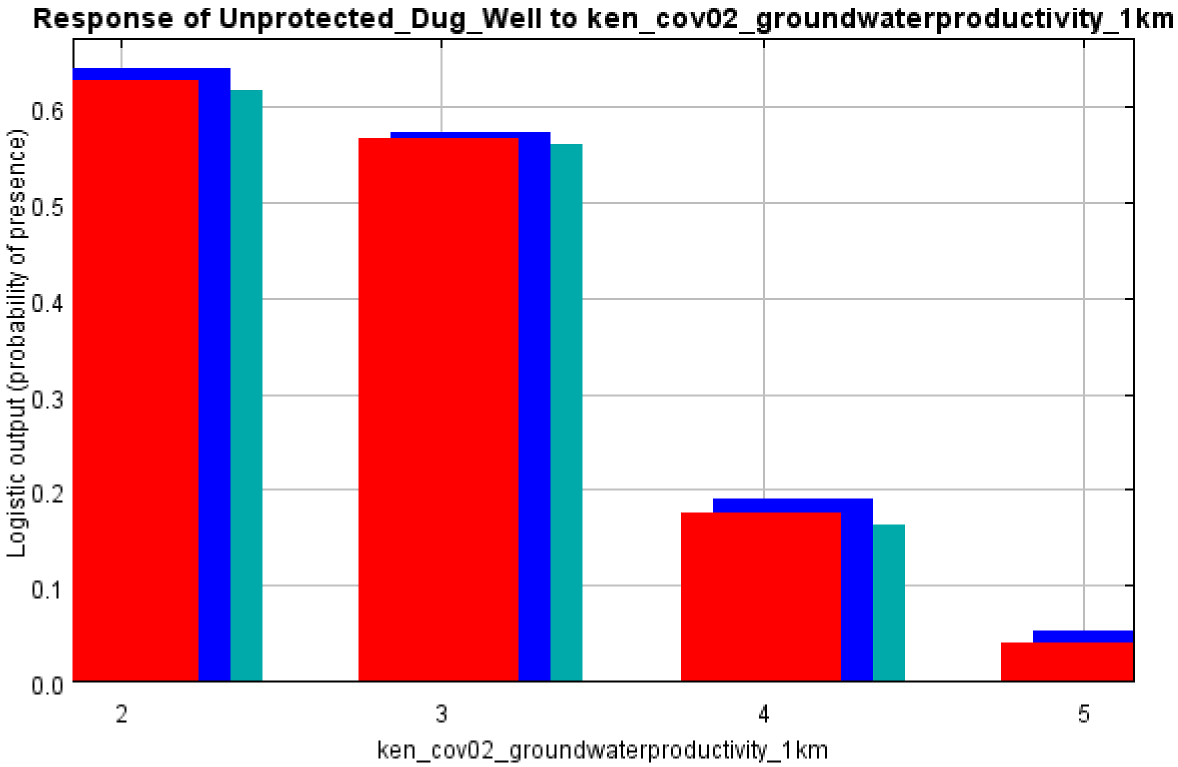

S1_Fig.4. Response curve of groundwater productivity presented as means (red) of 50 replicate runs with standard deviation in blue; model built without other predictive covariates. X-axis: productivity: 2 – Low (0.1-0.5 l/s); 3 – Low-Moderate (0.5-1 l/s); 4 – Moderate (1-5 l/s); 5 – High (5-20 l/s).

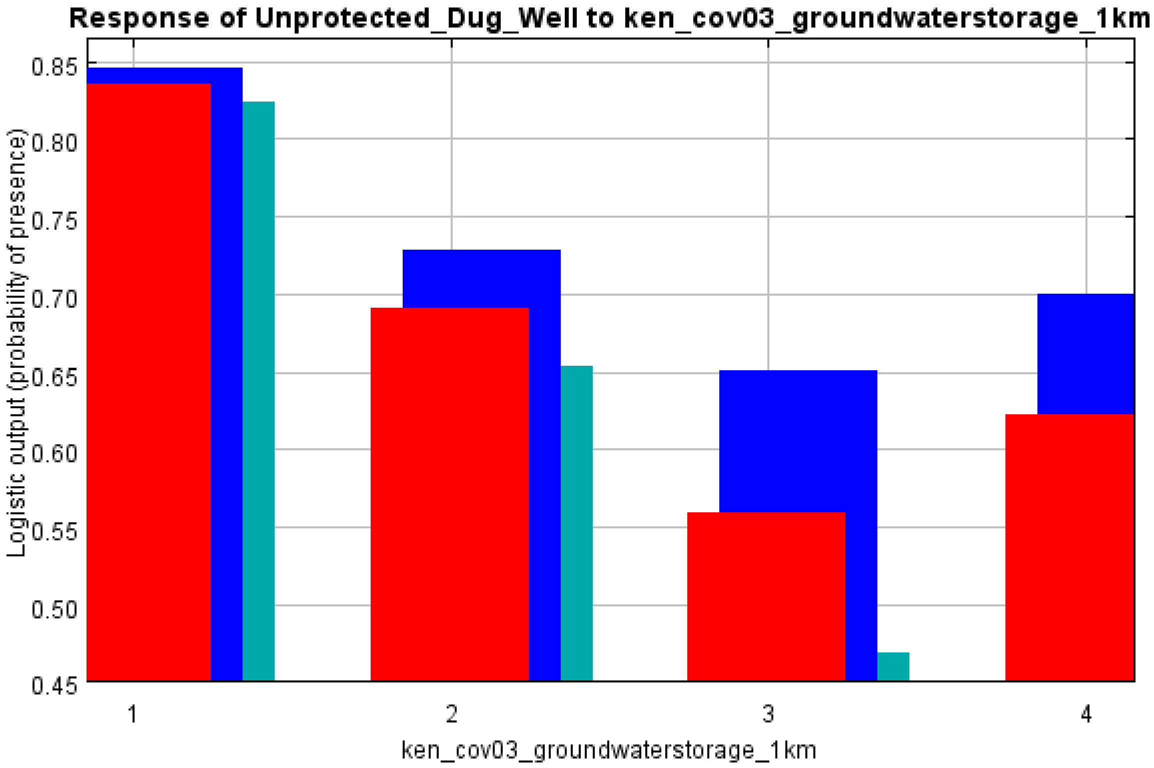

S1_Fig.5. Response curve of groundwater storage presented as means (red) of 50 replicate runs with standard deviation in blue; model built with other predictive covariates being kept at their average sample values. X-axis: storage (water depth in mm): 1 – Low (<1000); 2 – Low-Moderate (1000-10,000); 3 – Moderate (10,000-25,000); 4 – High (25,000-50,000).

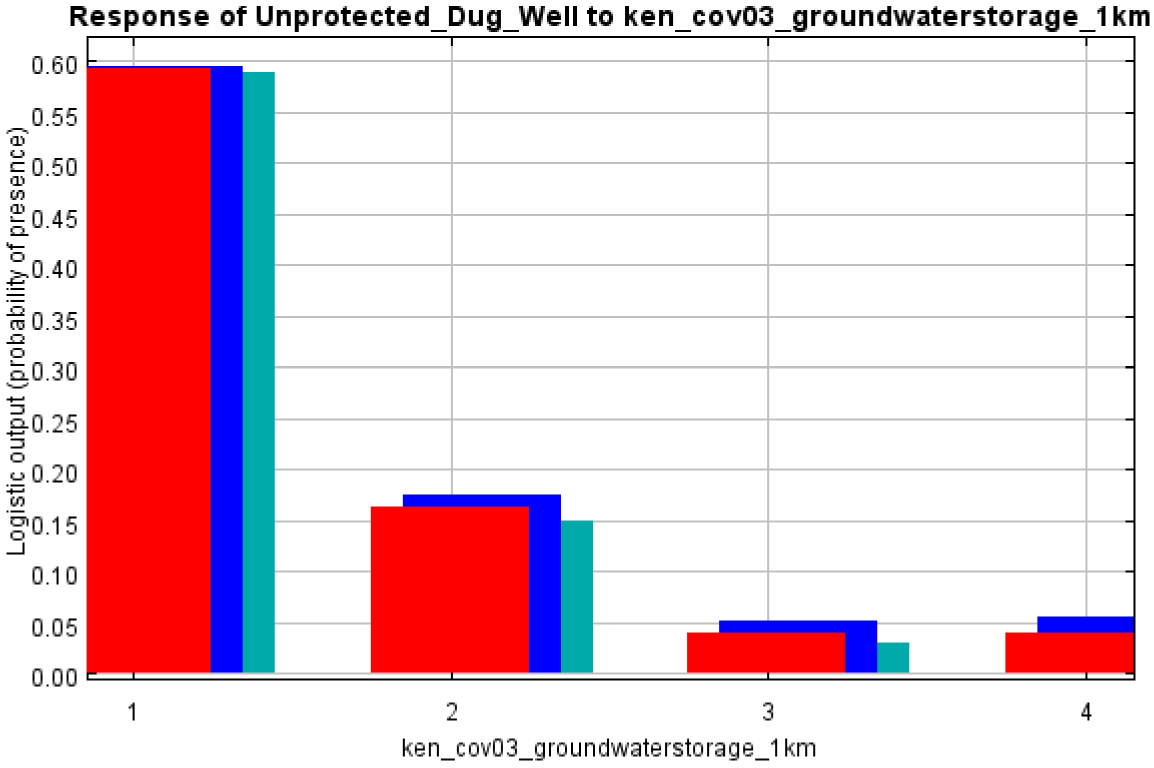

S1_Fig.6. Response curve of groundwater storage presented as means (red) of 50 replicate runs with standard deviation in blue; model built without other predictive covariates. X-axis: storage (water depth in mm): 1 – Low (<1000); 2 – Low-Moderate (1000-10,000); 3 – Moderate (10,000-25,000); 4 – High (25,000-50,000).

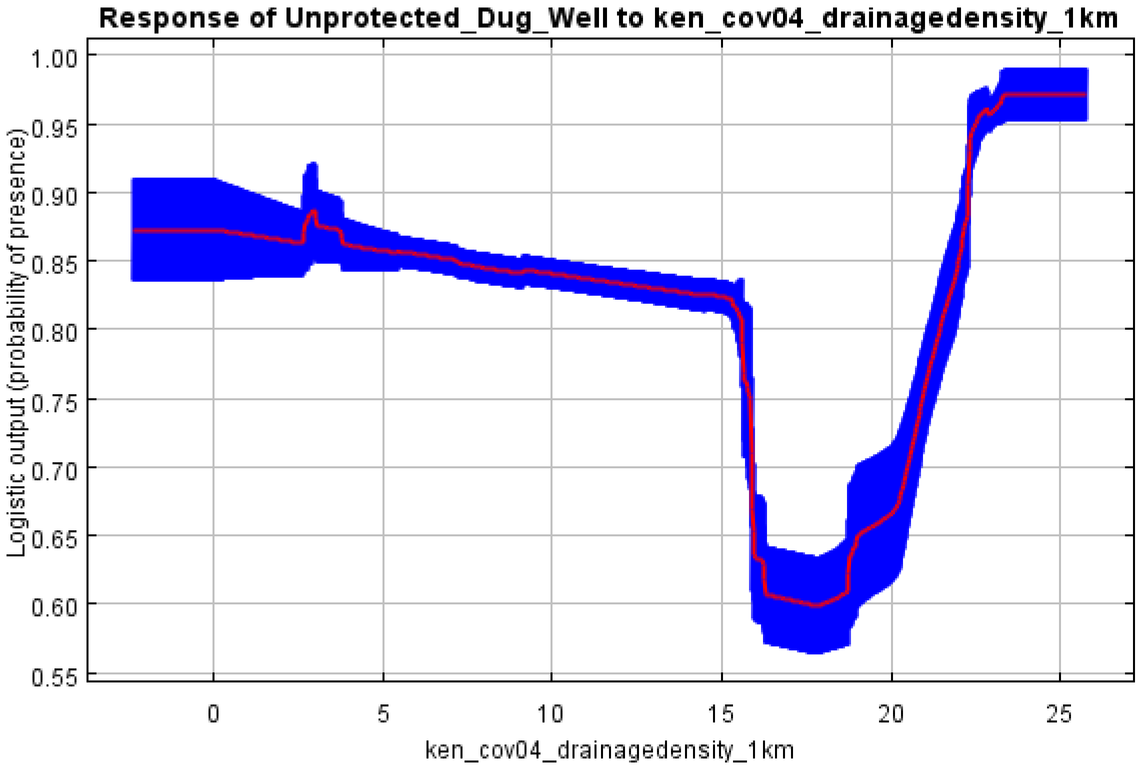

S1_Fig.7. Response curve of drainage density presented as means (red) of 50 replicate runs with standard deviation in blue; model built with other predictive covariates being kept at their average sample values. X-axis: drainage density – channel length per area size of a grid cell (lengths in decimal degrees).

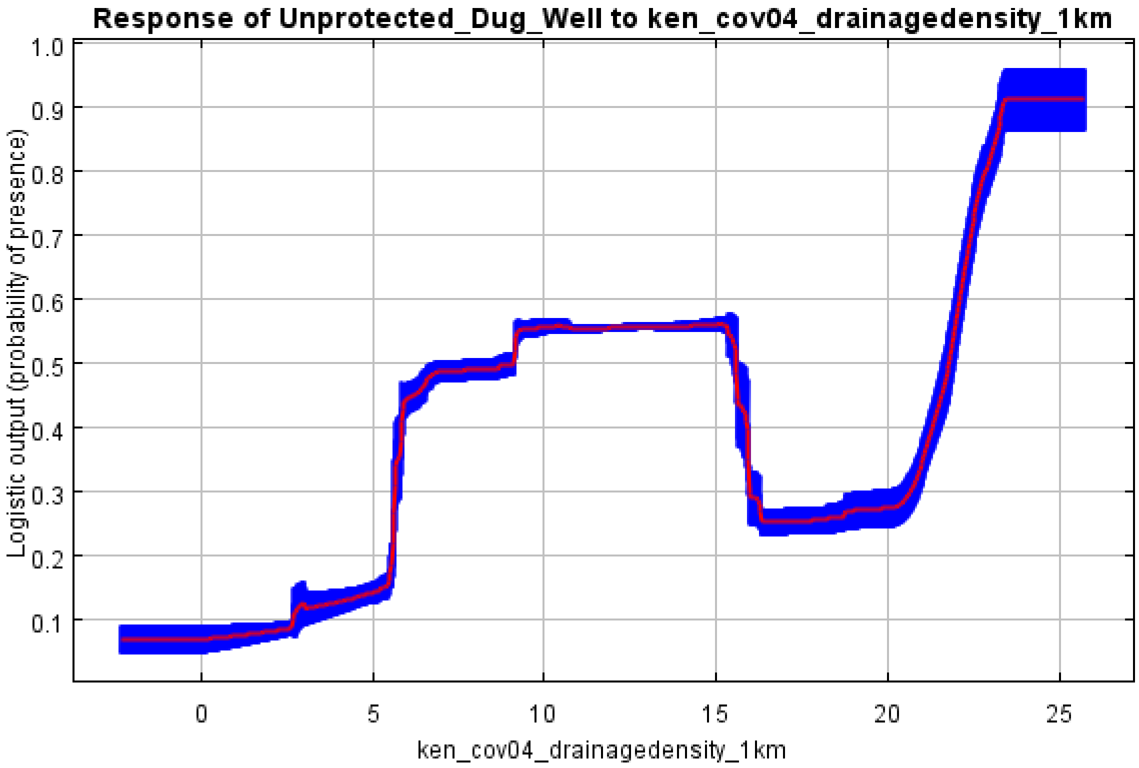

S1_Fig.8. Response curve of drainage density presented as means (red) of 50 replicate runs with standard deviation in blue; model built without other predictive covariates. X-axis: drainage density – channel length per area size of a grid cell (lengths in decimal degrees).

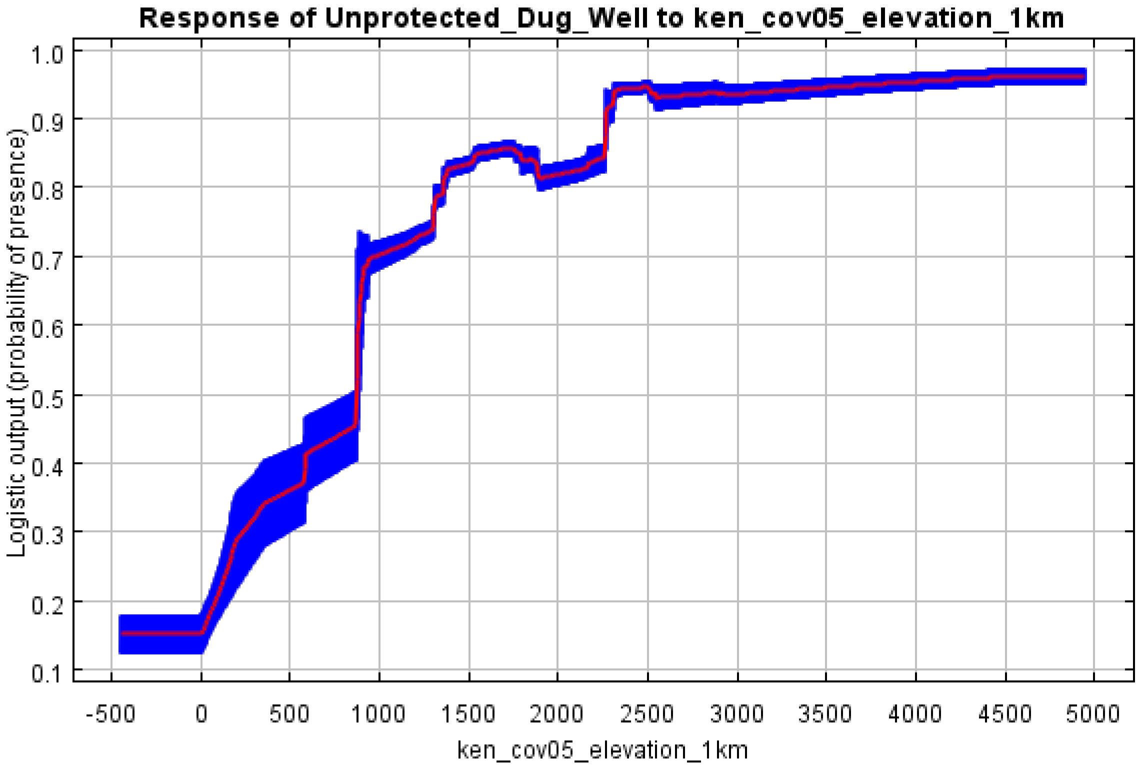

S1_Fig.9. Response curve of elevation presented as means (red) of 50 replicate runs with standard deviation in blue; model built with other predictive covariates being kept at their average sample values. X-axis: elevation (m).

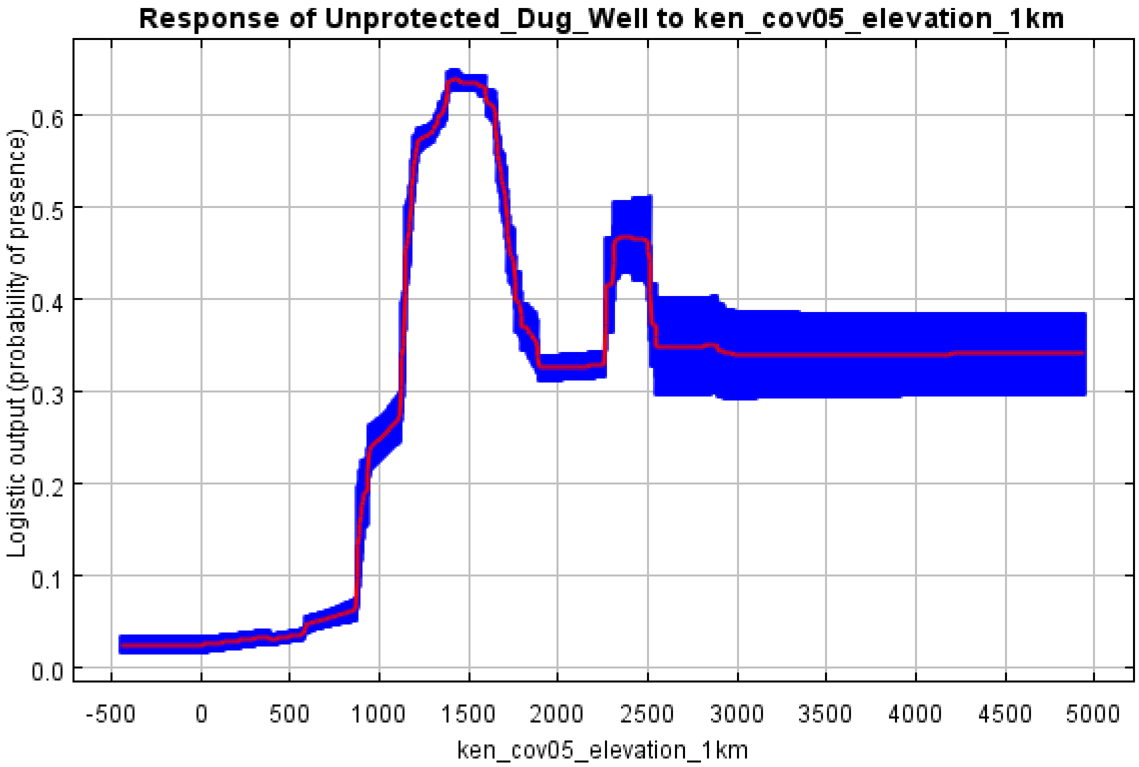

S1_Fig.10. Response curve of elevation presented as means (red) of 50 replicate runs with standard deviation in blue; model built without other predictive covariates. X-axis: elevation (m).

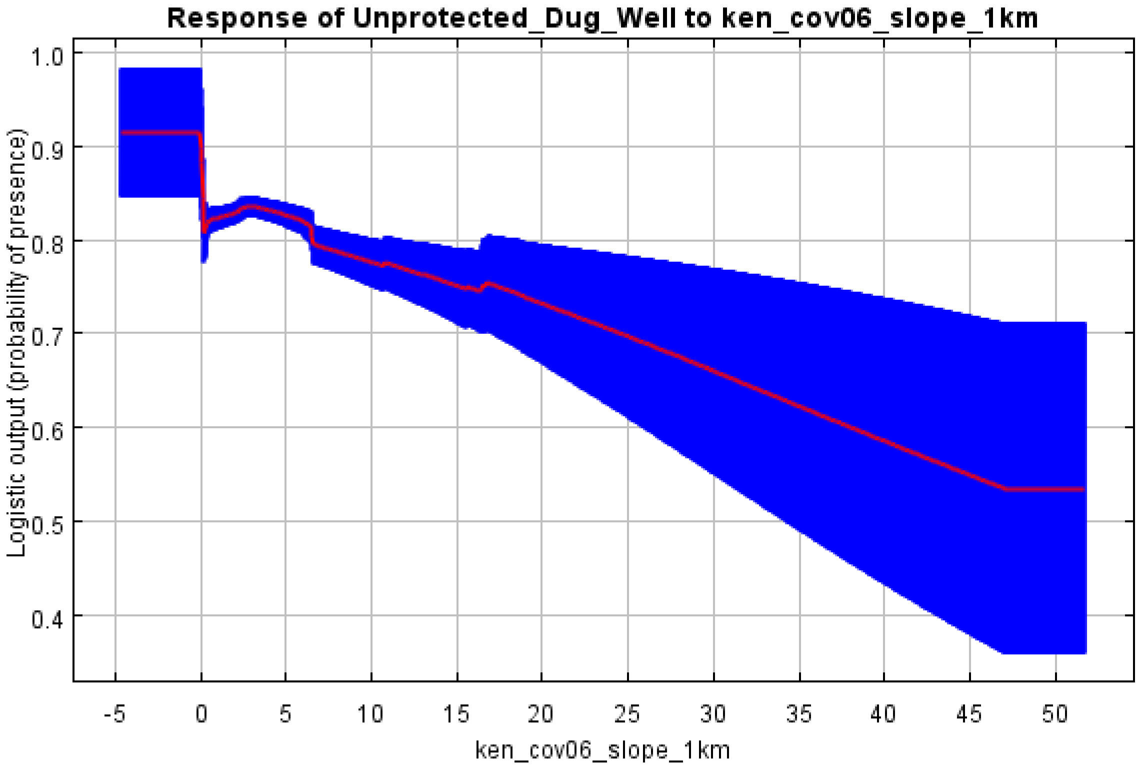

S1_Fig.11. Response curve of slope presented as means (red) of 50 replicate runs with standard deviation in blue; model built with other predictive covariates being kept at their average sample values. X-axis: slope (degree).

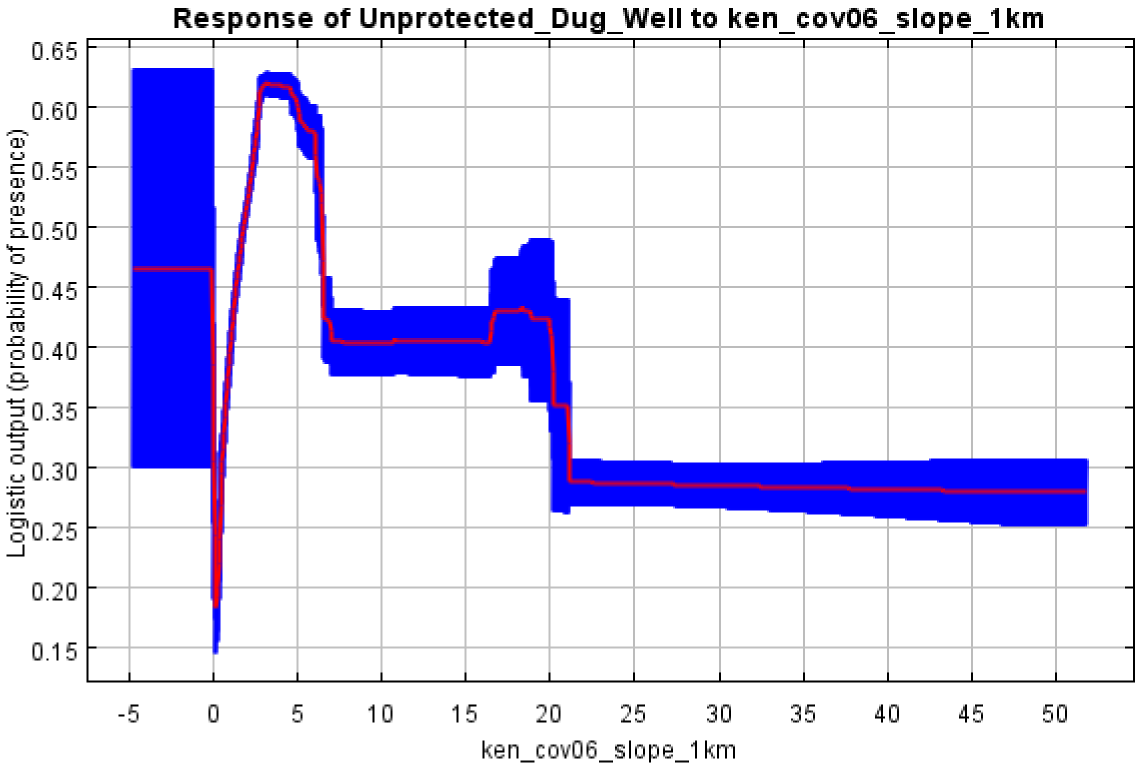

S1_Fig.12. Response curve of slope presented as means (red) of 50 replicate runs with standard deviation in blue; model built without other predictive covariates. X-axis: slope (degree).

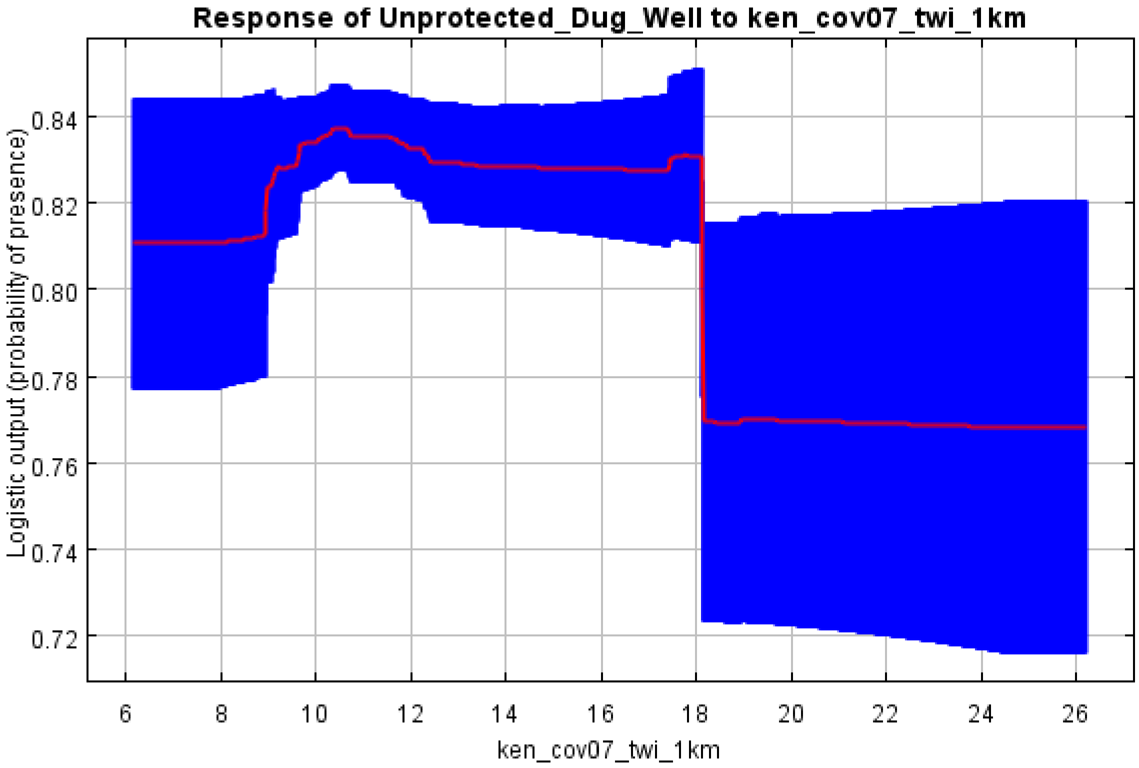

S1_Fig.13. Response curve of topographic wetness index presented as means (red) of 50 replicate runs with standard deviation in blue; model built with other predictive covariates being kept at their average sample values. X-axis: topographic unit index.

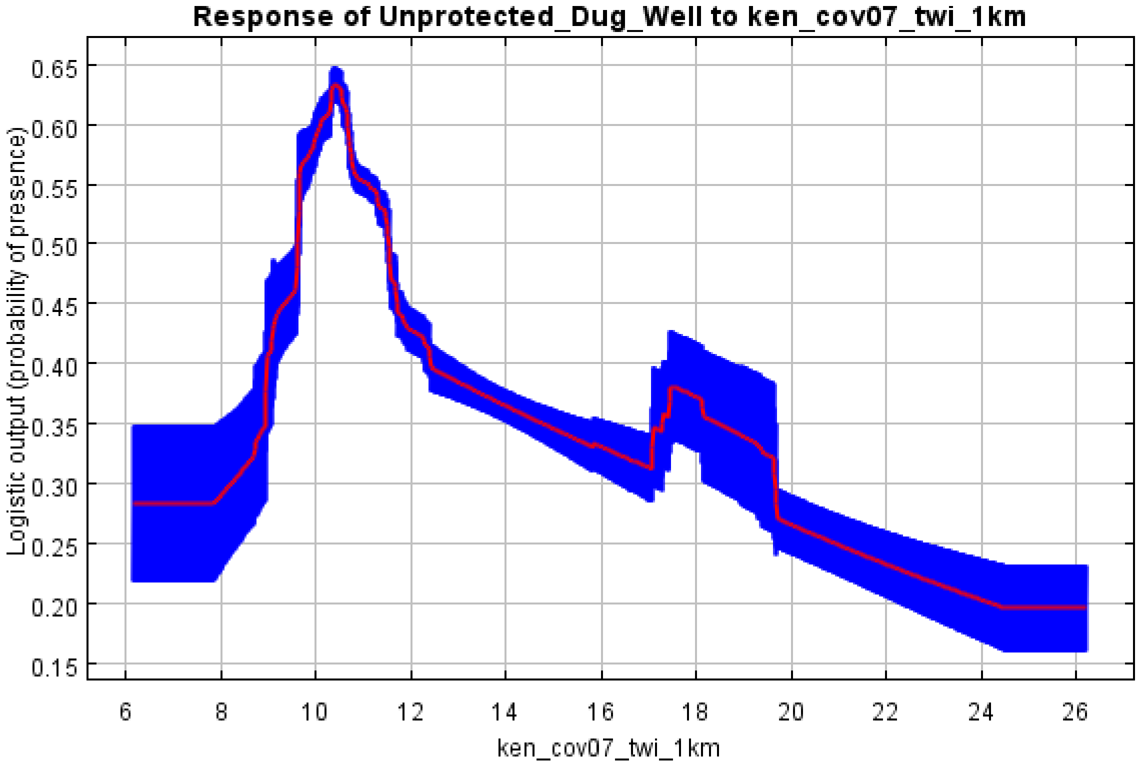

S1_Fig.14. Response curve of topographic wetness index presented as means (red) of 50 replicate runs with standard deviation in blue; model built without other predictive covariates. X-axis: topographic unit index.

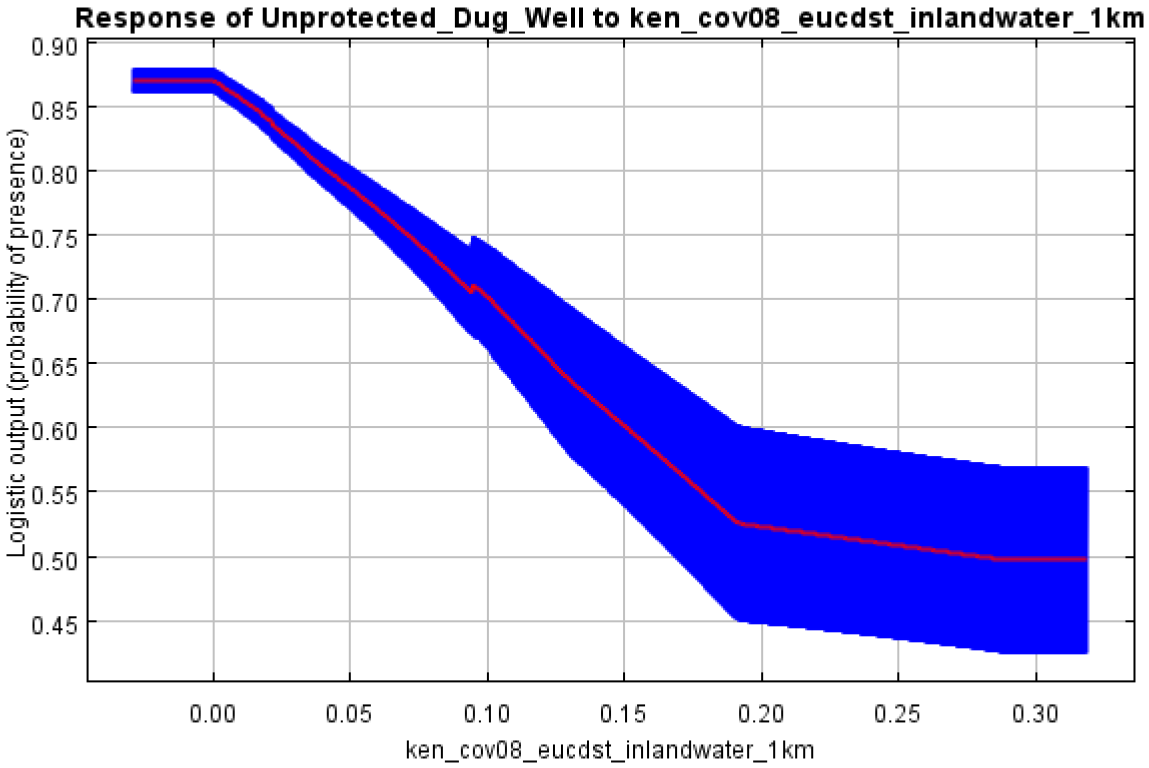

S1_Fig.15. Response curve of Euclidean distance to inland water presented as means (red) of 50 replicate runs with standard deviation in blue; model built with other predictive covariates being kept at their average sample values. X-axis: Euclidean distance (decimal degrees).

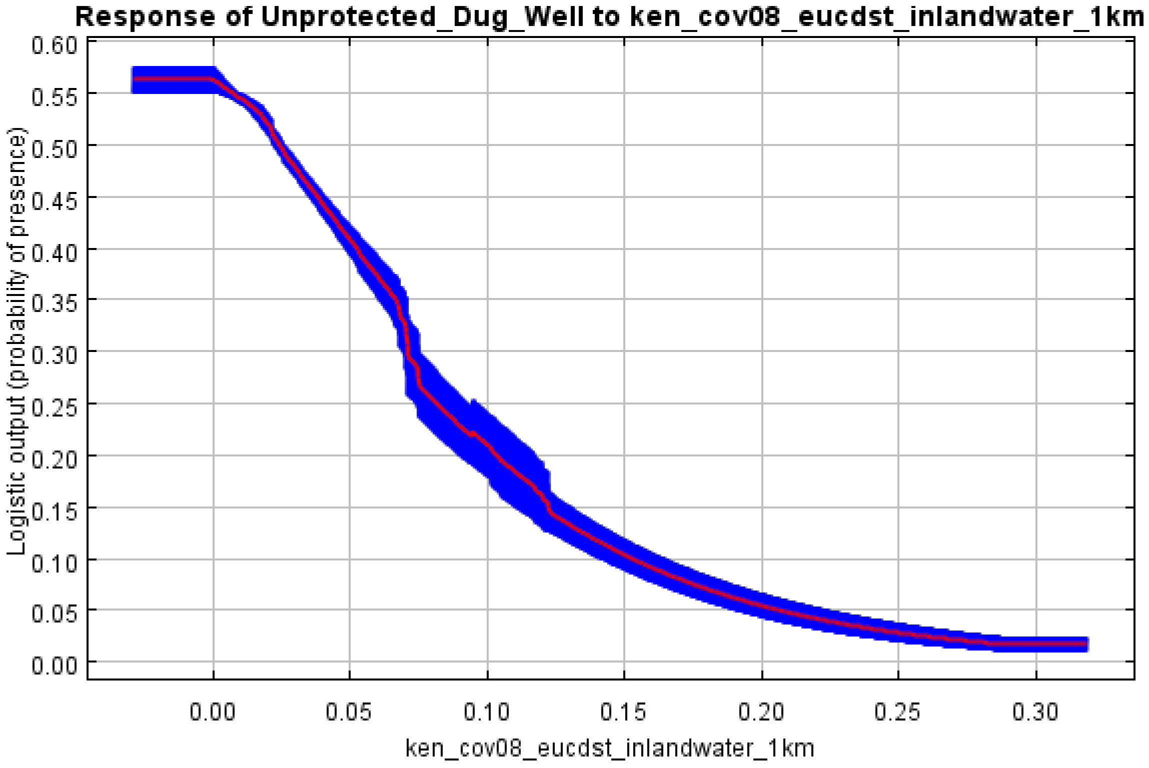

S1_Fig.16. Response curve of Euclidean distance to inland water presented as means (red) of 50 replicate runs with standard deviation in blue; model built without other predictive covariates. X-axis: Euclidean distance (decimal degrees).

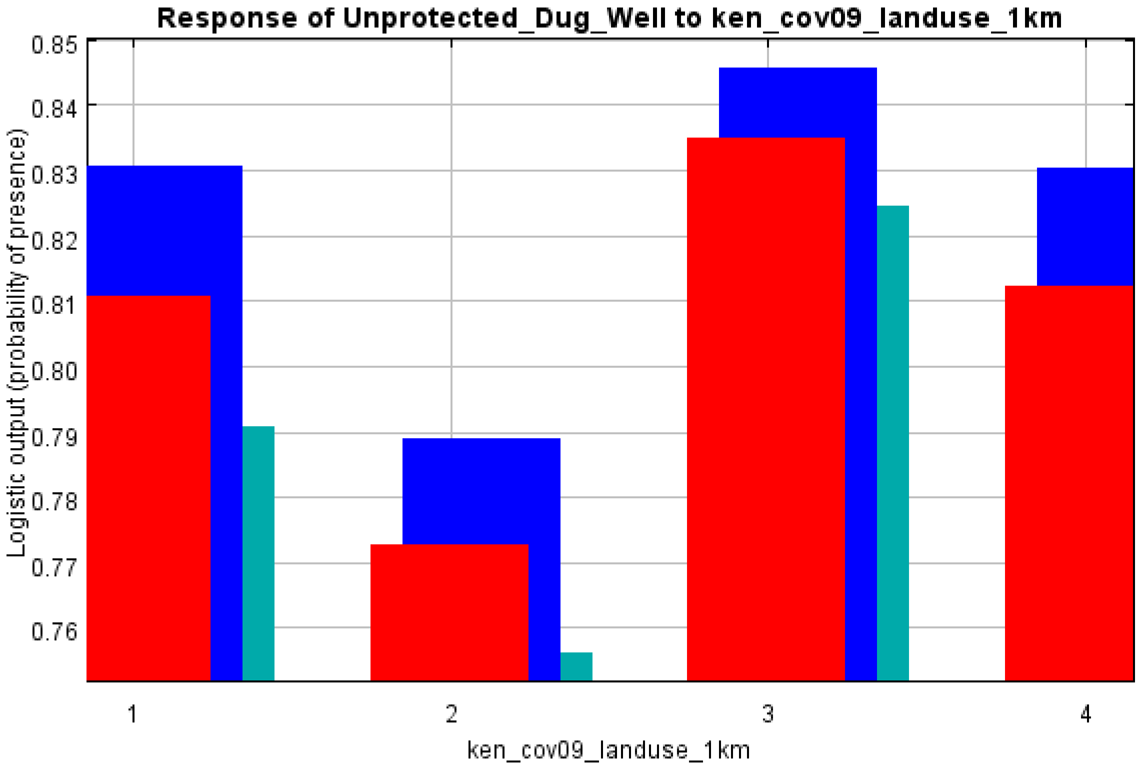

S1_Fig.17. Response curve of land use presented as means (red) of 50 replicate runs with standard deviation in blue; model built with other predictive covariates being kept at their average sample values. X-axis: land use: 1 – forest; 2 – others; 3 – agricultural land; 4 – urban area.

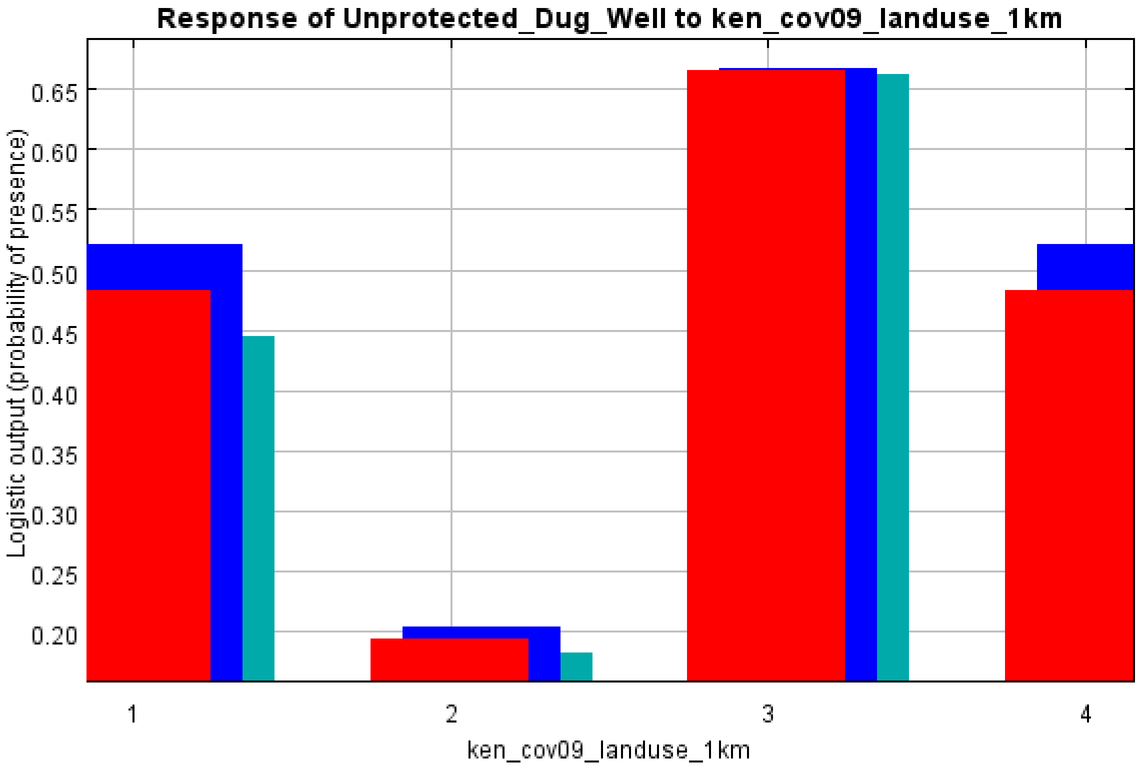

S1_Fig.18. Response curve of land use presented as means (red) of 50 replicate runs with standard deviation in blue; model built without other predictive covariates. X-axis: land use: 1 – forest; 2 – others; 3 – agricultural land; 4 – urban area.

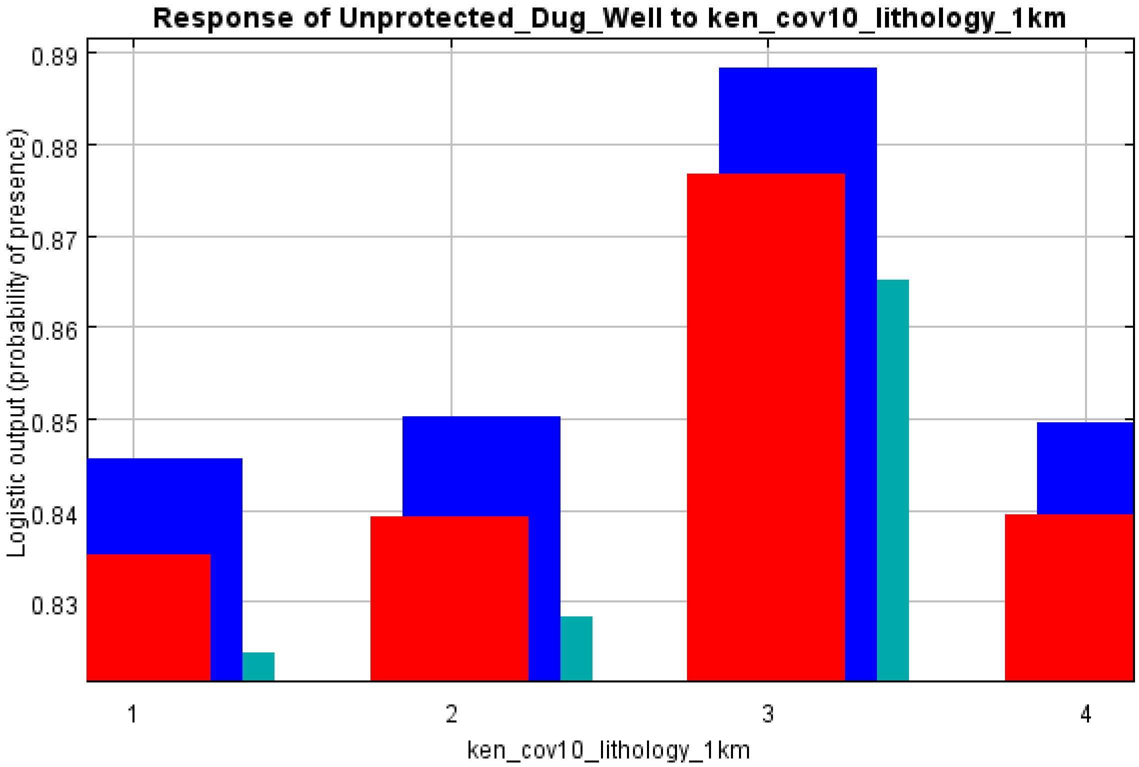

S1_Fig.19. Response curve of lithology presented as means (red) of 50 replicate runs with standard deviation in blue; model built with other predictive covariates being kept at their average sample values. X-axis: lithology: 1 – Igneous; 2 – Metamorphic rock; 3 – Sedimentary rock; 4 – Unconsolidated.

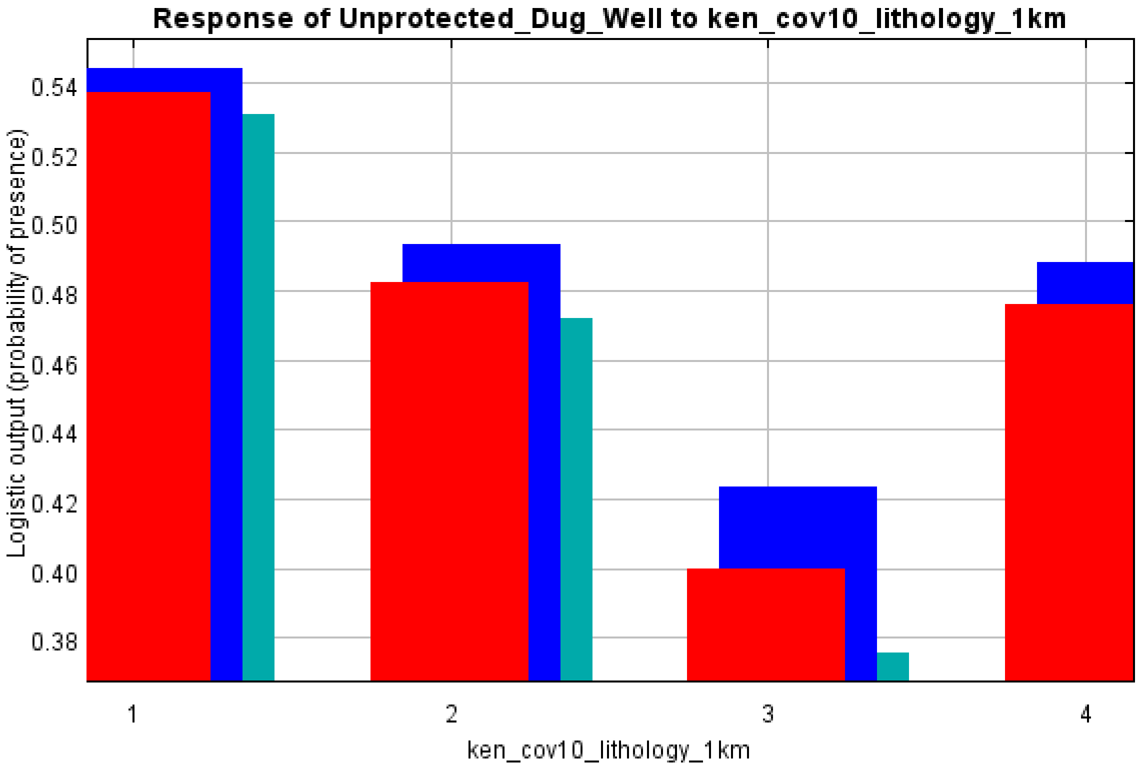

S1_Fig.20. Response curve of lithology presented as means (red) of 50 replicate runs with standard deviation in blue; model built without other predictive covariates. X-axis: lithology: 1 – Igneous; 2 – Metamorphic rock; 3 – Sedimentary rock; 4 – Unconsolidated.

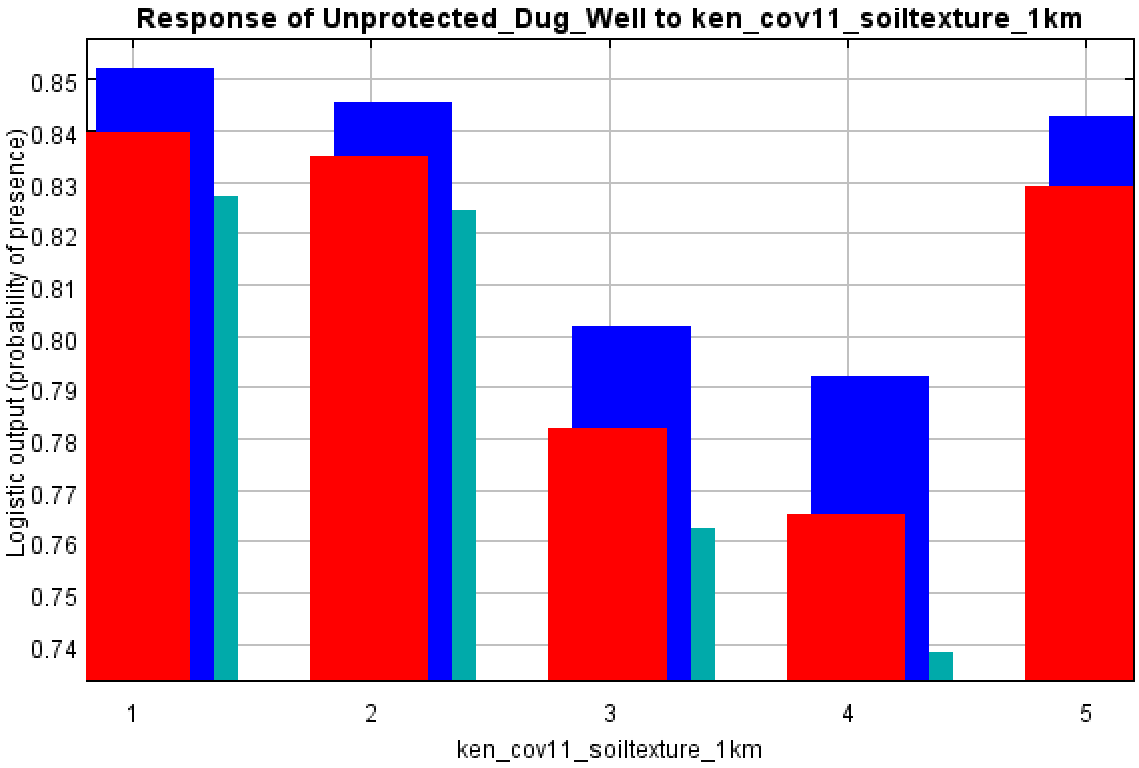

S1_Fig.21. Response curve of soil texture presented as means (red) of 50 replicate runs with standard deviation in blue; model built with other predictive covariates being kept at their average sample values. X-axis: lithology: 1 – Very clayey; 2 – Clayey; 3 – Loamy; 4 – Sandy; 5 – Extremely sandy.

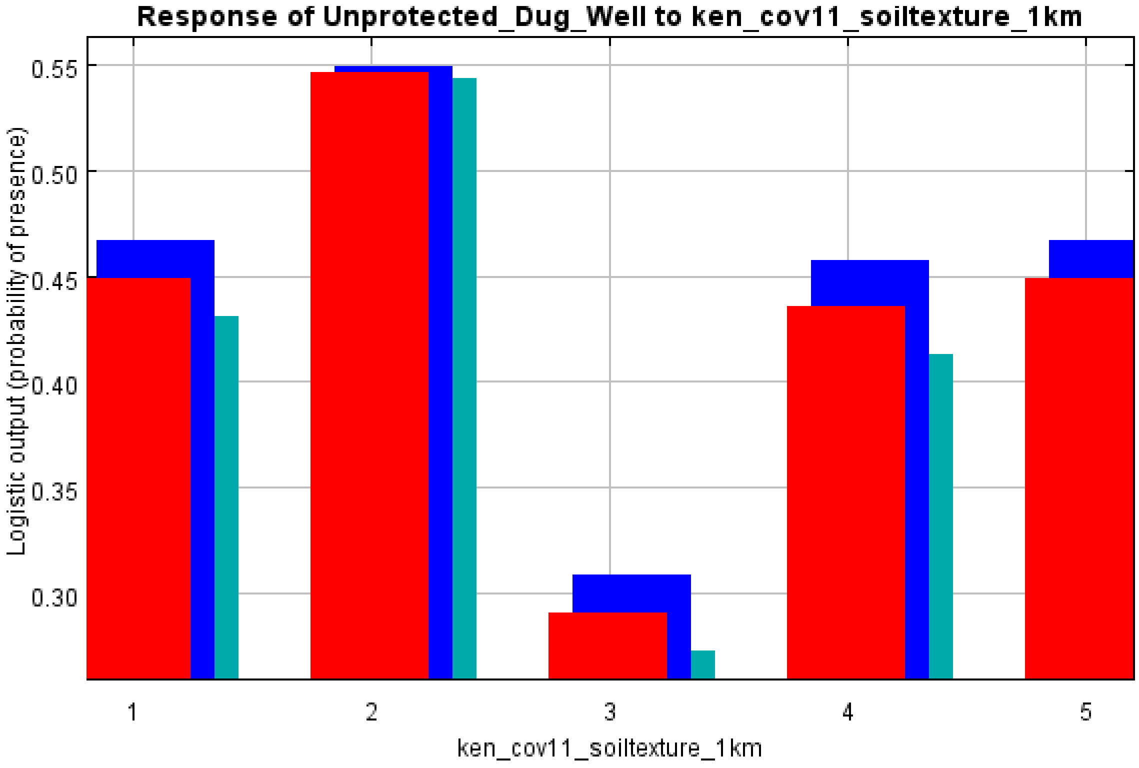

S1_Fig.22. Response curve of soil texture presented as means (red) of 50 replicate runs with standard deviation in blue; model built without other predictive covariates. X-axis: lithology: 1 – Very clayey; 2 – Clayey; 3 – Loamy; 4 – Sandy; 5 – Extremely sandy.

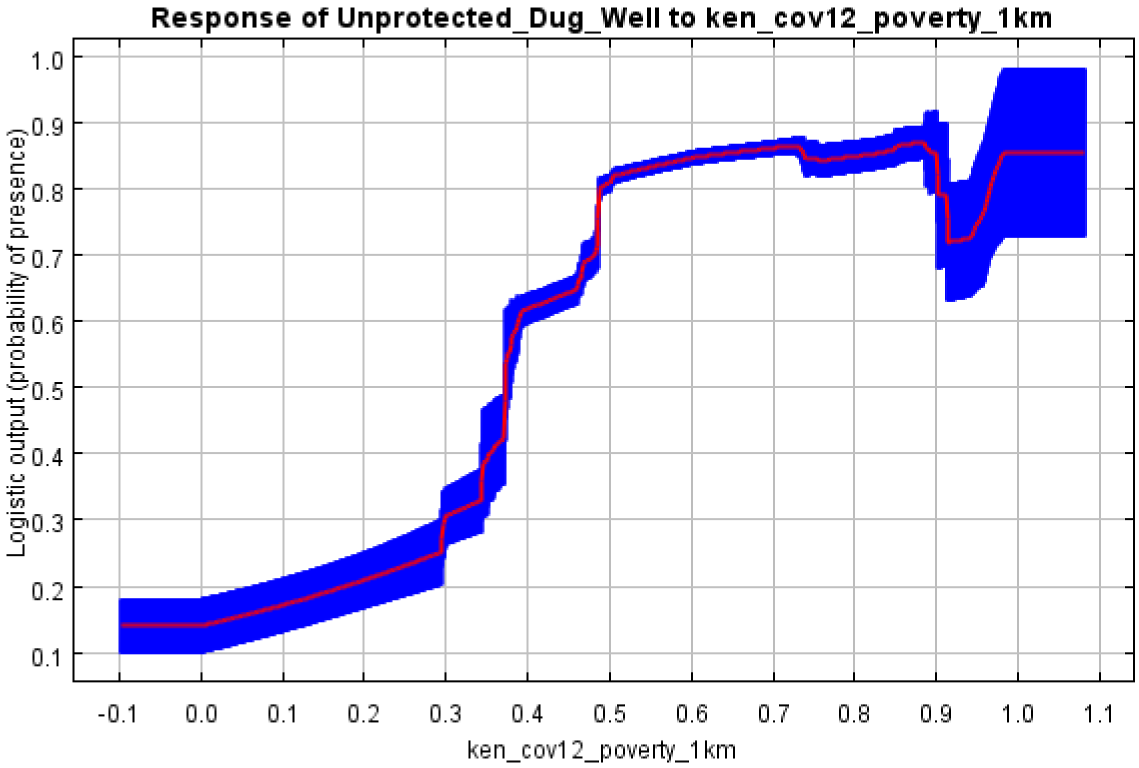

S1_Fig.23. Response curve of poverty presented as means (red) of 50 replicate runs with standard deviation in blue; model built with other predictive covariates being kept at their average sample values. X-axis: proportion of residents living in MPI-defined poverty.

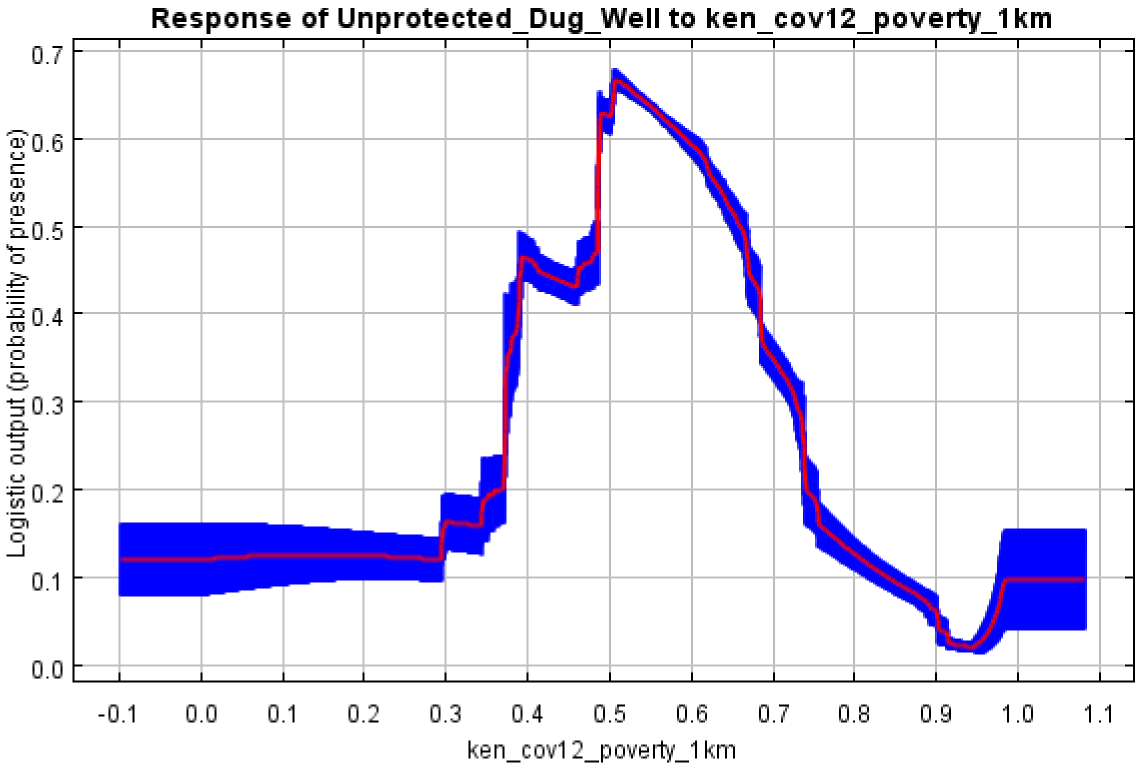

S1_Fig.24. Response curve of poverty presented as means (red) of 50 replicate runs with standard deviation in blue; model built without other predictive covariates. X-axis: proportion of residents living in MPI-defined poverty.

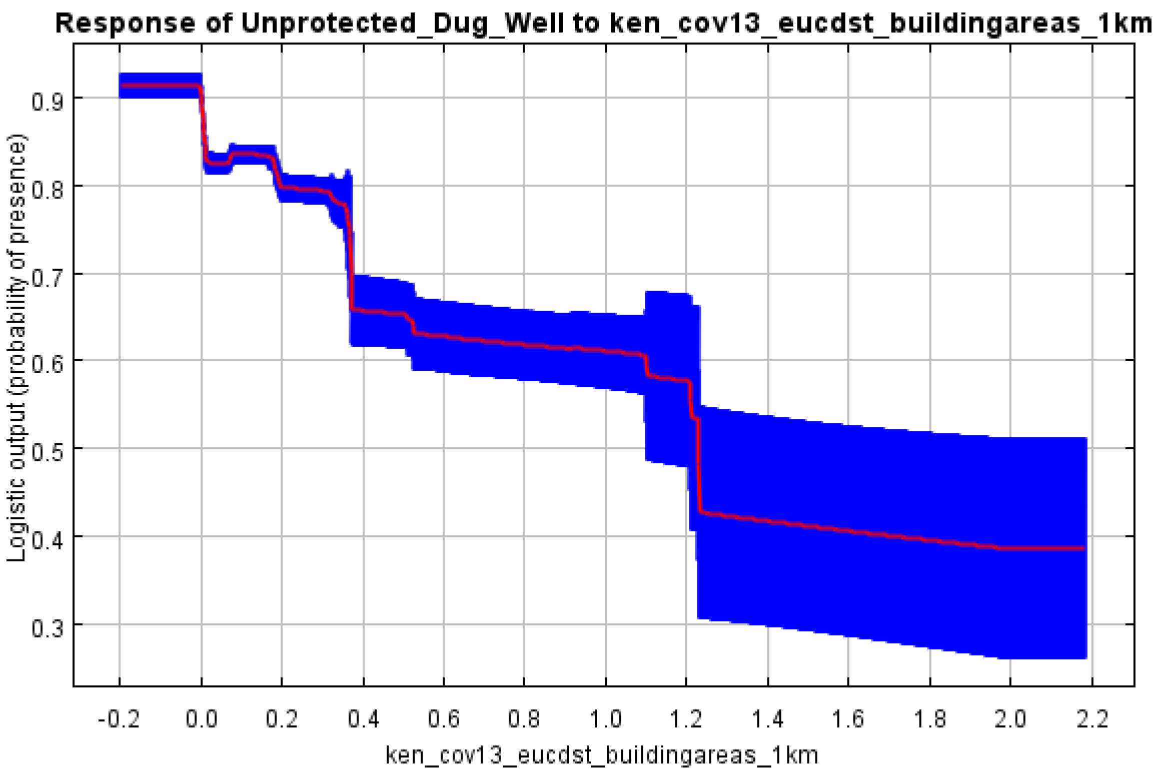

S1_Fig.25. Response curve of Euclidean distance to buildings presented as means (red) of 50 replicate runs with standard deviation in blue; model built with other predictive covariates being kept at their average sample values. X-axis: Euclidean distance (decimal degrees).

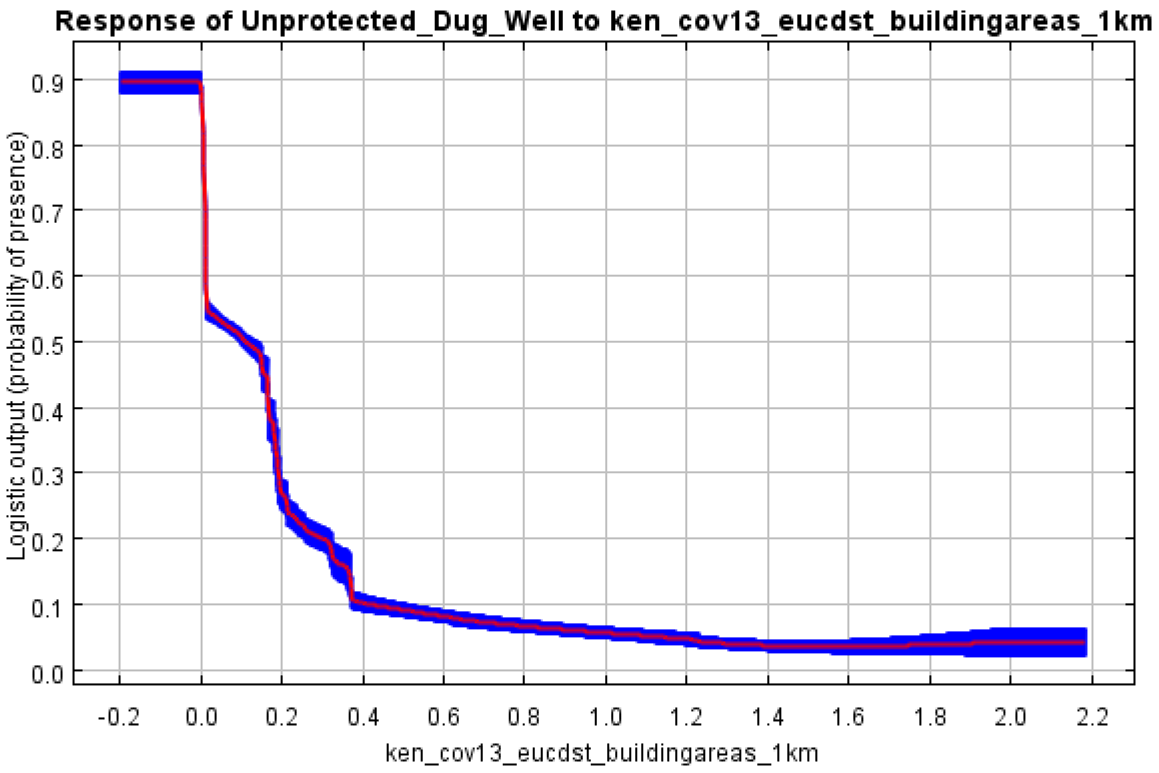

S1_Fig.26. Response curve of Euclidean distance to buildings presented as means (red) of 50 replicate runs with standard deviation in blue; model built without other predictive covariates. X-axis: Euclidean distance (decimal degrees).

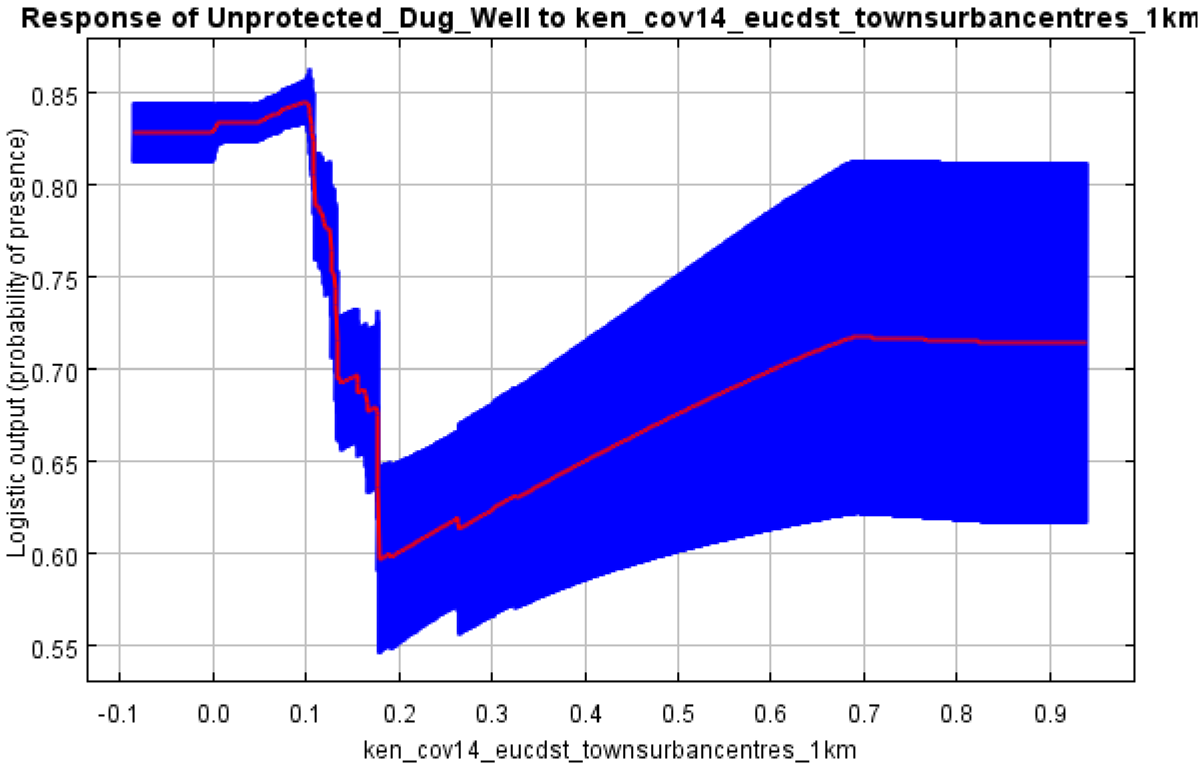

S1_Fig.27. Response curve of Euclidean distance to towns/urban centres presented as means (red) of 50 replicate runs with standard deviation in blue; model built with other predictive covariates being kept at their average sample values. X-axis: Euclidean distance (decimal degrees).

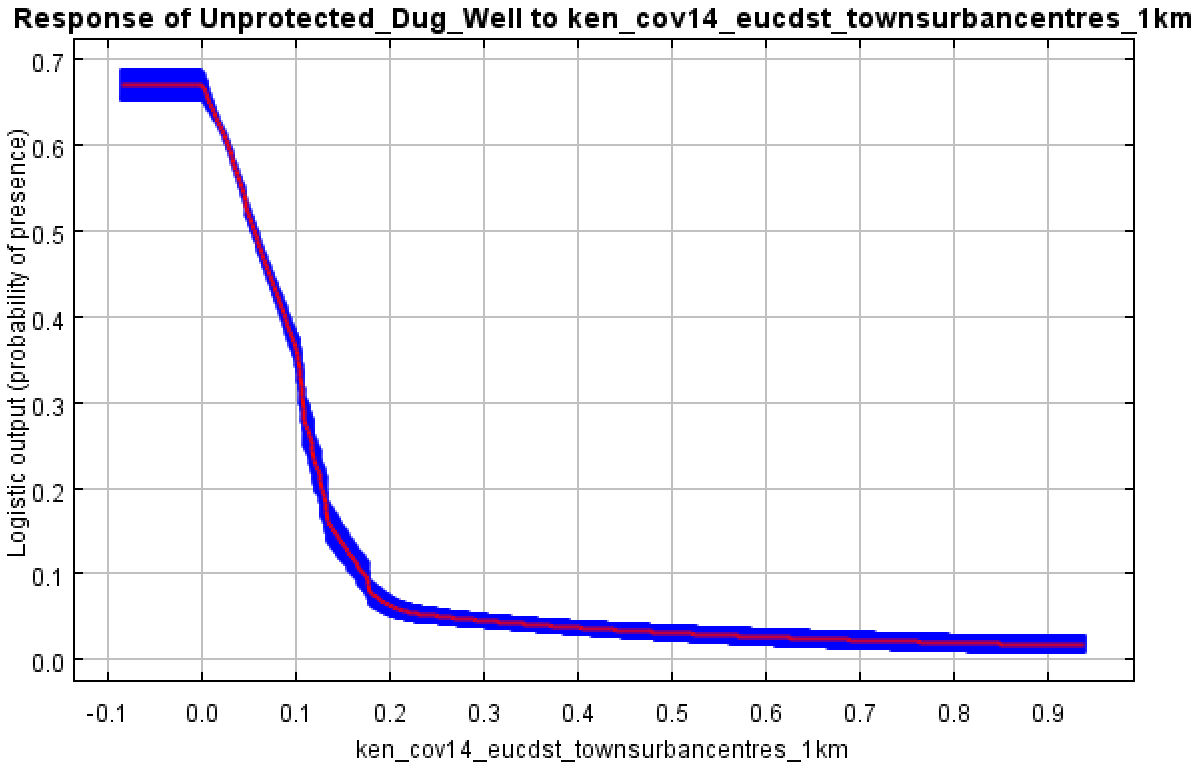

S1_Fig.28. Response curve of Euclidean distance to towns/urban centres presented as means (red) of 50 replicate runs with standard deviation in blue; model built without other predictive covariates. X-axis: Euclidean distance (decimal degrees).

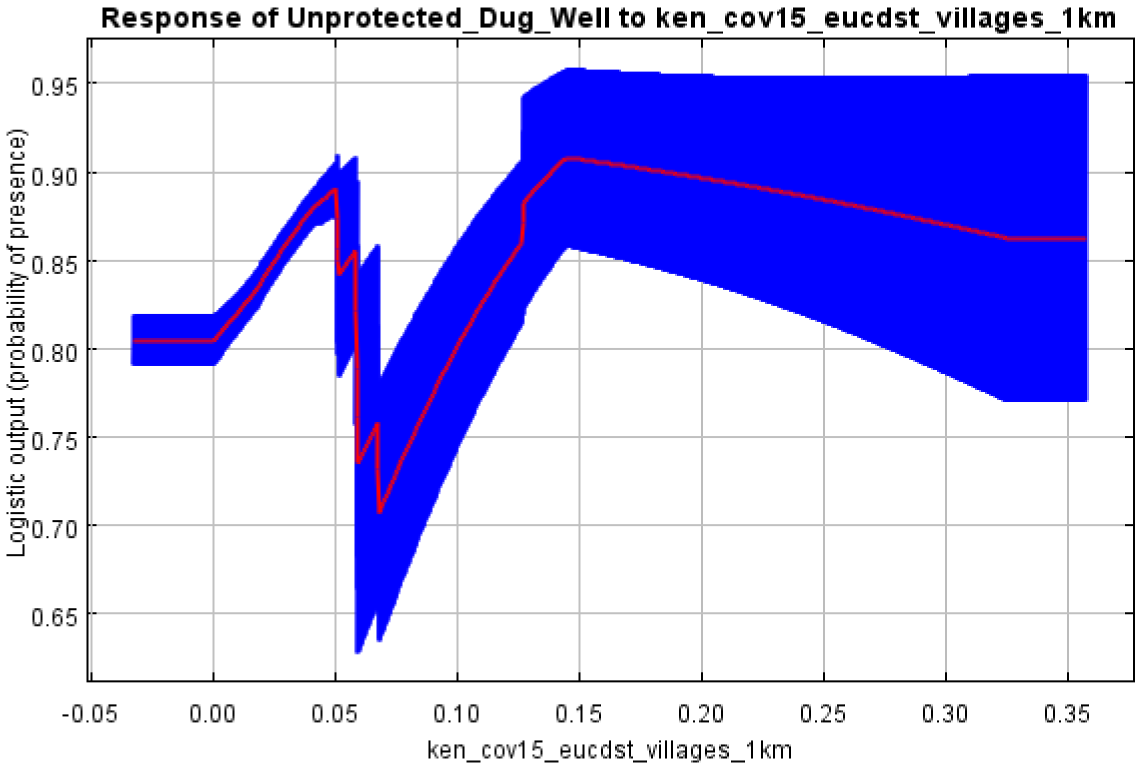

S1_Fig.29. Response curve of Euclidean distance to villages presented as means (red) of 50 replicate runs with standard deviation in blue; model built with other predictive covariates being kept at their average sample values. X-axis: Euclidean distance (decimal degrees).

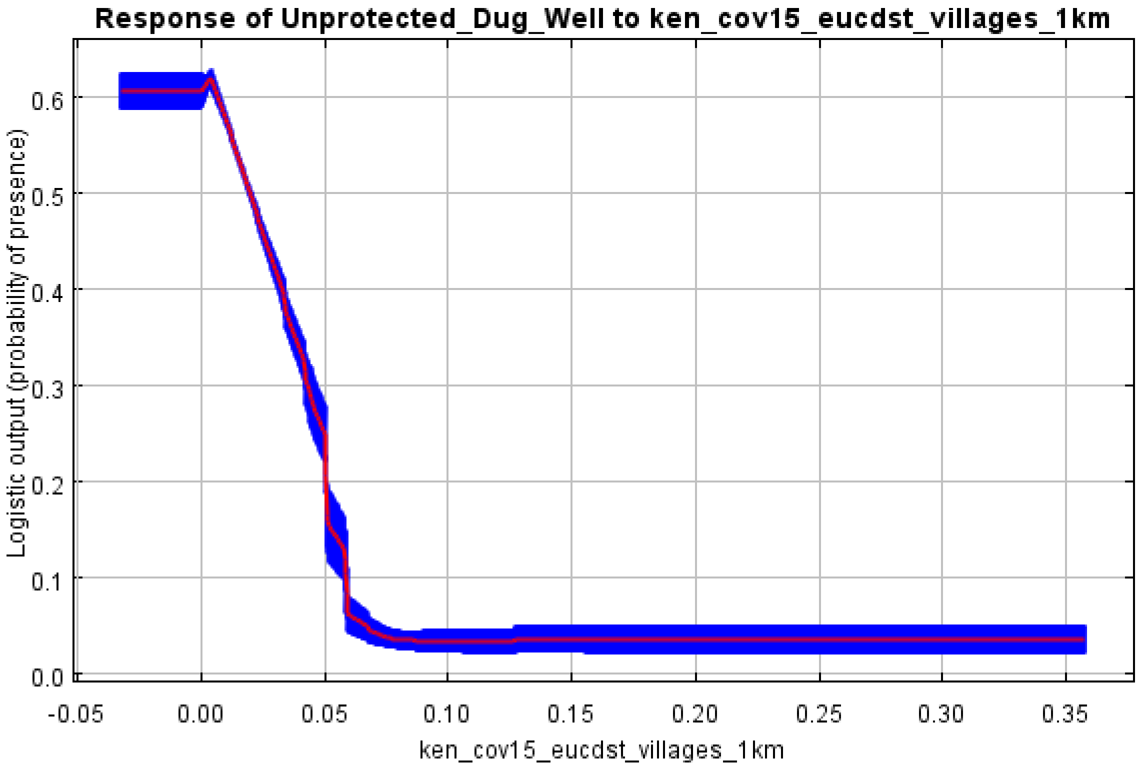

S1_Fig.30. Response curve of Euclidean distance to villages presented as means (red) of 50 replicate runs with standard deviation in blue; model built without other predictive covariates. X-axis: Euclidean distance (decimal degrees).

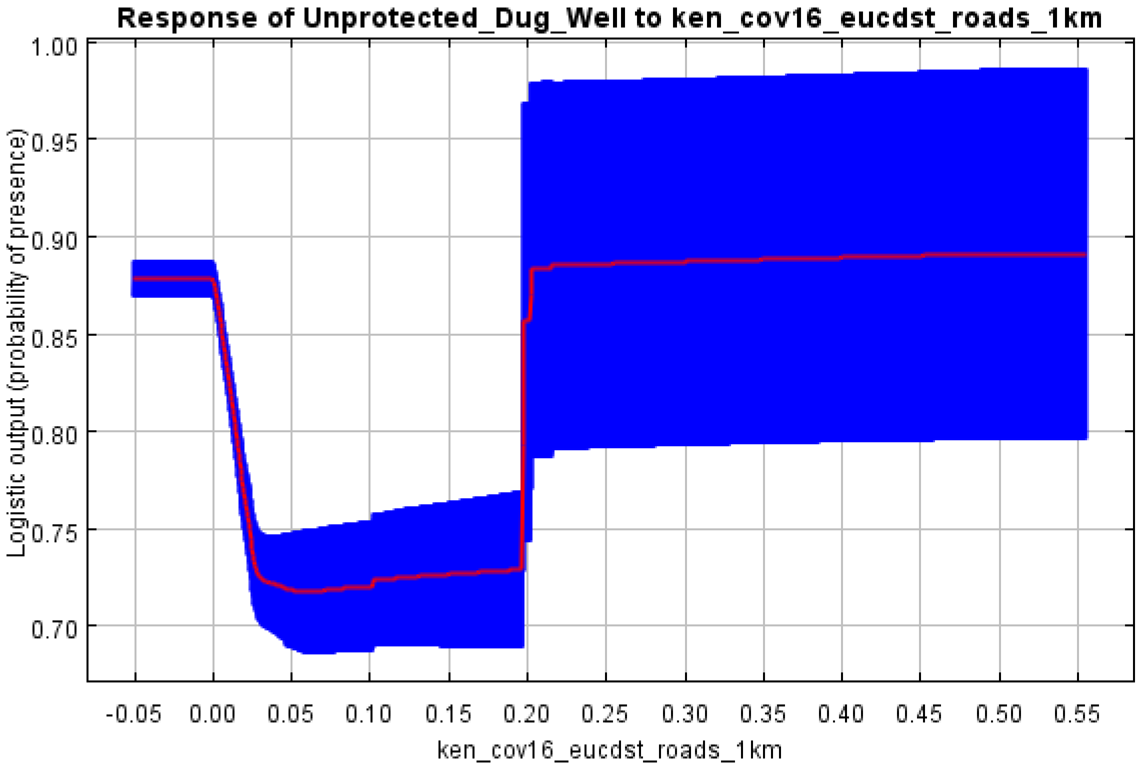

S1_Fig.31. Response curve of Euclidean distance to roads presented as means (red) of 50 replicate runs with standard deviation in blue; model built with other predictive covariates being kept at their average sample values. X-axis: Euclidean distance (decimal degrees).

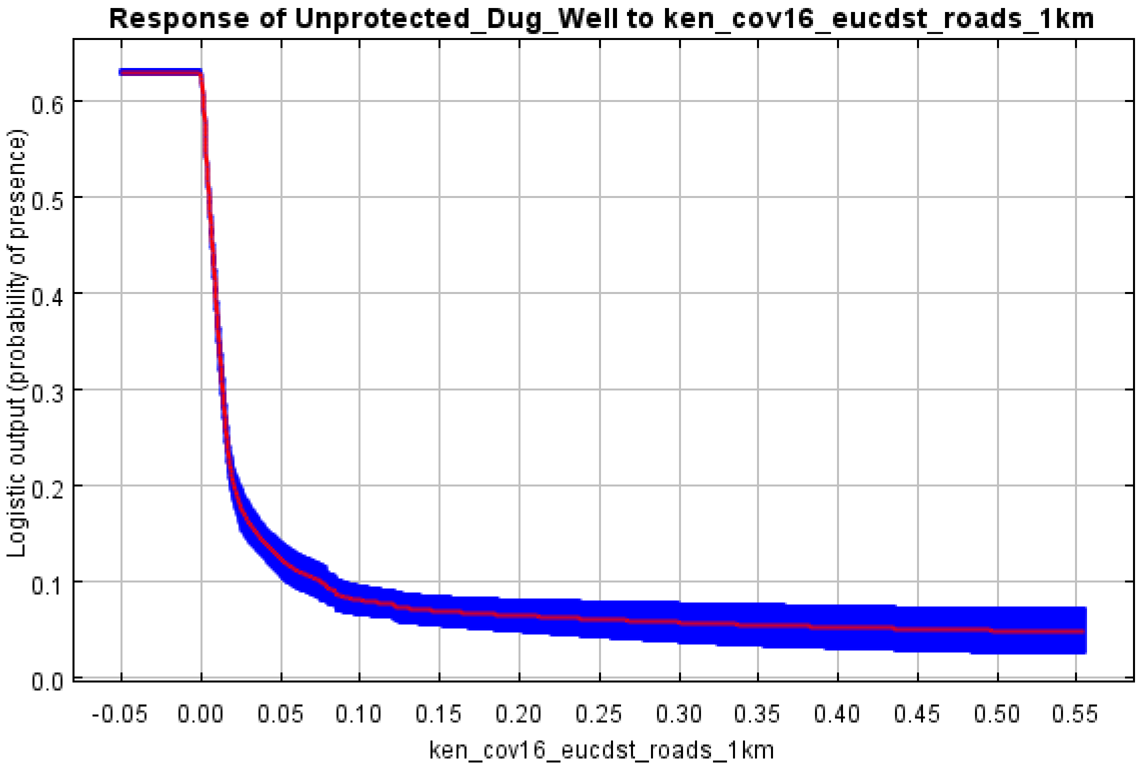

S1_Fig.32. Response curve of Euclidean distance to roads presented as means (red) of 50 replicate runs with standard deviation in blue; model built without other predictive covariates. X-axis: Euclidean distance (decimal degrees).

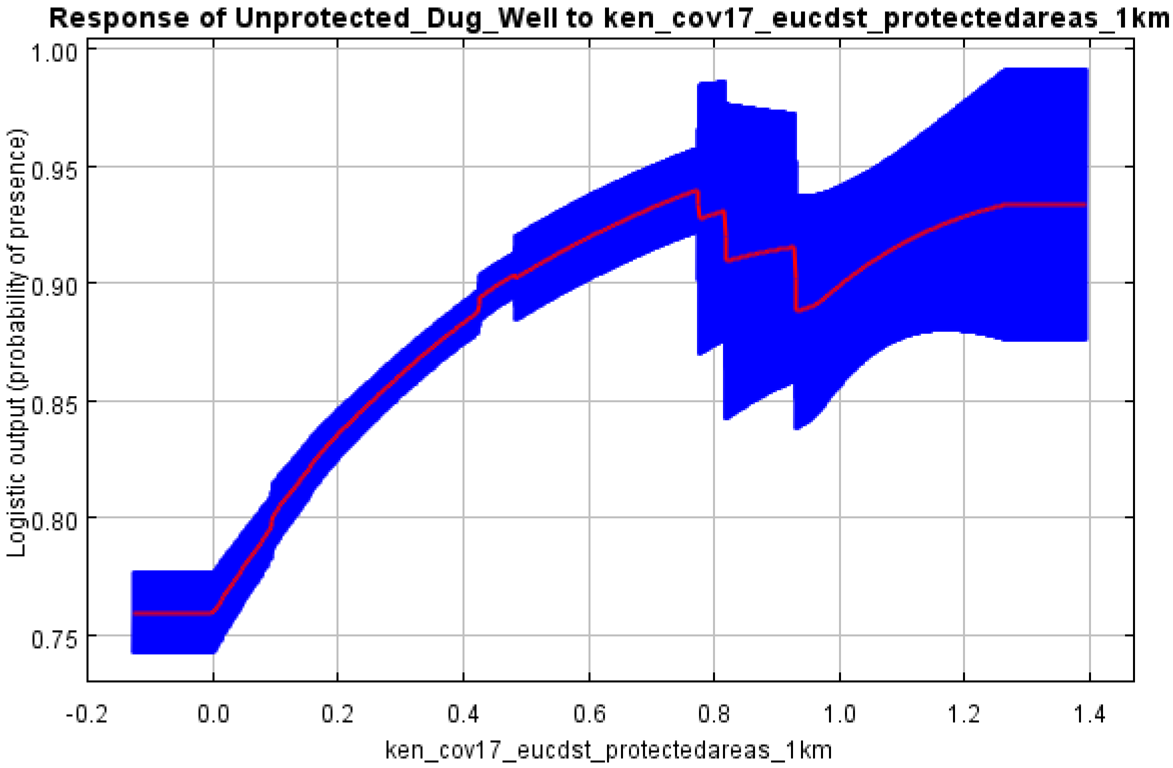

S1_Fig.33. Response curve of Euclidean distance to protected areas presented as means (red) of 50 replicate runs with standard deviation in blue; model built with other predictive covariates being kept at their average sample values. X-axis: Euclidean distance (decimal degrees).

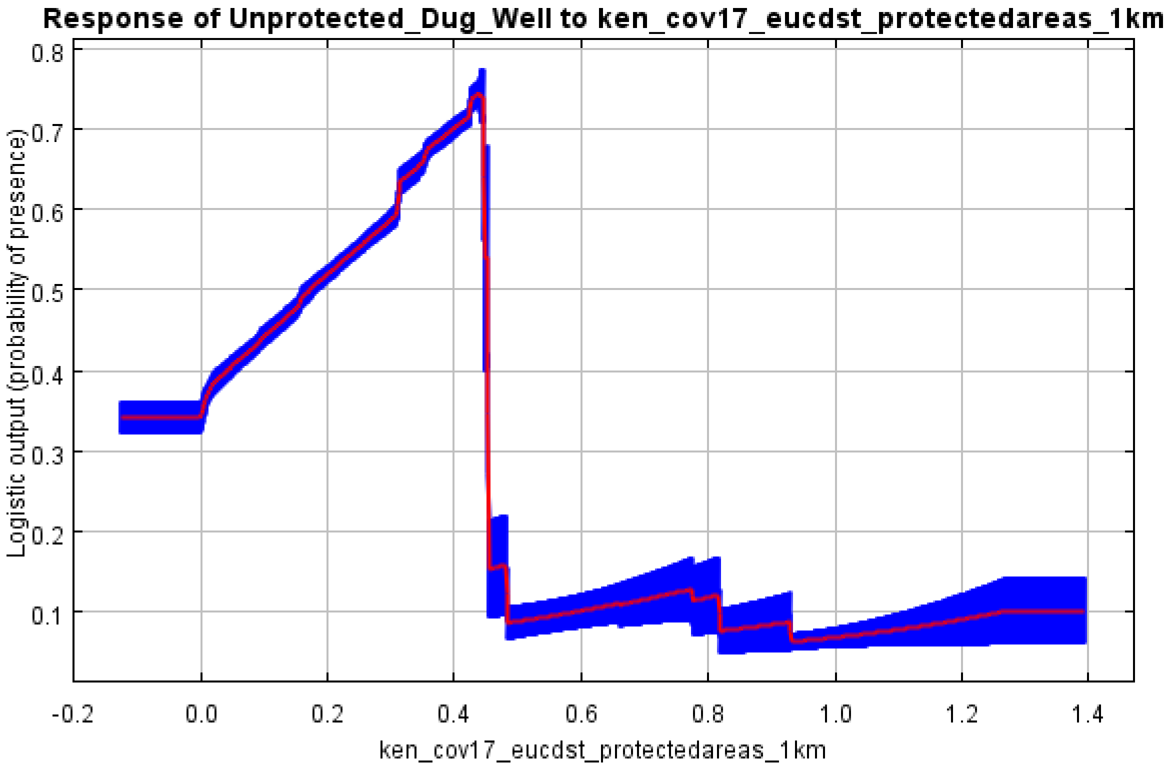

S1_Fig.34. Response curve of Euclidean distance to protected areas presented as means (red) of 50 replicate runs with standard deviation in blue; model built without other predictive covariates. X-axis: Euclidean distance (decimal degrees).

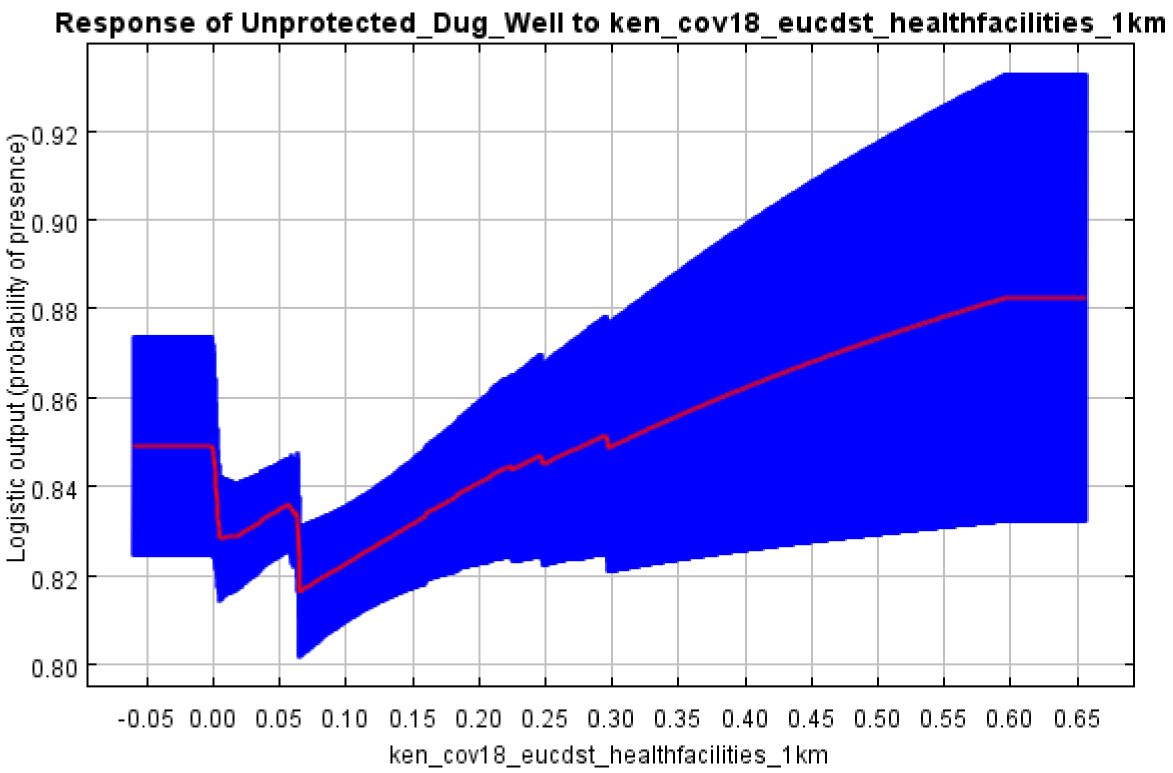

S1_Fig.35. Response curve of Euclidean distance to healthcare facilities presented as means (red) of 50 replicate runs with standard deviation in blue; model built with other predictive covariates being kept at their average sample values. X-axis: Euclidean distance (decimal degrees).

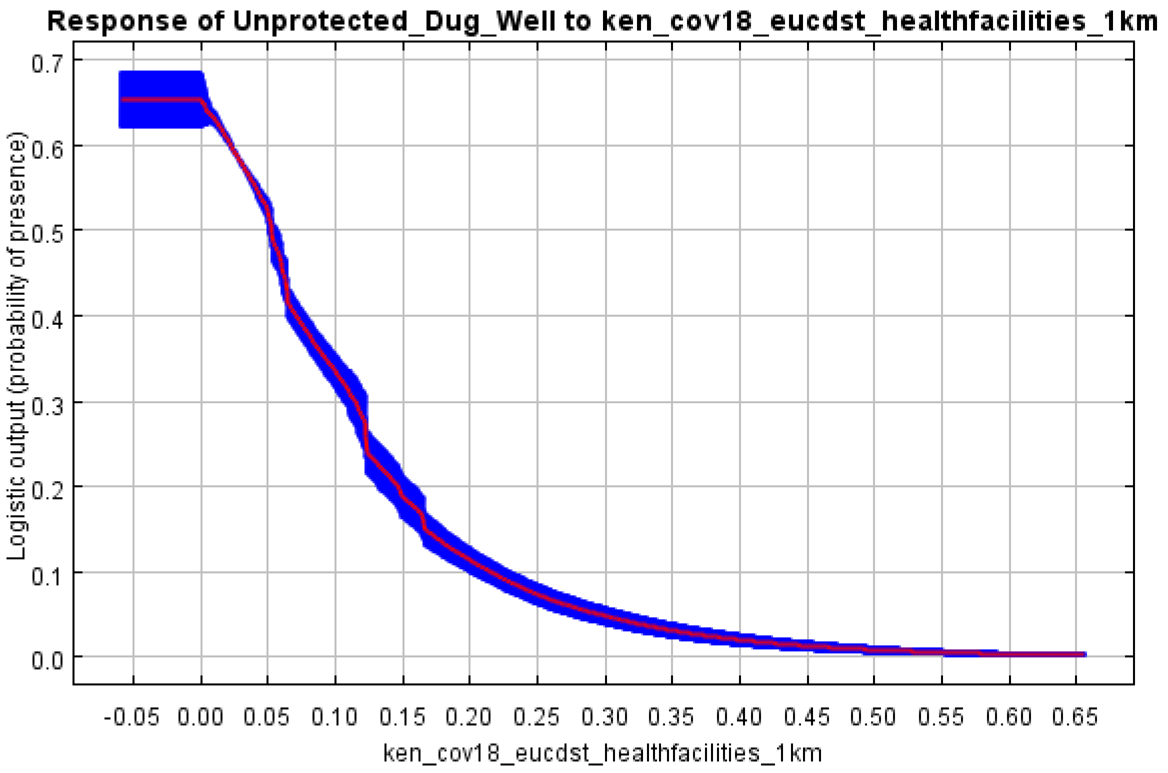

S1_Fig.36. Response curve of Euclidean distance to healthcare facilities presented as means (red) of 50 replicate runs with standard deviation in blue; model built without other predictive covariates. X-axis: Euclidean distance (decimal degrees).

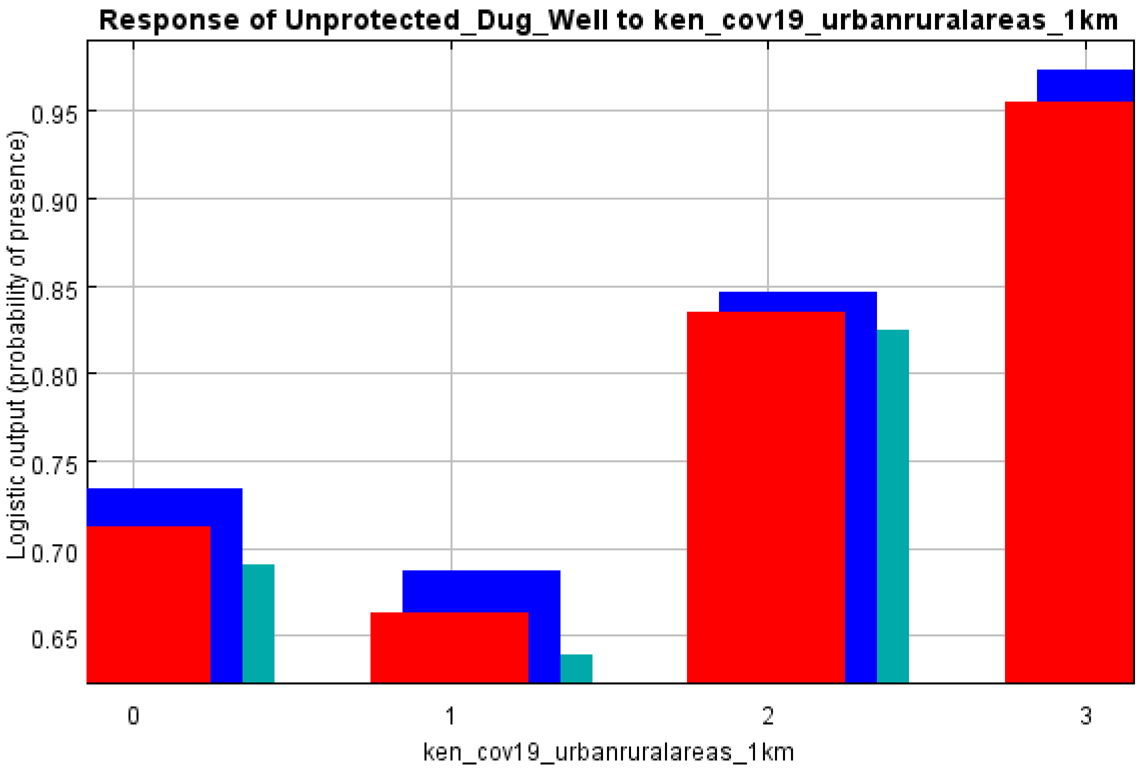

S1_Fig.37. Response curve of rural/urban areas presented as means (red) of 50 replicate runs with standard deviation in blue; model built with other predictive covariates being kept at their average sample values. X-axis: rurality: 0 – Others (not populated/no data); 1 – Rural areas; 2 – Urban clusters; 3 – Urban centres.

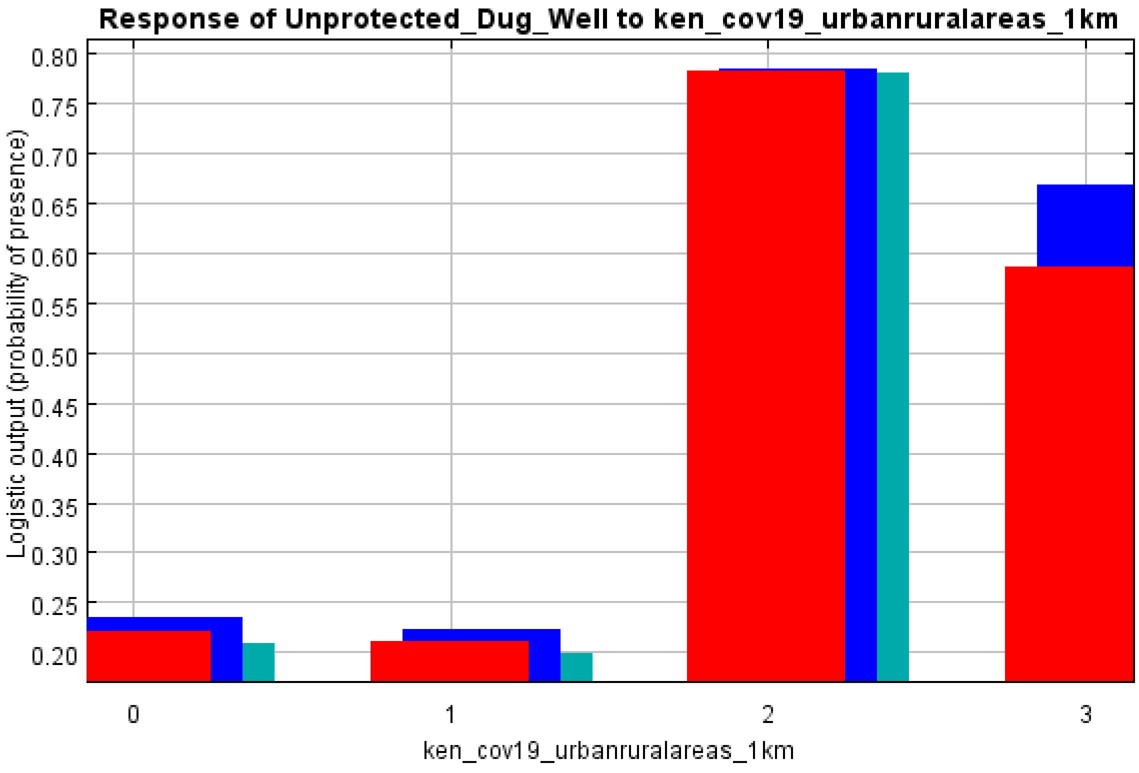

S1_Fig.38. Response curve of rural/urban areas presented as means (red) of 50 replicate runs with standard deviation in blue; model built without other predictive covariates. X-axis: rurality: 0 – Others (not populated/no data); 1 – Rural areas; 2 – Urban clusters; 3 – Urban centres.

Unprotected dug wells (bias file)

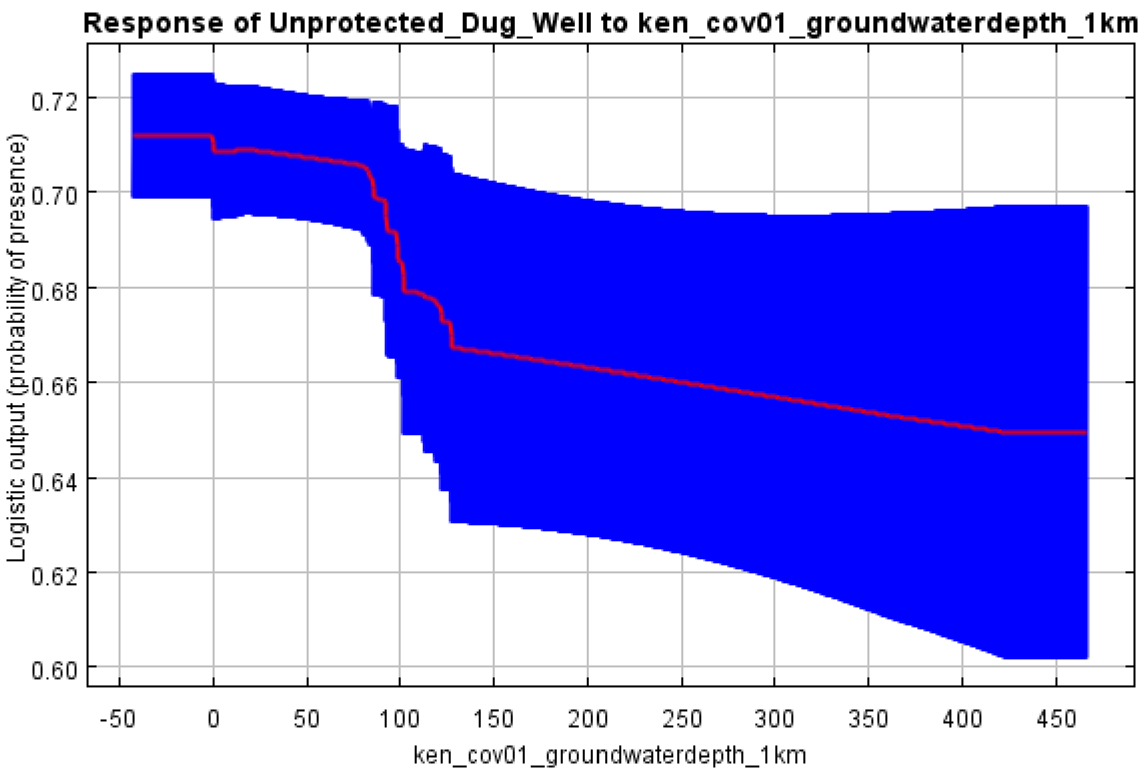

S1_Fig.39. Response curve of groundwater depth presented as means (red) of 50 replicate runs with standard deviation in blue; model built with other predictive covariates being kept at their average sample values. X-axis: depth to groundwater table (m).

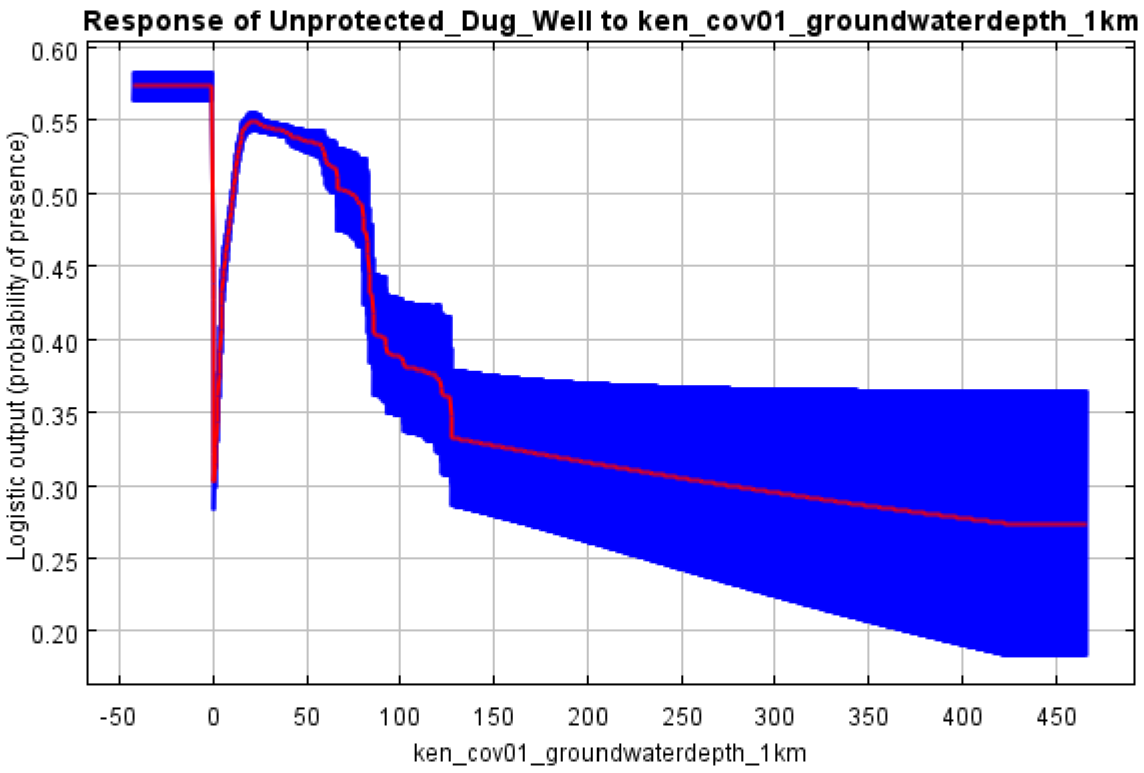

S1_Fig.40. Response curve of groundwater depth presented as means (red) of 50 replicate runs with standard deviation in blue; model built without other predictive covariates. X-axis: depth to groundwater table (m).

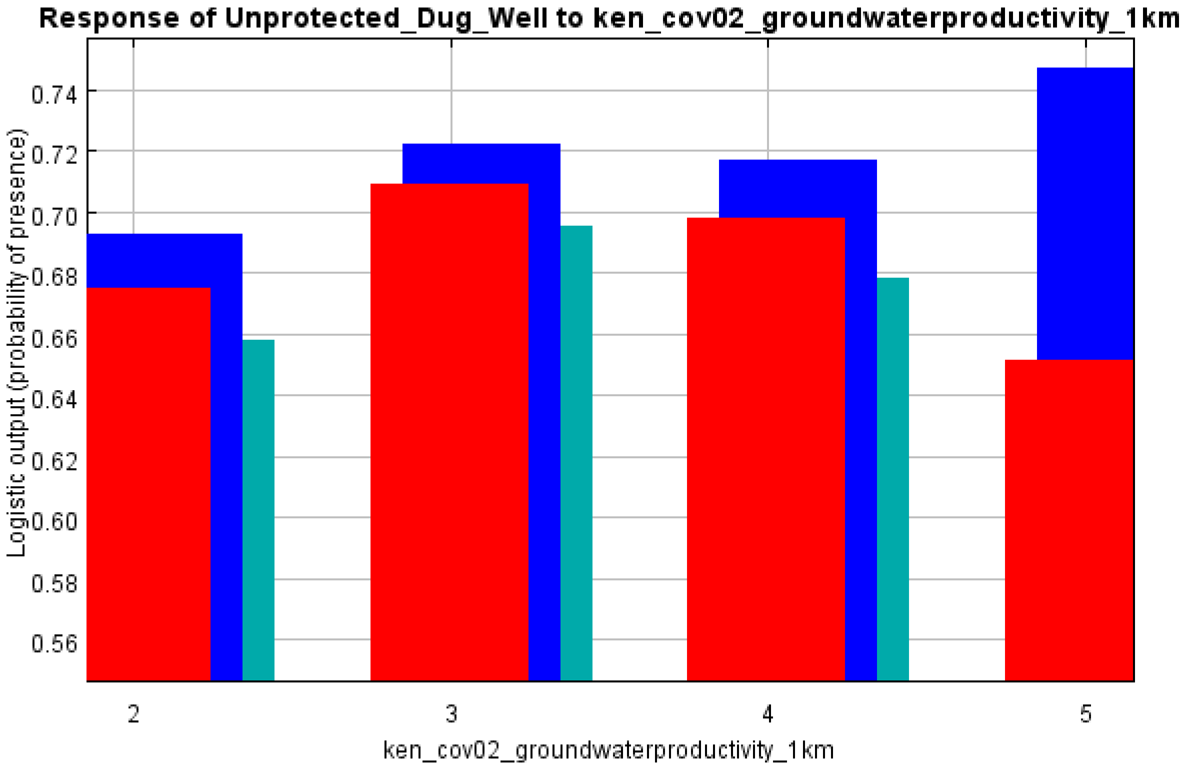

S1_Fig.41. Response curve of groundwater productivity presented as means (red) of 50 replicate runs with standard deviation in blue; model built with other predictive covariates being kept at their average sample values. X-axis: productivity: 2 – Low (0.1-0.5 l/s); 3 – Low-Moderate (0.5-1 l/s); 4 – Moderate (1-5 l/s); 5 – High (5-20 l/s).

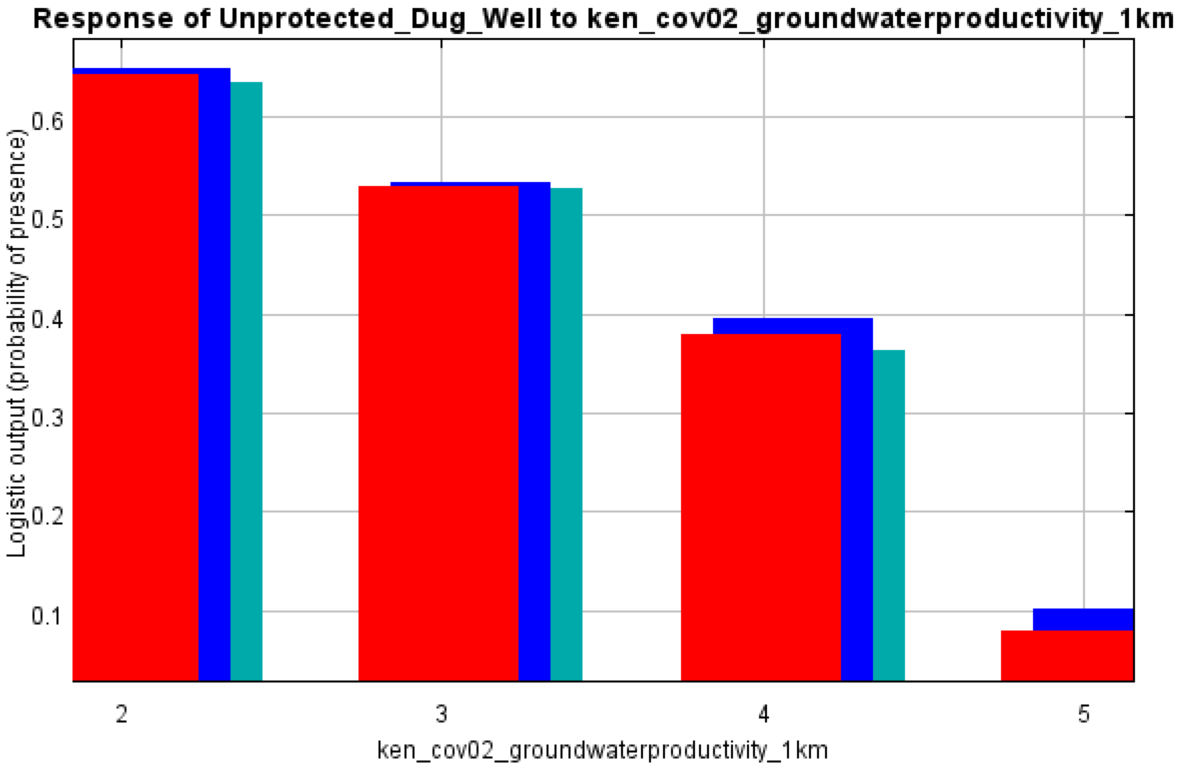

S1_Fig.42. Response curve of groundwater productivity presented as means (red) of 50 replicate runs with standard deviation in blue; model built without other predictive covariates. X-axis: productivity: 2 – Low (0.1-0.5 l/s); 3 – Low-Moderate (0.5-1 l/s); 4 – Moderate (1-5 l/s); 5 – High (5-20 l/s).

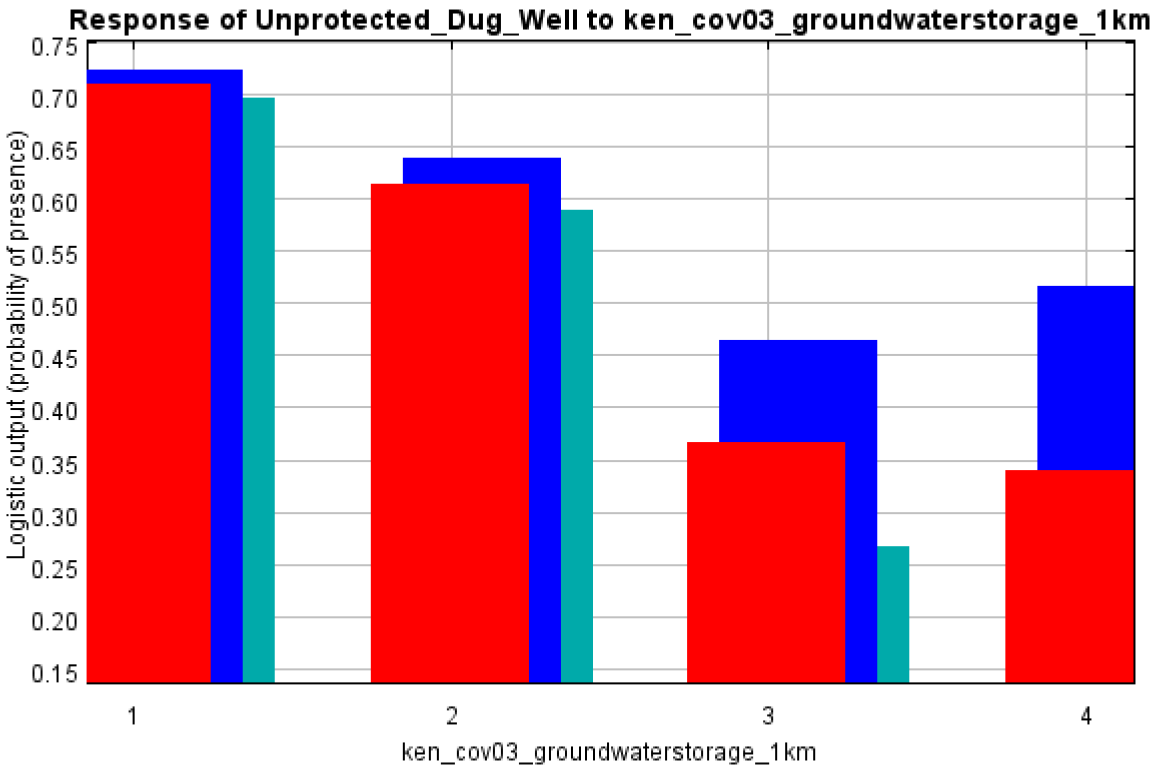

S1_Fig.43. Response curve of groundwater storage presented as means (red) of 50 replicate runs with standard deviation in blue; model built with other predictive covariates being kept at their average sample values. X-axis: storage (water depth in mm): 1 – Low (<1000); 2 – Low-Moderate (1000-10,000); 3 – Moderate (10,000-25,000); 4 – High (25,000-50,000).

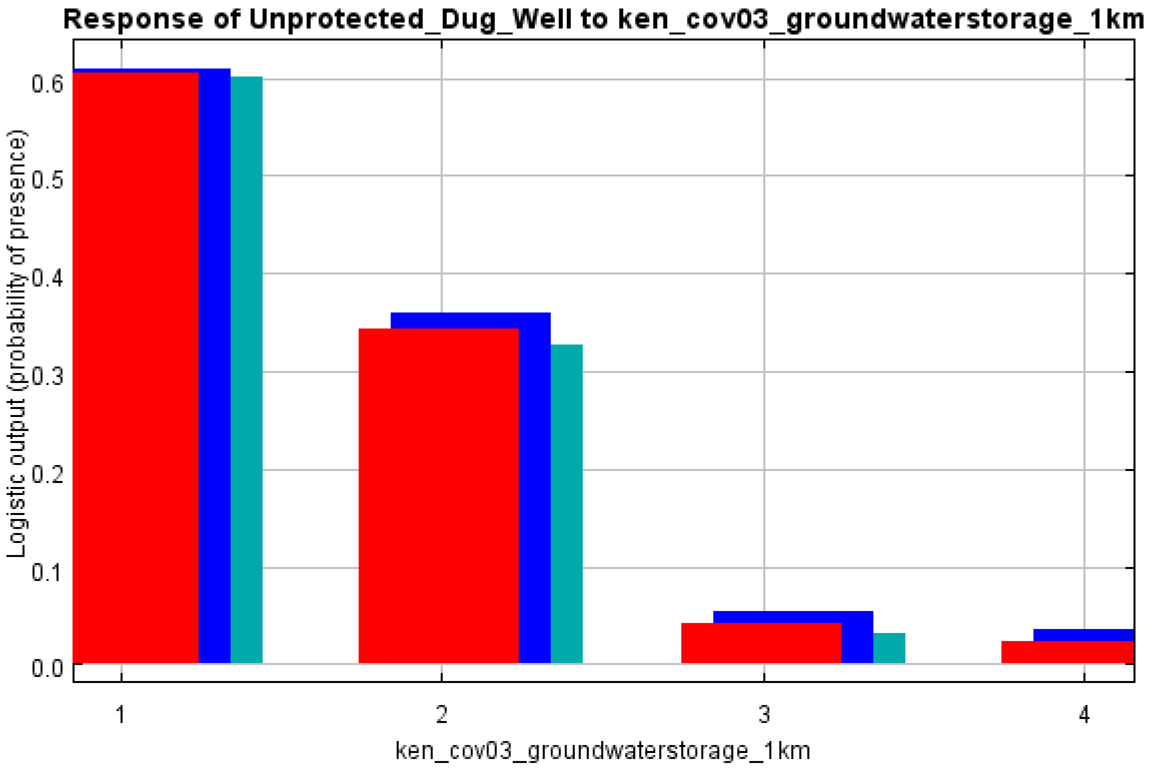

S1_Fig.44. Response curve of groundwater storage presented as means (red) of 50 replicate runs with standard deviation in blue; model built without other predictive covariates. X-axis: storage (water depth in mm): 1 – Low (<1000); 2 – Low-Moderate (1000-10,000); 3 – Moderate (10,000-25,000); 4 – High (25,000-50,000).

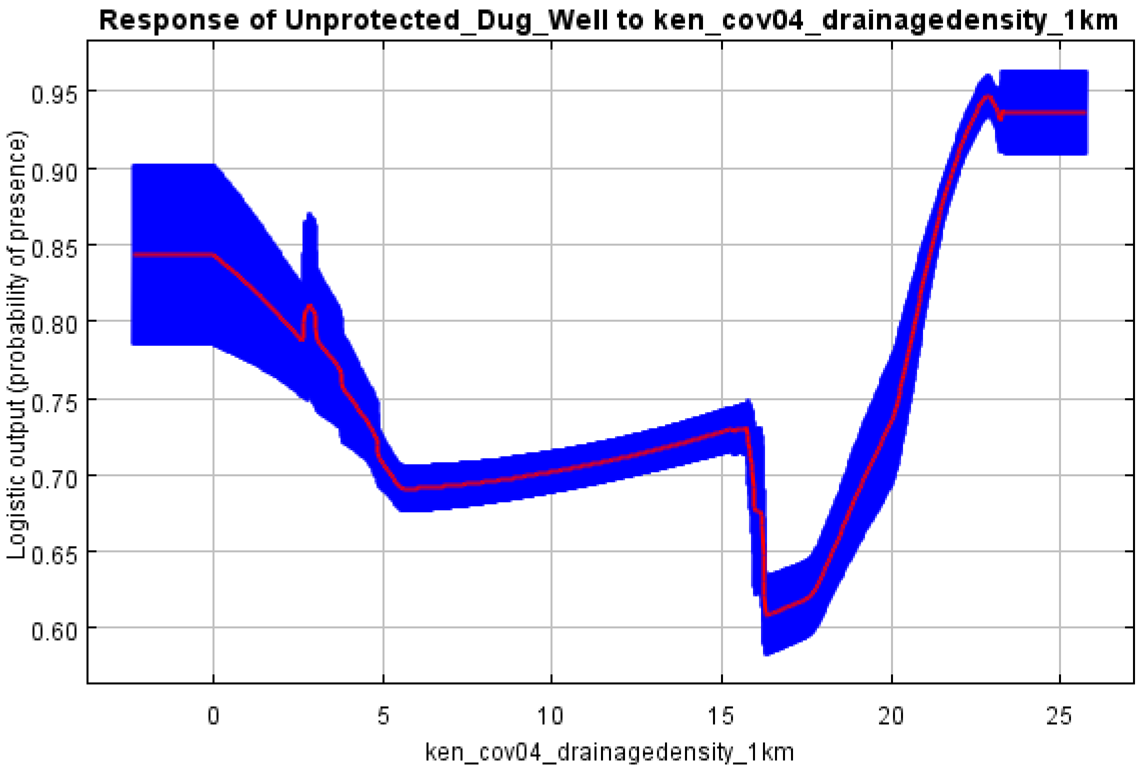

S1_Fig.45. Response curve of drainage density presented as means (red) of 50 replicate runs with standard deviation in blue; model built with other predictive covariates being kept at their average sample values. X-axis: drainage density – channel length per area size of a grid cell (lengths in decimal degrees).

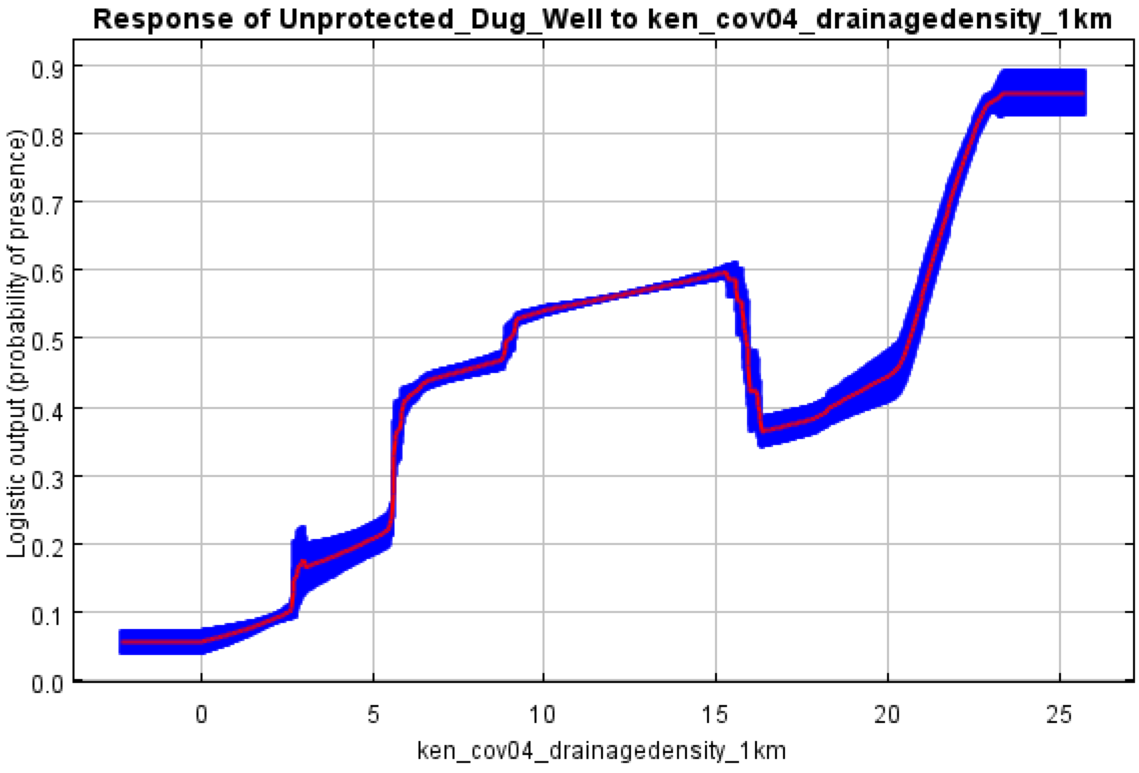

S1_Fig.46. Response curve of drainage density presented as means (red) of 50 replicate runs with standard deviation in blue; model built without other predictive covariates. X-axis: drainage density – channel length per area size of a grid cell (lengths in decimal degrees).

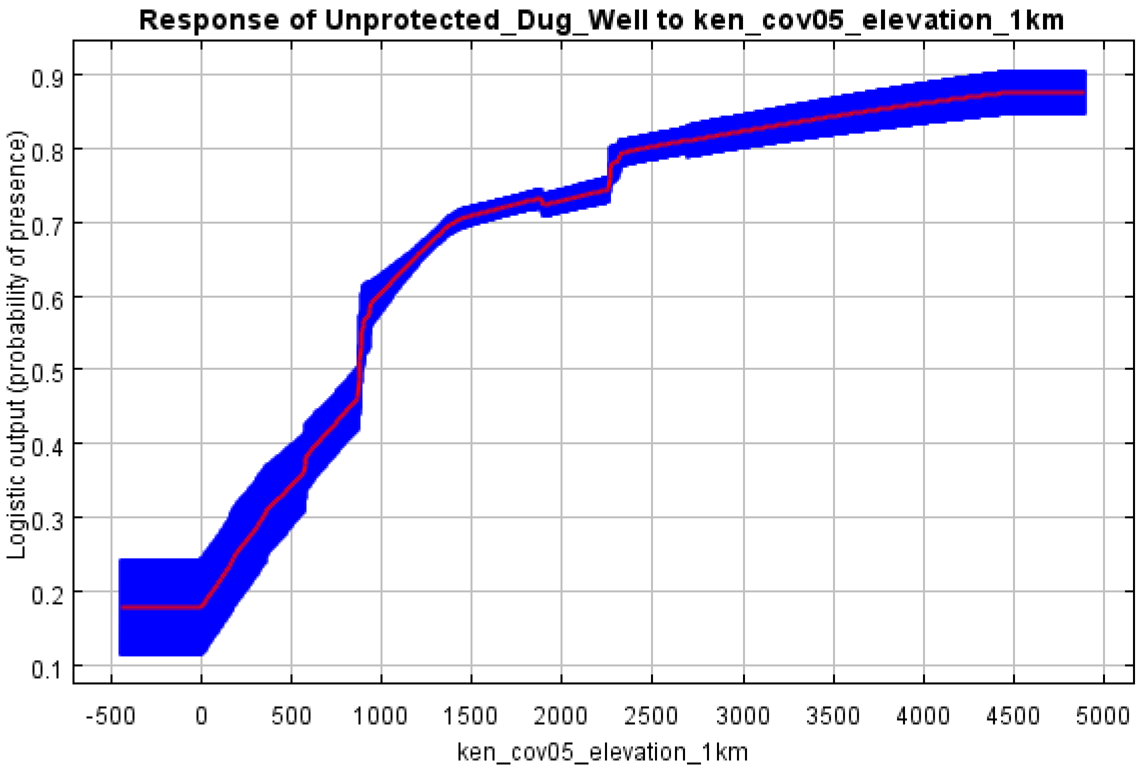

S1_Fig.47. Response curve of elevation presented as means (red) of 50 replicate runs with standard deviation in blue; model built with other predictive covariates being kept at their average sample values. X-axis: elevation (m).

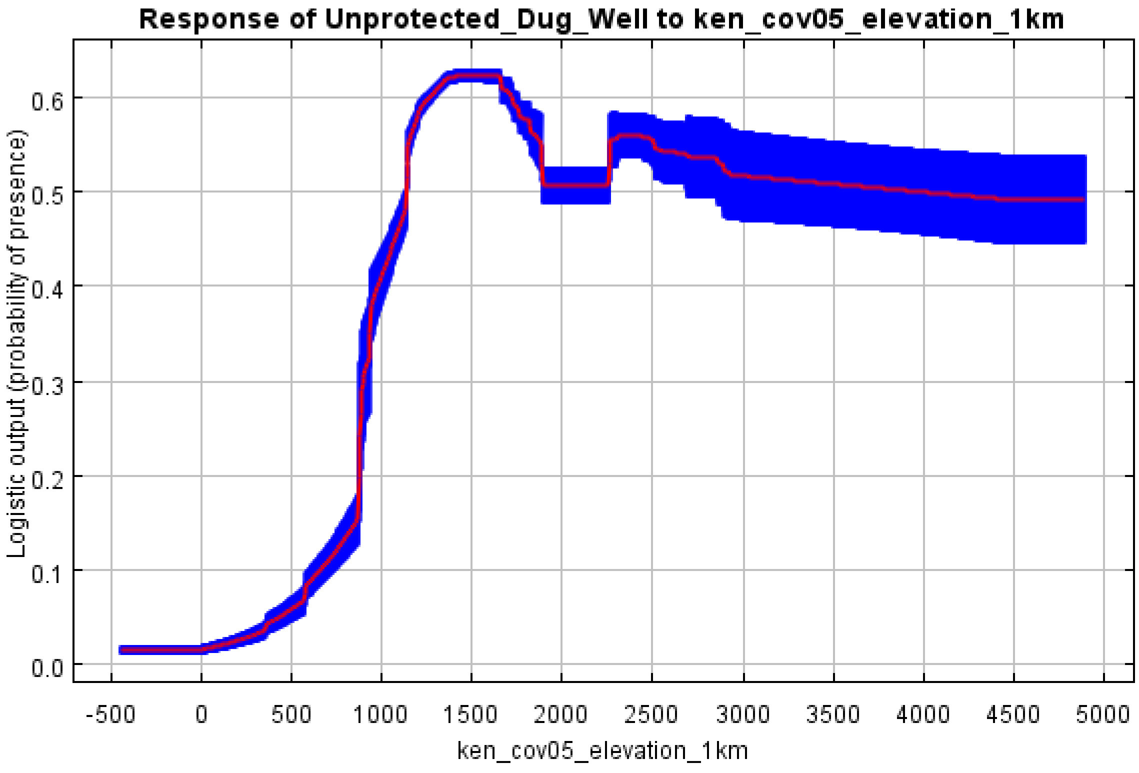

S1_Fig.48. Response curve of elevation presented as means (red) of 50 replicate runs with standard deviation in blue; model built without other predictive covariates. X-axis: elevation (m).

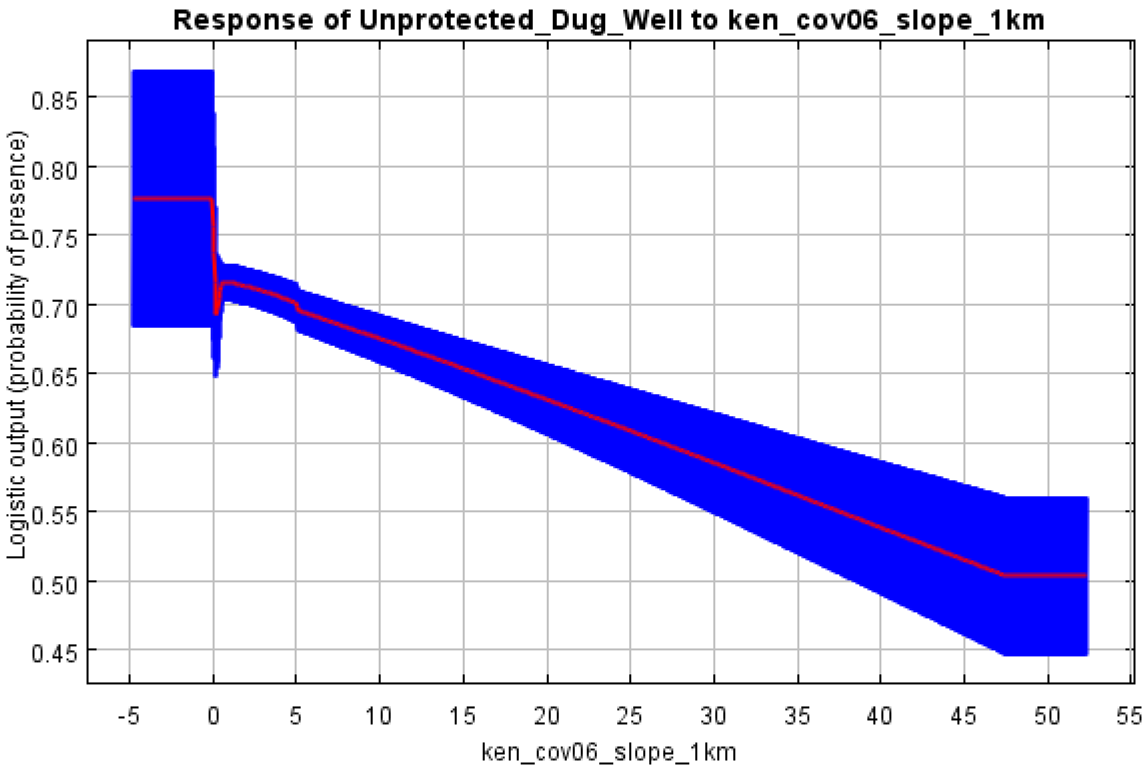

S1_Fig.49. Response curve of slope presented as means (red) of 50 replicate runs with standard deviation in blue; model built with other predictive covariates being kept at their average sample values. X-axis: slope (degree).

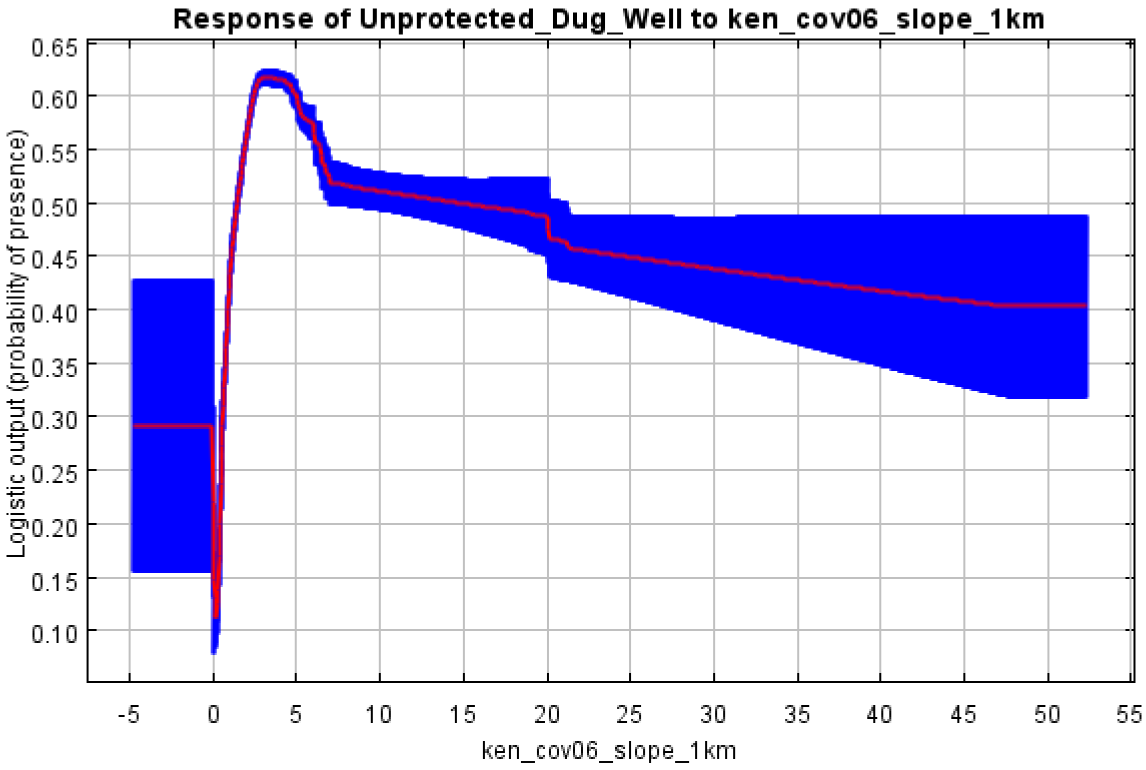

S1_Fig.50. Response curve of slope presented as means (red) of 50 replicate runs with standard deviation in blue; model built without other predictive covariates. X-axis: slope (degree).

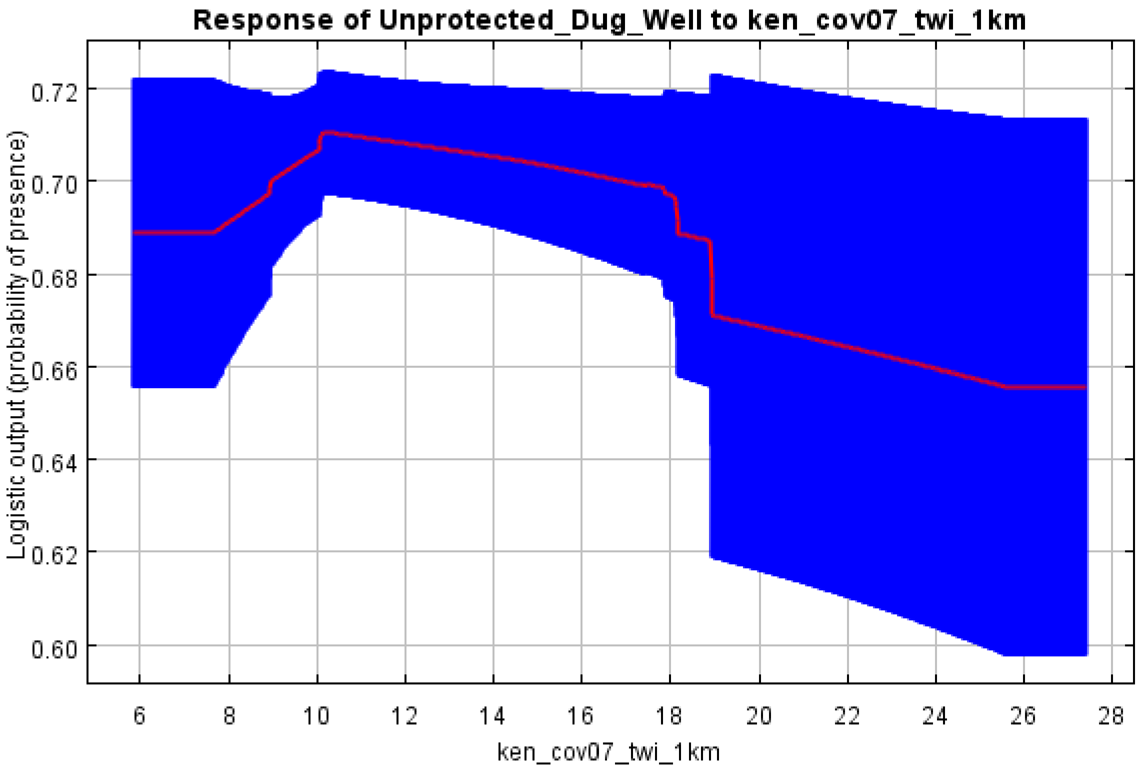

S1_Fig.51. Response curve of topographic wetness index presented as means (red) of 50 replicate runs with standard deviation in blue; model built with other predictive covariates being kept at their average sample values. X-axis: topographic unit index.

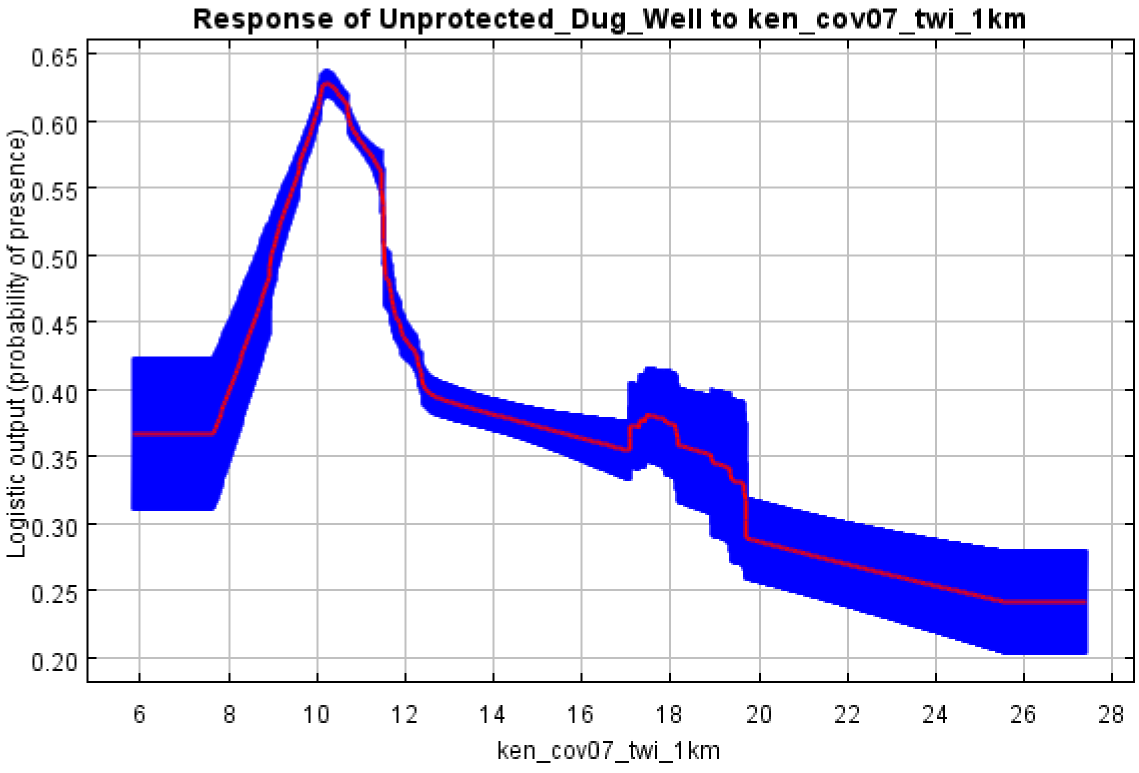

S1_Fig.52. Response curve of topographic wetness index presented as means (red) of 50 replicate runs with standard deviation in blue; model built without other predictive covariates. X-axis: topographic unit index.

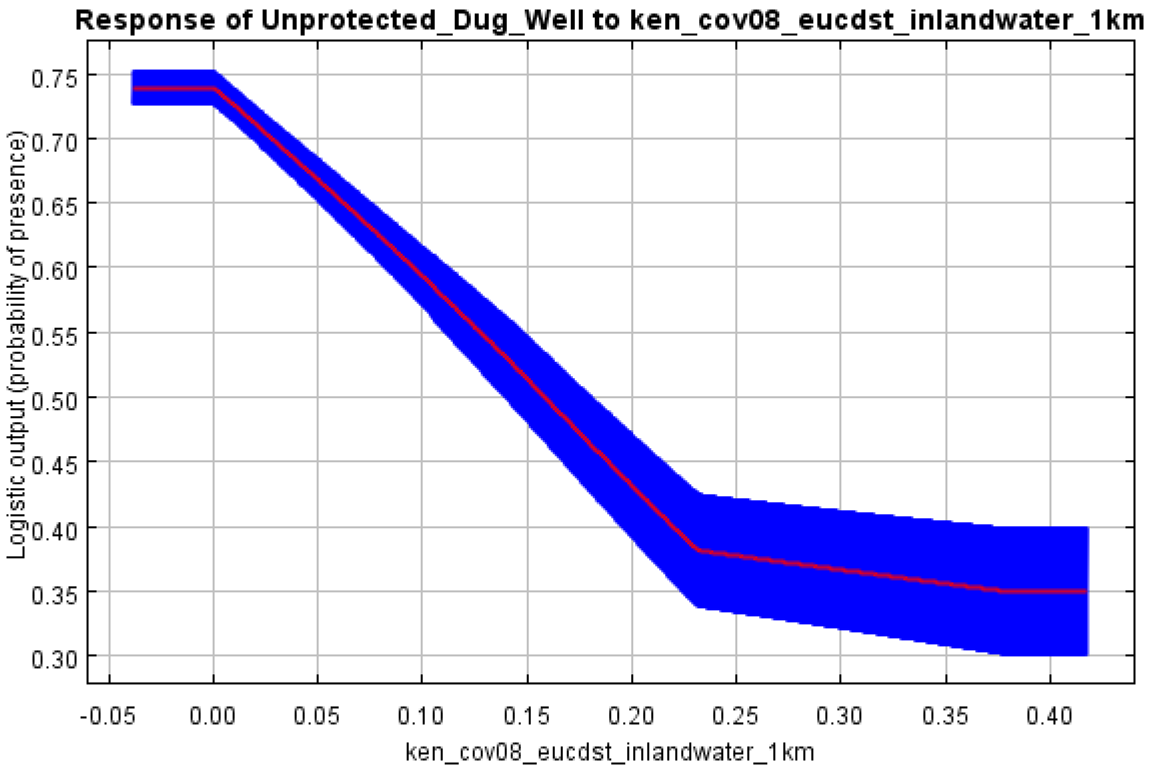

S1_Fig.53. Response curve of Euclidean distance to inland water presented as means (red) of 50 replicate runs with standard deviation in blue; model built with other predictive covariates being kept at their average sample values. X-axis: Euclidean distance (decimal degrees).

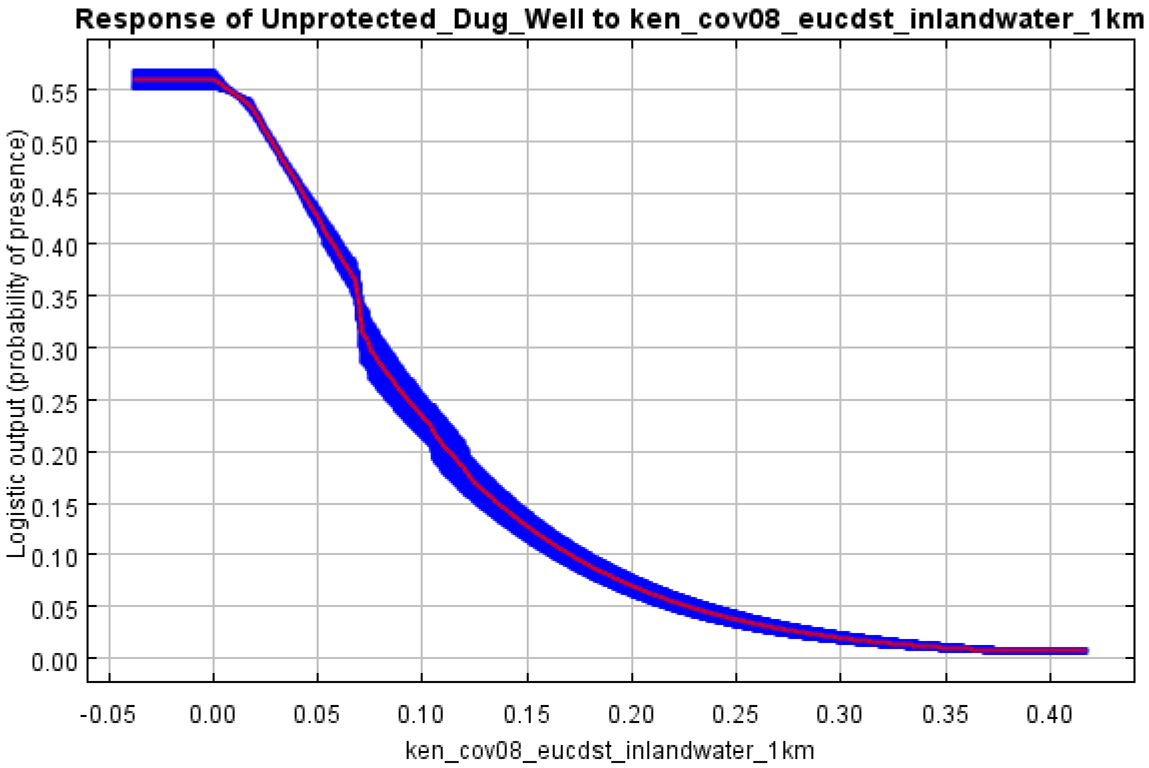

S1_Fig.54. Response curve of Euclidean distance to inland water presented as means (red) of 50 replicate runs with standard deviation in blue; model built without other predictive covariates. X-axis: Euclidean distance (decimal degrees).

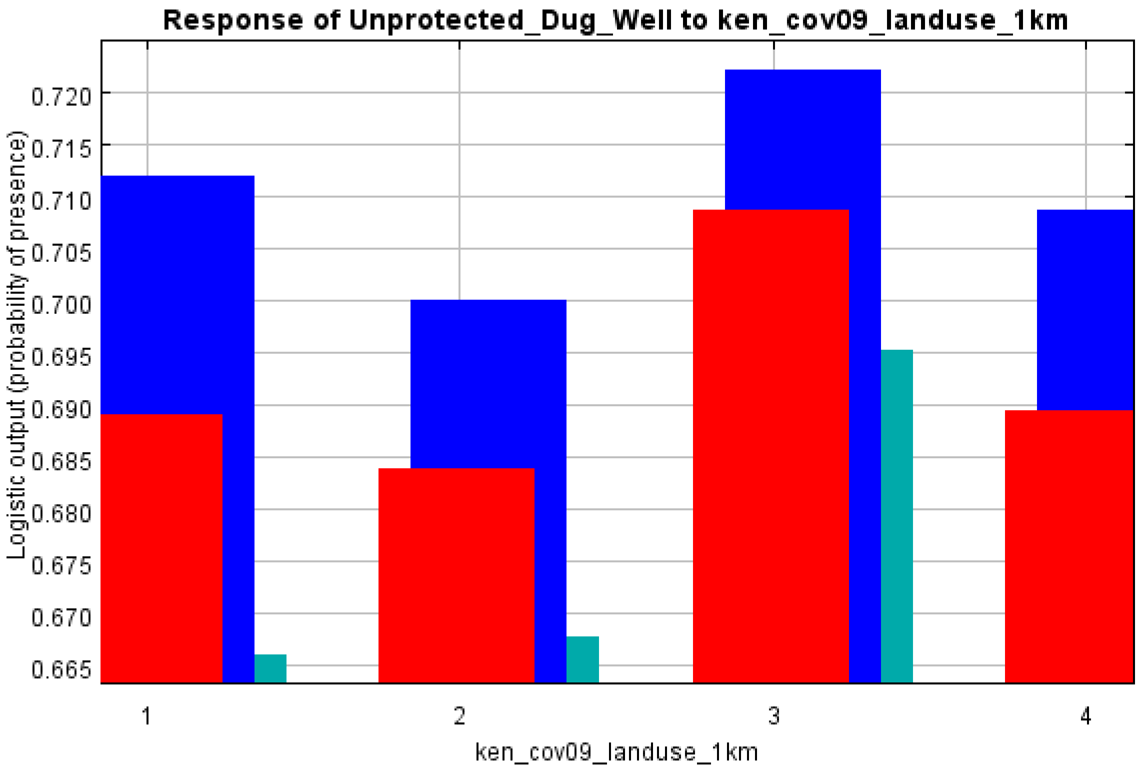

S1_Fig.55. Response curve of land use presented as means (red) of 50 replicate runs with standard deviation in blue; model built with other predictive covariates being kept at their average sample values. X-axis: land use: 1 – forest; 2 – others; 3 – agricultural land; 4 – urban area.

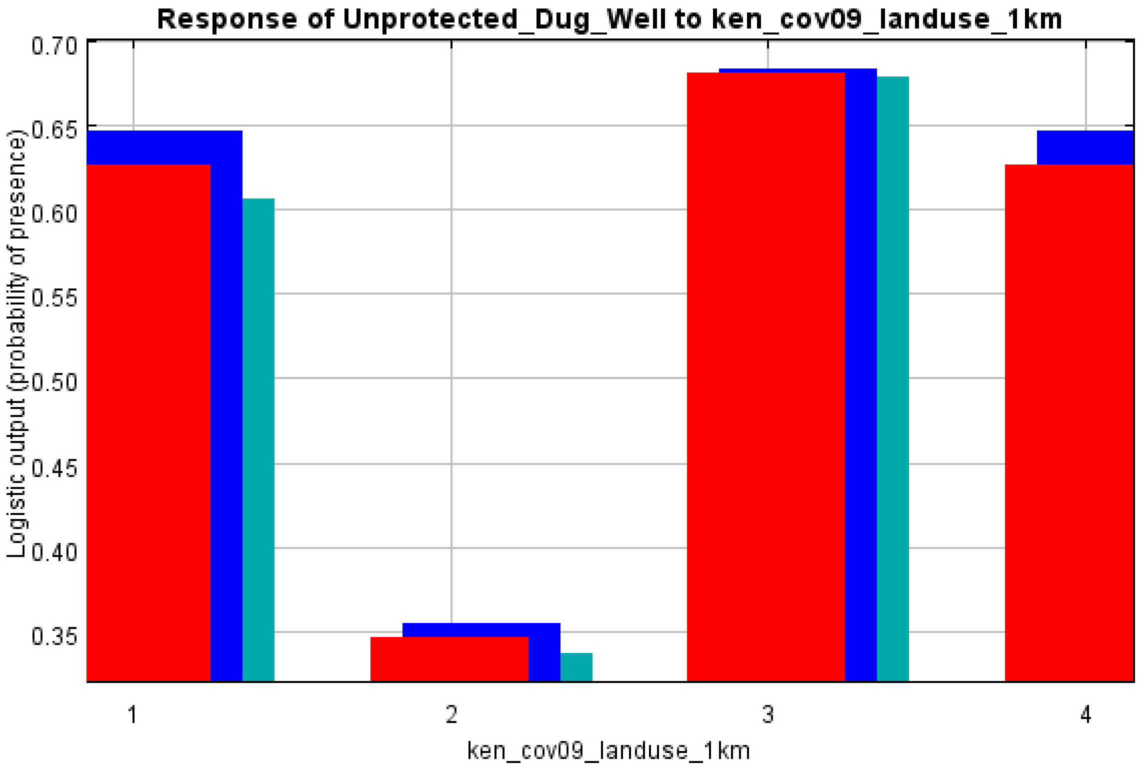

S1_Fig.56. Response curve of land use presented as means (red) of 50 replicate runs with standard deviation in blue; model built without other predictive covariates. X-axis: land use: 1 – forest; 2 – others; 3 – agricultural land; 4 – urban area.

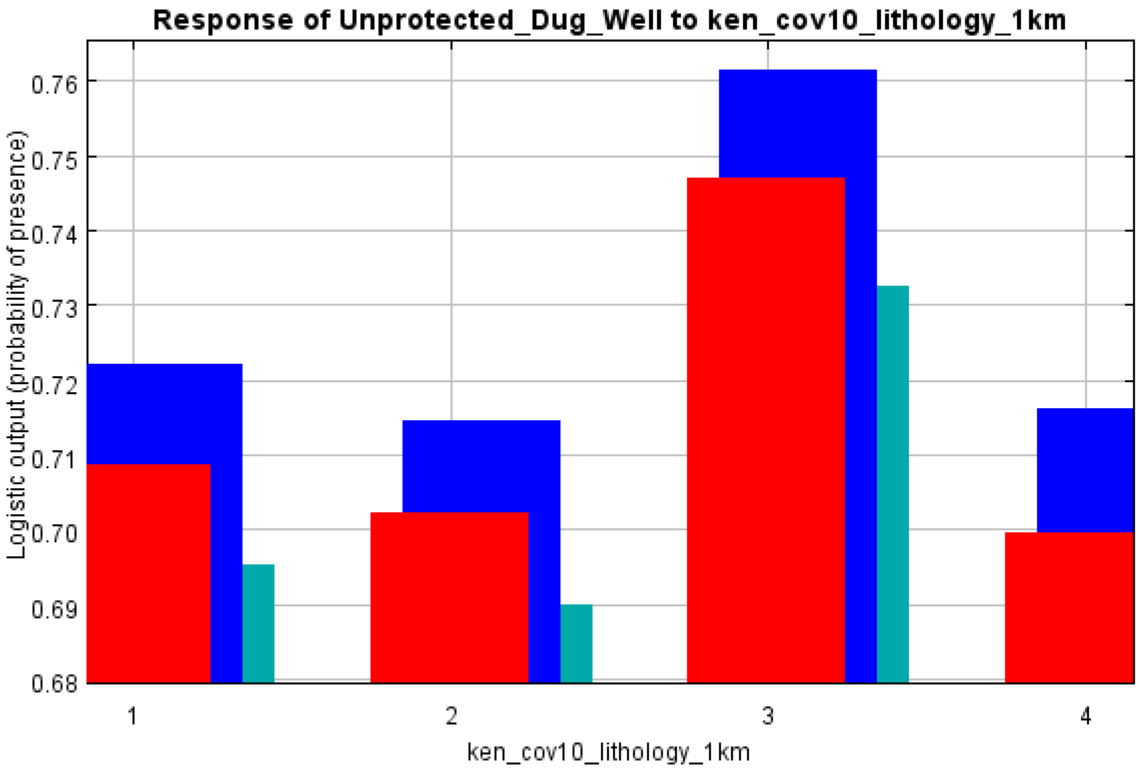

S1_Fig.57. Response curve of lithology presented as means (red) of 50 replicate runs with standard deviation in blue; model built with other predictive covariates being kept at their average sample values. X-axis: lithology: 1 – Igneous; 2 – Metamorphic rock; 3 – Sedimentary rock; 4 – Unconsolidated.

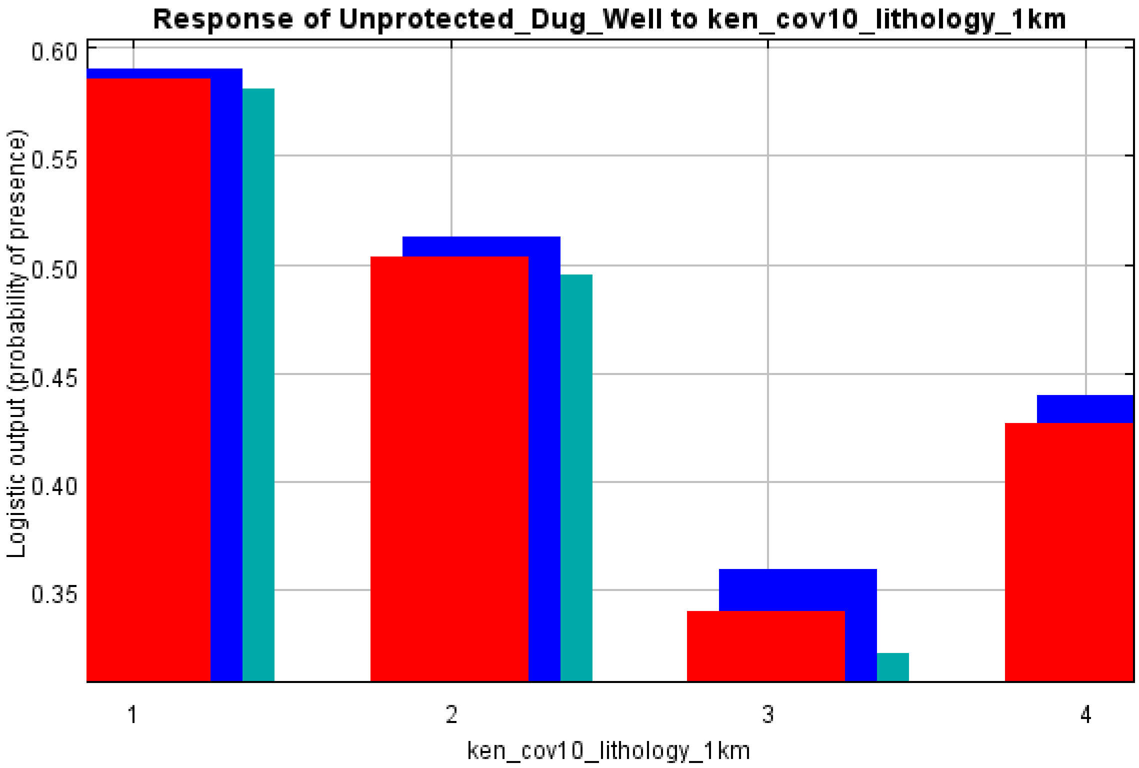

S1_Fig.58. Response curve of lithology presented as means (red) of 50 replicate runs with standard deviation in blue; model built without other predictive covariates. X-axis: lithology: 1 – Igneous; 2 – Metamorphic rock; 3 – Sedimentary rock; 4 – Unconsolidated.

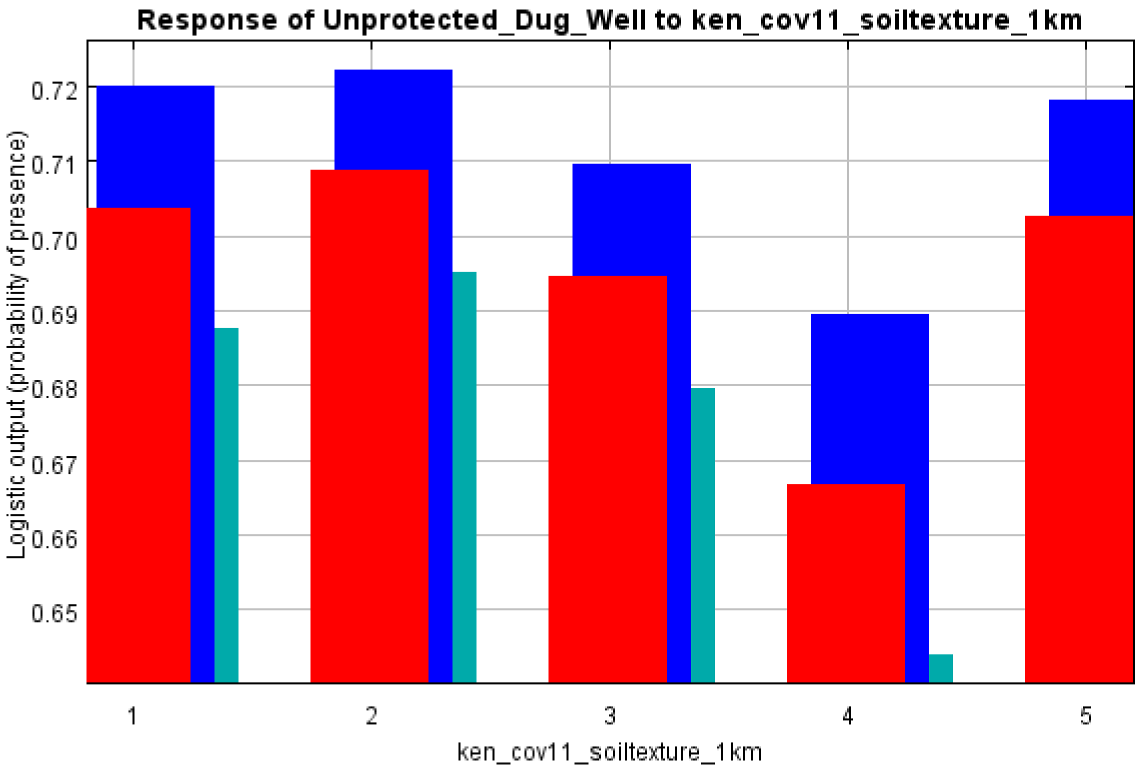

S1_Fig.59. Response curve of soil texture presented as means (red) of 50 replicate runs with standard deviation in blue; model built with other predictive covariates being kept at their average sample values. X-axis: lithology: 1 – Very clayey; 2 – Clayey; 3 – Loamy; 4 – Sandy; 5 – Extremely sandy.

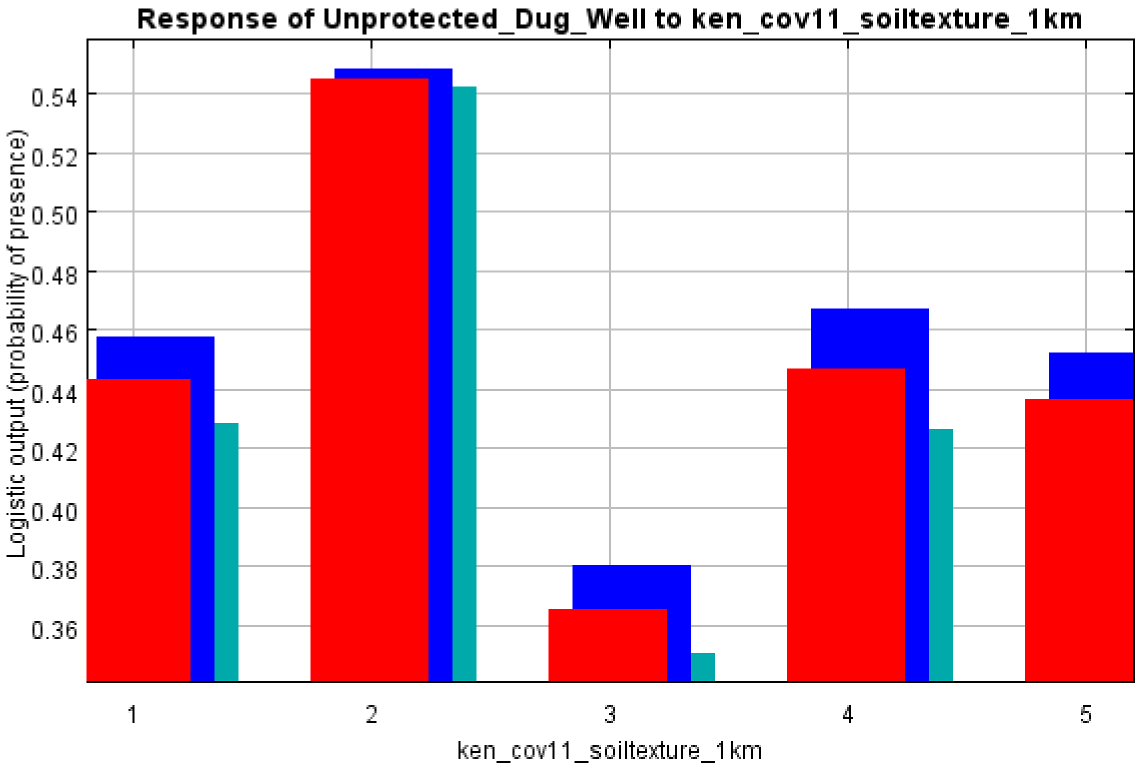

S1_Fig.60. Response curve of soil texture presented as means (red) of 50 replicate runs with standard deviation in blue; model built without other predictive covariates. X-axis: lithology: 1 – Very clayey; 2 – Clayey; 3 – Loamy; 4 – Sandy; 5 – Extremely sandy.

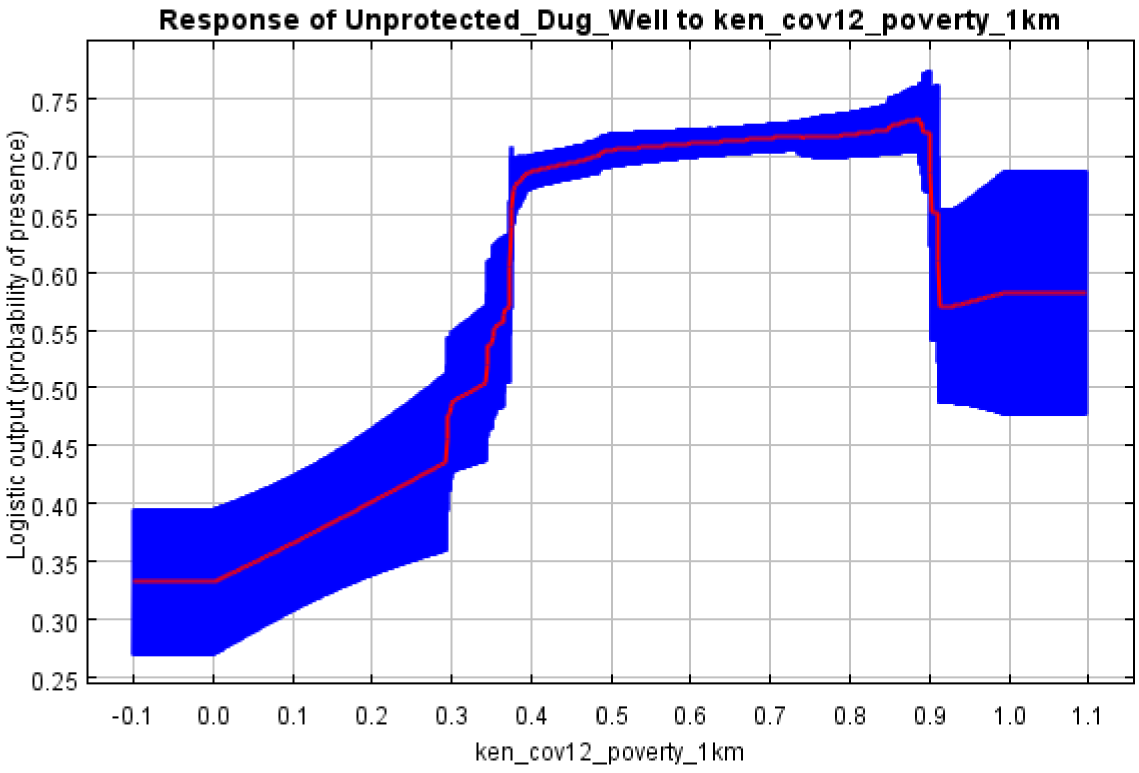

S1_Fig.61. Response curve of poverty presented as means (red) of 50 replicate runs with standard deviation in blue; model built with other predictive covariates being kept at their average sample values. X-axis: proportion of residents living in MPI-defined poverty.

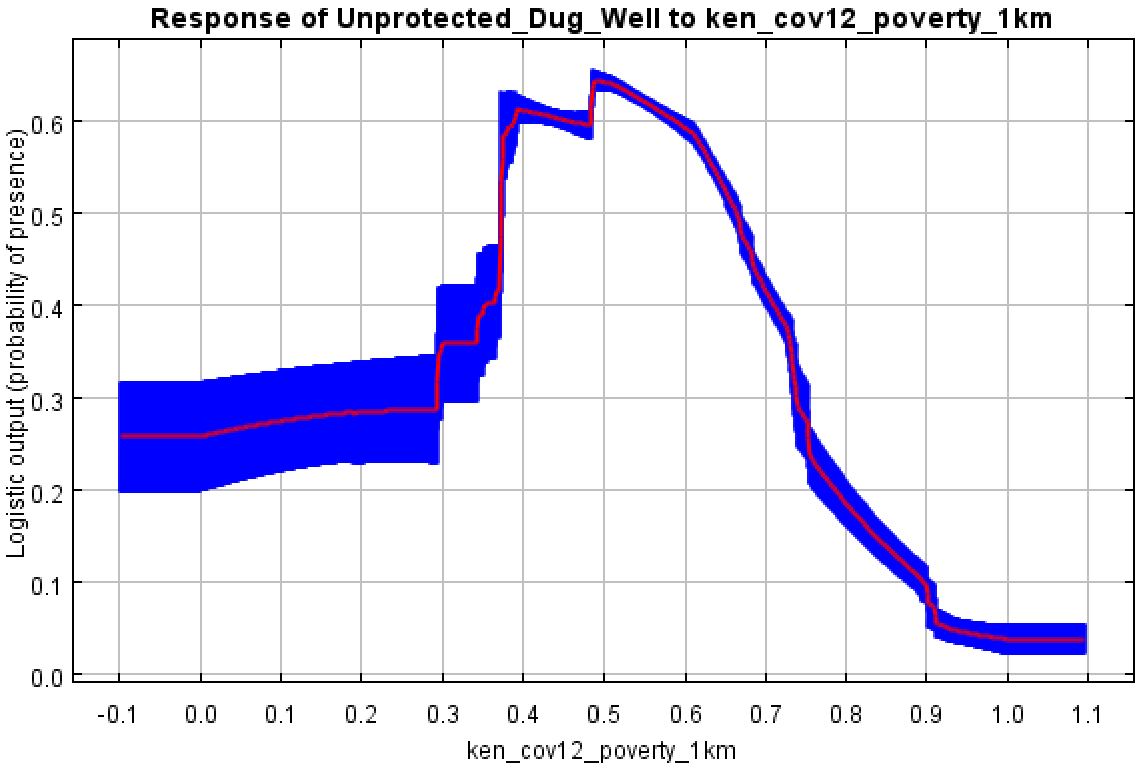

S1_Fig.62. Response curve of poverty presented as means (red) of 50 replicate runs with standard deviation in blue; model built without other predictive covariates. X-axis: proportion of residents living in MPI-defined poverty.

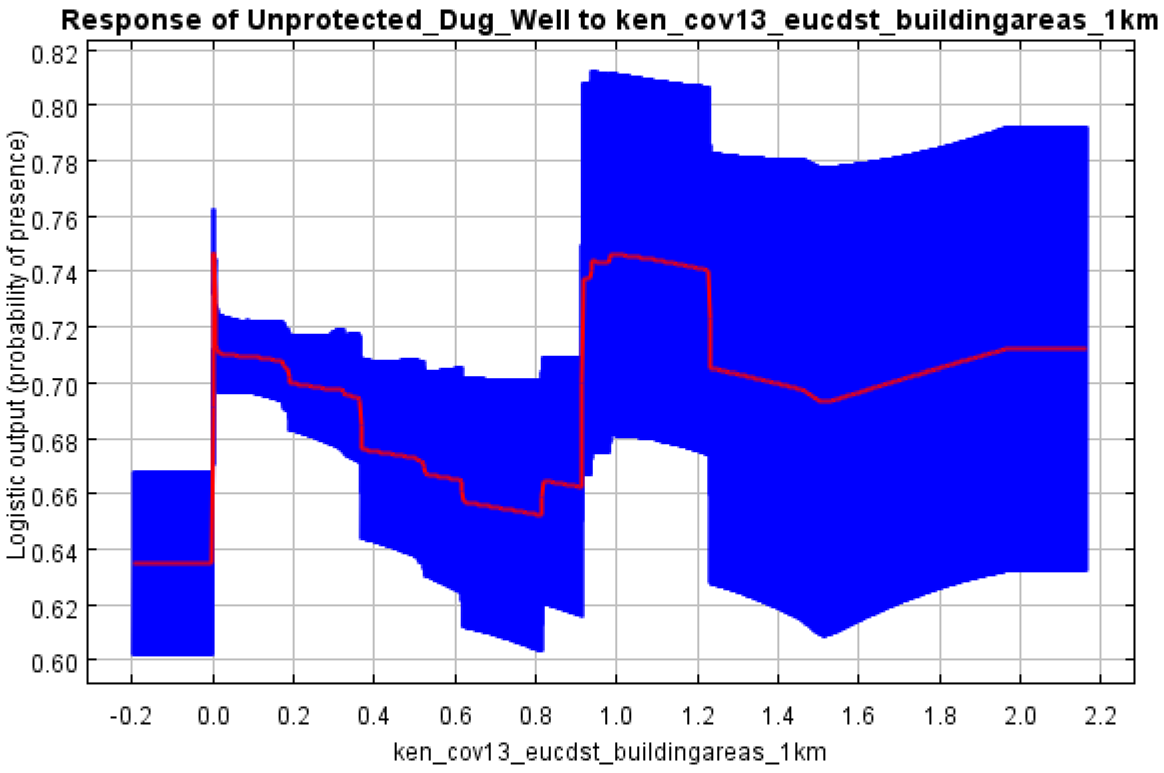

S1_Fig.63. Response curve of Euclidean distance to buildings presented as means (red) of 50 replicate runs with standard deviation in blue; model built with other predictive covariates being kept at their average sample values. X-axis: Euclidean distance (decimal degrees).

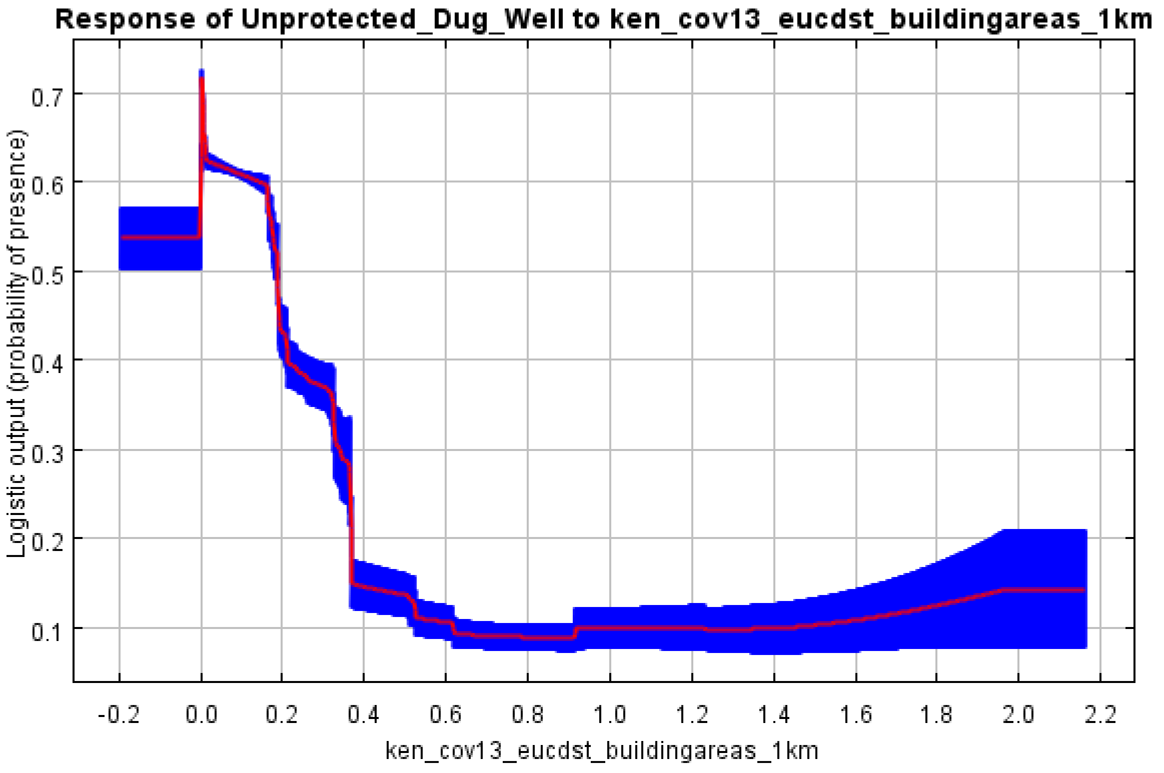

S1_Fig.64. Response curve of Euclidean distance to buildings presented as means (red) of 50 replicate runs with standard deviation in blue; model built without other predictive covariates. X-axis: Euclidean distance (decimal degrees).

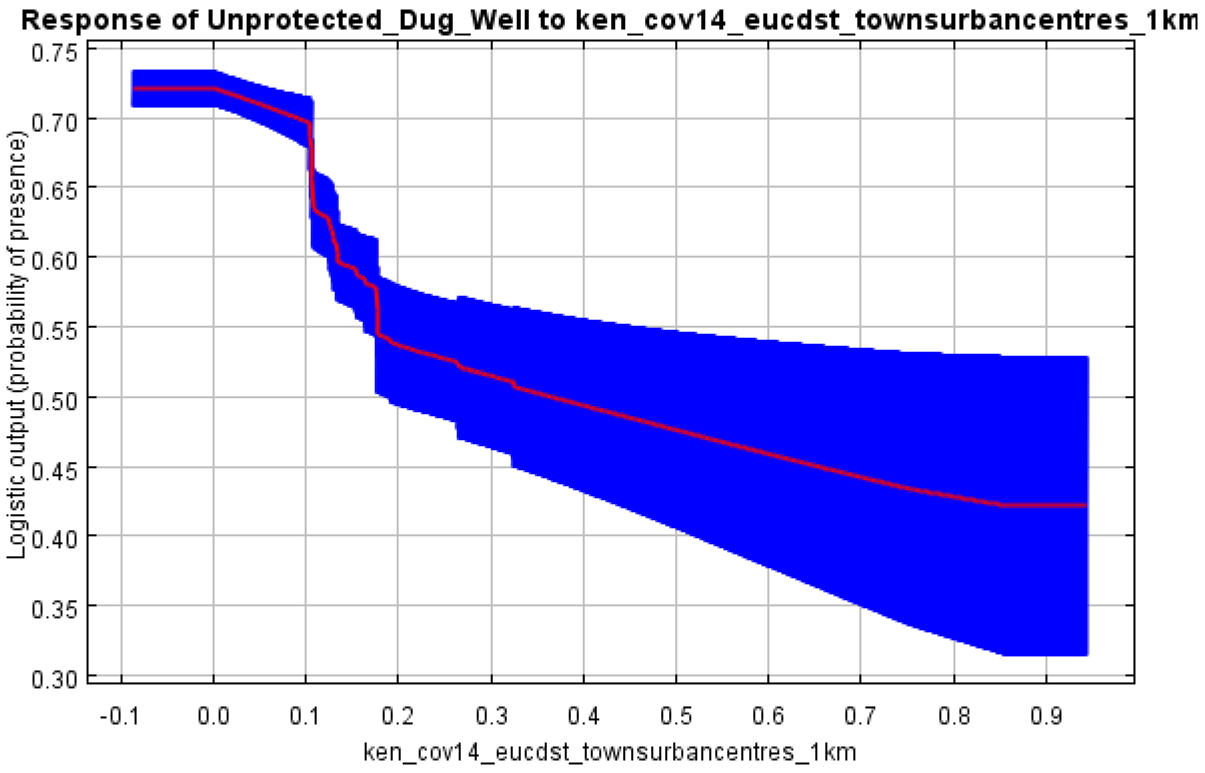

S1_Fig.65. Response curve of Euclidean distance to towns/urban centres presented as means (red) of 50 replicate runs with standard deviation in blue; model built with other predictive covariates being kept at their average sample values. X-axis: Euclidean distance (decimal degrees).

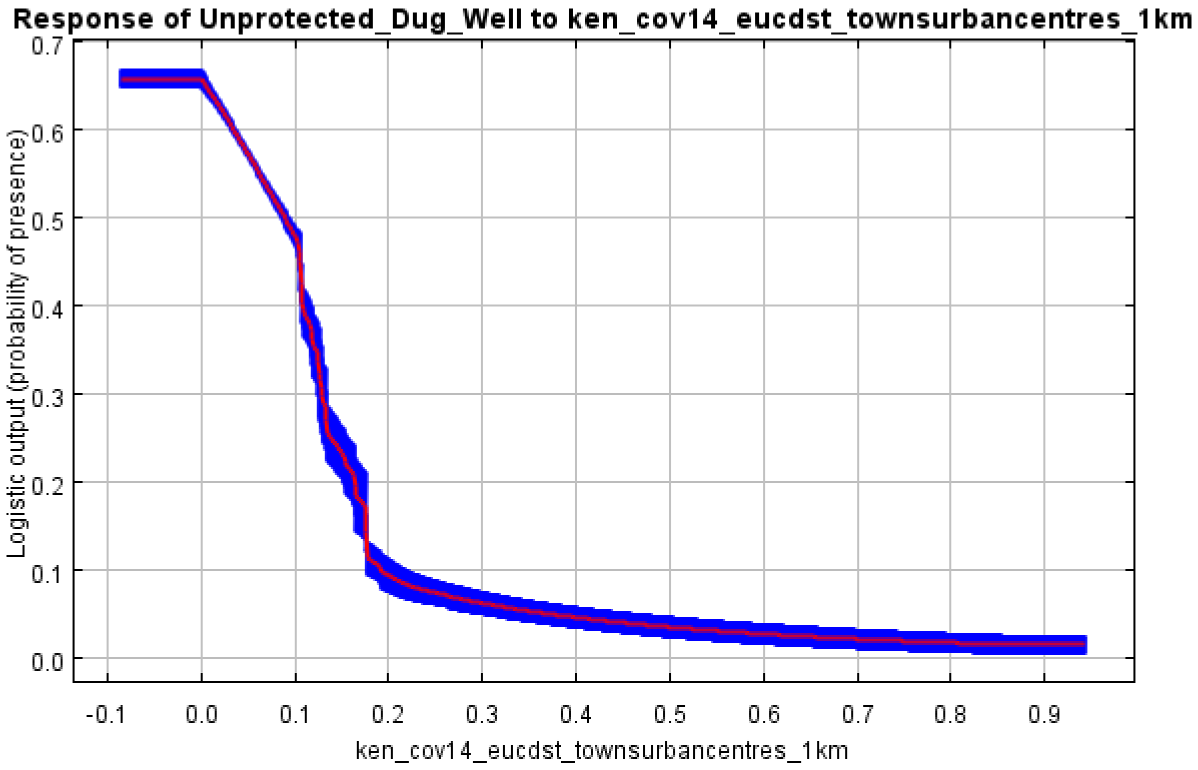

S1_Fig.66. Response curve of Euclidean distance to towns/urban centres presented as means (red) of 50 replicate runs with standard deviation in blue; model built without other predictive covariates. X-axis: Euclidean distance (decimal degrees).

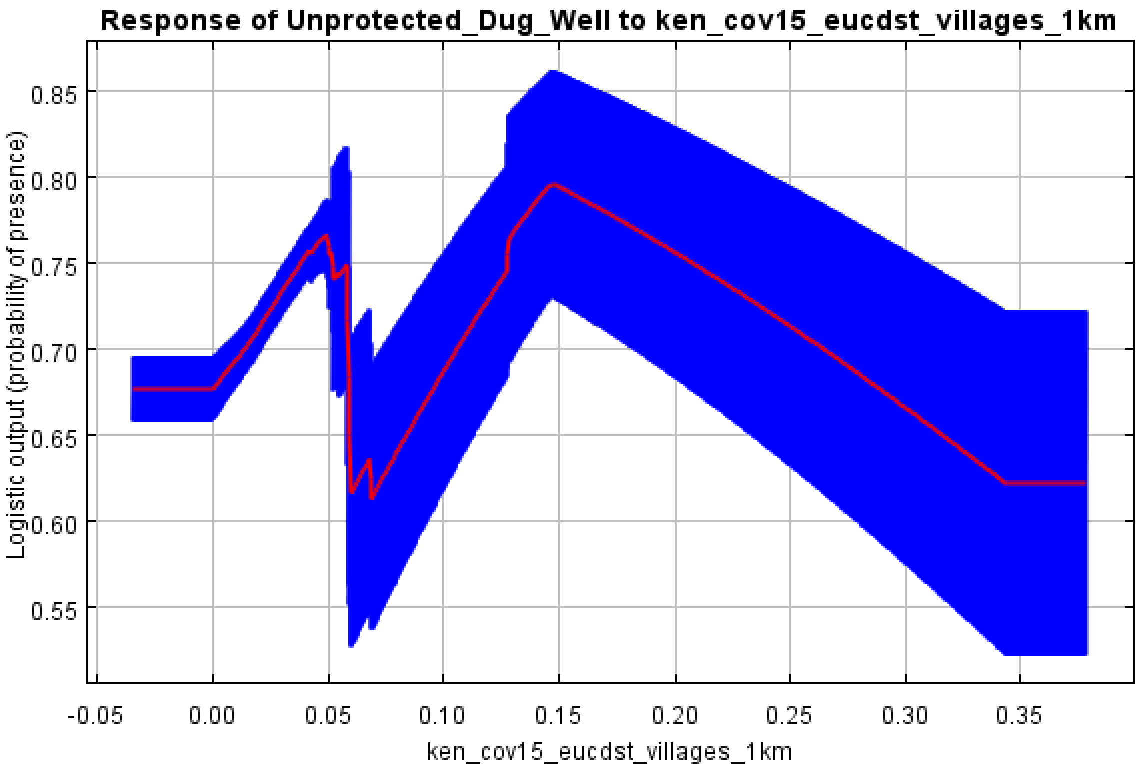

S1_Fig.67. Response curve of Euclidean distance to villages presented as means (red) of 50 replicate runs with standard deviation in blue; model built with other predictive covariates being kept at their average sample values. X-axis: Euclidean distance (decimal degrees).

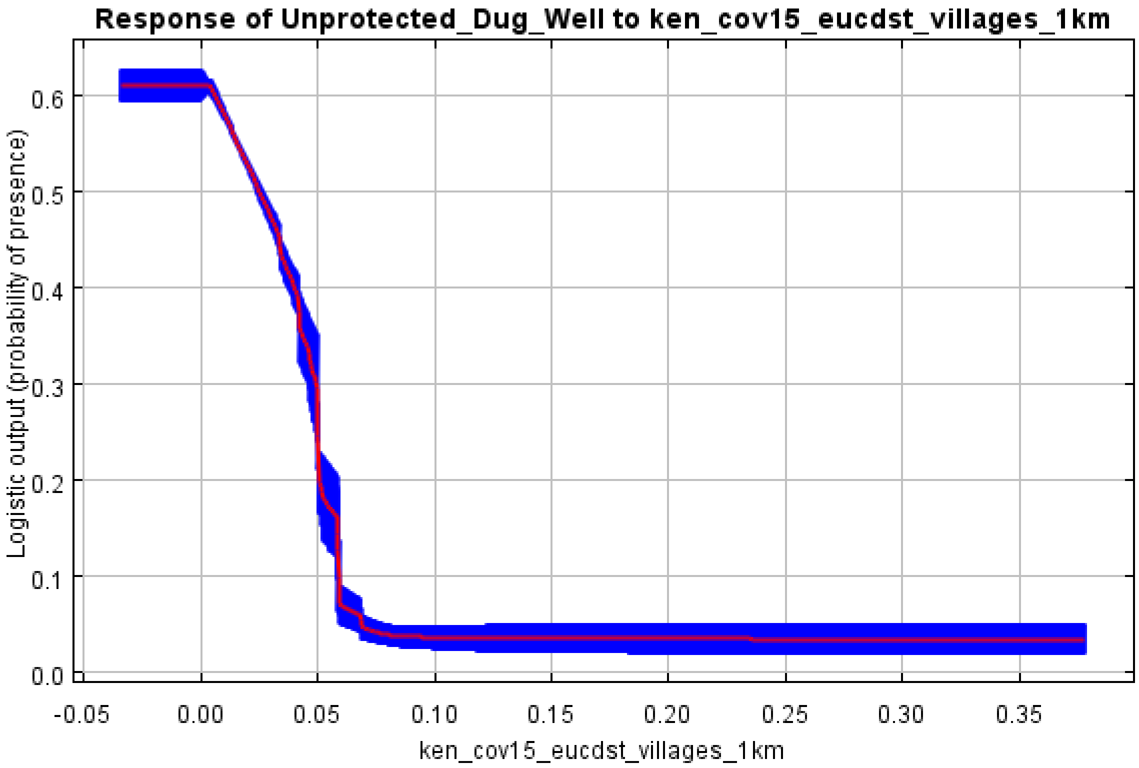

S1_Fig.68. Response curve of Euclidean distance to villages presented as means (red) of 50 replicate runs with standard deviation in blue; model built without other predictive covariates. X-axis: Euclidean distance (decimal degrees).

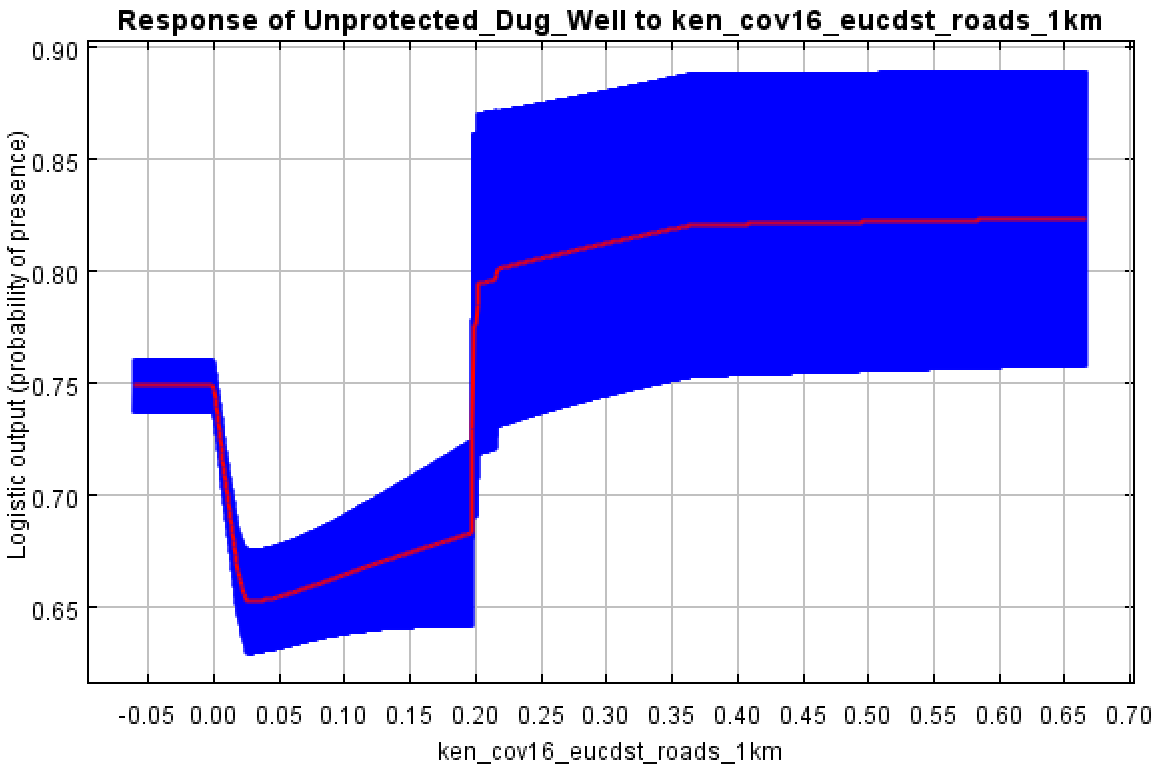

S1_Fig.69. Response curve of Euclidean distance to roads presented as means (red) of 50 replicate runs with standard deviation in blue; model built with other predictive covariates being kept at their average sample values. X-axis: Euclidean distance (decimal degrees).

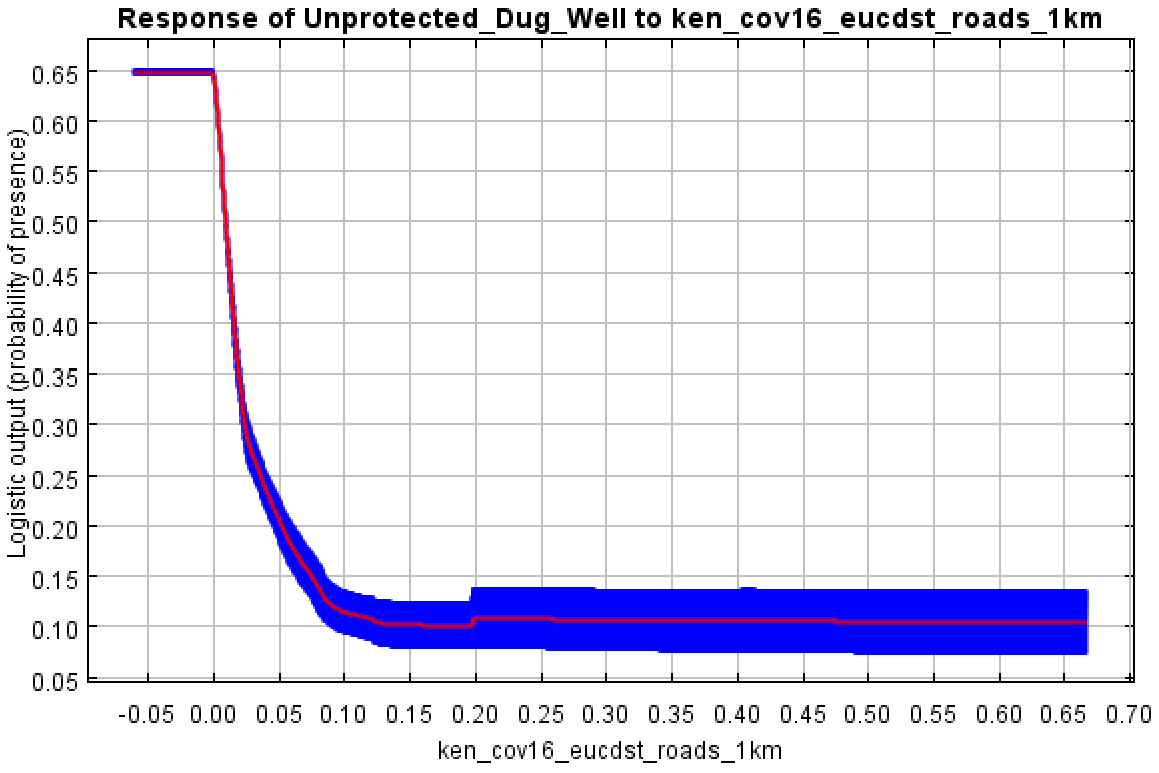

S1_Fig.70. Response curve of Euclidean distance to roads presented as means (red) of 50 replicate runs with standard deviation in blue; model built without other predictive covariates. X-axis: Euclidean distance (decimal degrees).

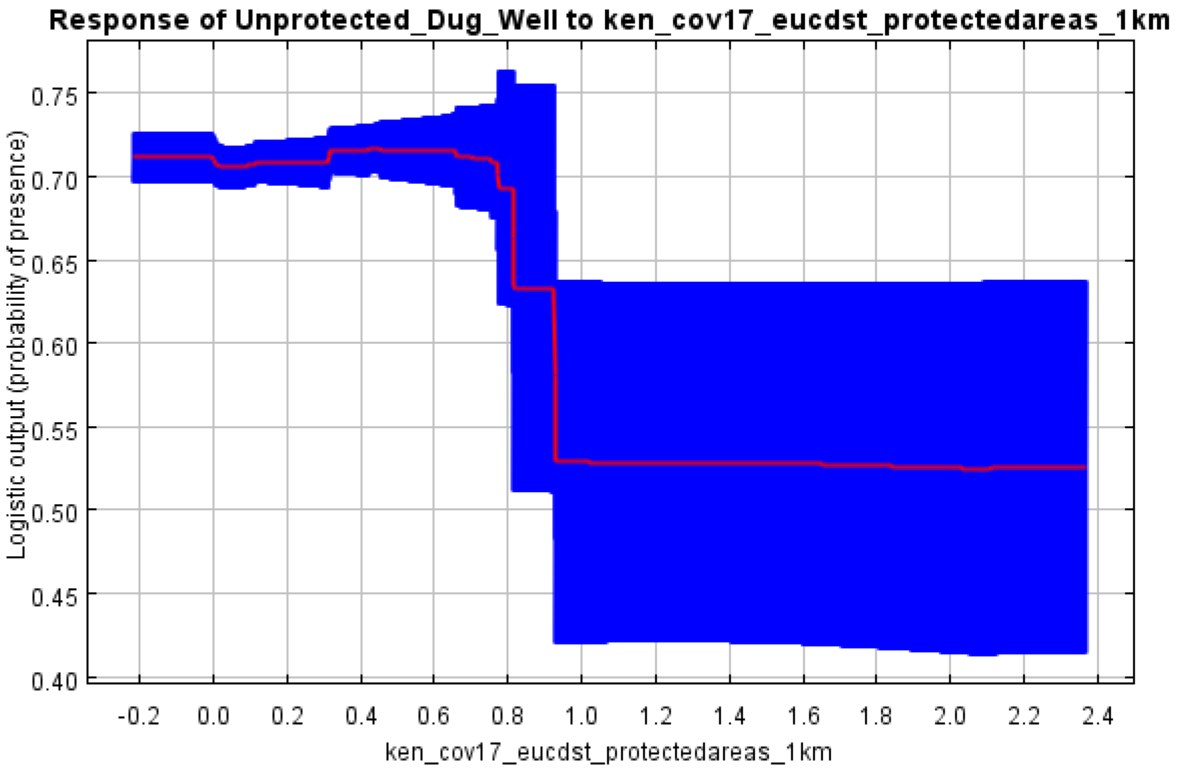

S1_Fig.71. Response curve of Euclidean distance to protected areas presented as means (red) of 50 replicate runs with standard deviation in blue; model built with other predictive covariates being kept at their average sample values. X-axis: Euclidean distance (decimal degrees).

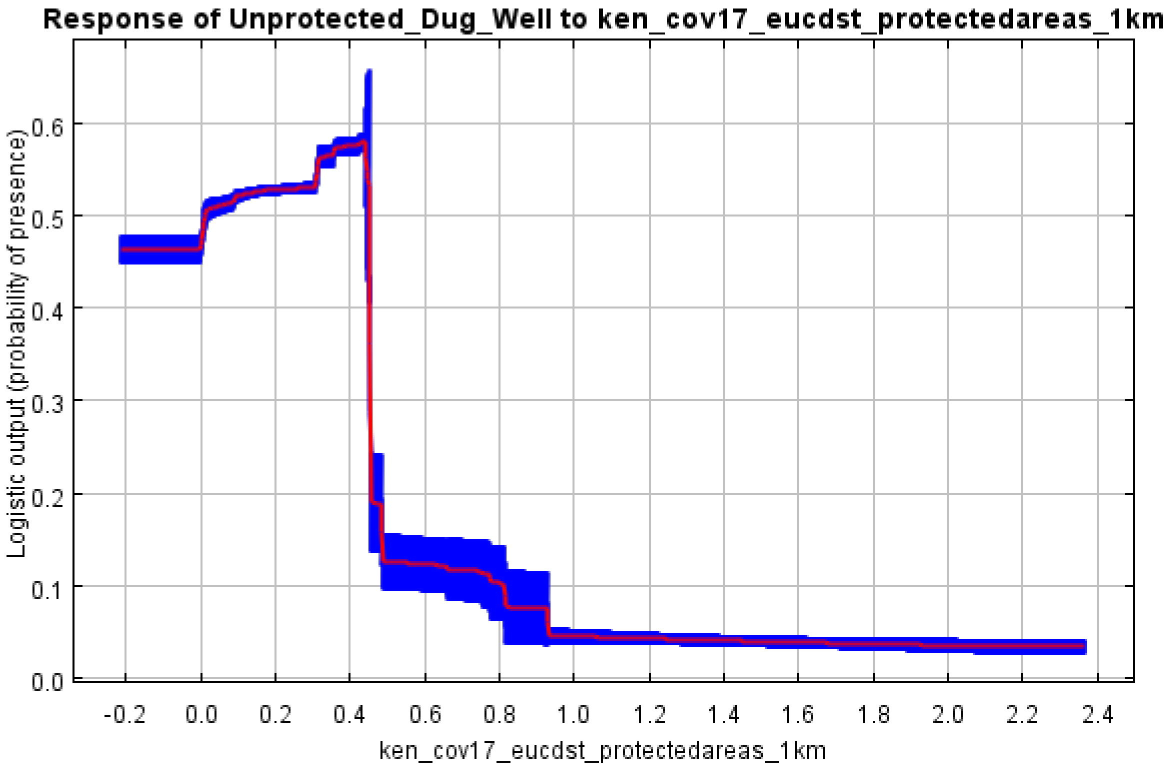

S1_Fig.72. Response curve of Euclidean distance to protected areas presented as means (red) of 50 replicate runs with standard deviation in blue; model built without other predictive covariates. X-axis: Euclidean distance (decimal degrees).

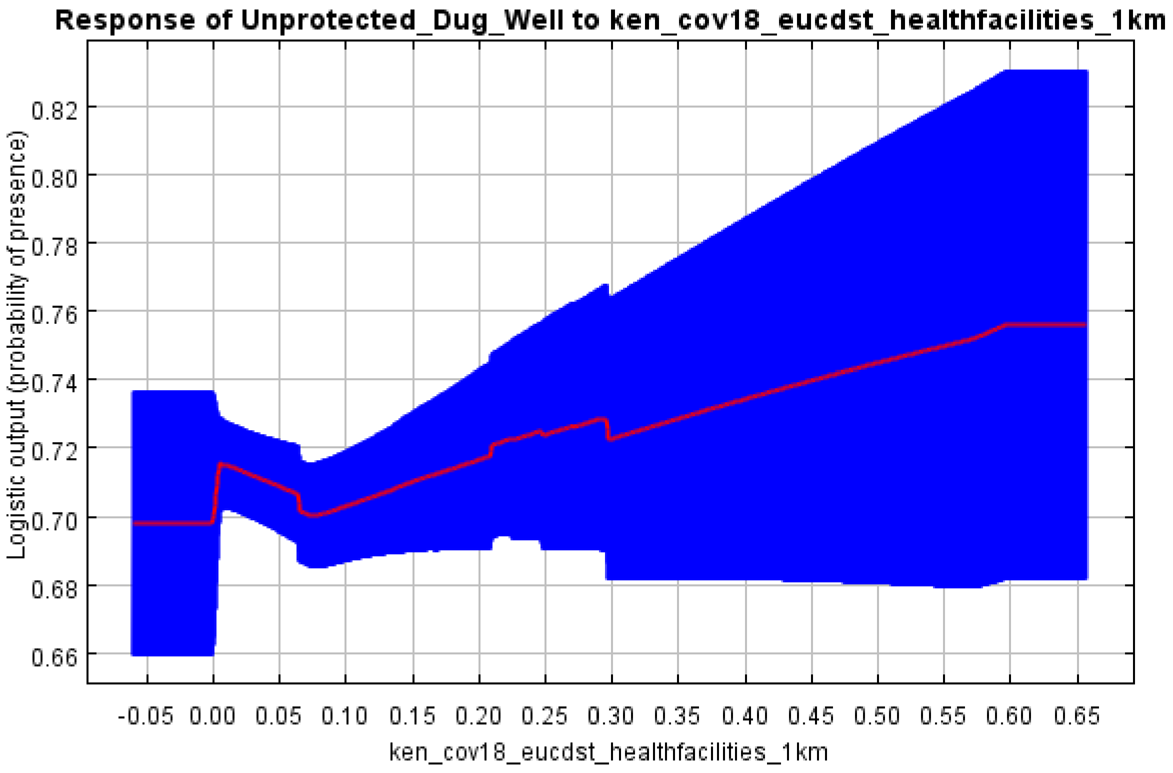

S1_Fig.73. Response curve of Euclidean distance to healthcare facilities presented as means (red) of 50 replicate runs with standard deviation in blue; model built with other predictive covariates being kept at their average sample values. X-axis: Euclidean distance (decimal degrees).

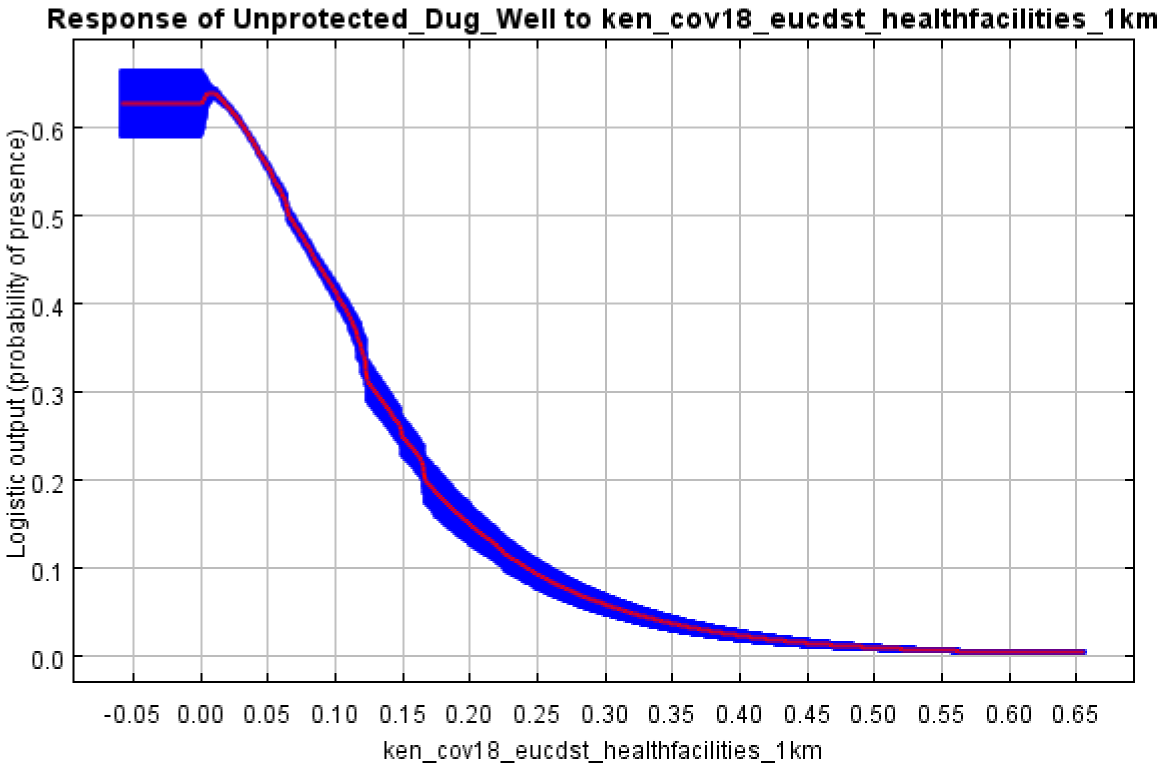

S1_Fig.74. Response curve of Euclidean distance to healthcare facilities presented as means (red) of 50 replicate runs with standard deviation in blue; model built without other predictive covariates. X-axis: Euclidean distance (decimal degrees).

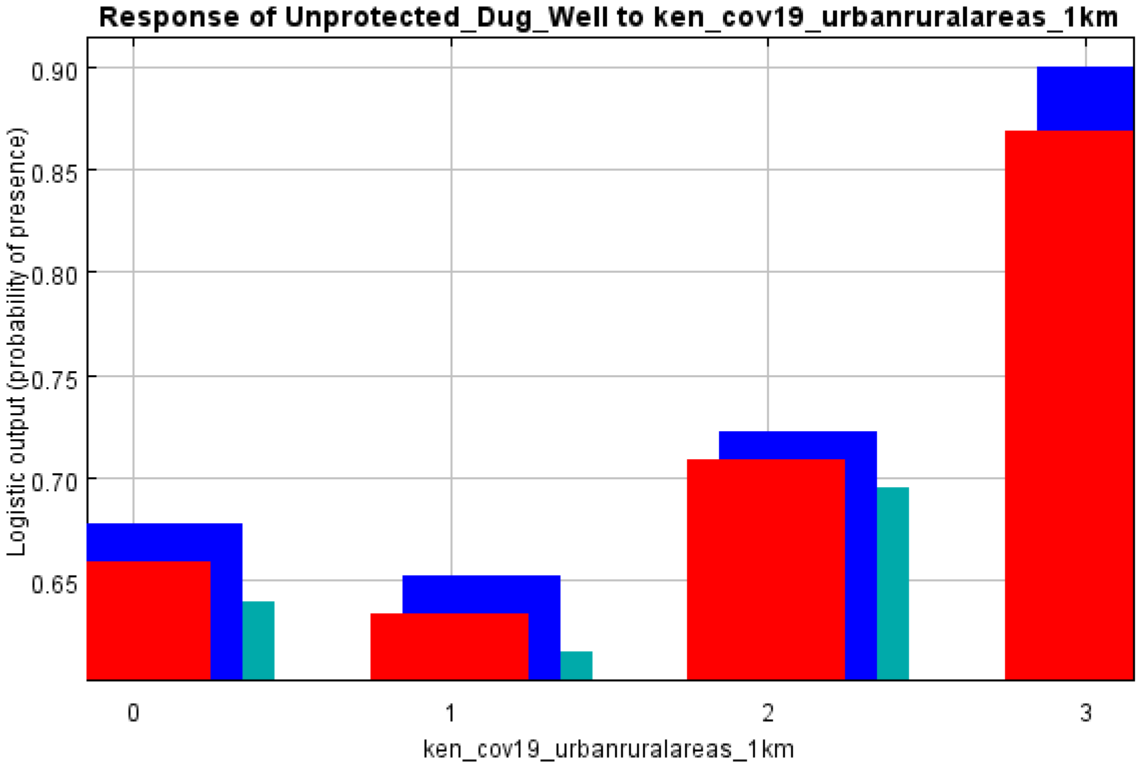

S1_Fig.75. Response curve of rural/urban areas presented as means (red) of 50 replicate runs with standard deviation in blue; model built with other predictive covariates being kept at their average sample values. X-axis: rurality: 0 – Others (not populated/no data); 1 – Rural areas; 2 – Urban clusters; 3 – Urban centres.

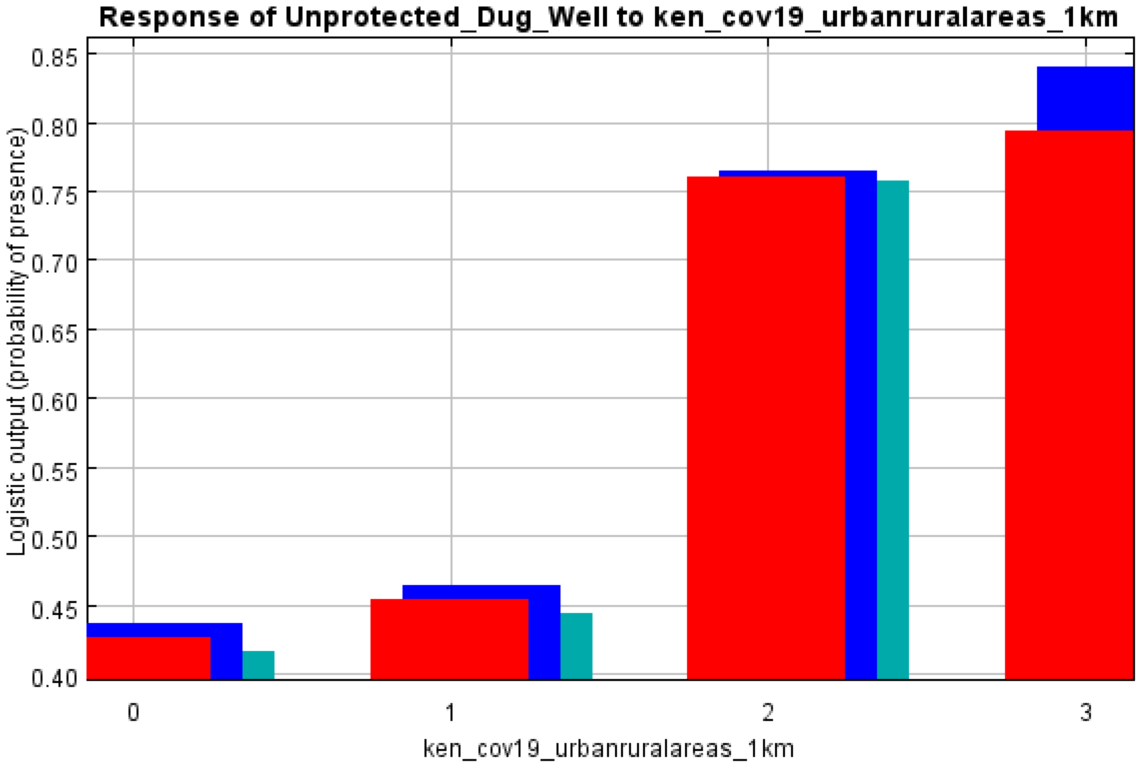

S1_Fig.76. Response curve of rural/urban areas presented as means (red) of 50 replicate runs with standard deviation in blue; model built without other predictive covariates. X-axis: rurality: 0 – Others (not populated/no data); 1 – Rural areas; 2 – Urban clusters; 3 – Urban centres.

Surface water (restricted background)

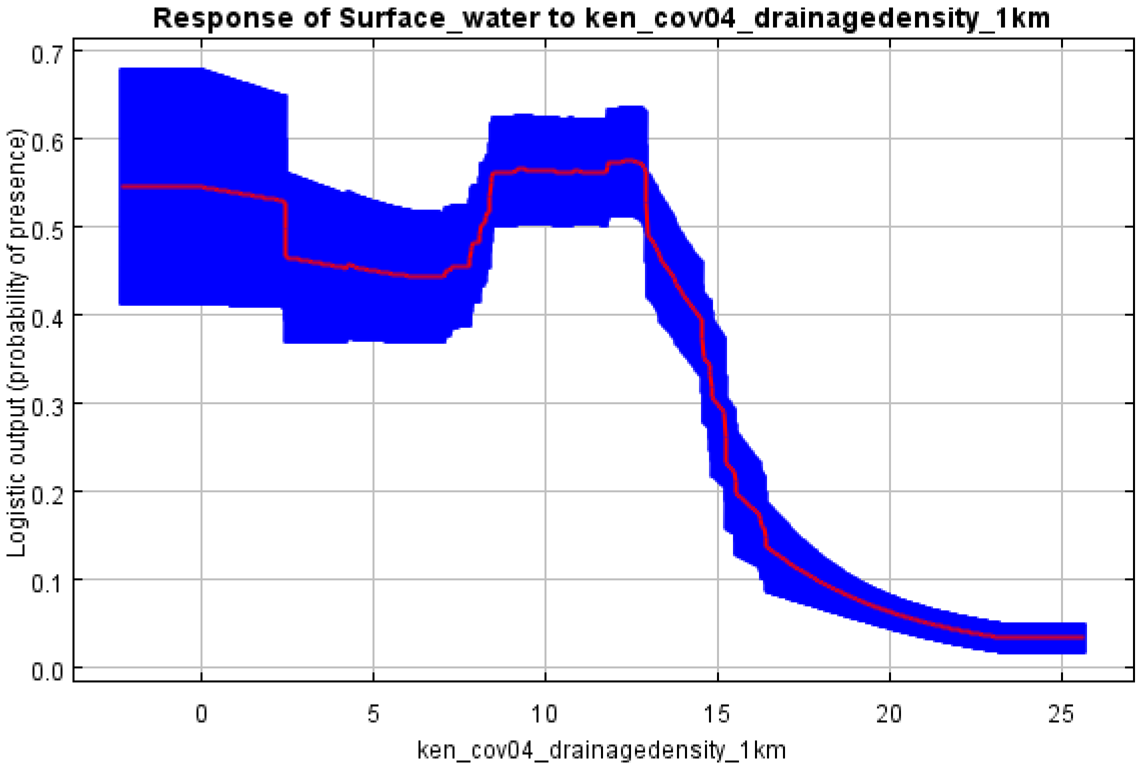

S1_Fig.77. Response curve of drainage density presented as means (red) of 50 replicate runs with standard deviation in blue; model built with other predictive covariates being kept at their average sample values. X-axis: drainage density – channel length per area size of a grid cell (lengths in decimal degrees).

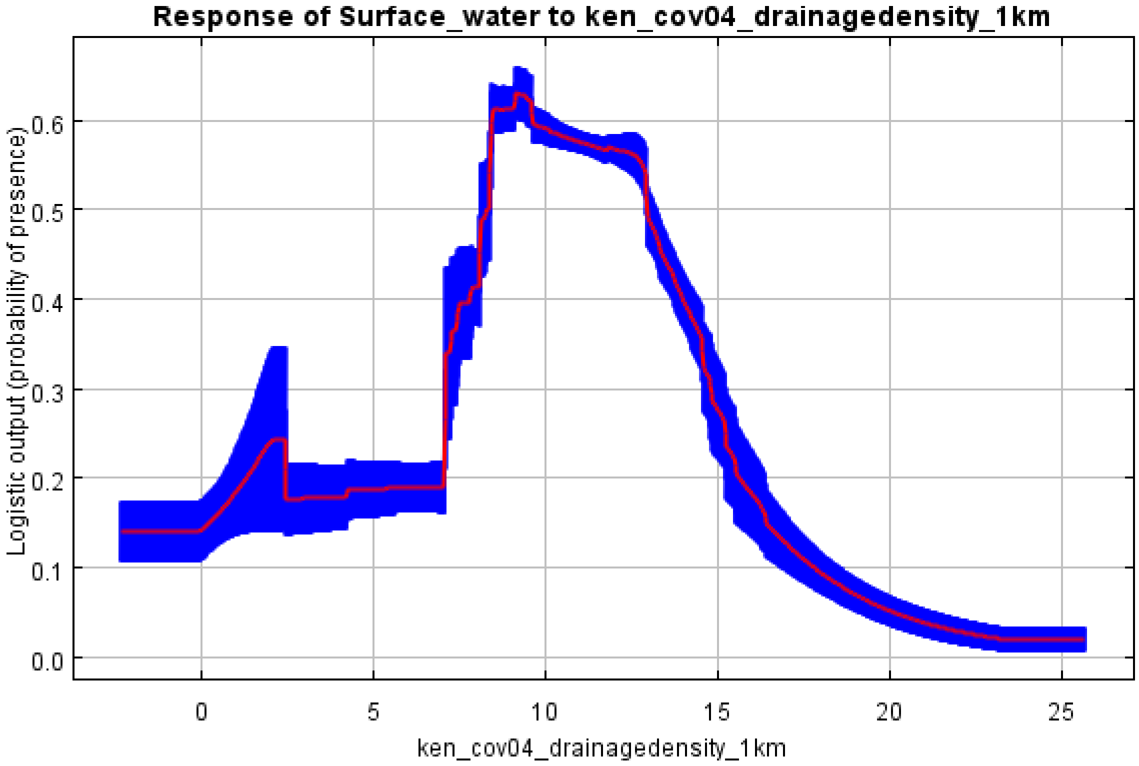

S1_Fig.78. Response curve of drainage density presented as means (red) of 50 replicate runs with standard deviation in blue; model built without other predictive covariates. X-axis: drainage density – channel length per area size of a grid cell (lengths in decimal degrees).

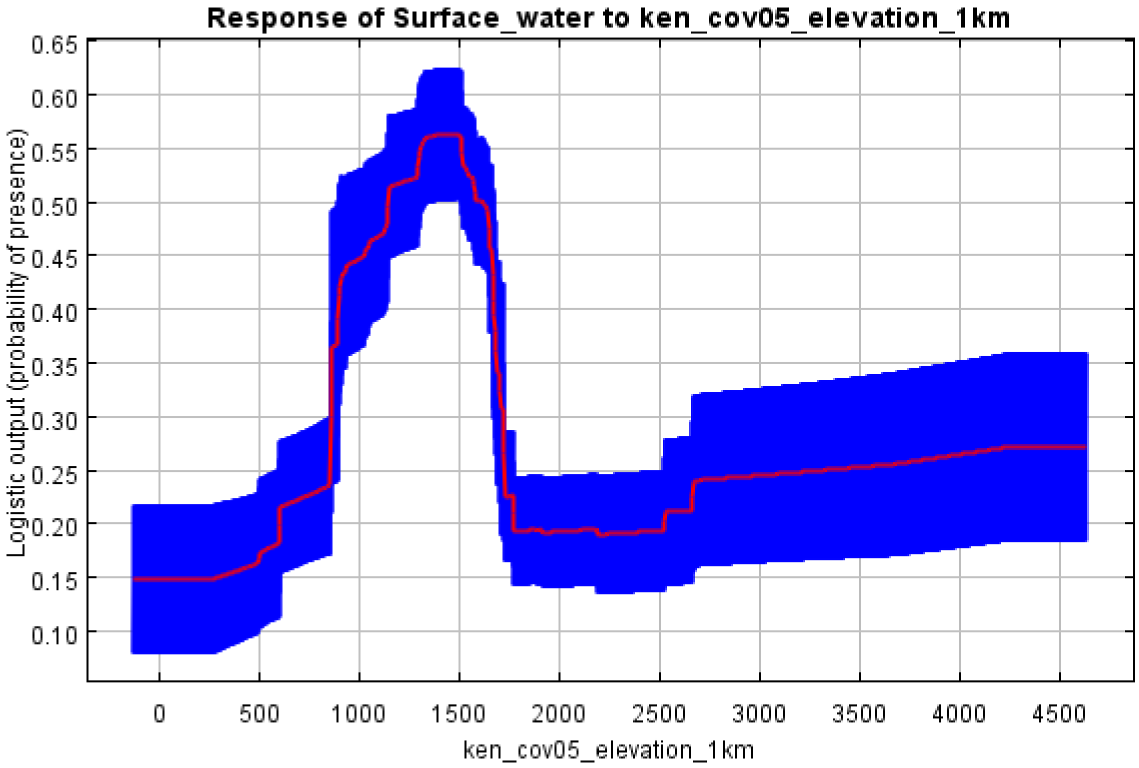

S1_Fig.79. Response curve of elevation presented as means (red) of 50 replicate runs with standard deviation in blue; model built with other predictive covariates being kept at their average sample values. X-axis: elevation (m).

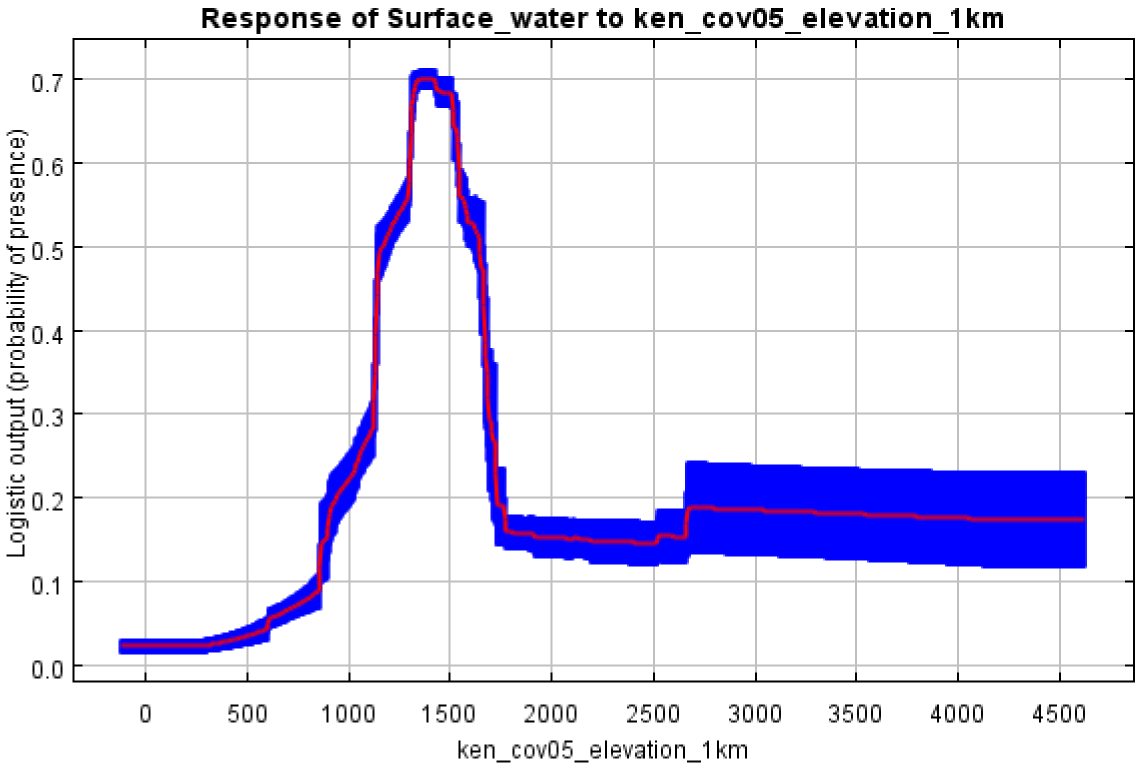

S1_Fig.80. Response curve of elevation presented as means (red) of 50 replicate runs with standard deviation in blue; model built without other predictive covariates. X-axis: elevation (m).

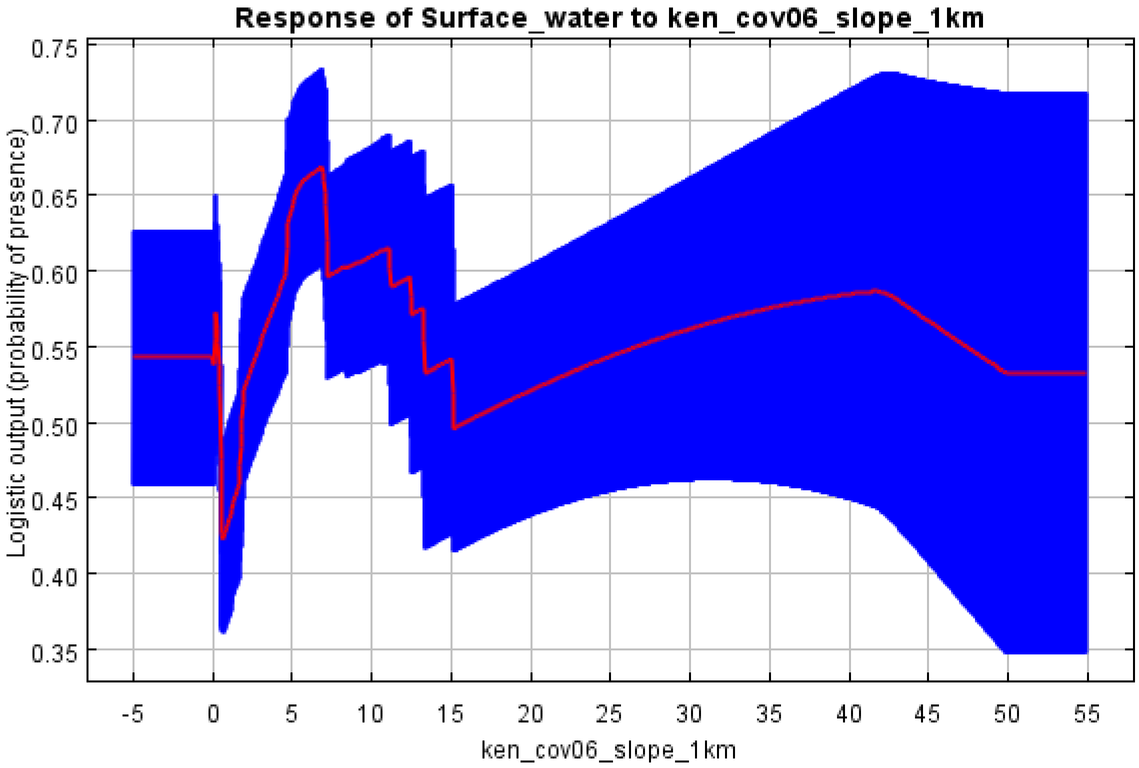

S1_Fig.81. Response curve of slope presented as means (red) of 50 replicate runs with standard deviation in blue; model built with other predictive covariates being kept at their average sample values. X-axis: slope (degree).

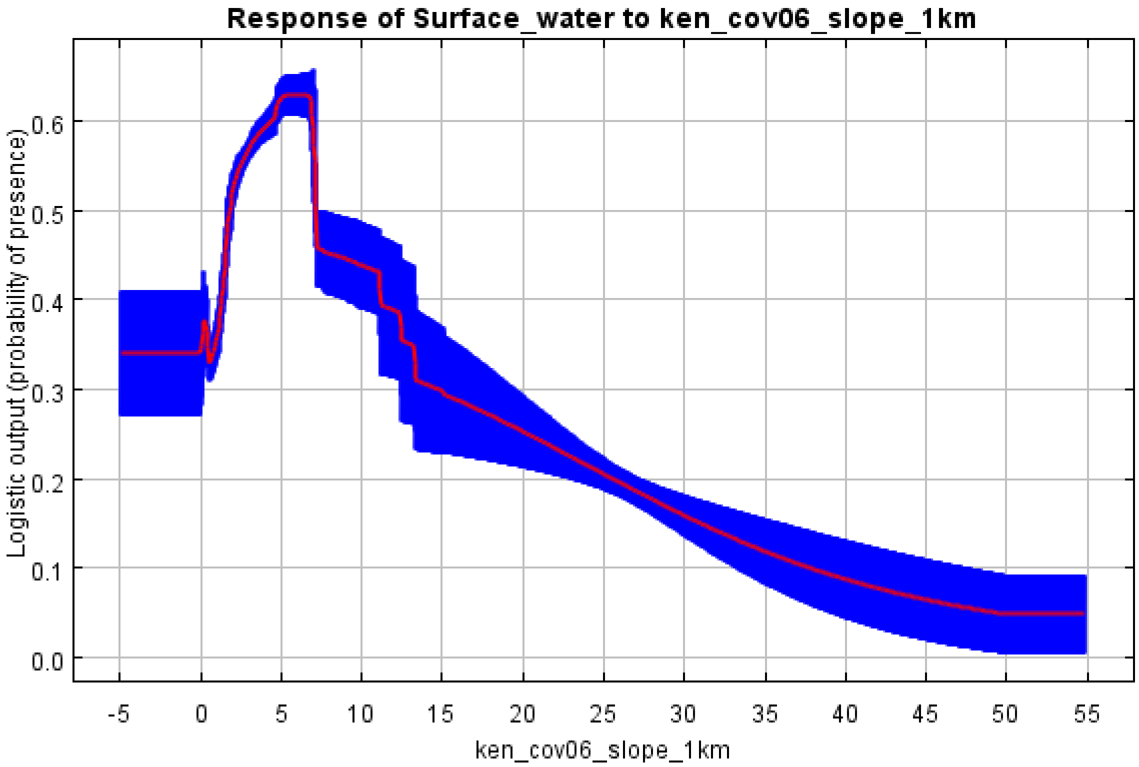

S1_Fig.82. Response curve of slope presented as means (red) of 50 replicate runs with standard deviation in blue; model built without other predictive covariates. X-axis: slope (degree).

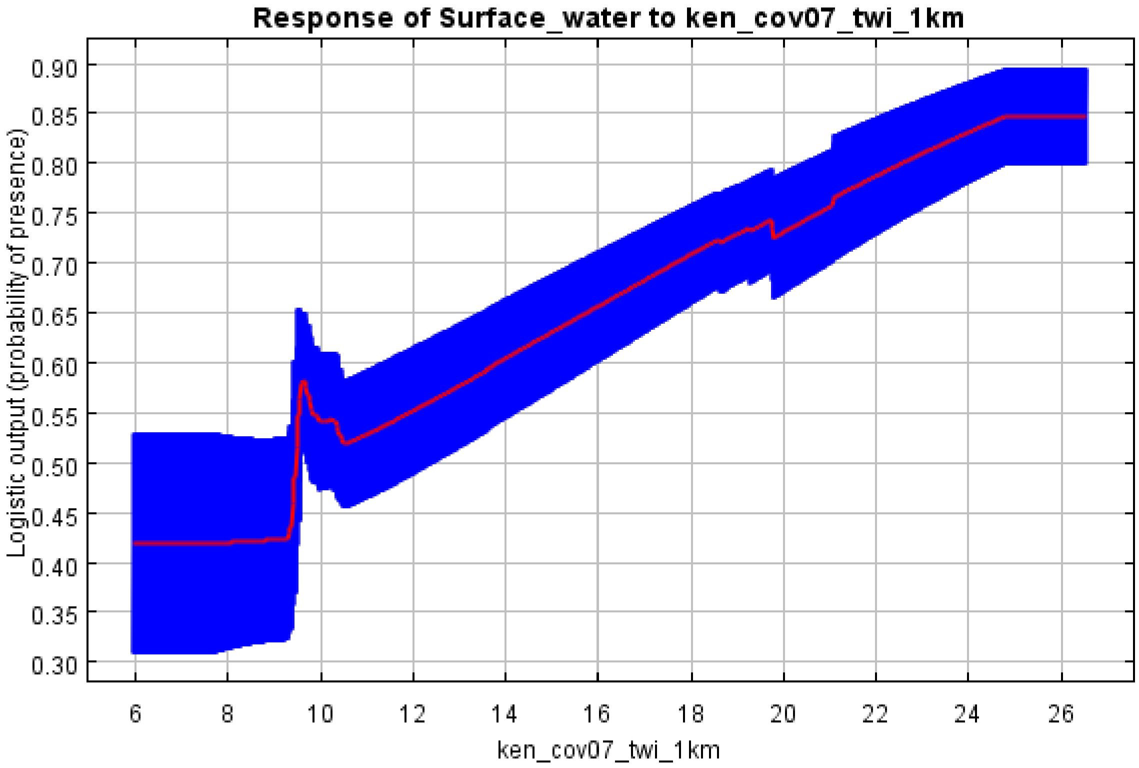

S1_Fig.83. Response curve of topographic wetness index presented as means (red) of 50 replicate runs with standard deviation in blue; model built with other predictive covariates being kept at their average sample values. X-axis: topographic unit index.

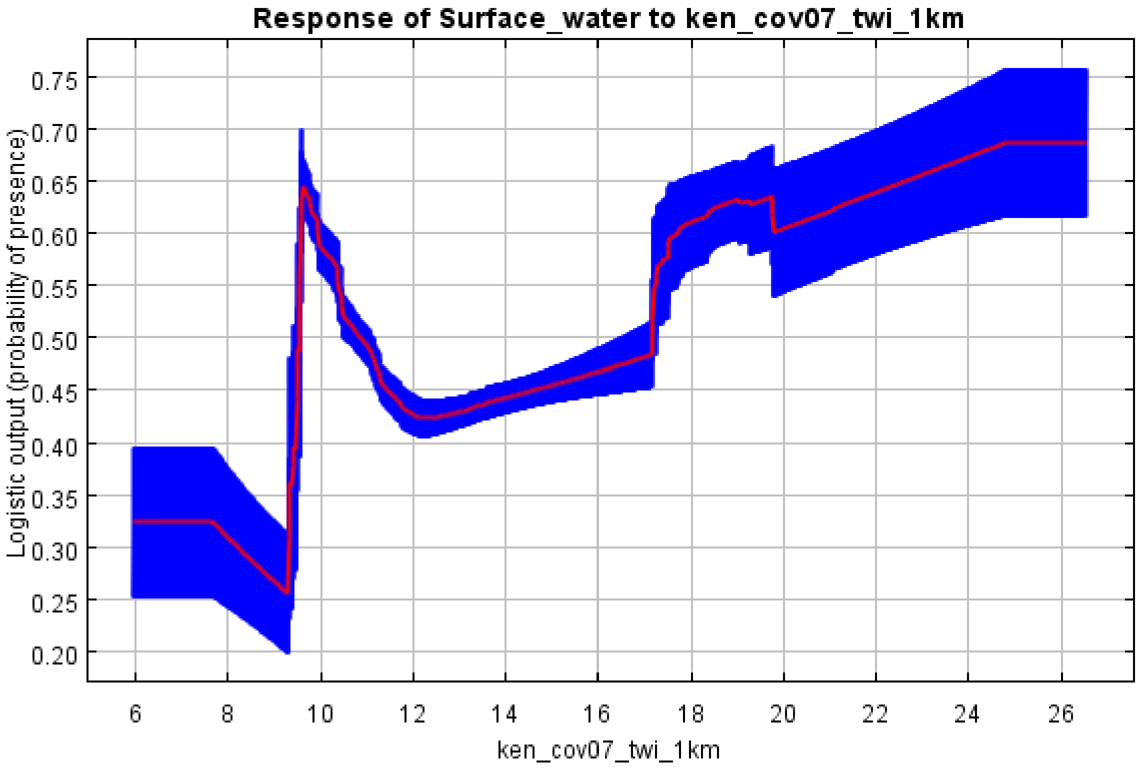

S1_Fig.84. Response curve of topographic wetness index presented as means (red) of 50 replicate runs with standard deviation in blue; model built without other predictive covariates. X-axis: topographic unit index.

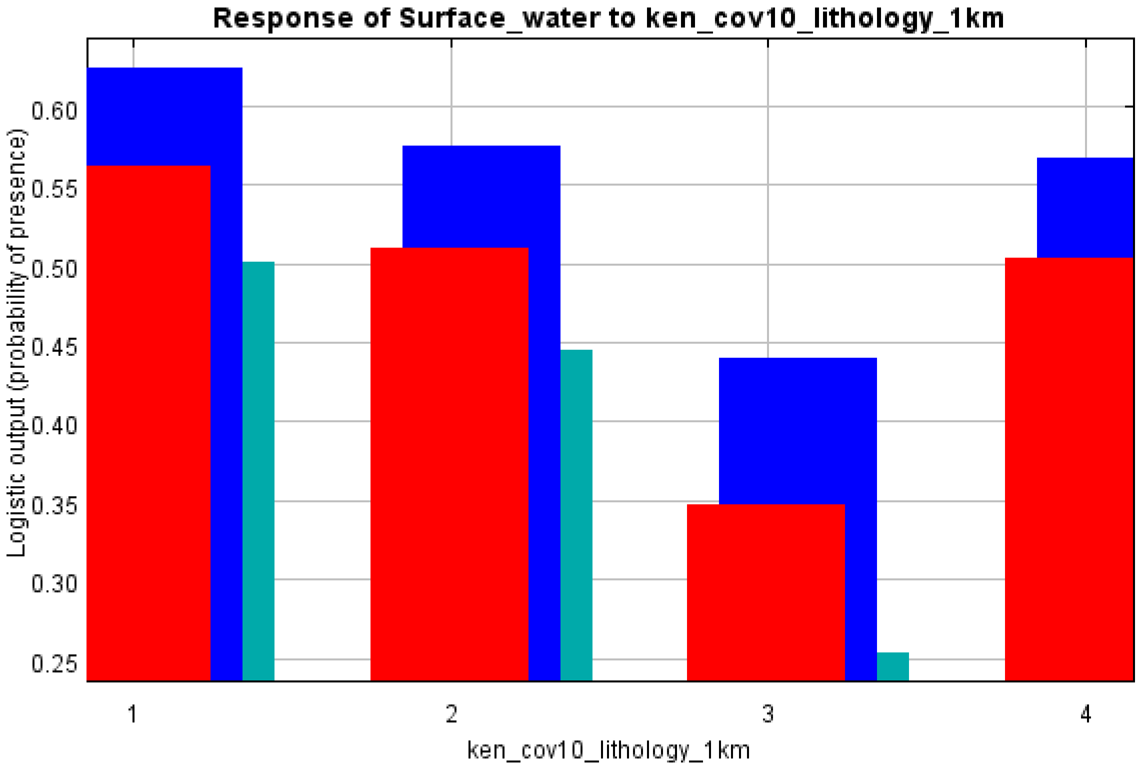

S1_Fig.85. Response curve of lithology presented as means (red) of 50 replicate runs with standard deviation in blue; model built with other predictive covariates being kept at their average sample values. X-axis: lithology: 1 – Igneous; 2 – Metamorphic rock; 3 – Sedimentary rock; 4 – Unconsolidated.

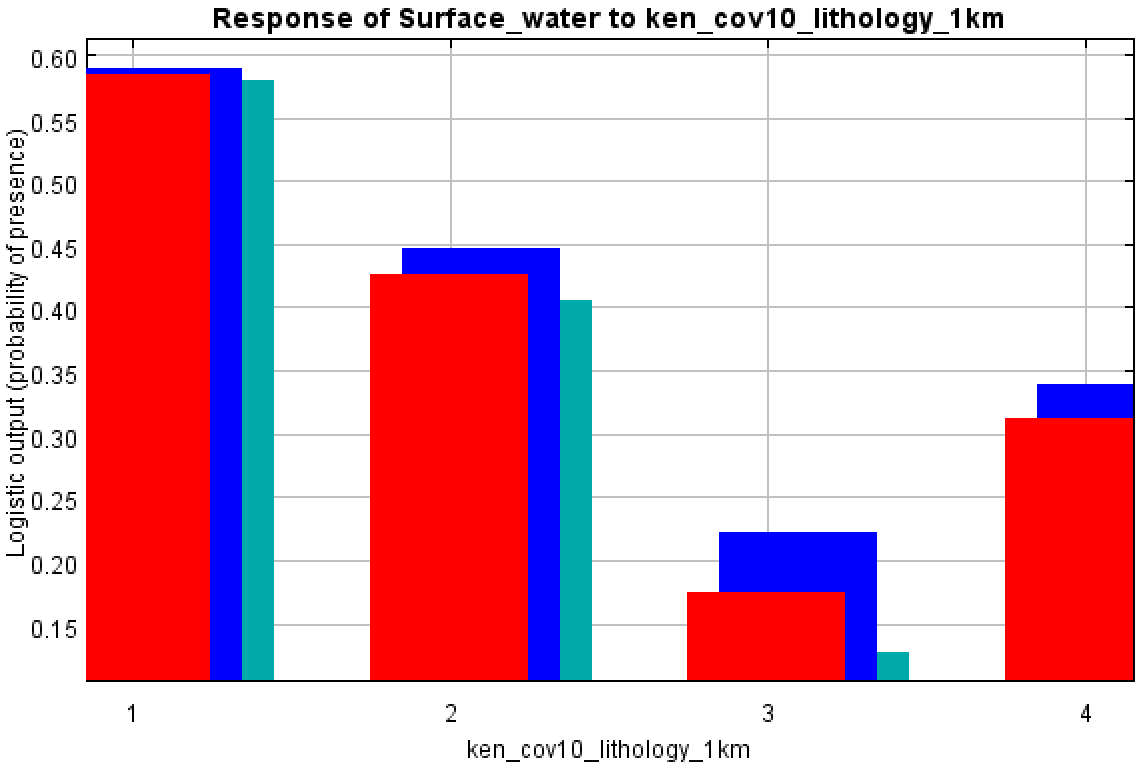

S1_Fig.86. Response curve of lithology presented as means (red) of 50 replicate runs with standard deviation in blue; model built without other predictive covariates. X-axis: lithology: 1 – Igneous; 2 – Metamorphic rock; 3 – Sedimentary rock; 4 – Unconsolidated.

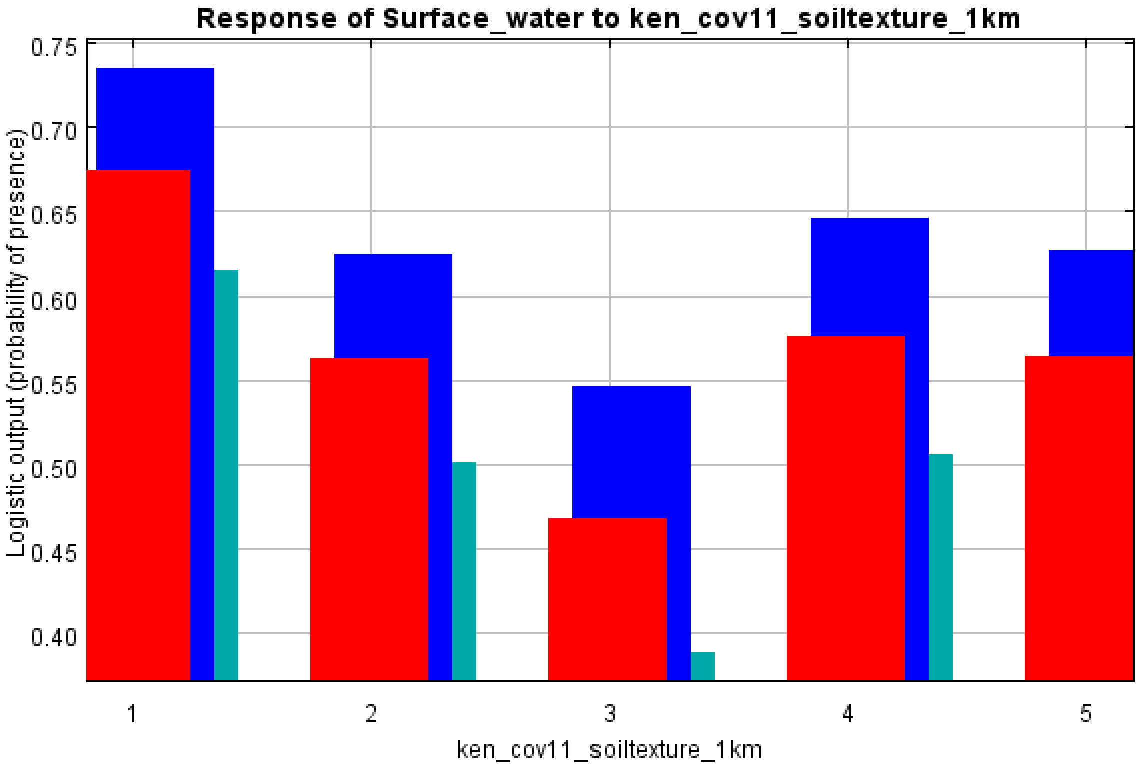

S1_Fig.87. Response curve of soil texture presented as means (red) of 50 replicate runs with standard deviation in blue; model built with other predictive covariates being kept at their average sample values. X-axis: lithology: 1 – Very clayey; 2 – Clayey; 3 – Loamy; 4 – Sandy; 5 – Extremely sandy.

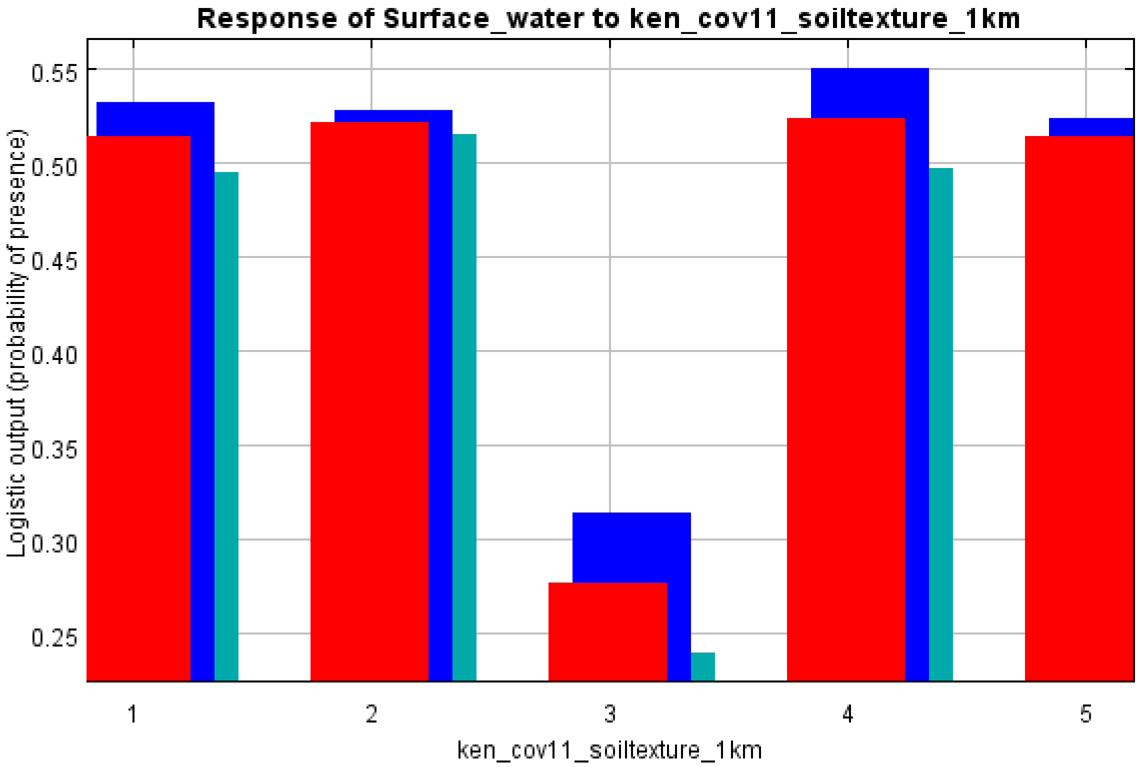

S1_Fig.88. Response curve of soil texture presented as means (red) of 50 replicate runs with standard deviation in blue; model built without other predictive covariates. X-axis: lithology: 1 – Very clayey; 2 – Clayey; 3 – Loamy; 4 – Sandy; 5 – Extremely sandy.

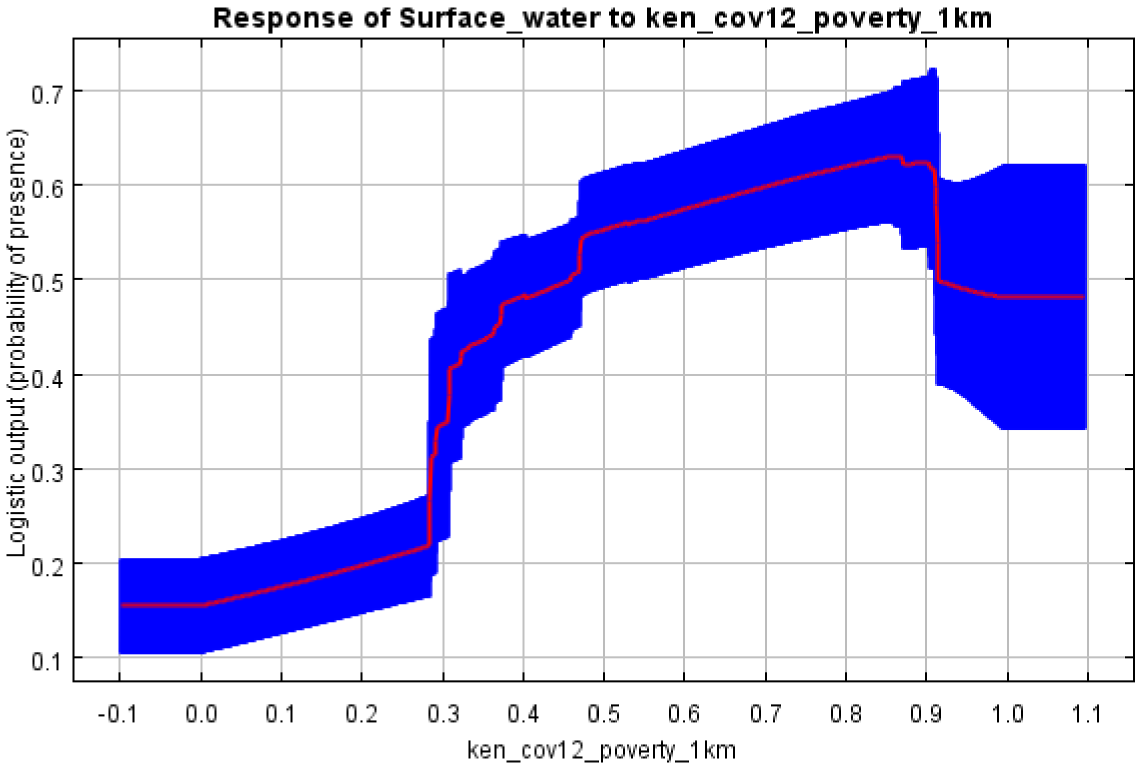

S1_Fig.89. Response curve of poverty presented as means (red) of 50 replicate runs with standard deviation in blue; model built with other predictive covariates being kept at their average sample values. X-axis: proportion of residents living in MPI-defined poverty.

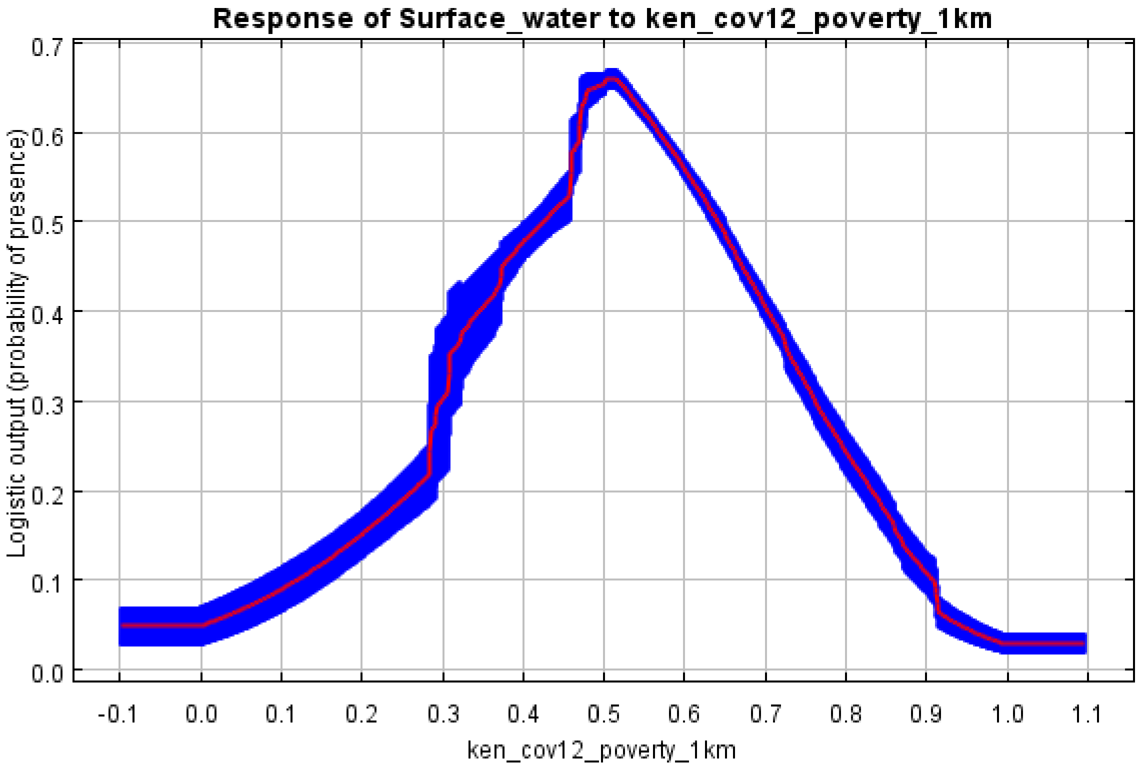

S1_Fig.90. Response curve of poverty presented as means (red) of 50 replicate runs with standard deviation in blue; model built without other predictive covariates. X-axis: proportion of residents living in MPI-defined poverty.

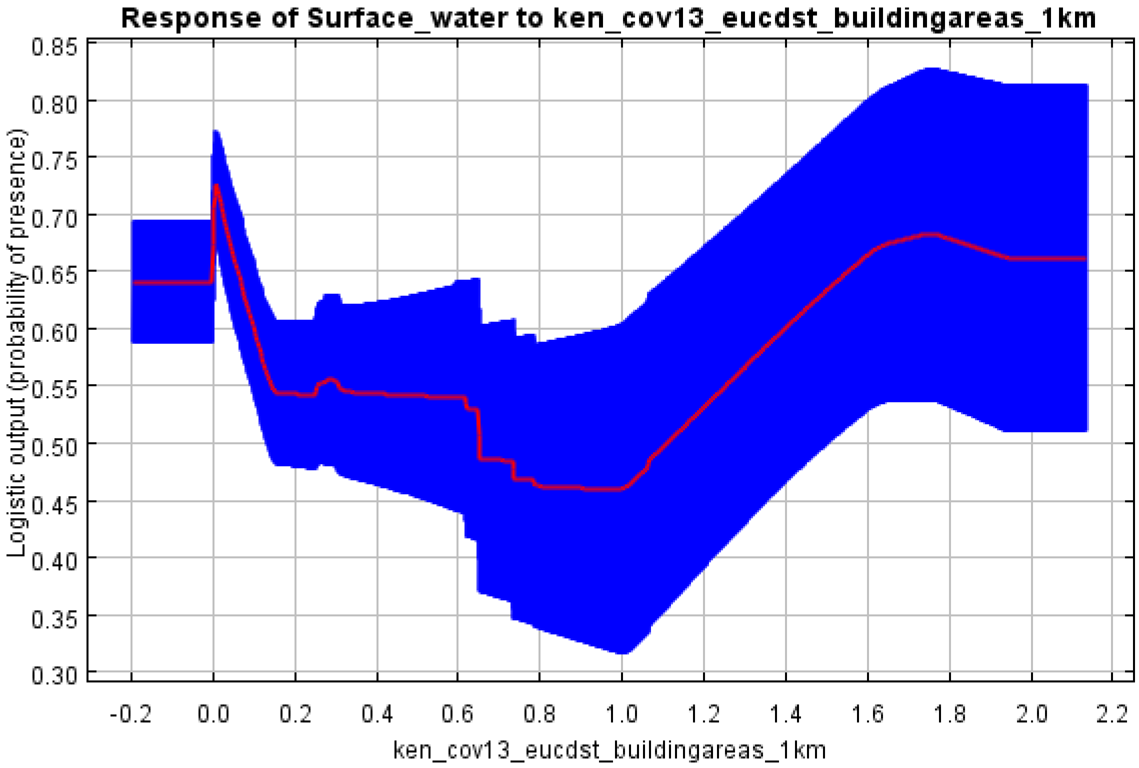

S1_Fig.91. Response curve of Euclidean distance to buildings presented as means (red) of 50 replicate runs with standard deviation in blue; model built with other predictive covariates being kept at their average sample values. X-axis: Euclidean distance (decimal degrees).

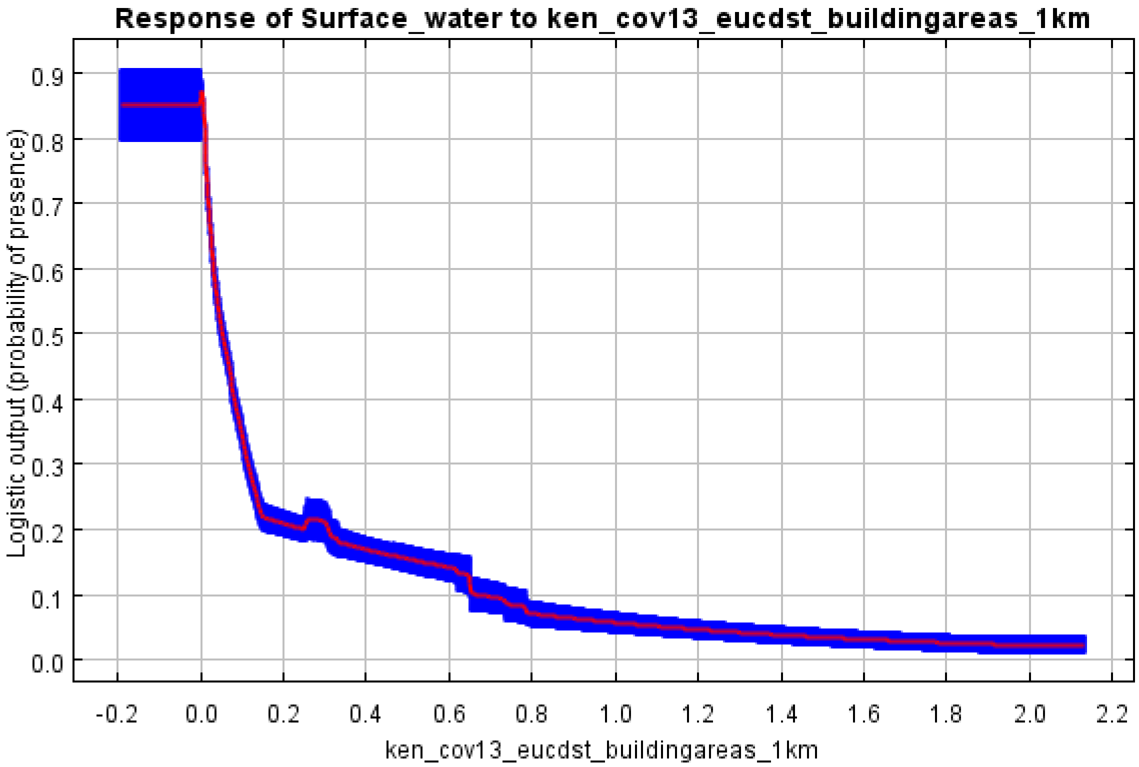

S1_Fig.92. Response curve of Euclidean distance to buildings presented as means (red) of 50 replicate runs with standard deviation in blue; model built without other predictive covariates. X-axis: Euclidean distance (decimal degrees).

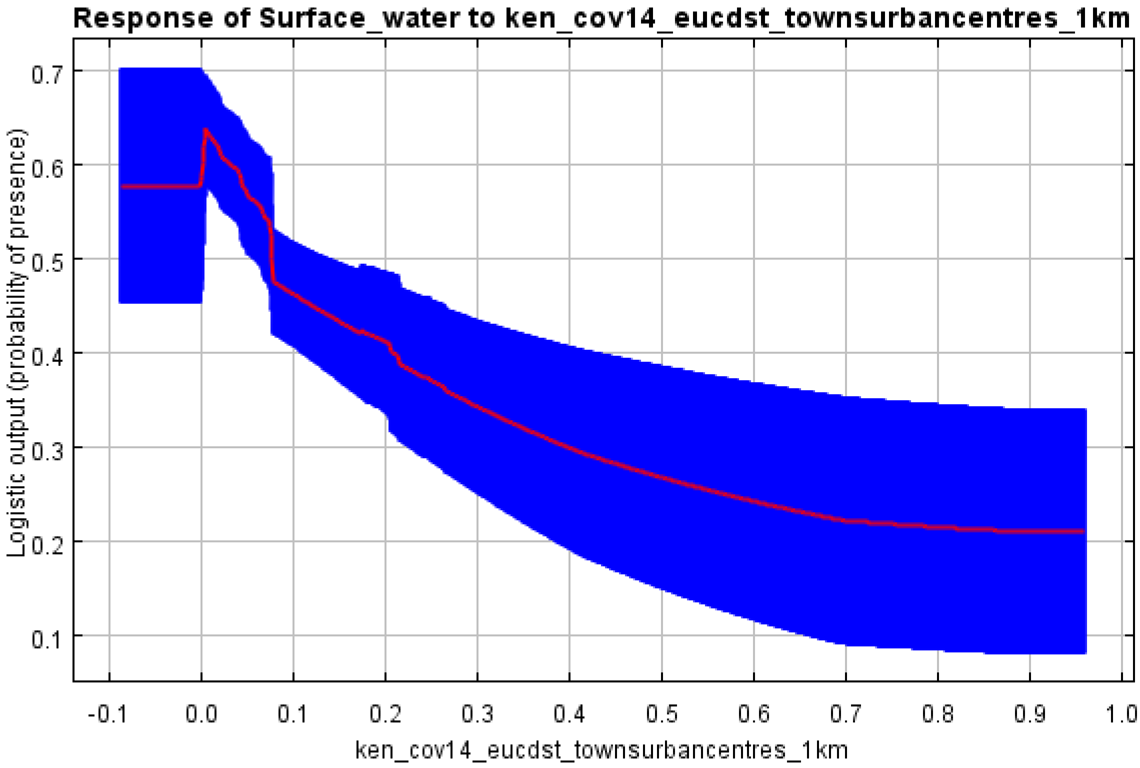

S1_Fig.93. Response curve of Euclidean distance to towns/urban centres presented as means (red) of 50 replicate runs with standard deviation in blue; model built with other predictive covariates being kept at their average sample values. X-axis: Euclidean distance (decimal degrees).

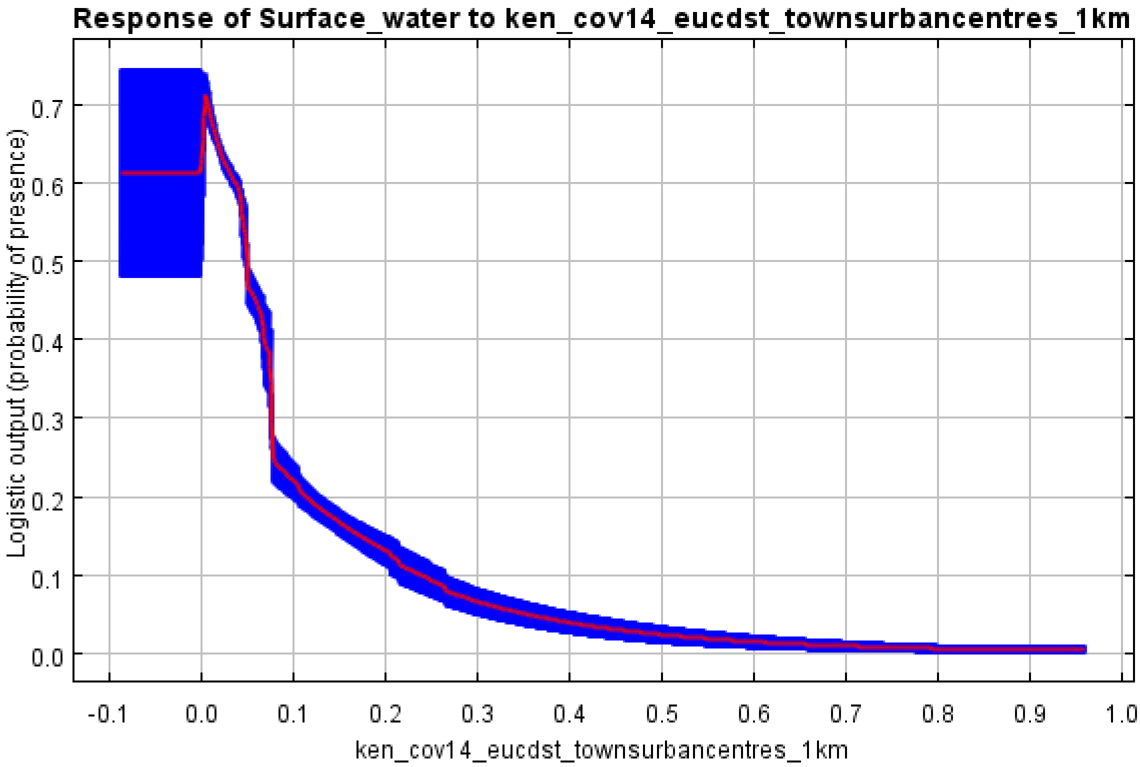

S1_Fig.94. Response curve of Euclidean distance to towns/urban centres presented as means (red) of 50 replicate runs with standard deviation in blue; model built without other predictive covariates. X-axis: Euclidean distance (decimal degrees).

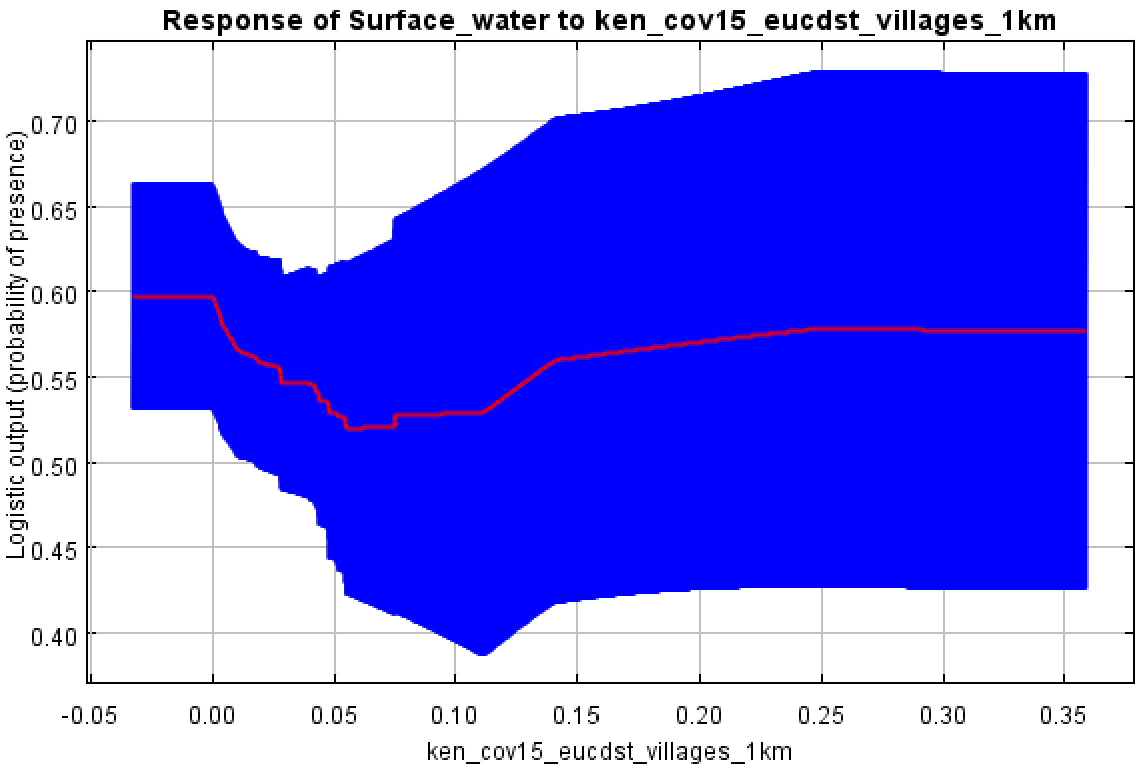

S1_Fig.95. Response curve of Euclidean distance to villages presented as means (red) of 50 replicate runs with standard deviation in blue; model built with other predictive covariates being kept at their average sample values. X-axis: Euclidean distance (decimal degrees).

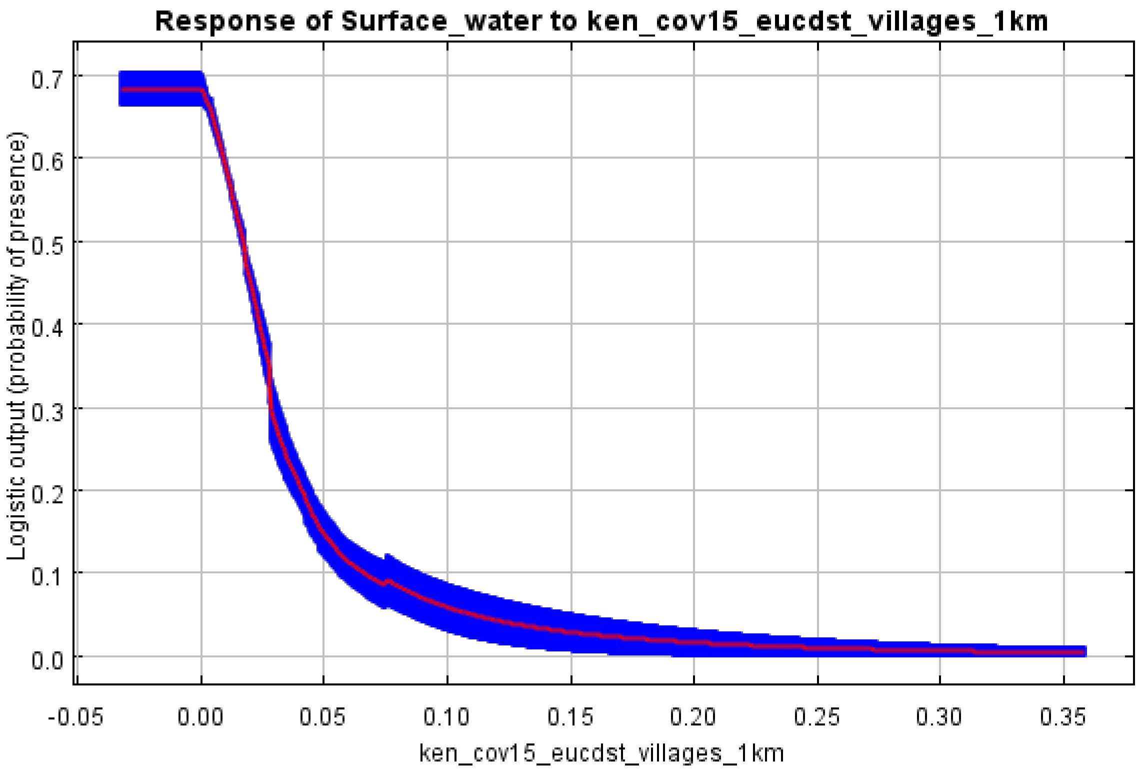

S1_Fig.96. Response curve of Euclidean distance to villages presented as means (red) of 50 replicate runs with standard deviation in blue; model built without other predictive covariates. X-axis: Euclidean distance (decimal degrees).

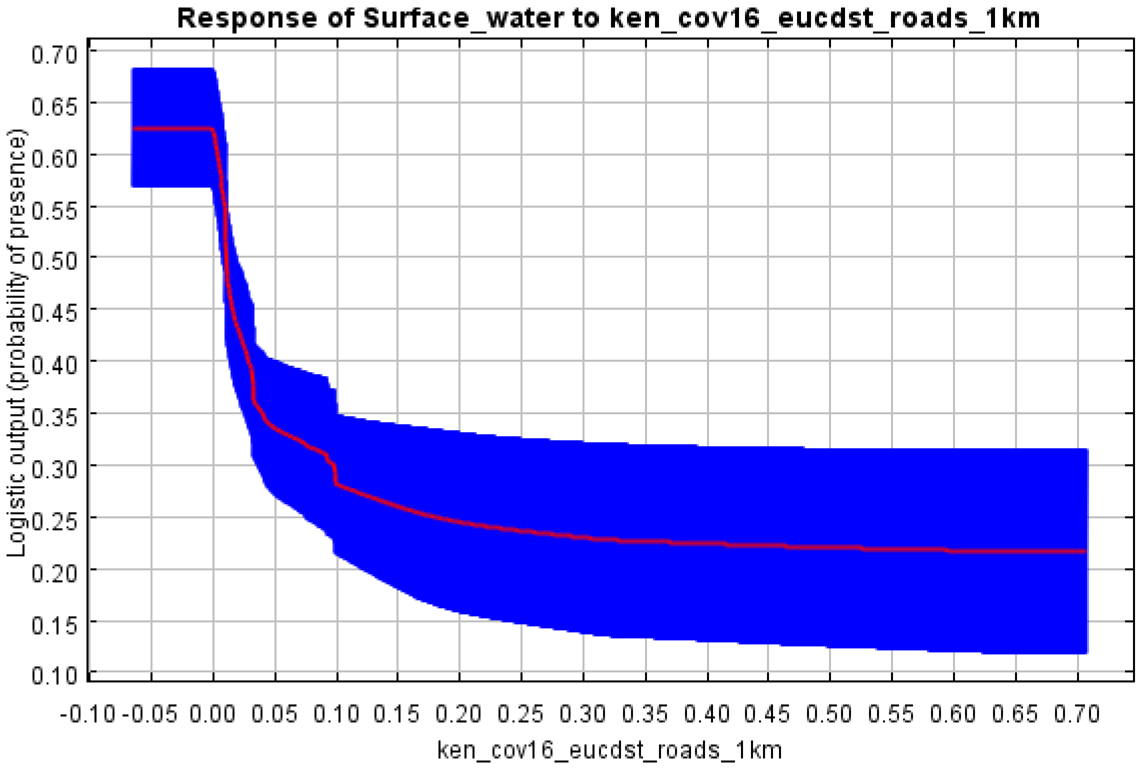

S1_Fig.97. Response curve of Euclidean distance to roads presented as means (red) of 50 replicate runs with standard deviation in blue; model built with other predictive covariates being kept at their average sample values. X-axis: Euclidean distance (decimal degrees).

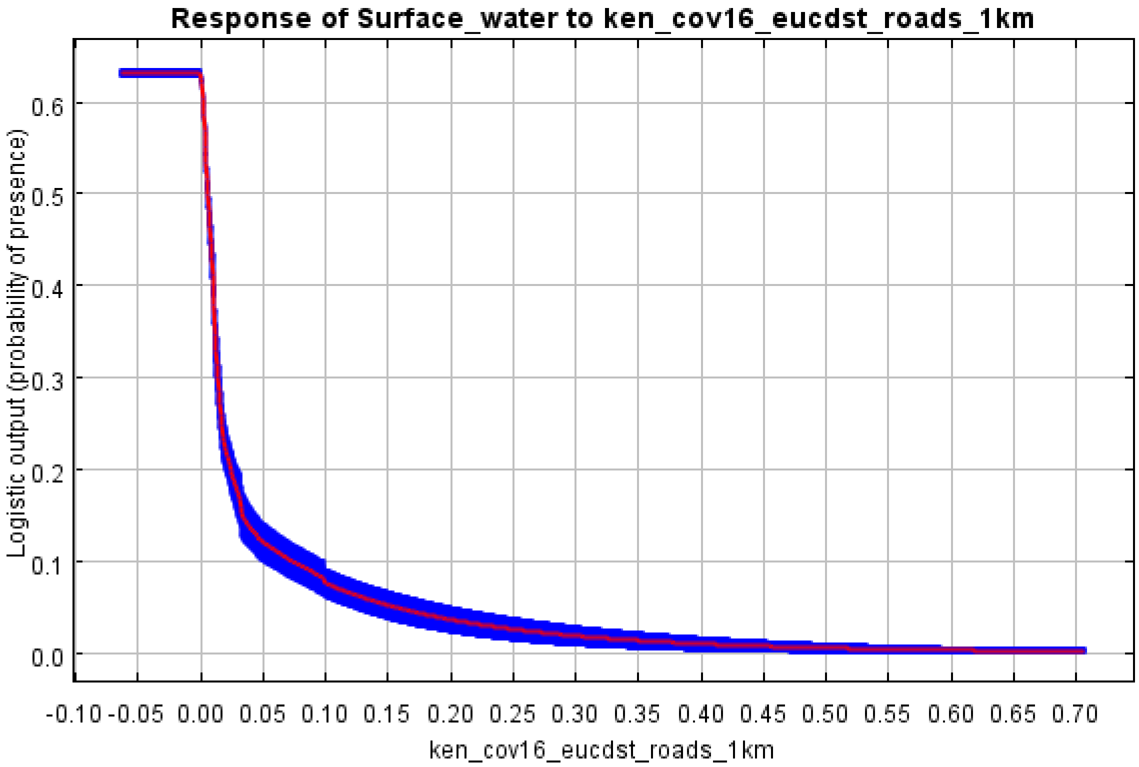

S1_Fig.98. Response curve of Euclidean distance to roads presented as means (red) of 50 replicate runs with standard deviation in blue; model built without other predictive covariates. X-axis: Euclidean distance (decimal degrees).

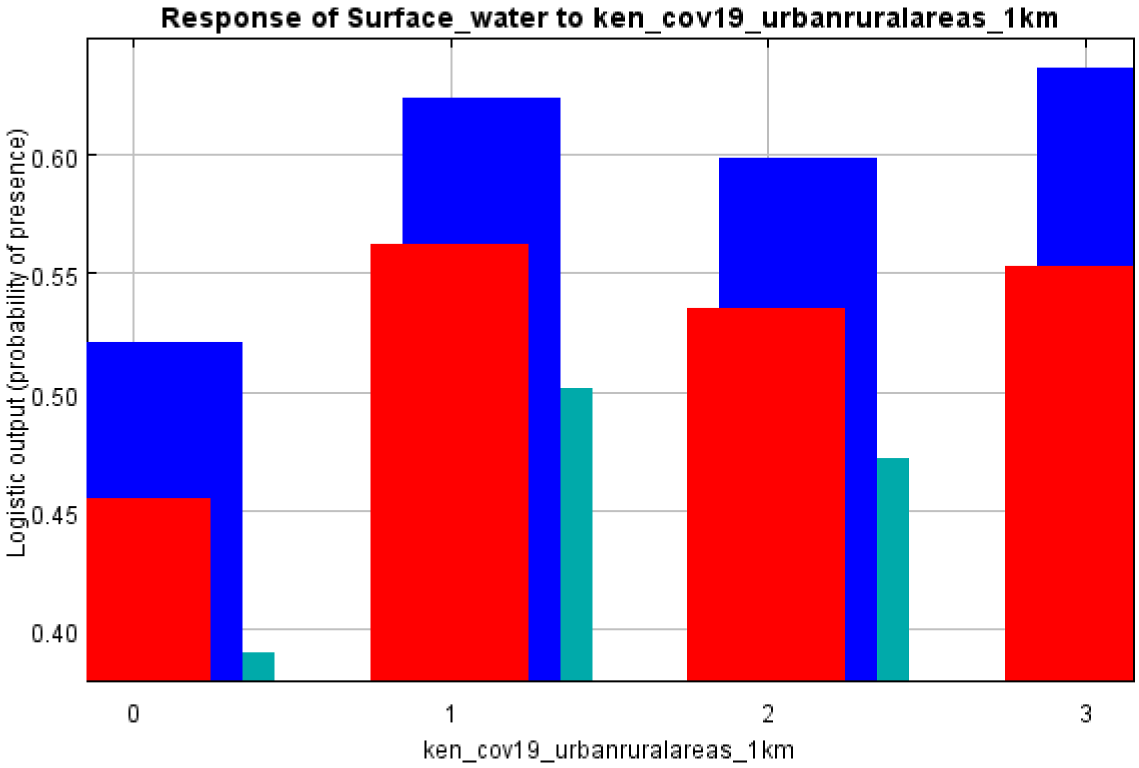

S1_Fig.99. Response curve of rural/urban areas presented as means (red) of 50 replicate runs with standard deviation in blue; model built with other predictive covariates being kept at their average sample values. X-axis: rurality: 0 – Others (not populated/no data); 1 – Rural areas; 2 – Urban clusters; 3 – Urban centres.

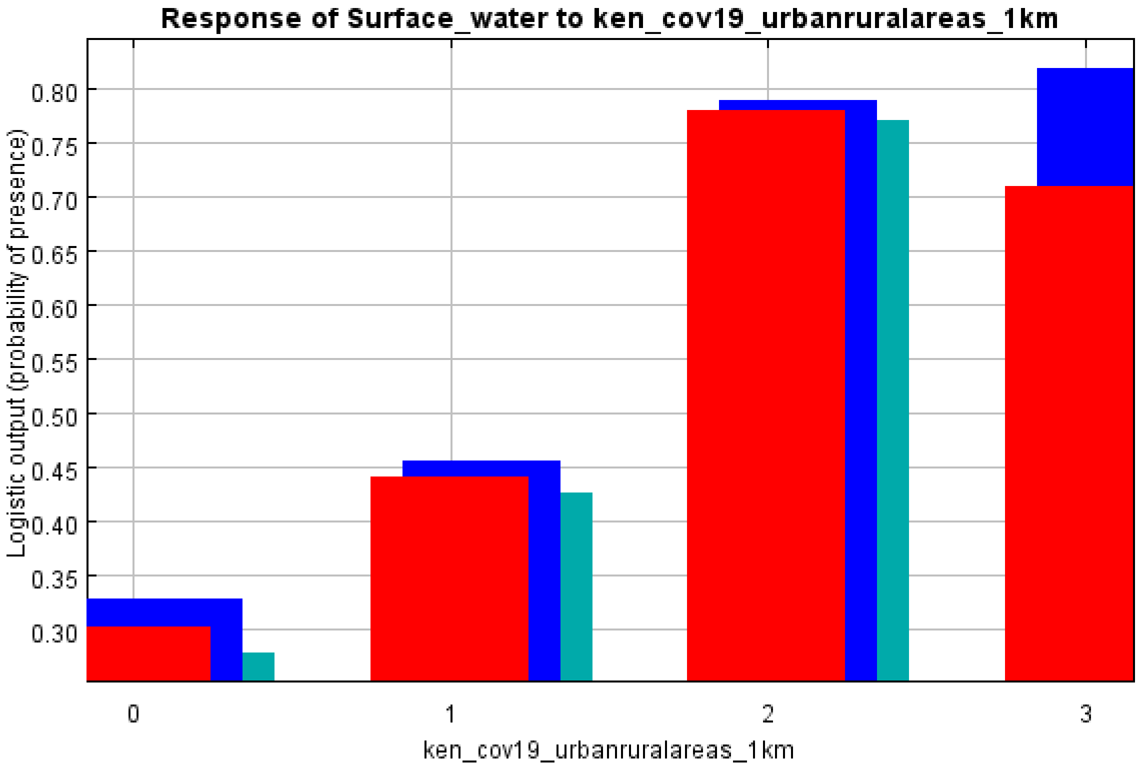

S1_Fig.100. Response curve of rural/urban areas presented as means (red) of 50 replicate runs with standard deviation in blue; model built without other predictive covariates. X-axis: rurality: 0 – Others (not populated/no data); 1 – Rural areas; 2 – Urban clusters; 3 – Urban centres.

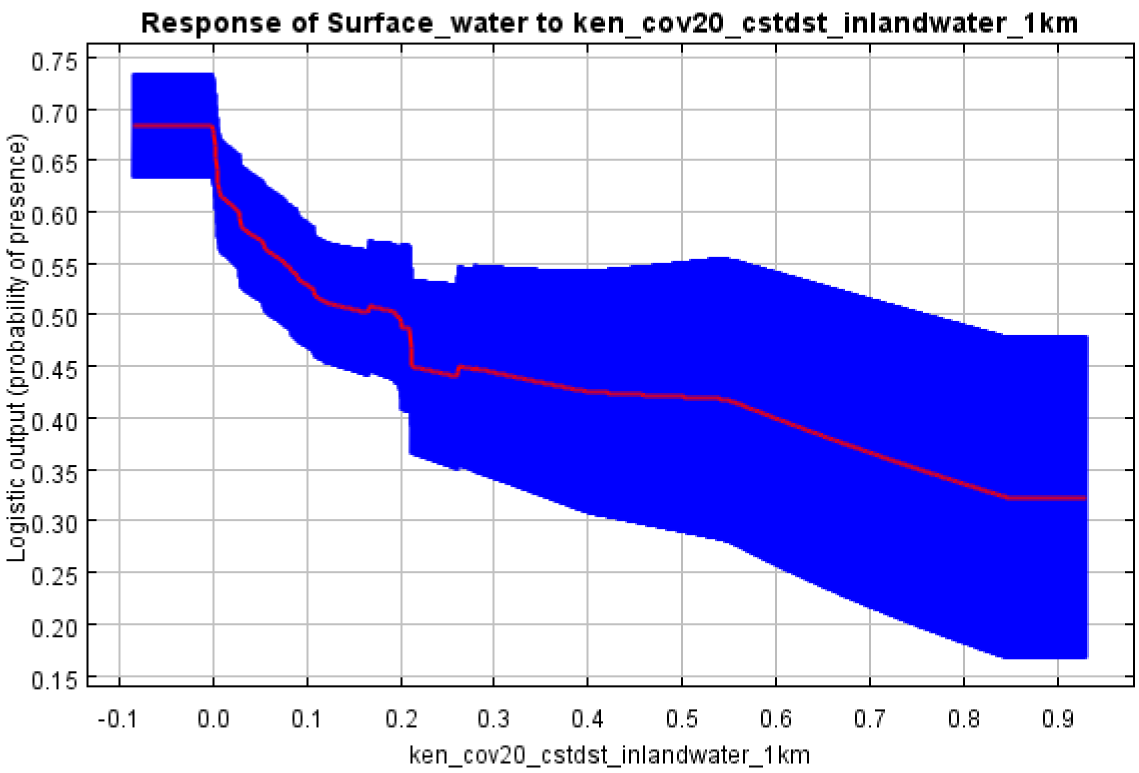

S1_Fig.101. Response curve of cost distance to inland water presented as means (red) of 50 replicate runs with standard deviation in blue; model built with other predictive covariates being kept at their average sample values. X-axis: cost distance (decimal degrees).

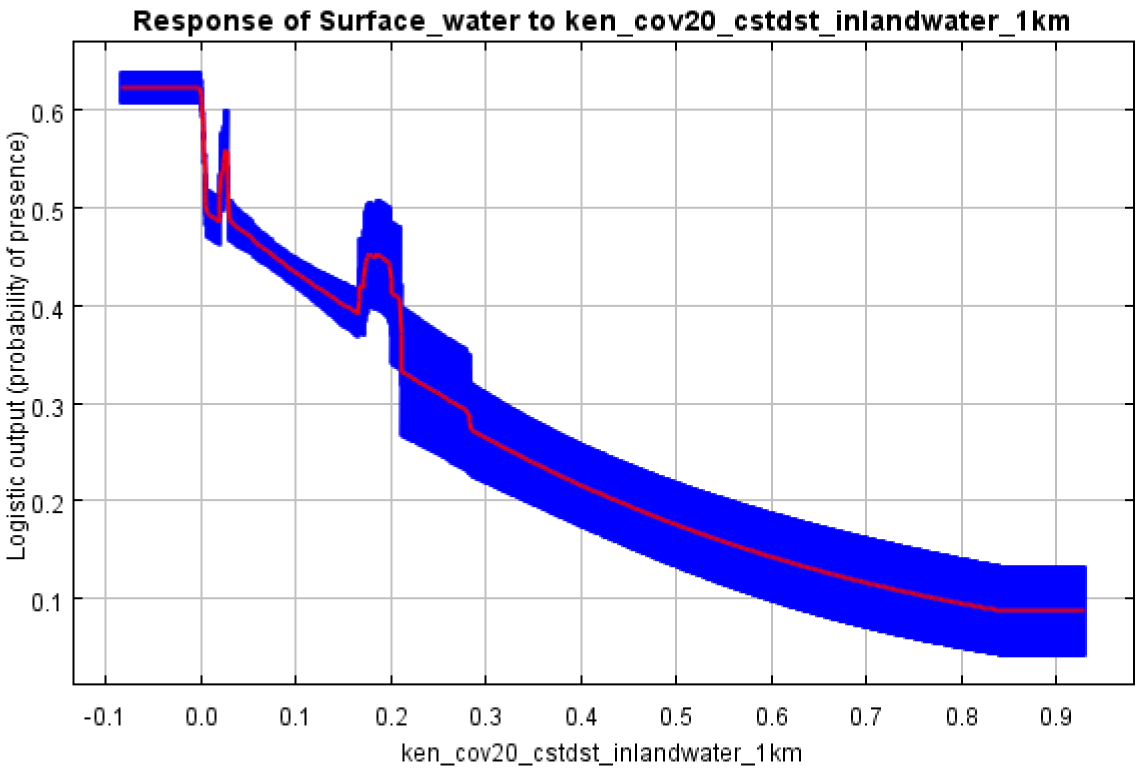

S1_Fig.102. Response curve of cost distance to inland water presented as means (red) of 50 replicate runs with standard deviation in blue; model built without other predictive covariates. X-axis: cost distance (decimal degrees).

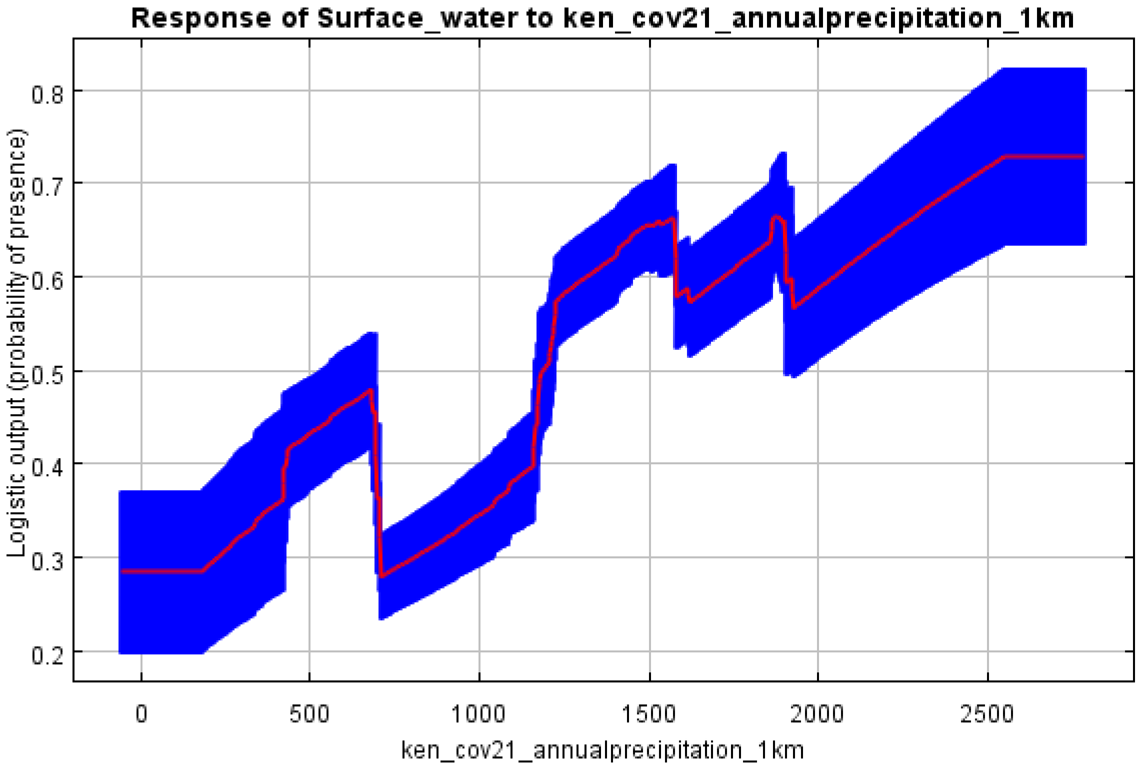

S1_Fig.103. Response curve of annual precipitation presented as means (red) of 50 replicate runs with standard deviation in blue; model built with other predictive covariates being kept at their average sample values. X-axis: annual precipitation (mm).

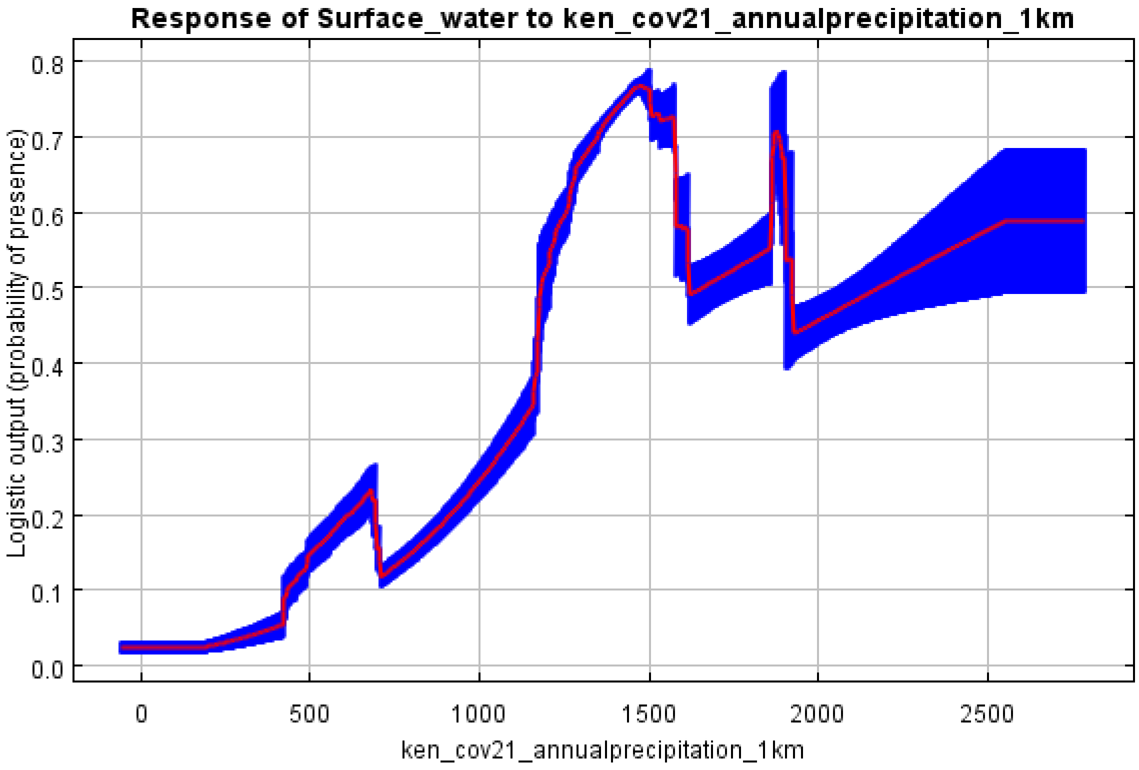

S1_Fig.104. Response curve of annual precipitation to inland water presented as means (red) of 50 replicate runs with standard deviation in blue; model built without other predictive covariates. X-axis: annual precipitation (mm).

Surface water (bias file)

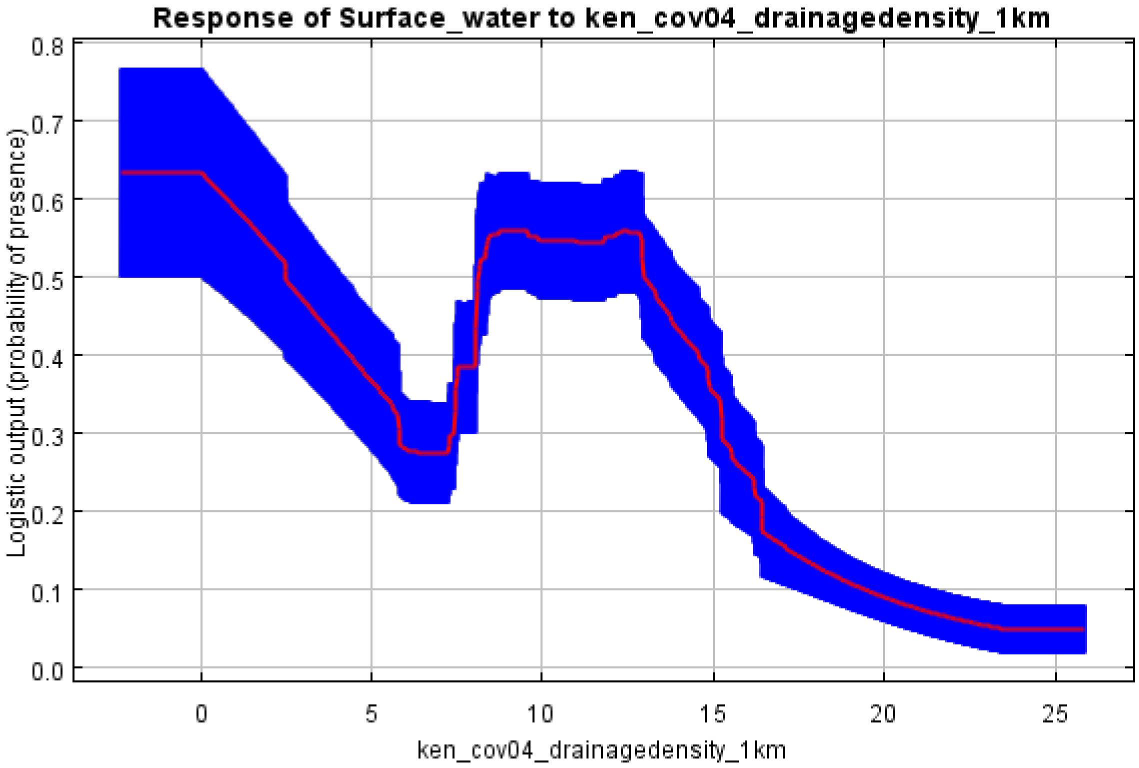

S1_Fig.105. Response curve of drainage density presented as means (red) of 50 replicate runs with standard deviation in blue; model built with other predictive covariates being kept at their average sample values. X-axis: drainage density – channel length per area size of a grid cell (lengths in decimal degrees).

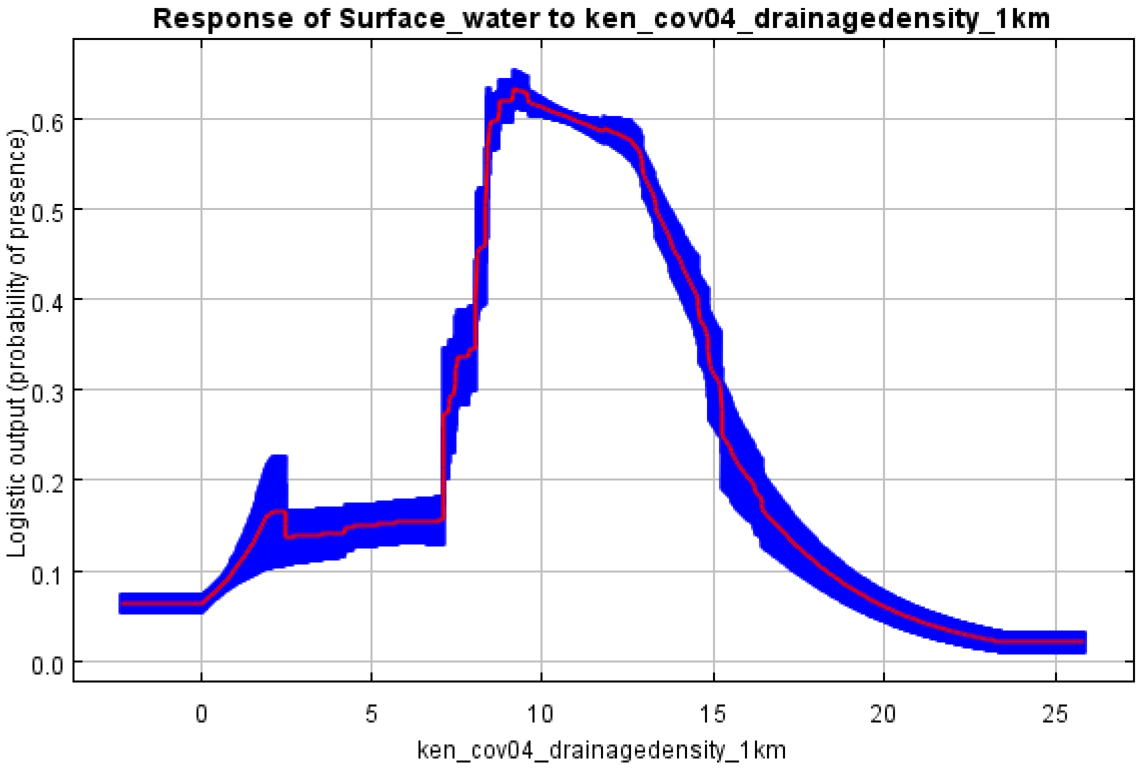

S1_Fig.106. Response curve of drainage density presented as means (red) of 50 replicate runs with standard deviation in blue; model built without other predictive covariates. X-axis: drainage density – channel length per area size of a grid cell (lengths in decimal degrees).

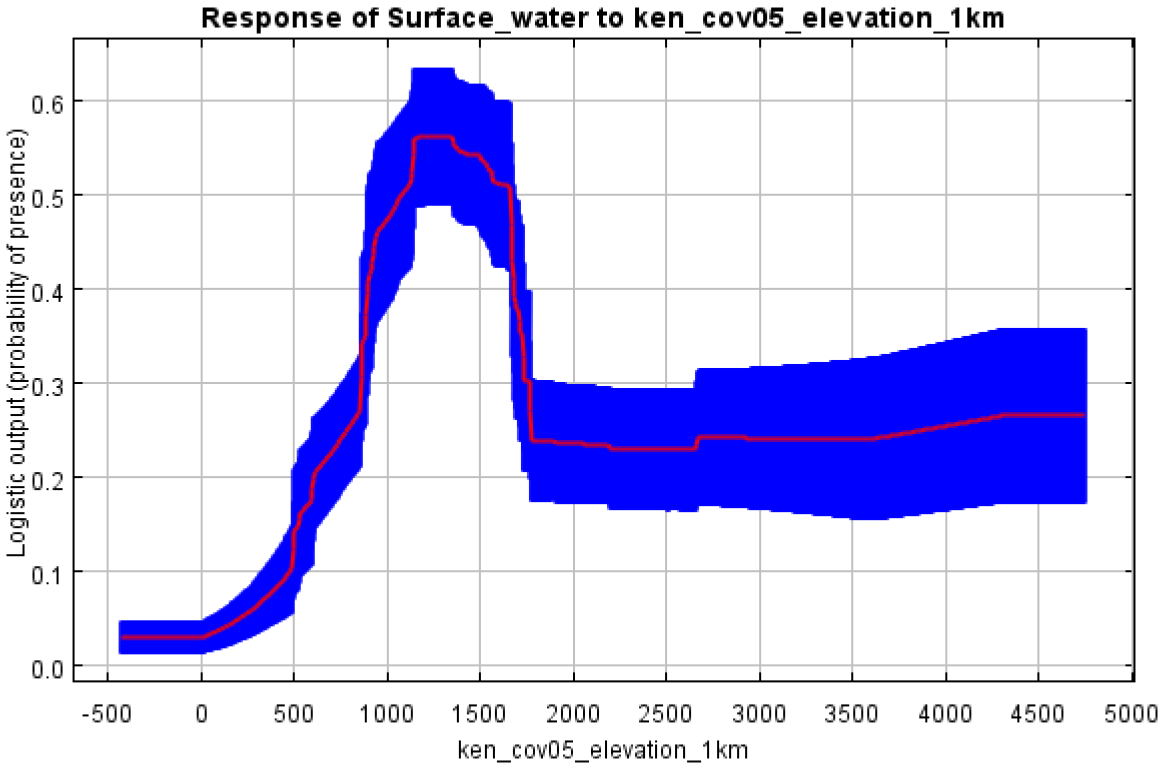

S1_Fig.107. Response curve of elevation presented as means (red) of 50 replicate runs with standard deviation in blue; model built with other predictive covariates being kept at their average sample values. X-axis: elevation (m).

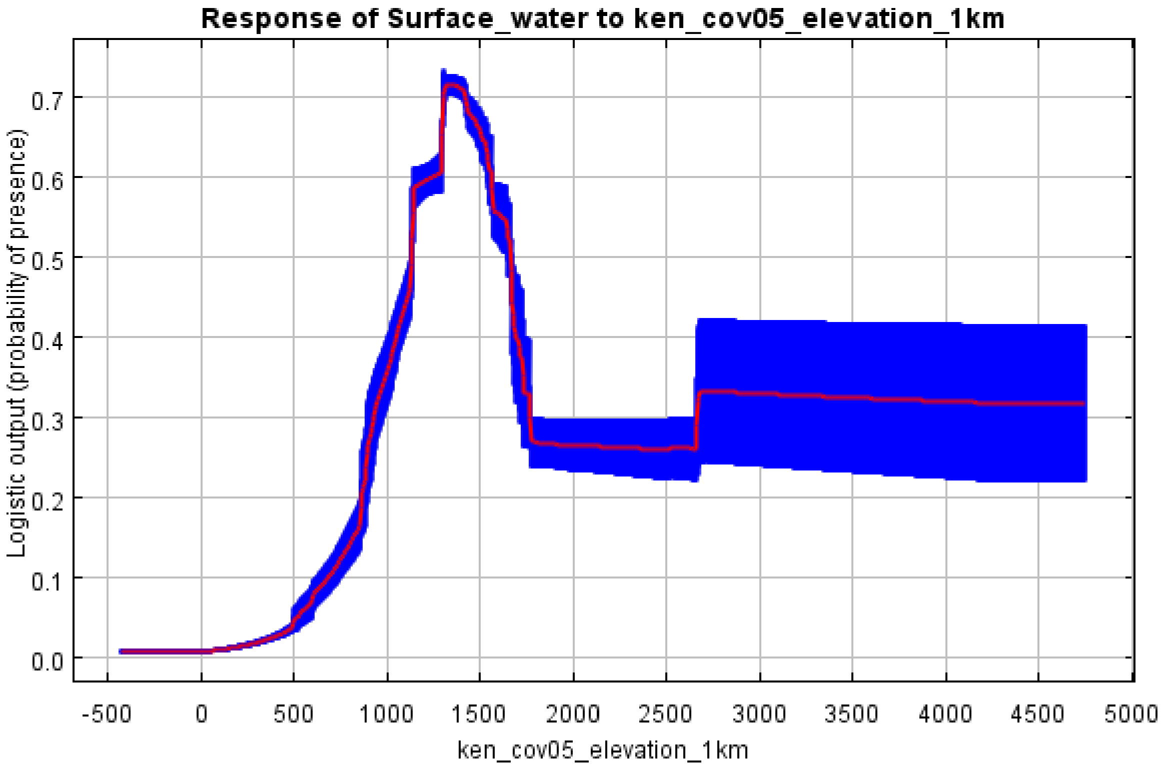

S1_Fig.108. Response curve of elevation presented as means (red) of 50 replicate runs with standard deviation in blue; model built without other predictive covariates. X-axis: elevation (m).

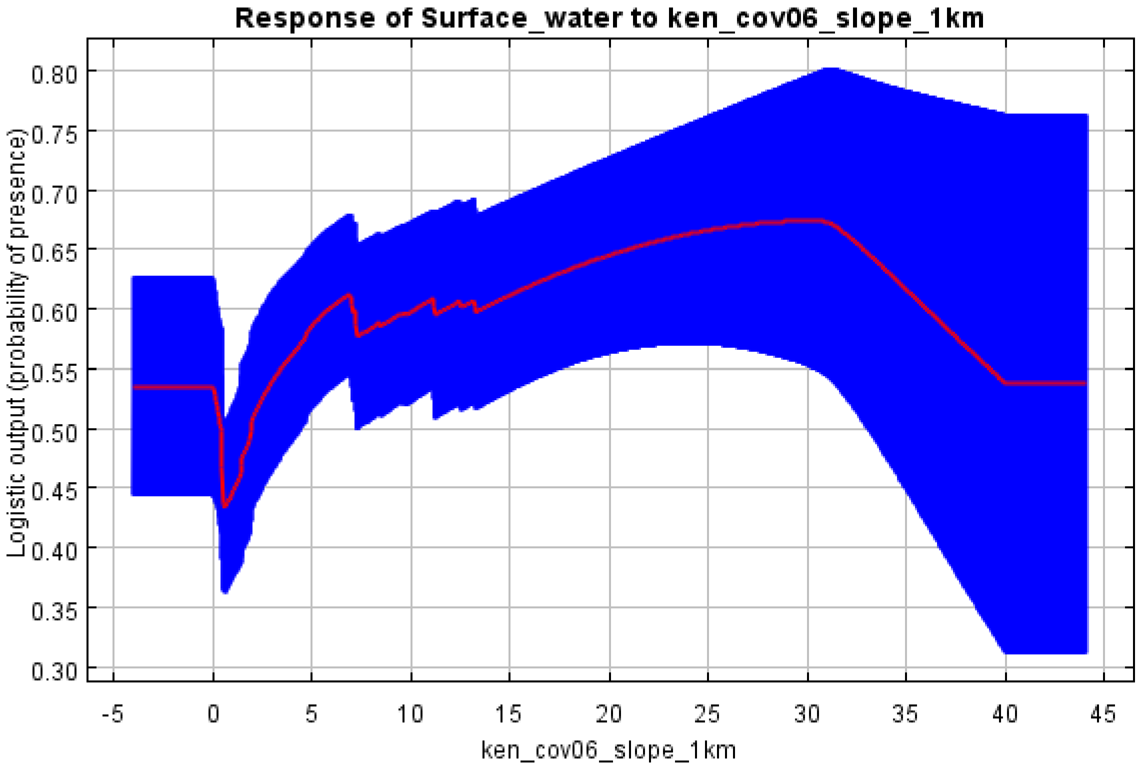

S1_Fig.109. Response curve of slope presented as means (red) of 50 replicate runs with standard deviation in blue; model built with other predictive covariates being kept at their average sample values. X-axis: slope (degree).

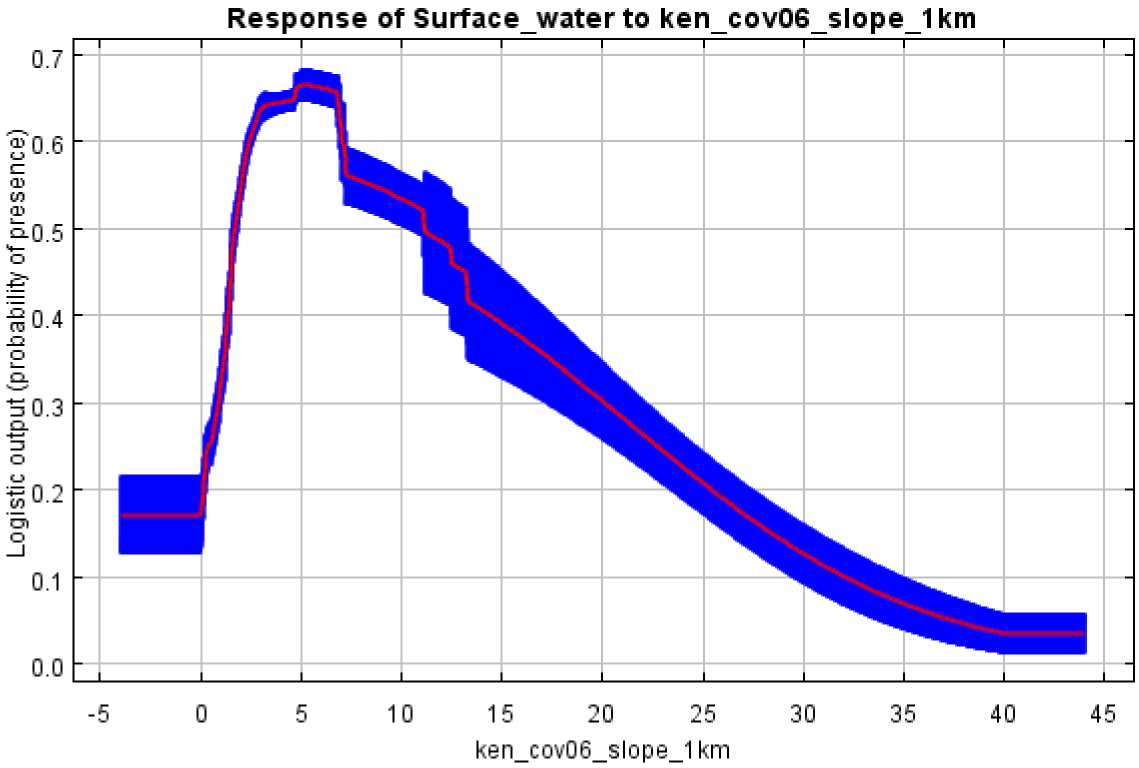

S1_Fig.110. Response curve of slope presented as means (red) of 50 replicate runs with standard deviation in blue; model built without other predictive covariates. X-axis: slope (degree).

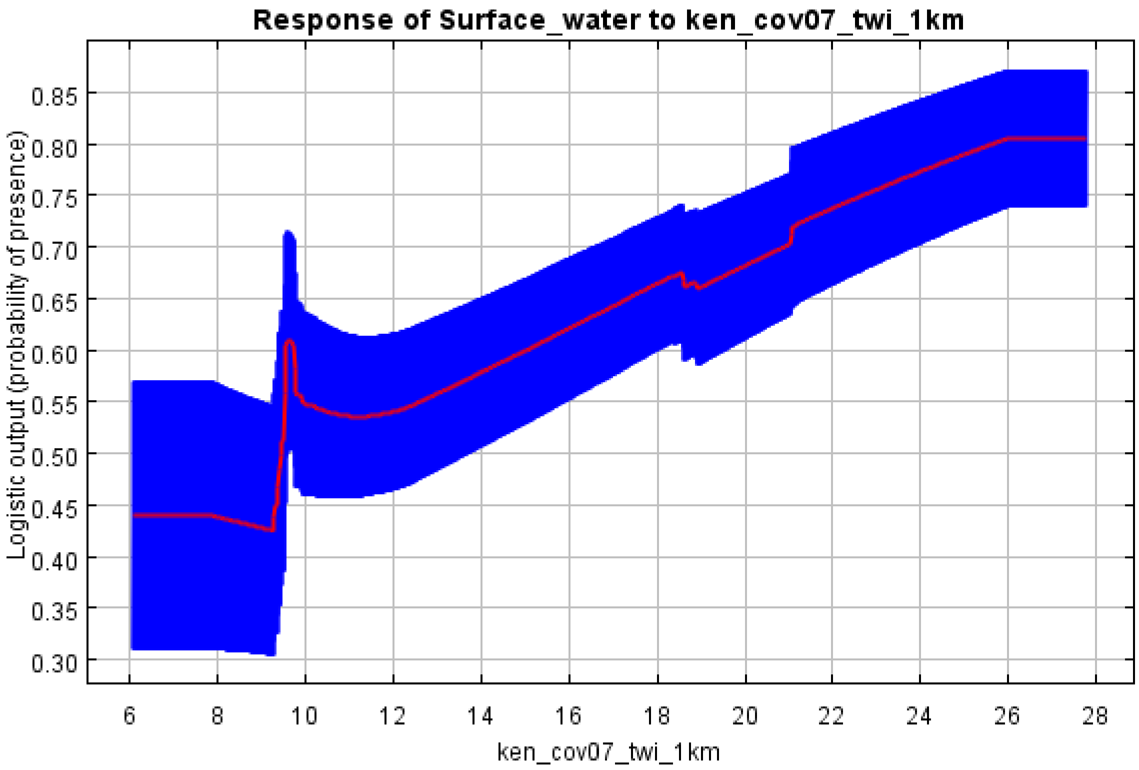

S1_Fig.111. Response curve of topographic wetness index presented as means (red) of 50 replicate runs with standard deviation in blue; model built with other predictive covariates being kept at their average sample values. X-axis: topographic unit index.

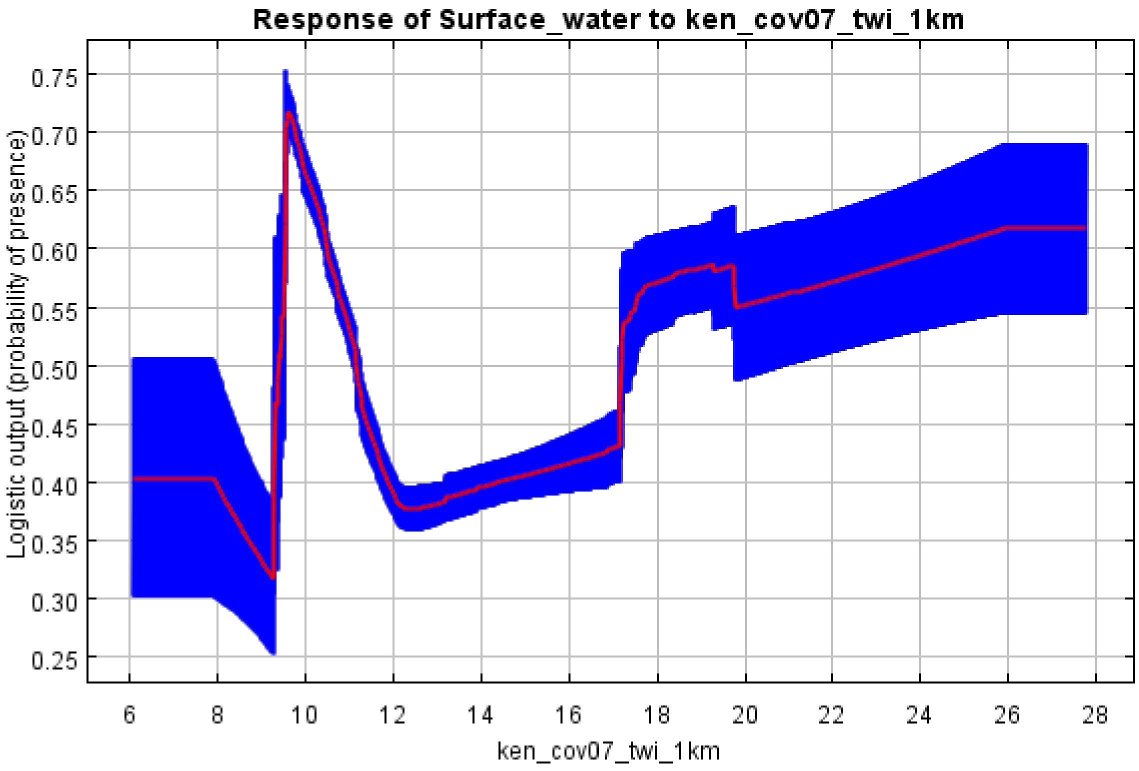

S1_Fig.112. Response curve of topographic wetness index presented as means (red) of 50 replicate runs with standard deviation in blue; model built without other predictive covariates. X-axis: topographic unit index.

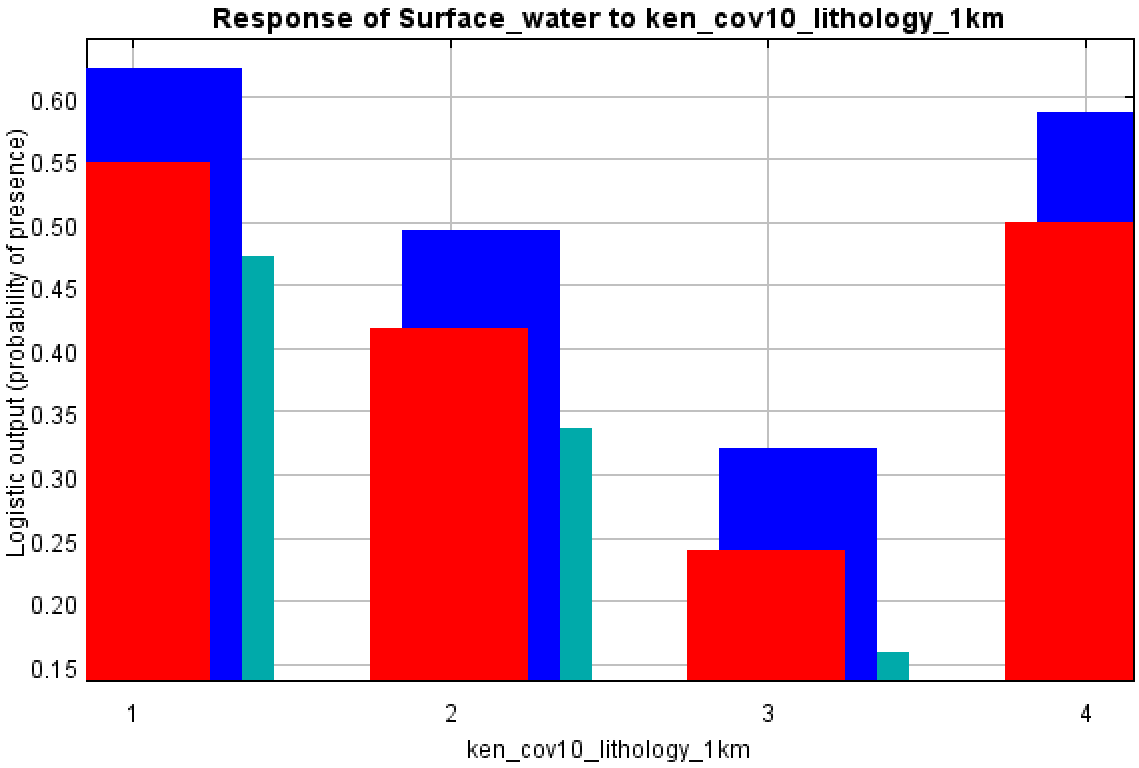

S1_Fig.113. Response curve of lithology presented as means (red) of 50 replicate runs with standard deviation in blue; model built with other predictive covariates being kept at their average sample values. X-axis: lithology: 1 – Igneous; 2 – Metamorphic rock; 3 – Sedimentary rock; 4 – Unconsolidated.

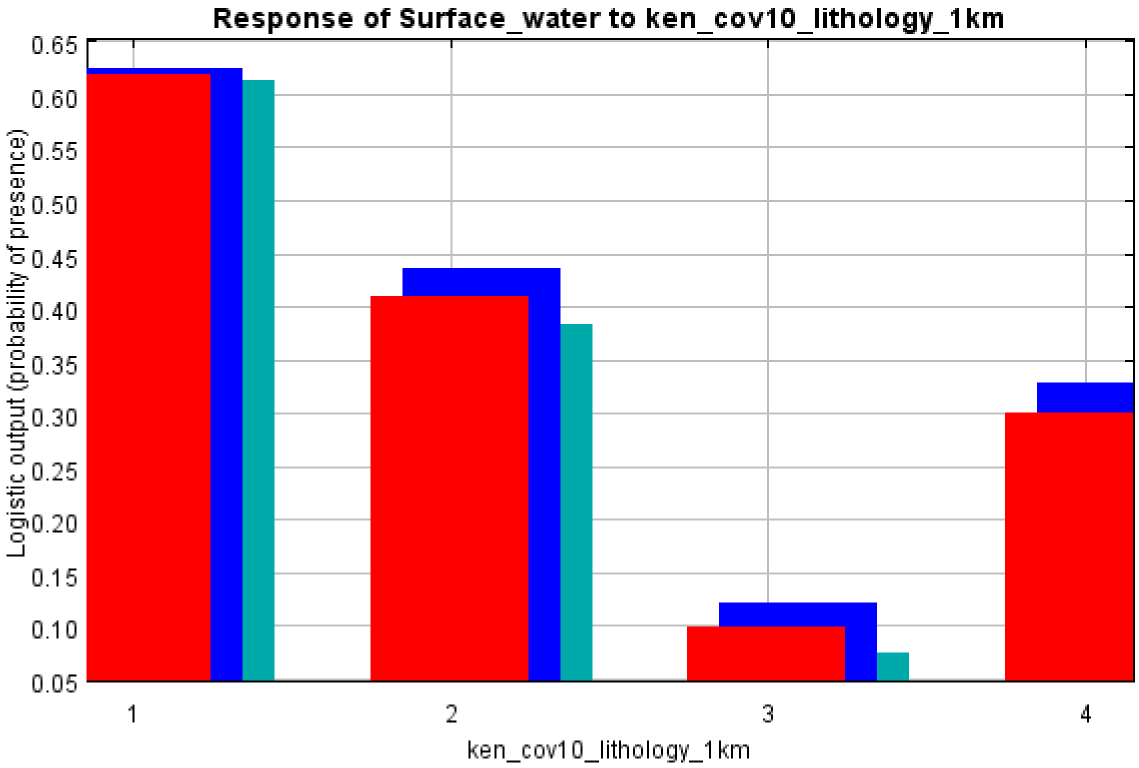

S1_Fig.114. Response curve of lithology presented as means (red) of 50 replicate runs with standard deviation in blue; model built without other predictive covariates. X-axis: lithology: 1 – Igneous; 2 – Metamorphic rock; 3 – Sedimentary rock; 4 – Unconsolidated.

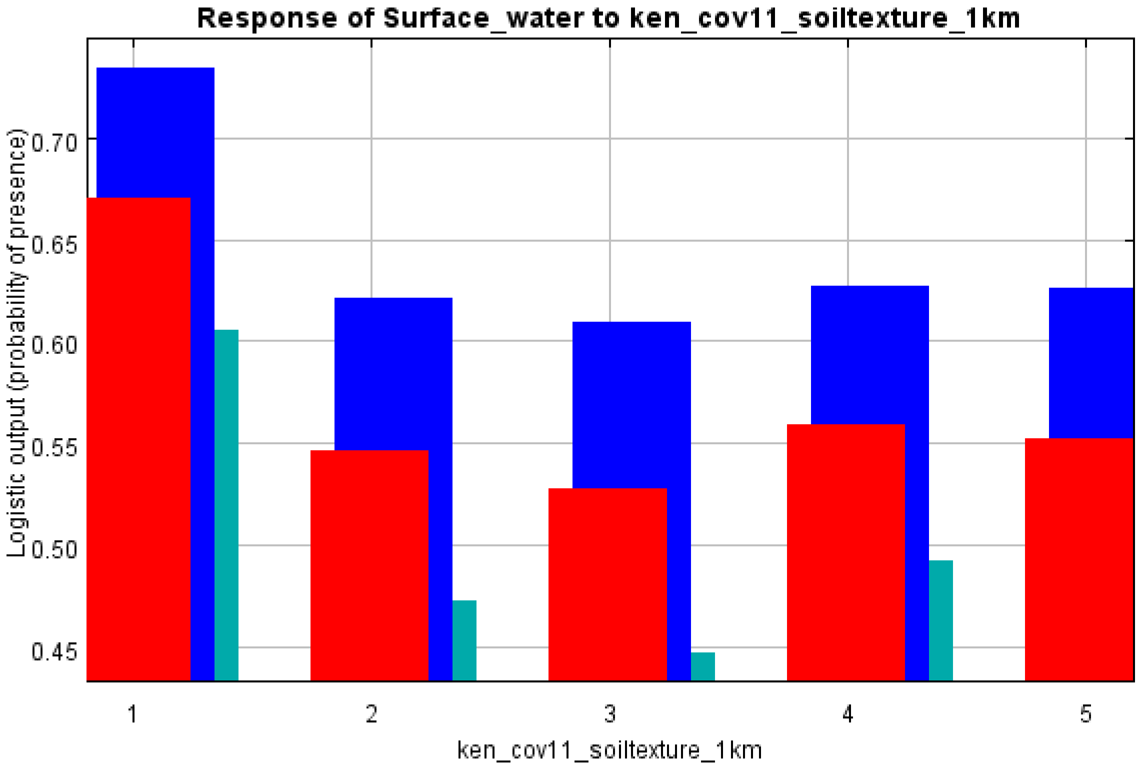

S1_Fig.115. Response curve of soil texture presented as means (red) of 50 replicate runs with standard deviation in blue; model built with other predictive covariates being kept at their average sample values. X-axis: lithology: 1 – Very clayey; 2 – Clayey; 3 – Loamy; 4 – Sandy; 5 – Extremely sandy.

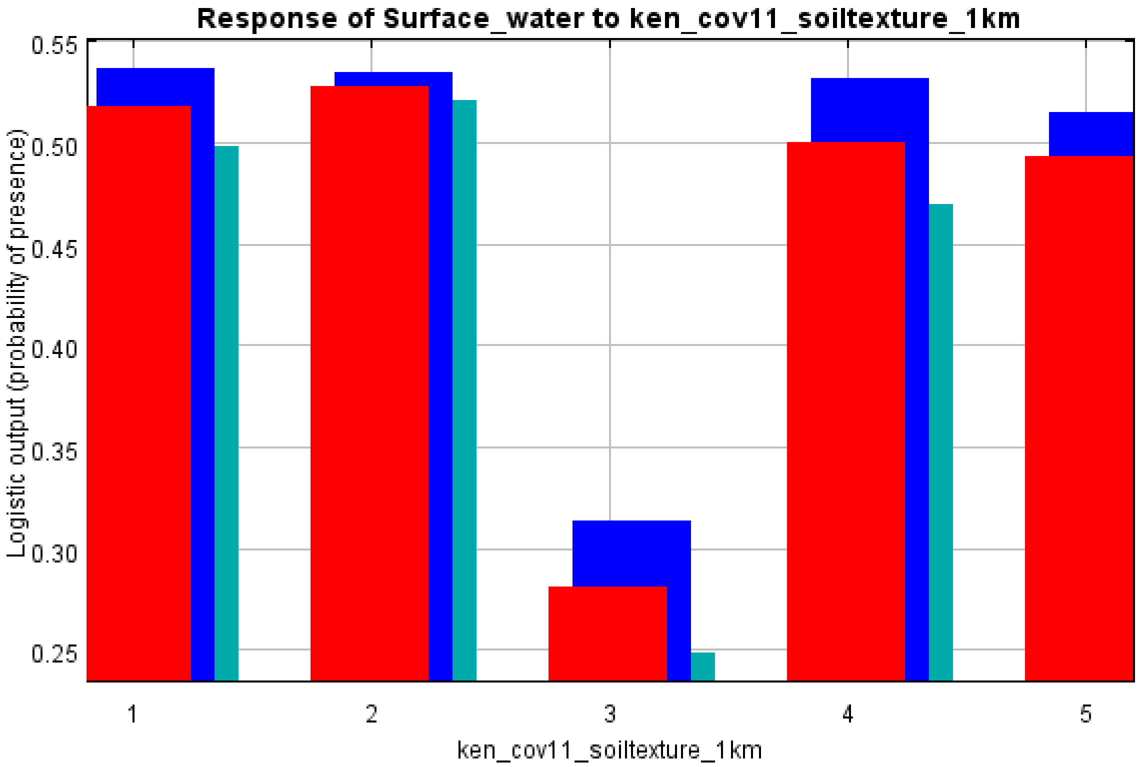

S1_Fig.116. Response curve of soil texture presented as means (red) of 50 replicate runs with standard deviation in blue; model built without other predictive covariates. X-axis: lithology: 1 – Very clayey; 2 – Clayey; 3 – Loamy; 4 – Sandy; 5 – Extremely sandy.

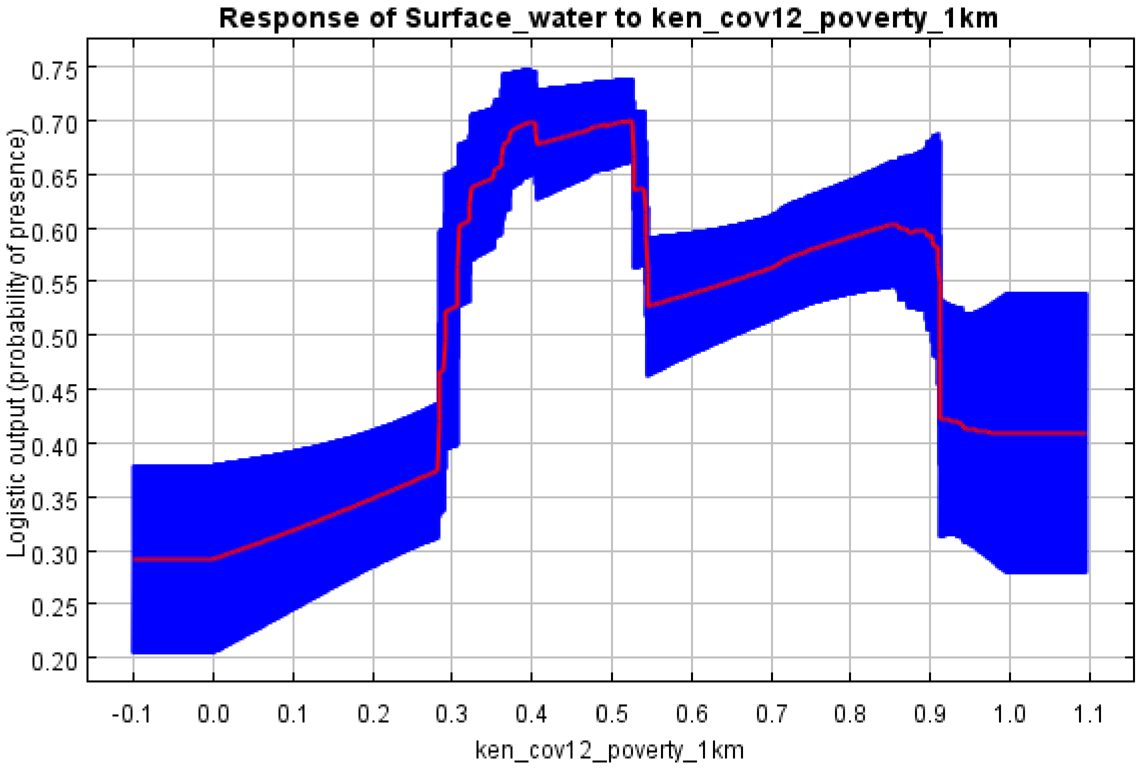

S1_Fig.117. Response curve of poverty presented as means (red) of 50 replicate runs with standard deviation in blue; model built with other predictive covariates being kept at their average sample values. X-axis: proportion of residents living in MPI-defined poverty.

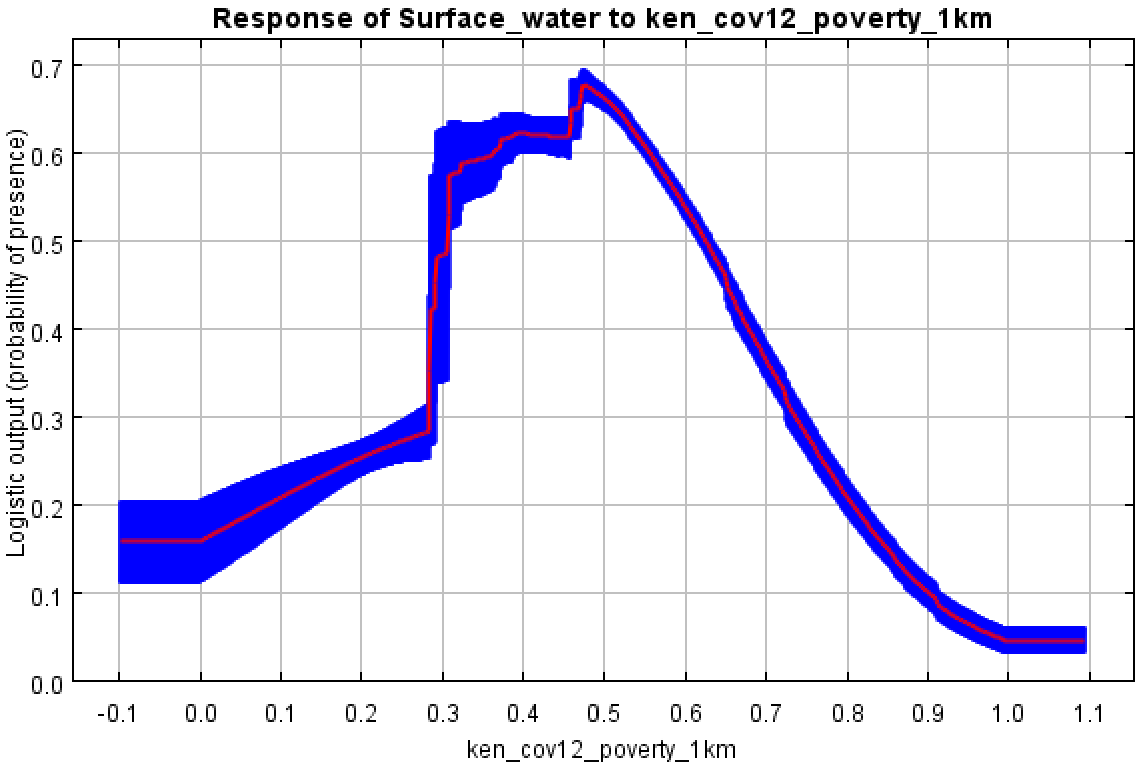

S1_Fig.118. Response curve of poverty presented as means (red) of 50 replicate runs with standard deviation in blue; model built without other predictive covariates. X-axis: proportion of residents living in MPI-defined poverty.

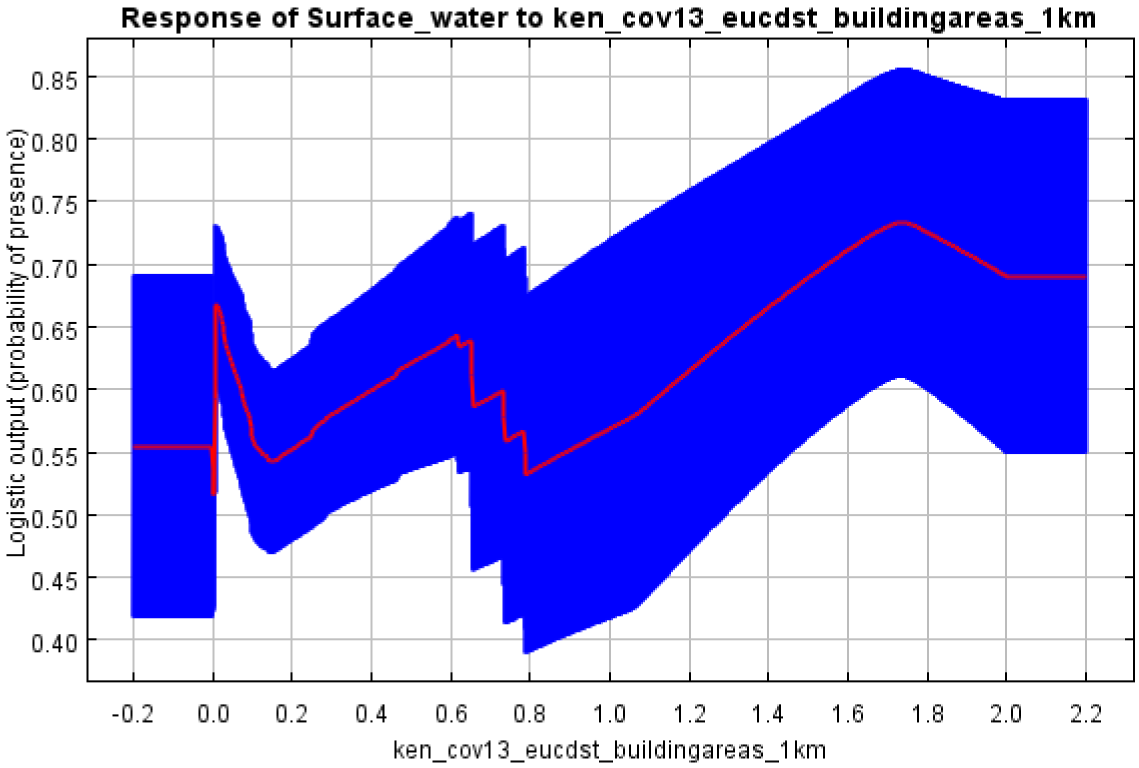

S1_Fig.119. Response curve of Euclidean distance to buildings presented as means (red) of 50 replicate runs with standard deviation in blue; model built with other predictive covariates being kept at their average sample values. X-axis: Euclidean distance (decimal degrees).

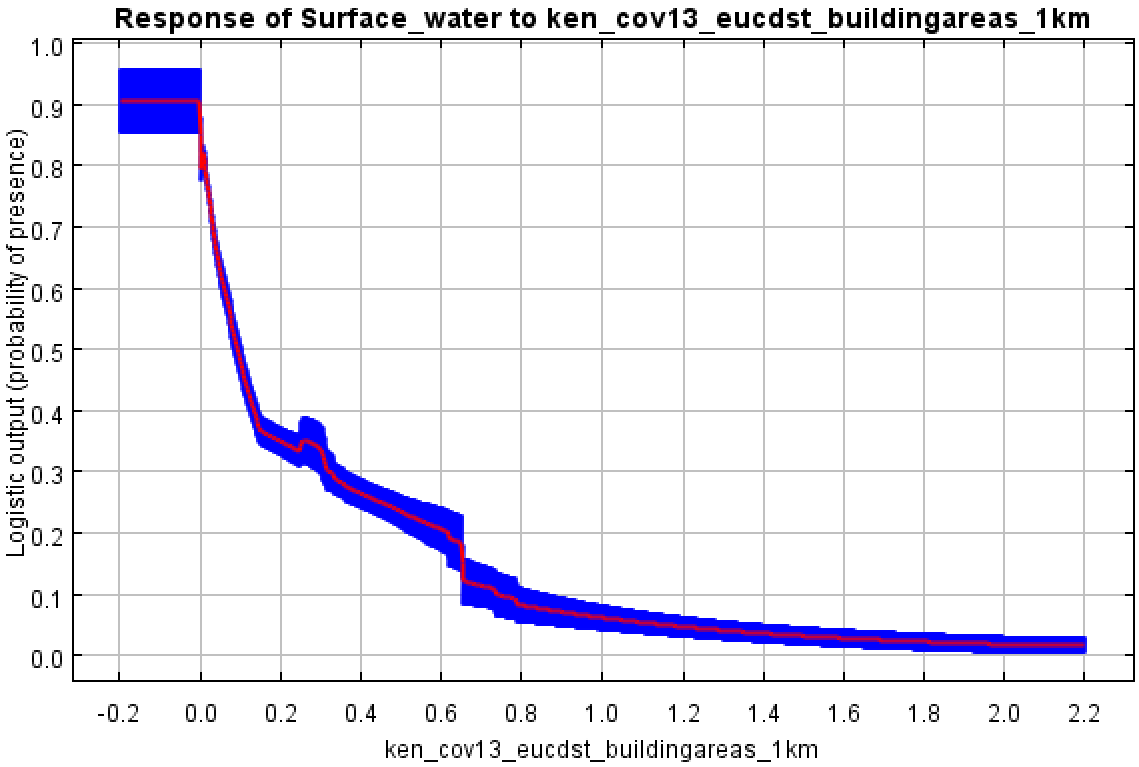

S1_Fig.120. Response curve of Euclidean distance to buildings presented as means (red) of 50 replicate runs with standard deviation in blue; model built without other predictive covariates. X-axis: Euclidean distance (decimal degrees).

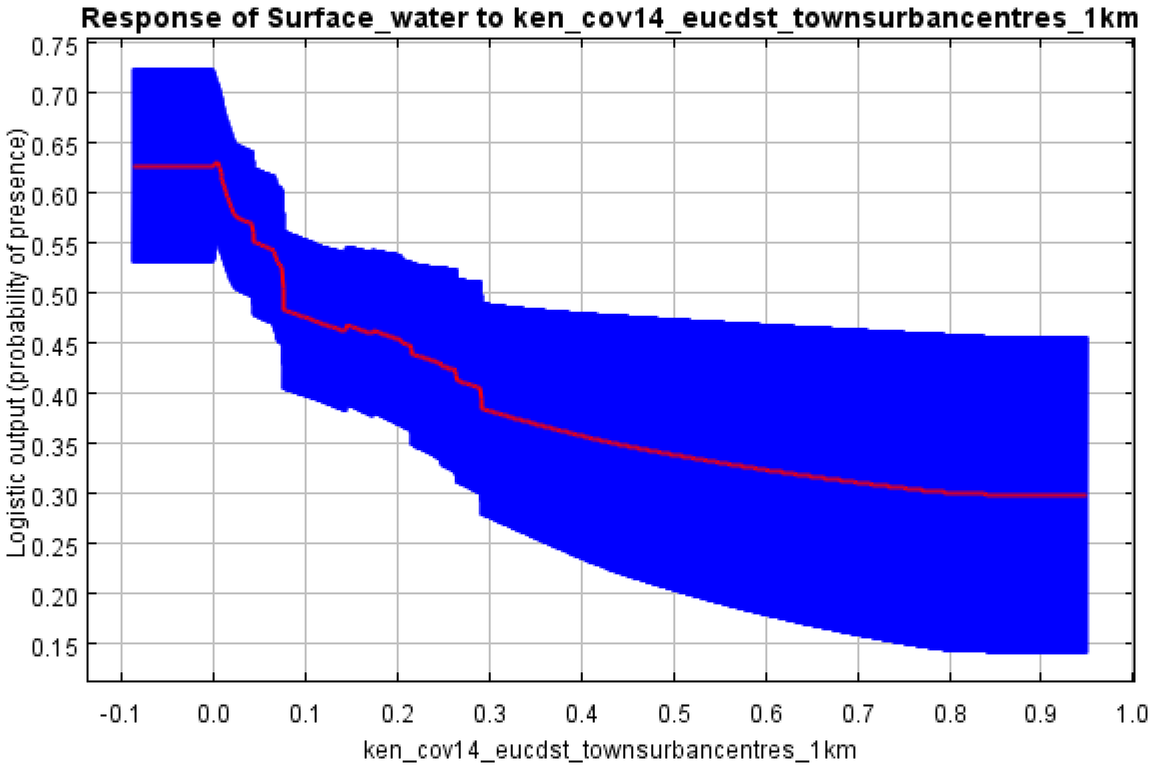

S1_Fig.121. Response curve of Euclidean distance to towns/urban centres presented as means (red) of 50 replicate runs with standard deviation in blue; model built with other predictive covariates being kept at their average sample values. X-axis: Euclidean distance (decimal degrees).

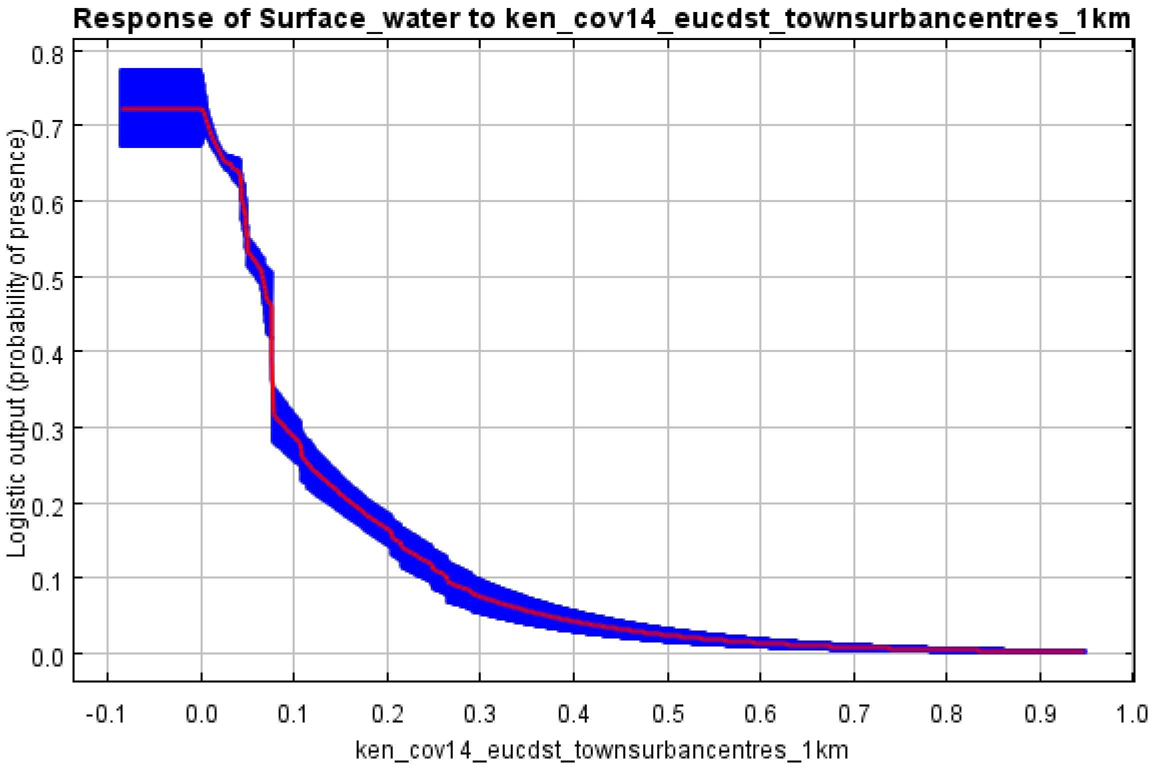

S1_Fig.122. Response curve of Euclidean distance to towns/urban centres presented as means (red) of 50 replicate runs with standard deviation in blue; model built without other predictive covariates. X-axis: Euclidean distance (decimal degrees).

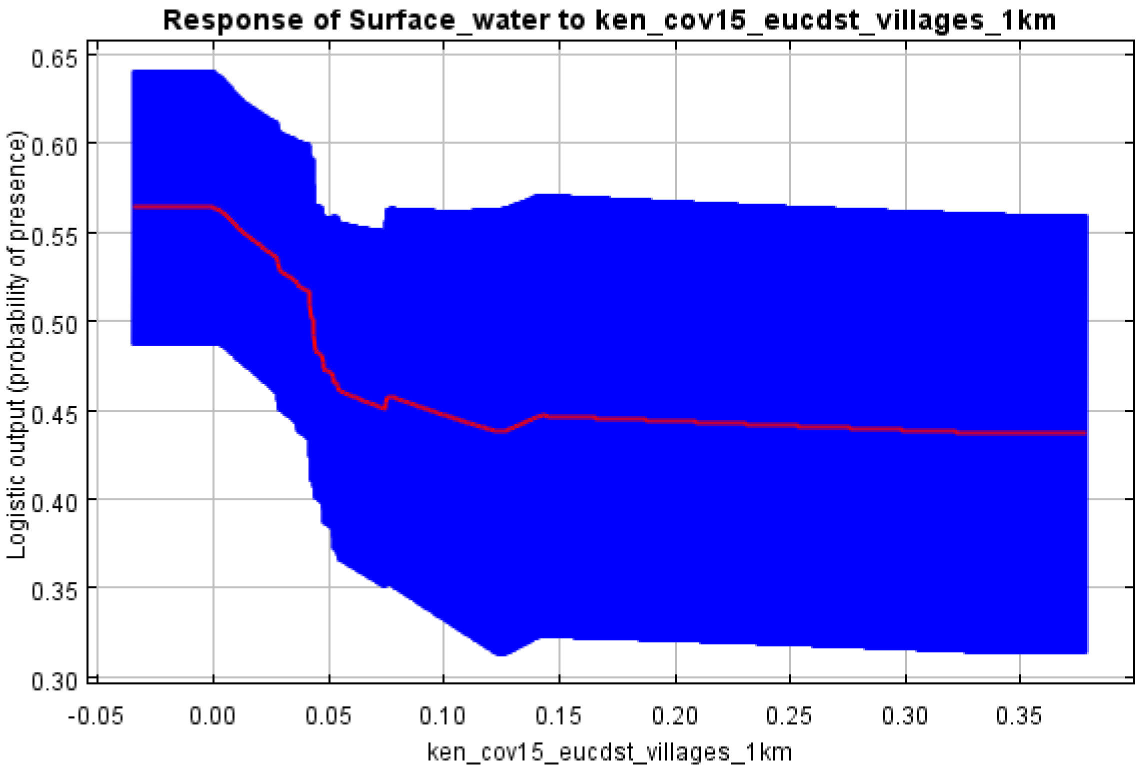

S1_Fig.123. Response curve of Euclidean distance to villages presented as means (red) of 50 replicate runs with standard deviation in blue; model built with other predictive covariates being kept at their average sample values. X-axis: Euclidean distance (decimal degrees).

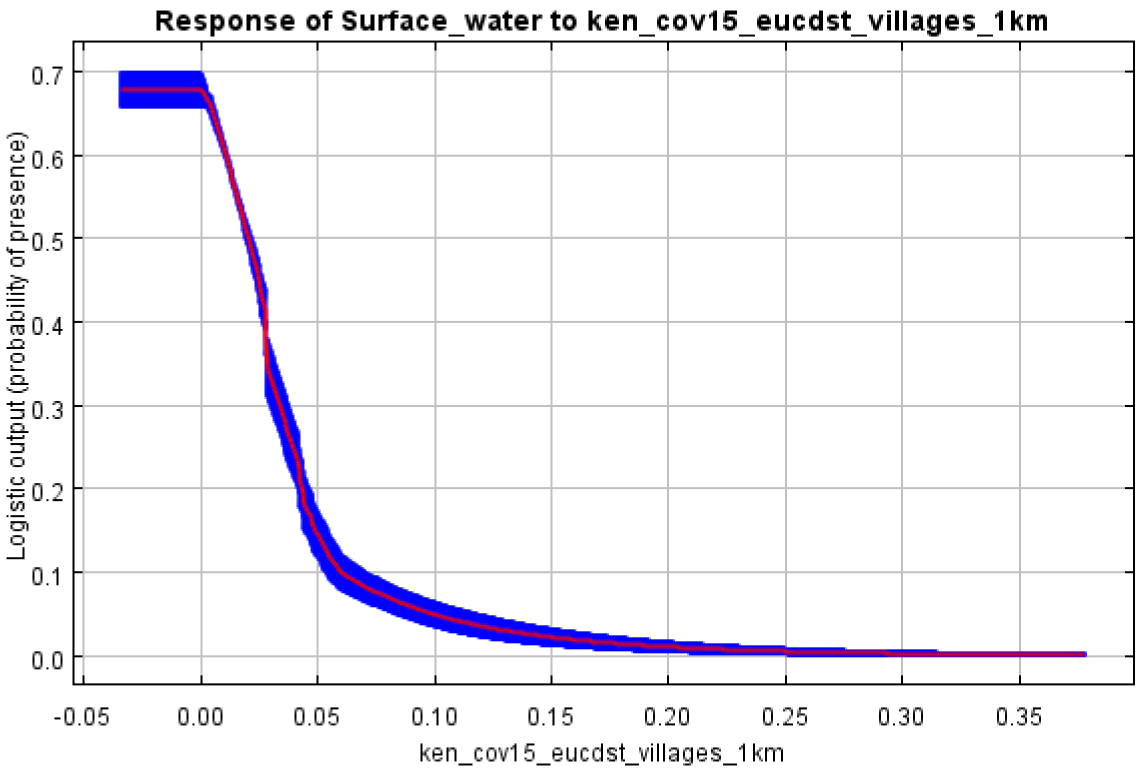

S1_Fig.124. Response curve of Euclidean distance to villages presented as means (red) of 50 replicate runs with standard deviation in blue; model built without other predictive covariates. X-axis: Euclidean distance (decimal degrees).

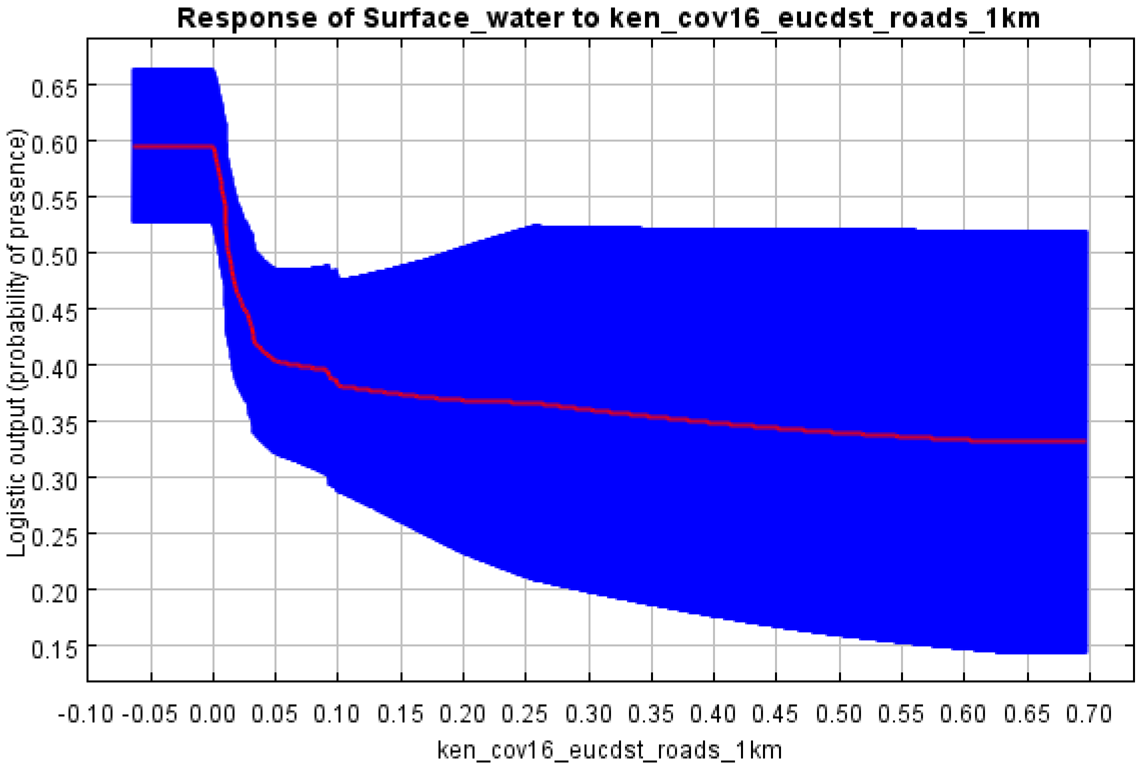

S1_Fig.125. Response curve of Euclidean distance to roads presented as means (red) of 50 replicate runs with standard deviation in blue; model built with other predictive covariates being kept at their average sample values. X-axis: Euclidean distance (decimal degrees).

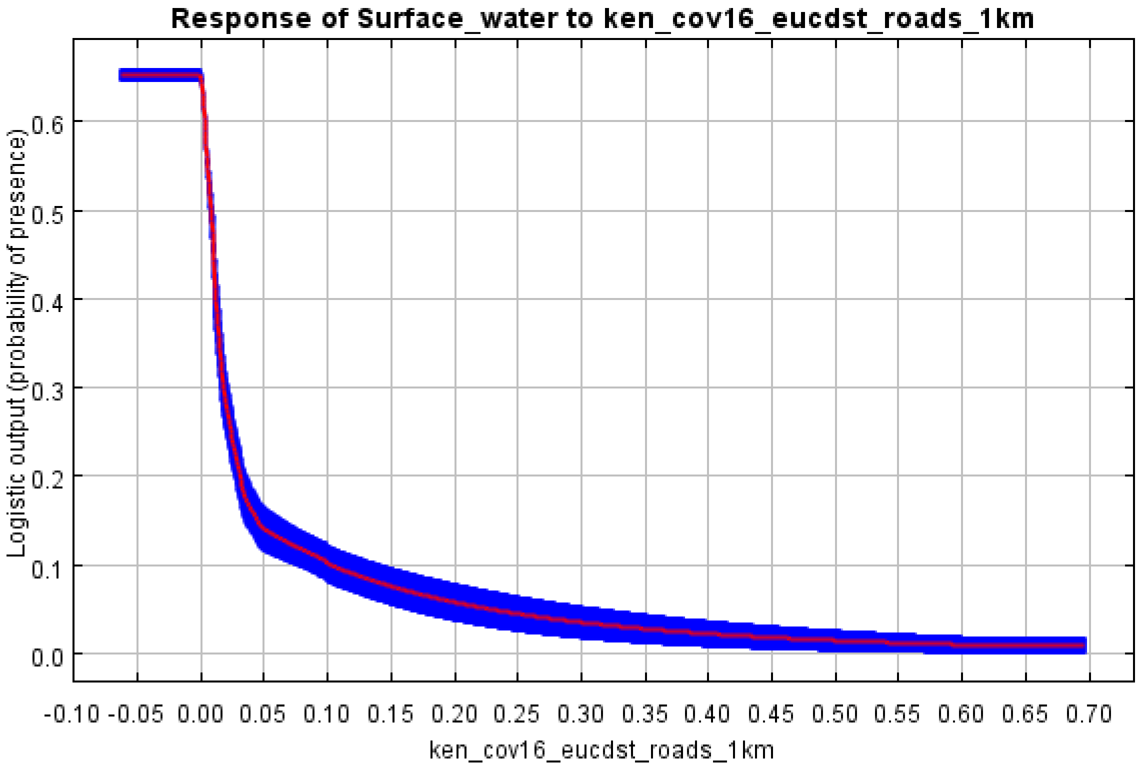

S1_Fig.126. Response curve of Euclidean distance to roads presented as means (red) of 50 replicate runs with standard deviation in blue; model built without other predictive covariates. X-axis: Euclidean distance (decimal degrees).

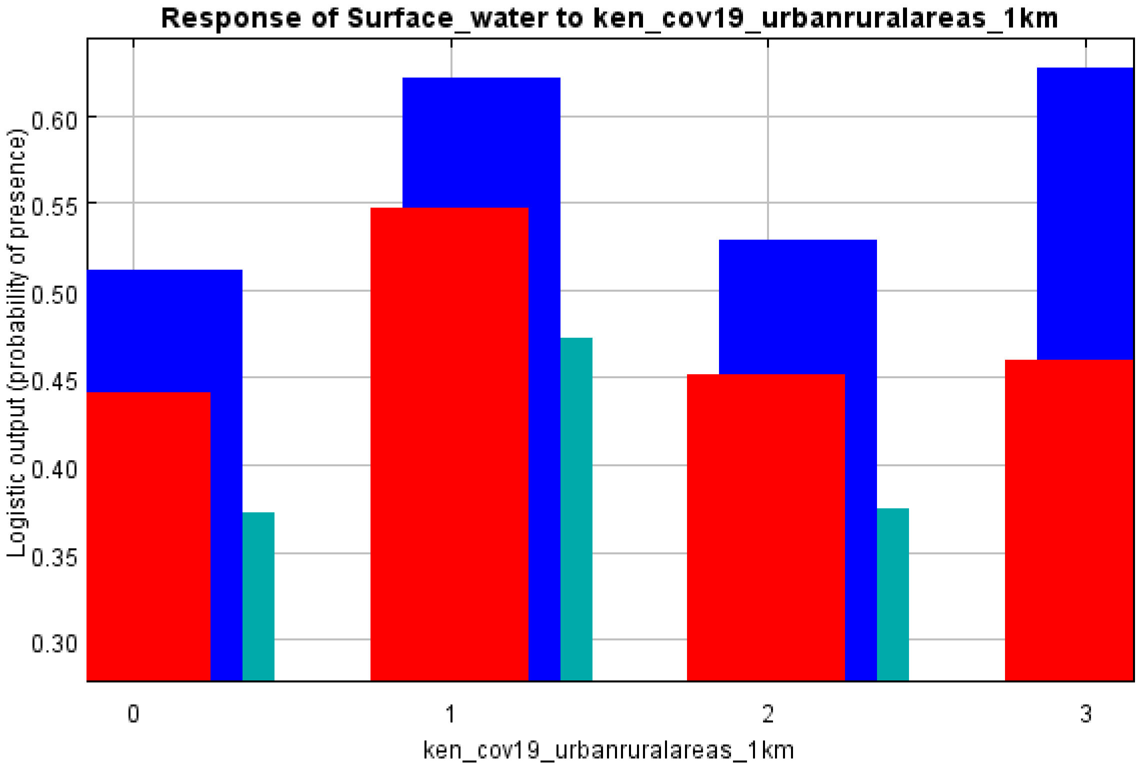

S1_Fig.127. Response curve of rural/urban areas presented as means (red) of 50 replicate runs with standard deviation in blue; model built with other predictive covariates being kept at their average sample values. X-axis: rurality: 0 – Others (not populated/no data); 1 – Rural areas; 2 – Urban clusters; 3 – Urban centres.

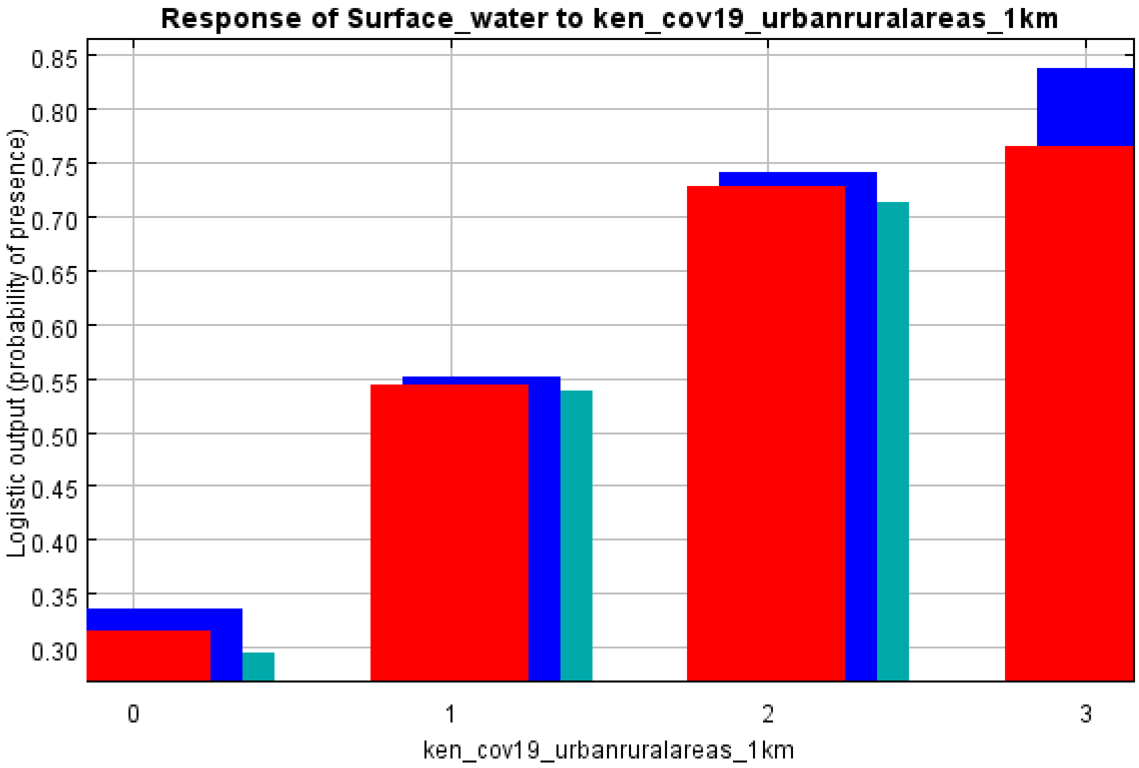

S1_Fig.128. Response curve of rural/urban areas presented as means (red) of 50 replicate runs with standard deviation in blue; model built without other predictive covariates. X-axis: rurality: 0 – Others (not populated/no data); 1 – Rural areas; 2 – Urban clusters; 3 – Urban centres.

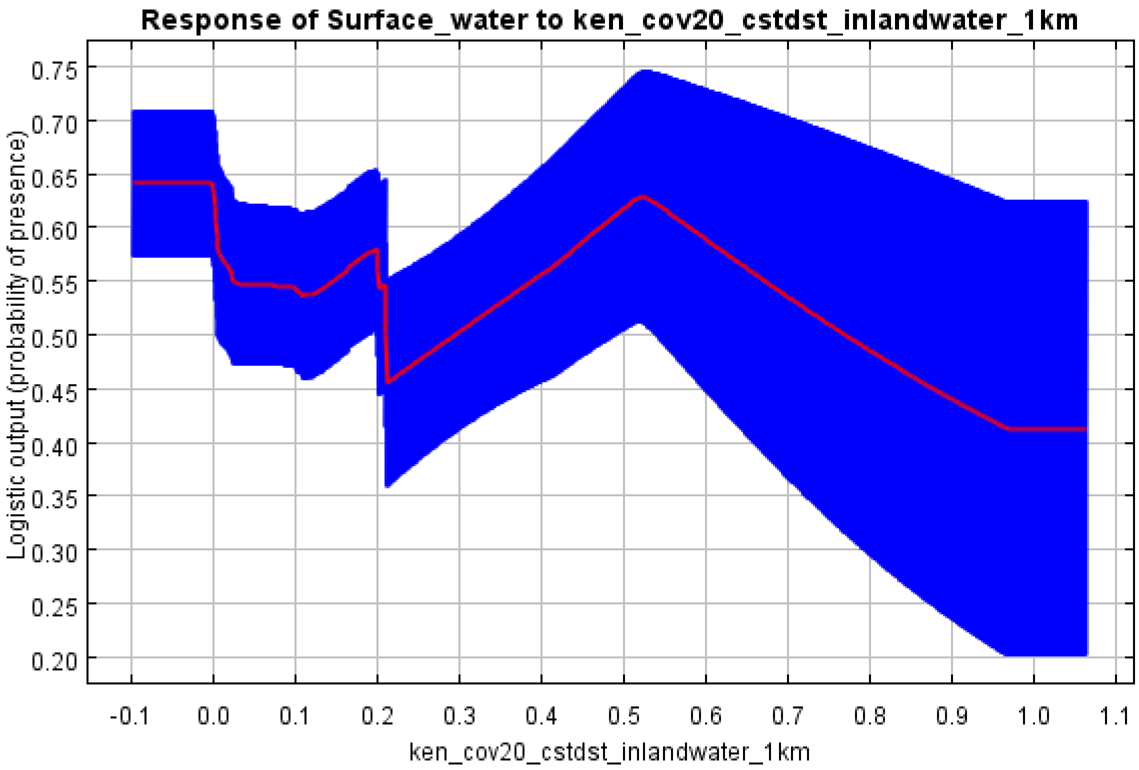

S1_Fig.129. Response curve of cost distance to inland water presented as means (red) of 50 replicate runs with standard deviation in blue; model built with other predictive covariates being kept at their average sample values. X-axis: cost distance (decimal degrees).

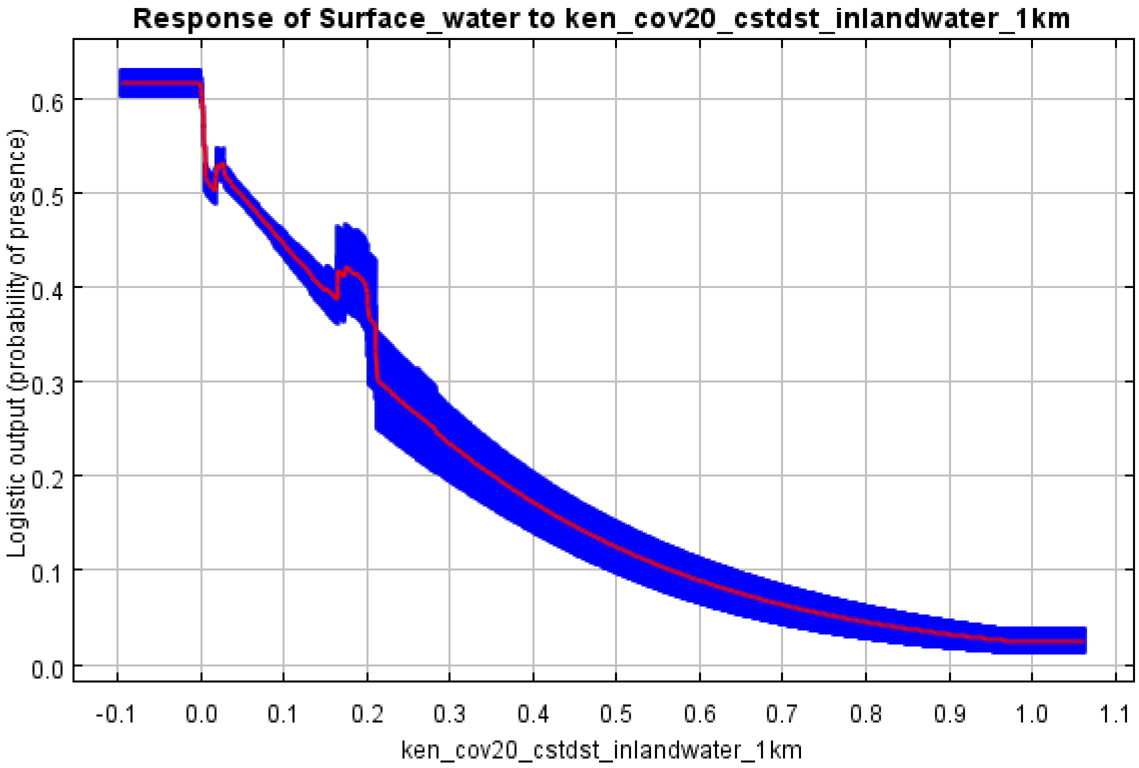

S1_Fig.130. Response curve of cost distance to inland water presented as means (red) of 50 replicate runs with standard deviation in blue; model built without other predictive covariates. X-axis: cost distance (decimal degrees).

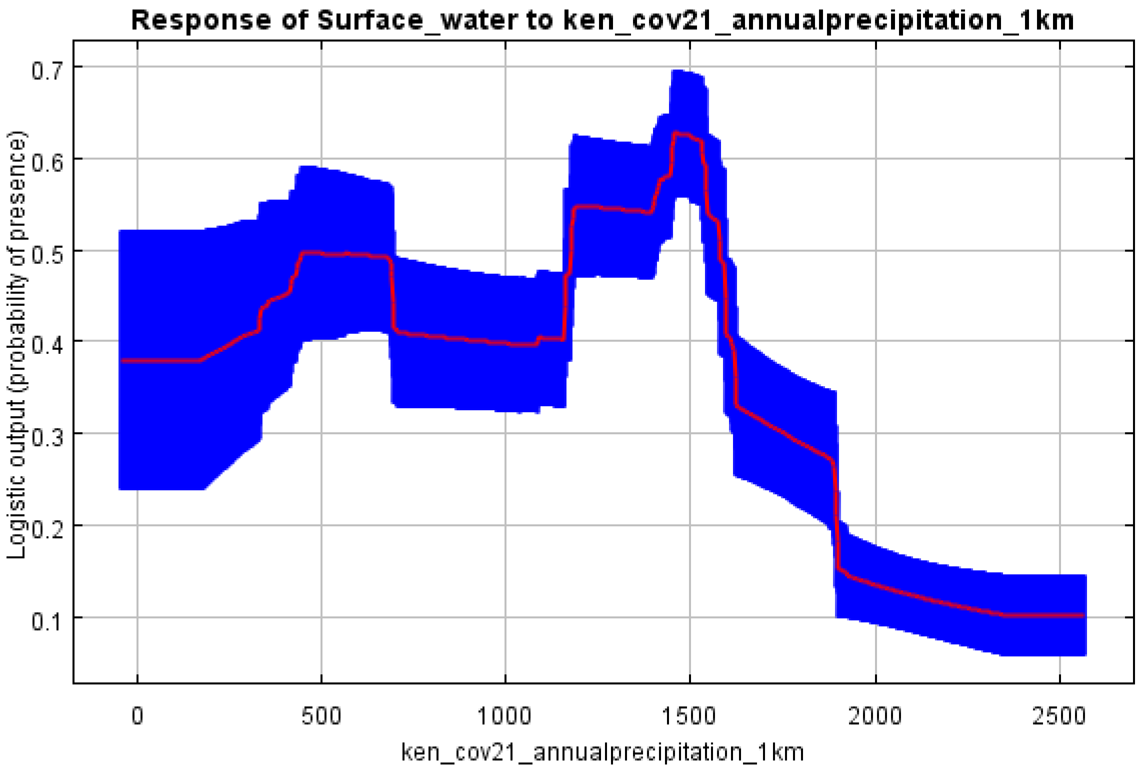

S1_Fig.131. Response curve of annual precipitation presented as means (red) of 50 replicate runs with standard deviation in blue; model built with other predictive covariates being kept at their average sample values. X-axis: annual precipitation (mm).

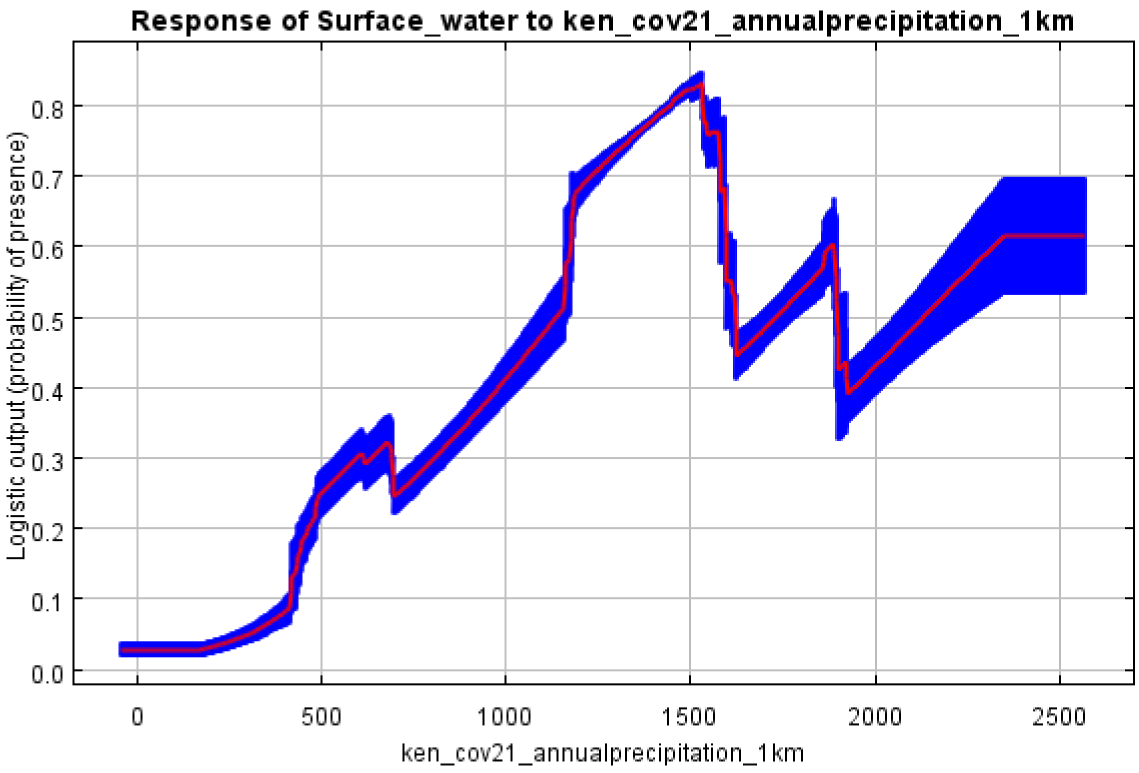

S1_Fig.132. Response curve of annual precipitation to inland water presented as means (red) of 50 replicate runs with standard deviation in blue; model built without other predictive covariates. X-axis: annual precipitation (mm).
